# The phylogenetics of Teleosauroidea (Crocodylomorpha, Thalattosuchia) and implications for their ecology and evolution

**DOI:** 10.7717/peerj.9808

**Published:** 2020-10-08

**Authors:** Michela M. Johnson, Mark T. Young, Stephen L. Brusatte

**Affiliations:** 1 School of GeoSciences, University of Edinburgh, Edinburgh, UK; 2 National Museum of Scotland, Edinburgh, UK

**Keywords:** Crocodylomorpha, Teleosauroidea, Phylogenetics, Nomenclature, Thalattosuchia, Ecomorphology, Jurassic, Vertebrate palaeontology, Taxonomy

## Abstract

Teleosauroidea was a clade of ancient crocodylomorphs that were a key element of coastal marine environments during the Jurassic. Despite a 300-year research history and a recent renaissance in the study of their morphology and taxonomy, macroevolutionary studies of teleosauroids are currently limited by our poor understanding of their phylogenetic interrelationships. One major problem is the genus *Steneosaurus*, a wastebasket taxon recovered as paraphyletic or polyphyletic in phylogenetic analyses. We constructed a newly updated phylogenetic data matrix containing 153 taxa (27 teleosauroids, eight of which were newly added) and 502 characters, which we analysed under maximum parsimony using TNT 1.5 (weighted and unweighted analyses) and Bayesian inference using MrBayes v3.2.6 (standard, gamma and variation). The resulting topologies were then analysed to generate comprehensive higher-level phylogenetic hypotheses of teleosauroids and shed light on species-level interrelationships within the clade. The results from our parsimony and Bayesian analyses are largely consistent. Two large subclades within Teleosauroidea are recovered, and they are morphologically, ecologically and biogeographically distinct from one another. Based on comparative anatomical and phylogenetic results, we propose the following major taxonomic revisions to Teleosauroidea: (1) redefining Teleosauridae; (2) introducing one new family and three new subfamilies; (3) the resurrection of three historical genera; and (4) erecting seven new generic names and one new species name. The phylogeny infers that the Laurasian subclade was more phenotypically plastic overall than the Sub-Boreal-Gondwanan subclade. The proposed phylogeny shows that teleosauroids were more diverse than previously thought, in terms of morphology, ecology, dispersal and abundance, and that they represented some of the most successful crocodylomorphs during the Jurassic.

## Introduction

Teleosauroid crocodylomorphs—distant extinct relatives of extant crocodylians (which include alligators, crocodiles, caimans and gavials)—were a near-globally distributed clade that frequented freshwater, brackish, lagoonal and deep-water marine ecosystems throughout the Jurassic (Buffetaut, 1982; Hua & Buffetaut, 1997; Hua, 1999; Young et al., 2014; Foffa, Young & Brusatte, 2015; Foffa et al., 2019; Johnson et al., 2015; Martin et al., 2016; Johnson et al., 2017, 2018; Johnson, Young & Brusatte, 2019). They have frequently been regarded as marine analogues of extant gavials, as the majority of species had an elongate and tubular snout, high tooth count and dorsally directed orbits, suggestive of a feeding style of catching small, fast-moving prey (Andrews, 1909, 1913; Buffetaut, 1982; Hua, 1999). Teleosauroids are part of the wider crocodylomorph clade Thalattosuchia, which also includes the metriorhynchoids: the only archosaurs to adopt a fully pelagic, open-ocean, swimming lifestyle in the manner of modern cetaceans (Young et al., 2010; Parrilla-Bel et al., 2013; Foffa & Young, 2014).

While teleosauroid skeletal and dental morphology has been well documented from the 18th Century to present (Chapman, 1758; Cuvier, 1824; Von Meyer, 1837; Eudes-Deslongchamps, 1867; Blake, 1876; Andrews, 1909, 1913; Westphal, 1961, 1962; Young et al., 2014; Johnson et al., 2017; Johnson, Young & Brusatte, 2019; Foffa et al., 2019; Sachs et al., 2019a), the evolutionary relationships of these crocodylomorphs are poorly understood and little studied. This is problematic, as phylogenies are crucial when evaluating evolutionary changes throughout time (Purvis, Gittleman & Brooks, 2005; Mishra & Thines, 2014). One of the major problems in teleosauroid systematics is the nomenclatural nightmare that is the taxon *Steneosaurus*. Widespread taxonomic lumping has seen this genus become a ‘wastebasket’ for a multitude of species. The validity of *Steneosaurus* has recently been called into question (Jouve et al., 2017; Johnson, Young & Brusatte, 2020) as the type specimen of the type species, *Steneosaurus rostromajor* Geoffroy Saint-Hilaire, 1825 (MNHN.RJN 134c-d), has rarely been referenced or figured in the literature since its preliminary descriptions by Cuvier (1800, 1808, 1812, 1824) and Geoffroy Saint-Hilaire (1825, 1831). Another problematic issue reinforced during the 20th Century (Andrews, 1909, 1913) is the contention that while there are noticeable differences between the skulls of teleosauroid species, the postcranial skeleton only shows superficial differences. This led to the assumption that teleosauroids must have lived in similar habitats with a conservative body plan (Andrews, 1913; Buffetaut, 1982). However, recent studies (Young et al., 2014; Johnson et al., 2017; Foffa et al., 2019; Martin et al., 2016, 2019; Wilberg, Turner & Brochu, 2019) have begun to dispute this notion, showing, in terms of postcranial anatomy and palaeoenvironment, that teleosauroids were more diverse than originally thought.

Herein we present an in-depth, comprehensive phylogenetic study of Teleosauroidea, using the most recently updated crocodylomorph dataset. We will: (1) explore the historical background of teleosauroid phylogenetics; (2) discuss the materials and phylogenetic methods used; (3) provide a novel, comprehensive taxonomic layout of Teleosauroidea; (4) list detailed descriptions of both newly scored and morphologically important characters; (5) evaluate the results of the phylogenetic analyses; and (6) elucidate what this new phylogeny implies about teleosauroid ecomorphological and distributional patterns.

## Historical Background

### Previous teleosauroid phylogenetics—late 1900s, early 2000s and Mueller-Töwe’s (2006) contributions

Although descriptions of teleosauroid fossils were prevalent during the mid-18th and 19th Centuries (Chapman, 1758; Morton & Wooller, 1758; Cuvier, 1808, 1812, 1824; Geoffroy Saint-Hilaire, 1825, 1831; Von Meyer, 1837; Eudes-Deslongchamps, 1867; Westphal, 1961), investigation into their evolutionary relationships remains a relatively new area of study. While Buffetaut (1980a, 1980b) and Vignaud (1995) briefly took note on the general interrelationships within Thalattosuchia, Benton & Clark (1988) examined the overall phylogenetic affinities of crocodylomorphs as a group. During the early 21st Century, thalattosuchians continued to be incorporated into larger crocodylomorph studies. However, these analyses were not focused on the interrelationships between thalattosuchians, and usually included only one or two teleosauroid taxa, namely *Steneosaurus bollensis* Jäger, 1828 and *Pelagosaurus typus* Bronn, 1841, which was considered a basal teleosauroid during that time (Gasparini, Pol & Spalletti, 2006; Pol & Gasparini, 2009).

Mueller-Töwe’s (2006) unpublished thesis included the first analysis that focused specifically on thalattosuchian phylogenetics, in particular Teleosauridae, and was built upon a preliminary study (Mueller-Töwe, 2005). Mueller-Töwe’s (2006) dataset included 189 characters, with twelve teleosauroids out of 29 taxa: *Machimosaurus hugii* Von Meyer, 1837; *Platysuchus multiscrobiculatus* (Berckhemer, 1929) Westphal (1961); *Steneosaurus baroni* Newton, 1893; *S*. *bollensis*; *Steneosaurus edwardsi* Eudes-Deslongchamps, 1868a; *Steneosaurus boutilieri* Eudes-Deslongchamps, 1868b; *Steneosaurus brevior* Blake, 1876; *Steneosaurus gracilirostris* Westphal, 1961; *Steneosaurus leedsi* Andrews, 1909 (which also incorporated *Mycterosuchus nasutus* Andrews, 1913); *Steneosaurus megarhinus* Hulke, 1871; *Steneosaurus obtusidens* Andrews, 1909; *Steneosaurus* (*Aeolodon*) *priscus* Von Sömmerring, 1814; and *Teleosaurus cadomensis* (Lamouroux, 1820). Other taxa were considered insufficient to include in the dataset (e.g. specimens that the author felt contained insufficient information and/or skeletal material), and only four teleosauroids used in the analysis were studied in-depth: *Pl*. *multiscrobiculatus*, *S*. *brevior*, *S*. *bollensis* and *S*. *gracilirostris* (note that Mueller-Töwe (2006) focused specifically on Toarcian species). In addition, there were no ordered or weighted characters, and multi-state characters were treated as polymorphs (Mueller-Töwe, 2006). Disregarding ordered or weighted characters, however, presents a problem, as ordered parsimony is less artefactual and susceptible to polarization errors, and displays an overall higher performance level (Mueller-Töwe, 2006) than unordered parsimony (Grand et al., 2013; Rineau et al., 2015).

**Figure 1:**
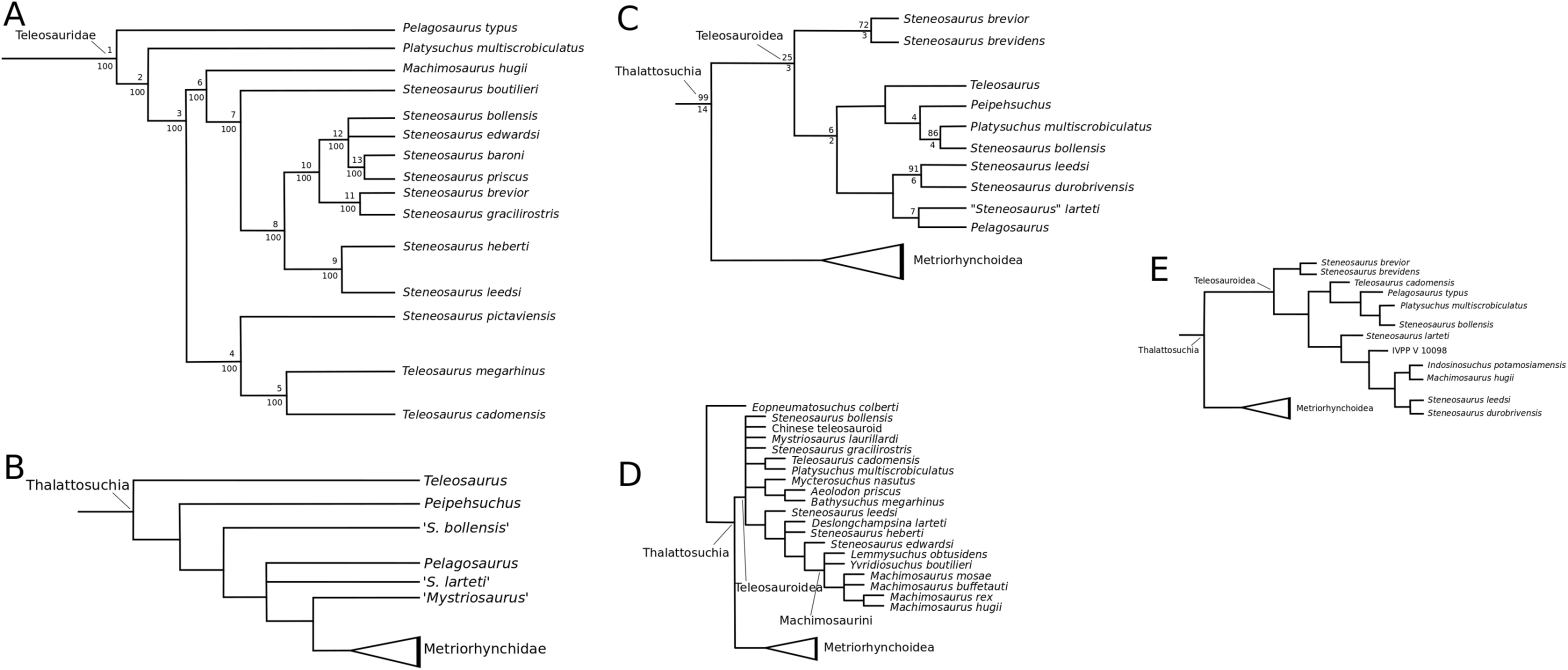
Previous thalattosuchian topologies. Recent strict consensus topologies focused on thalattosuchian phylogenetics, with attention to teleosauroids. Altered from (A) Mueller-Töwe (2006); (B) Jouve (2009); (C) Wilberg (2015b); (D) Johnson, Young & Brusatte (2019); and (E) Martin et al. (2019).

Mueller-Töwe’s (2006) strict consensus topology (Fig. 1A) produced 123 most parsimonious trees (MPTs) with a tree length of 423, an ensemble consistency index (CI) of 0.6312 and an ensemble retention index (RI) of 0.6549. The teleosauroids were found to be monophyletic and included: (1) *Pel. typus* as the basal-most teleosauroid; (2) a paraphyletic *Steneosaurus*; and (3) *Platysuchus* as the most closely related taxon to *Machimosaurus* (Fig. 1A). However, it is important to note that in Mueller-Töwe (2006) there are several factual errors and inconsistencies, particularly in the anatomical descriptions, which may have had an influence on the phylogenetic results. Note that as her final analyses were not subject to peer-review publication, it is unfair to give undue criticism.

When re-describing *T. cadomensis*, Jouve (2009) performed a phylogenetic analysis consisting of 75 taxa and 343 characters, and included the teleosauroids *Teleosaurus cadomensis*, *Peipehsuchus teleorhinus* Young, 1948 (now known as the Chinese teleosauroid IVPP V 10098), *S. bollensis*, *Pel. typus* (still considered to be a teleosauroid by some, although there was growing support for it as a metriorhynchoid: for example Buffetaut, 1980a; Mercier, 1933), *Steneosaurus larteti* Eudes-Deslongchamps, 1866 and ‘*Mystriosaurus*’ Kaup, 1834 (= *Pelagosaurus tomarensis*, MUHNAC unnumbered specimen: Telles-Antunes, 1967). The strict consensus (Fig. 1B) was found from four MPTs. Another study (Pierce, Angielczyk & Rayfield, 2009) conducted a parsimony analysis based off Mueller-Töwe’s (2006) unpublished character matrix; however, species they considered synonymous (e.g. *S*. *leedsi* and *S*. *megarhinus*) were combined and taxa not used in the authors’ landmark-based geometric morphometric analysis were deleted. Therefore, only seven teleosauroids were included (*Steneosaurus heberti* Morel de Glasville, 1876, *S*. *gracilirostris*, *Pl*. *multiscrobiculatus*, *Mac. hugii*, *S*. *leedsi*, *S*. *bollensis* and *S*. *brevior*), as well as *Pel. typus*, and *Metriorhynchus superciliosus* De Blainville, 1853 as the outgroup (Pierce, Angielczyk & Rayfield, 2009). This dataset produced two MPTs with 115 steps (CI = 0.621).

### The leisurely rise of teleosauroid phylogenetics—post-2010

Bronzati, Montefeltro & Langer (2012) presented an in-depth crocodylomorph supertree and included 19 teleosauroid species in their analysis; however, the Chinese teleosaurid (IVPP V 10098) was attributed to the metriorhynchoid *Peipehsuchus*; *S*. *edwardsi*, and *Steneosaurus durobrivensis* Andrews, 1909 (which is now considered a subjective junior synonym of *S*. *edwardsi*; see Johnson et al. (2015)) were treated as separate taxa; and *Steneosaurus pictaviensis* Vignaud, 1998, was included (which is a subjective junior synonym of *S*. *leedsi*; see below). Several key taxa were also absent in the analysis (e.g. *Myc. nasutus*, *S*. *obtusidens*, *Machimosaurus mosae* Sauvage & Liénard, 1879). In addition, Bronzati, Montefeltro & Langer (2012) searched for their source trees on Web of Science, other Internet search engines and published references, synthesizing published phylogenies and thus not personally examining the specimens. The result was a major polytomy of Teleosauroidea as a whole, with ‘*Mystriosaurus*’ and *Pl*. *multiscrobiculatus* unresolved at the base.

Wilberg (2015a) devised an updated crocodylomorph matrix (referred herein as the W matrix) which included nine teleosauroid taxa (*S*. *brevior*; *Steneosaurus brevidens* Phillips, 1871; ‘*Teleosaurus*’; *Mac. hugii*; *S*. *leedsi*; *S*. *durobrivensis*; *Pl*. *multiscrobiculatus*; *S*. *bollensis*; and *Peipehsuchus* [again considered a teleosauroid]). The strict consensus topology produced 566 MPTs and 1,649 steps (CI = 0.312; RI = 0.703) and a monophyletic teleosauroid clade, which continued to be stable regardless of different constraints placed on thalattosuchians as a whole (Wilberg, 2015a). This is somewhat similar to the results seen in follow-up studies by Wilberg (2015b) (Fig. 1C), Wilberg (2017) and Wilberg, Turner & Brochu (2019), and these produced comparable results to the recently updated Hastings+Young matrices (see below). However, there is one major change from Wilberg (2015a) to the updated results in Wilberg (2015b) and Wilberg, Turner & Brochu (2019): *Pel. typus* is now moved to the base of Metriorhynchoidea.

Recently, several new re-descriptions of teleosauroid taxa have begun to investigate crocodylomorph, notably thalattosuchian, phylogenetics (Foffa et al., 2019; Johnson, Young & Brusatte, 2019; Sachs et al., 2019a). In particular, a dataset known as the Hastings+Young (H+Y) dataset is being continuously updated to assess these evolutionary relationships. In 2016, Hastings and Young combined their respective crocodylomorph matrices to create this dataset, which acted as the foundation for the Crocodylomorph SuperMatrix Project. Ristevski et al. (2018), focusing on the interrelationhsips within goniopholidids, ran the first comprehensive version of this dataset, which included 14 thalattosuchians and three teleosauroids (*Pl*. *multiscrobiculatus*, *S. heberti* and *S. bollensis*). Ősi et al. (2018), describing the metriorhynchoid *Magyarosuchus fitosi*, ran an updated version of the H+Y matrix with 140 OTUs (operational taxonomic units) for 454 characters, resulting in 84 MPTs with 1,477 steps. Fifteen teleosauroids were included and Teleosauroidea was recovered as a monophyletic group, with *S*. *gracilirostris* as the basal-most teleosauroid and two distinct subgroups. When re-describing ‘*S*.’ *megarhinus*, Foffa et al. (2019) used a slightly modified version of the H+Y dataset: 140 OTUs, 18 of these teleosauroid taxa, for 456 characters, producing 85 MPTs with 1,494 steps (CI = 0.414, RI = 0.841). The strict consensus topology was similar to that found in Ősi et al. (2018) (*S*. *gracilirostris* as the basal taxon, two distinct subgroups), but showed different positions of certain taxa, most notably *Aeolodon priscus* and ‘*Teleosaurus*’ (*Bathysuchus*) *megarhinus*. In Johnson, Young & Brusatte (2019) and Sachs et al. (2019a), subsequent versions of the H+Y dataset were used; the phylogenetic analyses included 19 and 18 teleosauroid taxa, respectively, both producing an overall similar appearance of Teleosauroidea as that of Ősi et al. (2018) and Foffa et al. (2019). The H+Y dataset used in Johnson, Young & Brusatte (2019) included 143 OTUs for 464 characters, producing 201 MPTs with 1,526 steps (CI = 0.415; RI = 0.845) (Fig. 1D), whereas Sachs et al. (2019a) produced 197 MPCs and 1513 steps (CI = 0.417; RI = 0.846) from 142 OTUs for 462 characters.

Curiously, Martin et al. (2019) used Wilberg’s (2015a) dataset, with no explanation as to why they did not use one of the more recent versions of the Wilberg dataset then published (Wilberg, 2015b, Wilberg, 2017, or the W dataset in Ősi et al., 2018) or the most currently updated H+Y matrix (provided in Foffa et al. (2019) at that time). The W dataset (Wilberg, 2015a) was also used in Martin et al. (2016), again with no clarification as to why an updated W dataset (Wilberg, 2015b) was not used. Out of 78 OTUs, only 24 thalattosuchians (14 teleosauroids) were included (Martin et al., 2019), with similar taxonomic concerns found in Mueller-Töwe’s (2006) analysis. For example *S*. *durobrivensis* (= subjective junior synonym of *S*. *edwardsi*; Johnson et al., 2015) was treated as a distinct taxon, and many distinct species were excluded from the analysis. *Machimosaurus buffetauti* Young et al., 2015b (initially described as a valid taxon in Young et al. (2014)) was treated as *Mac. hugii* due to the monospecific hypothesis put forth in Martin & Vincent (2013) (for more information, see Foffa et al. (2019)). Furthermore, while *I. potamosiamensis* and *Mac. hugii* were coded in their entirety into the W matrix, three characters (174, 176 and 184) were altered from the original used by Wilberg (2015a), but only for the Chinese teleosauroid (IVPP V 10098) (Martin et al., 2019). Thus, the results (12 MPTs with 1666 steps) (Fig. 1E) were drastically different than those found in Wilberg (2015b), Young et al. (2016), Ristevski et al. (2018), Ősi et al. (2018), Foffa et al. (2019), Johnson, Young & Brusatte (2019) and Sachs et al. (2019a).

## Methods

### Objectives and taxonomic sample

Our phylogenetic analysis focused specifically on valid Teleosauroidea taxa, which range from the Early Jurassic (lower Toarcian, for example *Steneosaurus gracilirostris*) to the Early Cretaceous (*Machimosaurus rex* Fanti et al., 2016). The current dataset is a newly modified version of the H+Y dataset. It has since grown substantially over the past three years, with the addition of new taxa and characters. It was first presented in Ristevski et al. (2018) and has been updated subsequently since then (Ősi et al., 2018; Foffa et al., 2019; Johnson, Young & Brusatte, 2019; Sachs et al., 2019a, 2019b).

Our taxonomic sample consisted of 153 crocodylomorph taxa (OTUs) with *Postosuchus kirkpatricki* Chatterjee, 1985 as the outgroup taxon. Eighty OTUs are thalattosuchians, and 27 of these are teleosauroids, listed as follows: ‘*Steneosaurus*’ *gracilirostris*; *Mystriosaurus laurillardi* Kaup, 1834; ‘*Steneosaurus*’ *stephani* Hulke, 1877; the Chinese teleosauroid IVPP V 10098 previously referred to as *Peipehsuchus teleorhinus* (Li, 1993); *Indosinosuchus potamosiamensis* Martin et al., 2019; *Indosinosuchus kalasinensis* sp. nov. (see below); ‘*Steneosaurus*’ *baroni*; *Platysuchus multiscrobiculatus*; *Teleosaurus cadomensis*; *Mycterosuchus nasutus*; *Bathysuchus megarhinus*; ‘*Steneosaurus*’ *bollensis*; ‘*Steneosaurus*’ *leedsi*; *Sericodon jugleri* Von Meyer, 1845; *Aeolodon priscus*; ‘*Steneosaurus*’ *megistorhynchus* Eudes-Deslongchamps, 1866; *Yvridiosuchus boutilieri* (Eudes-Deslongchamps, 1868b) Johnson, Young & Brusatte, 2019; *Deslongchampsina larteti* (Eudes-Deslongchamps, 1866) Johnson, Young & Brusatte, 2019; ‘*Steneosaurus*’ *bouchardi* Sauvage, 1872; ‘*Steneosaurus*’ *heberti*; *Steneosaurus rostromajor* Geoffroy Saint-Hilaire, 1825; ‘*Steneosaurus*’ *edwardsi*; *Lemmysuchus obtusidens*; *Machimosaurus buffetauti*; *Machimosaurus mosae*; *Machimosaurus hugii*; and *Machimosaurus rex*. Certain taxa were excluded from the dataset, being either fragmentary, lost or correspondent with known species (see discussion below). First-hand examination of all aforementioned teleosauroid taxa (excluding ‘*S*.’ *bouchardi* and certain *Ser. jugleri* specimens) by MM Johnson resulted in the modification of the dataset. The differences between this dataset and that provided in the most recently updated H+Y analysis (Johnson, Young & Brusatte, 2019) are as follows:

1. Eight new taxa were added: ‘*S*.’ *stephani*, *I. potamosiamensis*, *I. kalasinensis* sp. nov., *Ser*. *jugleri*, ‘*S*.’ *bouchardi*, ‘*S*.’ *baroni*, ‘*S*.’ *megistorhynchus* and *S*. *rostromajor*.
2. Generic names were changed for three previously included taxa (*Yvridiosuchus*, *Bathysuchus* and *Deslongchampsina*).
3. *Steneosaurus brevior* was changed to *Mystriosaurus laurillardi* following Sachs et al. (2019a).
4. All characters of all remaining teleosauroid taxa were re-examined and re-scored.
5. The number of characters increased from 464 to 502 (new characters 12, 13, 15, 43, 56, 58, 64, 124, 125, 167, 184, 208, 269, 270, 291, 292, 293, 294, 295, 296, 297, 339, 340, 394, 395, 396, 398, 417, 430, 431, 434, 438, 449, 456, 459, 464, 466 and 489).
6. Characters 32 and 36 were re-written.
7. Character 27 was re-written and re-defined.
8. Characters 47 and 48 were re-written and re-scored, referring to characteristics of the pholidosaurid ‘beak’ (ch. 47) and teleosauroid premaxilla (ch. 48).
9. 19 additional characters were ordered (49, 57, 85, 101, 107, 178, 179, 203, 241, 256, 257, 309, 410, 408, 414, 447, 452, 457 and 471).
10. Two non-teleosauroid taxa were excluded (*Eoneustes bathonicus* (Mercier, 1933) Young et al., 2010; and Geosaurine indeterminate from Argentina) and four were included (the early crocodylomorph *Carnufex carolinensis* Zanno et al., 2015; Metriorhynchoid indeterminate T; *Maledictosuchus nuyivijanan* Barrientos-Lara, Alvarado-Ortega & Fernández, 2018; and Swiss ‘*Metriorhynchus hastifer*’).

### Character sampling and scoring

The foundation of our character sampling is the H+Y dataset, which initially included 387 characters (Ristevski et al., 2018), with 289 dental+craniomandibular, 95 post-cranial and three soft tissue. Ősi et al. (2018) contained 454 characters (334 dental+craniomandibular, 116 post-cranial and four soft tissue); Foffa et al. (2019) incorporated 456 characters (336 dental+craniomandibular, 116 postcranial and four soft tissue); Johnson, Young & Brusatte (2019) included 464 characters (339 dental+craniomandibular, 120 post-cranial and five soft tissue); Sachs et al. (2019a) incorporated 462 characters (337 dental+craniomandibular, 120 post-cranial and five soft tissue); and Sachs et al. (2019b) used 460 characters (337 dental+craniomandibular, 118 post-cranial and five soft tissue).

In our updated version of the H+Y dataset, 38 new characters were added (362 dental+craniomandibular, 135 post-cranial and 5 soft tissue). The complete character list comprises of 502 characters, including 286 craniomandibular (57%), 76 dental (15%), 135 post-cranial (27%) and 5 soft tissue (1%). Out of 502 characters, 45 were treated as ordered: 7, 26, 39, 47, 49, 59, 62, 71, 85, 101, 107, 112, 178, 179, 181, 183, 193, 203, 224, 241, 242, 250, 256, 257, 282, 301, 309, 359, 385, 388, 397, 408, 409, 410, 414, 447, 450, 452, 453, 457, 467, 468, 470, 471 and 482. The characters were scored based on first-hand examination of numerous teleosauroid specimens. Additional, unavailable or lost specimens pertaining to *Mac. hugii*, *Mac. mosae* and *Sericodon* were also examined from photographs (Hua, 1999; Lepage et al., 2008; Young et al., 2014; Schaefer, Püntener & Billon-Bruyat, 2018), and photographs of ‘*S*.’ *bouchardi* were provided by Y. Lepage. In addition, multiple *Steneosaurus* sp., *Machimosaurus* sp., *Teleosaurus* sp. and Teleosauroidea indeterminate specimens were examined. Overall, approximately 550 teleosauroid specimens were personally studied by MM Johnson.

The complete list of 502 characters are presented the Supplemental Material SD1, similar to Ősi et al. (2018), Foffa et al. (2019), Johnson, Young & Brusatte (2019) and Sachs et al. (2019a, 2019b). Newly added characters are represented by (NEW), ordered characters are specified by (ORDERED), and characters that cannot be scored (e.g. are inapplicable) for all taxa are marked with an asterisk (*) following the character descriptions. Additional comments and references are included, and characters are organized in the following anatomical order:

1. Skull geometry and dimensions
2. Craniomandibular ornamentation
3. Internal neuroanatomy, sensory systems and cranial exocrine glands
4. Craniomandibular pneumaticity
5. Rostral neurovascular foramina
6. Cranial rostrum
7. Skull roof
8. Orbit and temporal region
9. Palate and perichoanal structures
10. Occipital
11. Braincase, basicranium and suspensorium
12. Mandibular geometry
13. Mandible
14. Dentition and alveolar morphologies
15. Axial post-cranial skeleton
16. Appendicular skeleton: pectoral girdle and forelimbs
17. Appendicular skeleton: pelvic girdle and hind limbs
18. Dermal ossifications: osteoderms
19. Dermal ossifications: gastralia
20. Soft tissue

### Methodology

Our dataset, which includes 153 OTUs and 502 characters, was analysed by conducting unweighted and weighted maximum parsimony analyses using TNT 1.5 Willi Hennig Society Edition (Goloboff, Farris & Nixon, 2008; Goloboff & Catalano, 2016), following previous iterations (Ősi et al., 2018; Foffa et al., 2019; Johnson, Young & Brusatte, 2019; Sachs et al., 2019a, 2019b).

Our dataset was analysed as previously described in Foffa et al. (2019), Johnson, Young & Brusatte (2019) and Sachs et al. (2019a, 2019b). Specifically, memory settings were increased with General RAM set to 900 Mb and the maximum number of trees to be held set to 99,999. Cladogram space was searched by means of the ‘New Technology search’ option in TNT (Sectorial Search, Ratchet, Drift, and Tree fusing) with 1,000 random-addition replicates (RAS). The trees were then subjected to a Traditional Search, with ‘tree bisection reconnection’ (TBR) branch swapping, using 1,000 replications and 10 trees saved per replication. In addition, the default setting was increased for the iterations of each method (except for Tree fusing, which was kept at three rounds). In the Sectorial Search, 1,000 Drift cycles (for selections of above 75) were run, as well as 1,000 starts and fuse trees (for selections below 75) and 1,000 rounds of Consensus Sectorial Searches (CSSs) and Exclusive Sectorial Searches (XSSs). For Ratchet, the program used 1000 ratchet iterations set to stop the perturbation when 1,000 substitutions were made or 99% of the swapping was reached. Lastly, in Drift, the analysis included 1,000 Drift cycles set to stop the perturbation when 1,000 substitutions were made or 99% of the swapping was reached. The collapsing rule used was 50%, and Bremer support values of 10 were also computed which measure branch support and indicate the number of extra steps required for a clade to collapse (Bremer, 1988; Müller, 2004). In addition, a majority rules unweighted consensus (50% cut-off) was examined, as it summarizes a specific collection of MPTs (Holder, Sukumaran & Lewis, 2008). The analysis was run again using implied weighing (*k* = 12), with the ‘New Technology search’ options (Sectorial Search, Ratchet, Drift and Tree fusing) with the same settings as outlined above.

In addition, our dataset was also analysed under Bayesian inference using MrBayes v3.2.6 (Huelsenbeck & Ronquist, 2001; Huelsenbeck et al., 2001; Ronquist & Huelsenbeck, 2003; Ronquist et al., 2012). While Bayesian methods are generally more popular when using molecular phylogenetics, they are becoming more common in morphological studies, including those involving fossil data (Lewis, 2001; Prieto-Márquez, 2010; Slater, 2013; Brusatte & Carr, 2016). We chose to run our dataset in MrBayes to compare its results with that of the unweighted and weighted topologies in TNT. The Markov (Mk) model of Lewis (2001) was used, with three different variations applied. The first was a generalized test, using the default setting of MrBayes: this is the simplest model, in that all substitutions have the same rate or involves equal rates of character change (*rates = equal*). The second involved a gamma parameter distribution with four rate categories (*rates = gamma ngammacat = 4*), which allows for differing rates of character change. The *rates = gamma* refers to gamma distribution rates across sites, and *ngammacat* sets the number of rate categories for the gamma distribution. The third involves a slightly different gamma parameter distribution (*lset applyto = (1) coding = variable rates = gamma*). This test specifies how characters are sampled, with *variable* indicating that only variable characters have the possibility of being sampled. In all three analyses, four chains were used and ran for 4,000,000 generations, sampled every 100 generations. Trees that were generated during the first 20,000 generations were disregarded as ‘burn in’.

## Systematic Palaeontology—genus and Species Level Taxonomy

As mentioned previously, the most historically important and commonly utilized teleosauroid genus *Steneosaurus* has been recognized as a ‘wastebasket’ taxon by researchers and has continuously been recovered as paraphyletic or polyphyletic in phylogenetic analyses (Mueller-Töwe, 2006; Wilberg, 2015b; Foffa et al., 2019; Johnson, Young & Brusatte, 2019). In addition, no type species had until recently been officially designated for *Steneosaurus* under International Commission on Zoological Nomenclature (ICZN) Code rules. Johnson, Young & Brusatte (2020) set out to rectify this problem by evaluating the validity of *Steneosaurus*. The authors designated *Steneosaurus rostromajor* Geoffroy Saint-Hilaire, 1825, as the type species of *Steneosaurus*, designated MNHN.RJN 134c-d as the lectotype, provided a thorough literature and descriptive review of the specimen, and compared it with other relevant teleosauroid taxa. Their final verdict considered *S. rostromajor* (MNHN.RJN 134c-d) to be a nomen dubium, and proposed that the genus *Steneosaurus* is undiagnostic, due to (1) lack of autapomorphic characters (2) poor preservation (3) a generic concept that has changed multiple times through time; and (4) uncertainty of teleosauroid ontogenetic variation and sexual dimorphism (Johnson, Young & Brusatte, 2020).

Johnson, Young & Brusatte (2020) suggested that establishing a ‘clean’ foundation of teleosauroid taxonomy using diagnostic type species/specimens, with every nomenclatural act correctly formulated, was the next course of action. Therefore, we believe that it is necessary to erect new proposed teleosauroid genera first, as a direct result of the proposal of *Steneosaurus* as a nomen dubium.

This article in Portable Document Format (PDF) signifies a published work in accordance with the ICZN. As such, the new genus and species names contained will be effectively published under ICZN Code from the electronic edition. This work and the nomenclatural acts contained within it have been registered in ZooBank, the online registration system for the ICZN. The following ZooBank LSIDs (Life Science Identifiers) and associated information may be viewed through a standard web browser by adding the LSID to the prefix http://zoobank.org/. The LSID for this publication is: urn:lsid:zoobank.org:pub:7CC3CA17-F08F-48AD-9F16-8537B6BAAC1F.

CROCODYLOMORPHA Hay, 1930 (sensu Nesbitt, 2011)

THALATTOSUCHIA Fraas, 1901 (sensu Young & Andrade, 2009)

TELEOSAUROIDEA Geoffroy Saint-Hilaire, 1831 (sensu herein, see below)

*Plagiophthalmosuchus* **gen. nov.**

**Type species**—*Steneosaurus gracilirostris* Westphal, 1961. Now referred to as *Plagiophthalmosuchus gracilirostris* (Westphal, 1961), **comb. nov**. urn:lsid:zoobank.org:act:1AC91E3C-FC9A-470B-B9A9-3220B9823C0F

**Etymology**—‘Lateral-eyed crocodile.’ *Plágios* (πλάγιος) and *ofthalmós* (οφθαλμός) are Greek for ‘lateral’ and ‘eye’, respectively (referring to the laterally directed orbits of this taxon); *suchus* is the Latinized form of the Greek *soukhos* (σοῦχος), meaning crocodile.

**Diagnosis**—same as the only known species (monotypic genus).

*Plagiophthalmosuchus gracilirostris* (Westphal, 1961) **comb. nov.**

(Fig. 2)

**Figure 2:**
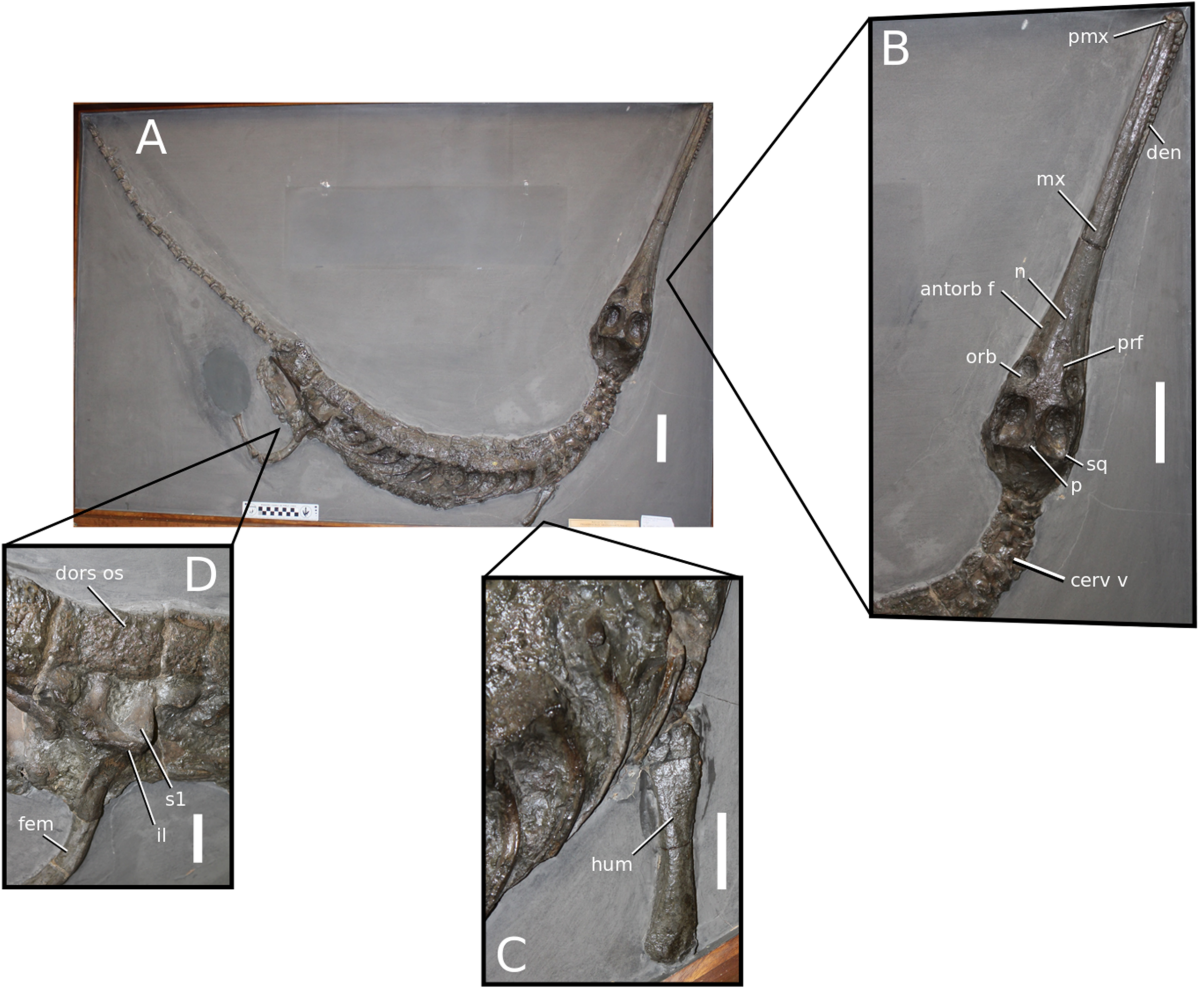
*Plagiophthalmosuchus gracilirostris*. *Plagiophthalmosuchus gracilirostris* (Westphal, 1961) **comb. nov.**, NHMUK PV OR 14792, holotype. (A) Nearly complete skeleton, with close-up views of: (B) the skull, (B) forelimb and (D) pelvic area. Refer to abbreviations list. Scale bars: 10 cm (A and B) and 4 cm (C and D).

**Holotype**—NHMUK PV OR 14792, a nearly complete skeleton.

**Paratype**—NHMUK PV OR 15500, a complete skull and mandible.

**Referred material**—DONMG specimen (nearly complete skull and mandible); MNHNL TU515 (nearly complete skull and mandible); YORM 2012.38 (nearly complete skull).

**Age**—early Toarcian, Early Jurassic.

**Localities**—Whitby, Yorkshire, UK; Dudelange-Bettembourg, southern Luxembourg.

**Stratigraphic horizons**—Alum Shale Member, Whitby Mudstone Formation, Lias Group; *Harpoceras serpentinum* ammonite Zone (‘*schistes bitumineux*’).

**Scoring Sources**—the holotype (NHMUK PV OR 14792), paratype and all referred specimens were studied first-hand. Photographs of DONMG were provided by D. Lomax.

**Autapomorphic characters of *Pla. gracilirostris***—in the antorbital fenestra, the external fenestra is significantly larger than internal fenestra (over 25%); antorbital fenestra is moderately large, being at least half the diameter of the orbit; internal fenestra is approximately 50% of the length of the orbit; supratemporal fossa is slightly larger (~25%) than the length of the orbit; basioccipital sub-vertical and somewhat visible in occipital view; exoccipital-opisthotics are dorsoventrally slender and paraoccipital processes have a straight distal margin; orbit positioned laterally with a slight dorsal inclination; dorsal border at dentary-surangular is relatively straight; glenoid fossa of the articular oriented subtly anterodorsally.

**Emended diagnosis**—longirostrine snout; tooth row and quadrate condyle aligned, both at a lower level than the occipital condyle (shared with *Macrospondylus*); ornamentation absent on prefrontal (shared with *I. potamosiamensis*, *Aeolodon*, *Bathysuchus* and *Sericodon*) and lacrimal (shared with *I. potamosiamensis*, *Sericodon*, *Aeolodon* and *Macrospondylus*); greater than 67% of the total premaxilla length is posterior to the external nares (similar to the Chinese teleosauroid, *I. potamosiamensis*, *Mycterosuchus*, *Aeolodon*, *Bathysuchus* and *Sericodon*); external nares oriented anterodorsally (shared with *Indosinosuchus*, the Chinese teleosauroid, *Teleosaurus*, *Platysuchus*, *Mycterosuchus*, *Aeolodon*, *Bathysuchus* and *Sericodon*); premaxilla anterior and anterolateral margins are not sub-vertical (shared with *Macrospondylus*, *Andrianavoay*, *Charitomenosuchus*, *Deslongchampsina*, *Proexochokefalos*, *Neosteneosaurus* and Machimosaurini); antorbital fenestra is anteroposteriorly elongated (similar to *Deslongchampsina*); frontal broader than orbital width (shared with *Mystriosaurus*, *Platysuchus*, *Teleosaurus*, *Mycterosuchus*, *Bathysuchus*, *Aeolodon*, *Pr*. cf. *bouchardi*, *Neosteneosaurus*, *Mac. buffetauti* and *Mac. mosae*); squamosal projects further posteriorly than the occipital condyle (shared with the Chinese teleosauroid, *Neosteneosaurus*, *Yvridiosuchus*, *Lemmysuchus* and *Mac. mosae*); orbit longitudinal ellipsoid in shape; basioccipital tubera reduced (shared with *Mycterosuchus*, *Bathysuchus* and *Sericodon*); supraoccipital dorsoventrally tall (shared with *Clovesuurdameredeor*, *Andrianavoay* and *Lemmysuchus*); angular straight and mainly horizontal, especially the anterior part (shared with *Mystriosaurus*); ventral margin of mandible is poorly curved (shared with *Mystriosaurus*); proximal humerus expanded and hooked (similar to *Platysuchus* and *Teleosaurus*); tibia evidently shorter than the femur (shared with *Platysuchus*).

*Mystriosaurus* Kaup, 1834

**Type species**—*Mystriosaurus laurillardi* Kaup, 1834.

**Etymology**—‘Spoon lizard’. *Mystrio* refers to the spoon-shaped anterior rostrum in dorsal view, and *saurus* is the Latinized version of *saûros* (σαυρoς), which is Ancient Greek for lizard.

**Diagnosis**—same as the only known species (monotypic genus).

*Mystriosaurus laurillardi* Kaup, 1834

(Fig. 3)

**Figure 3:**
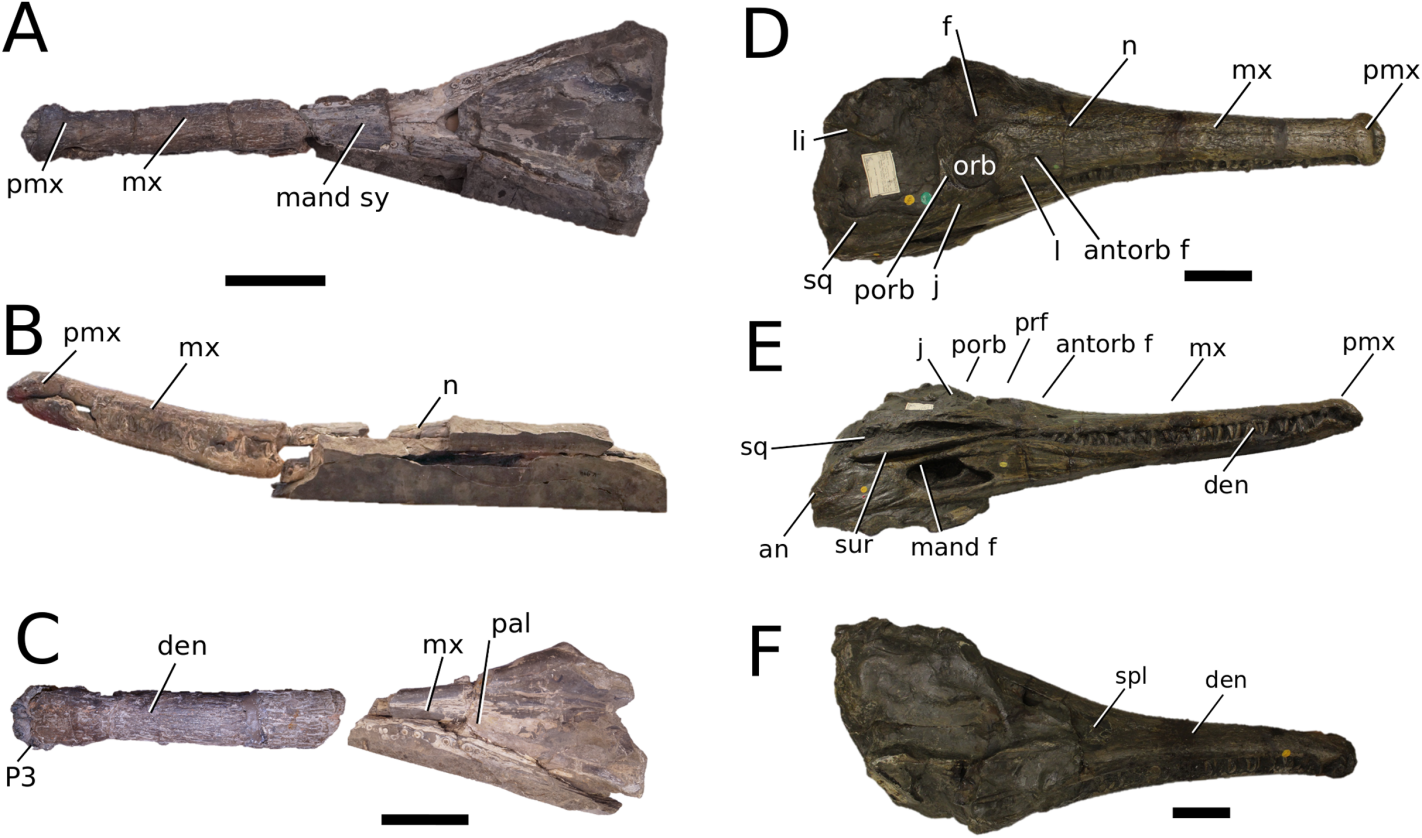
*Mystriosaurus laurillardi*. *Mystriosaurus laurillardi* Kaup, 1834, holotype HLMD V946-948 (A–C) and referred specimen NHMUK PV OR 14781 (D–F). (A and D) Dorsal, (B) left lateral, (C and F) ventral and (E) right lateral views. Refer to abbreviations list. Scale bars: 10 cm. Photographs A to C provided by S. Sachs.

**Holotype**—HLMD V946-948, a partial skull.

**Referred material**—NHMUK PV OR 14781 (nearly complete skull and mandible), holotype of *Steneosaurus brevior*.

**Age**—*Harpoceras serpentinum* Sub-Boreal ammonite Zone, early Toarcian, Early Jurassic.

**Localities**—Altdorf, Germany; Whitby, Yorkshire, UK.

**Stratigraphic horizons**—Posidonia Shale Formation; Mulgrave Shale Member, Whitby Mudstone Formation, Lias Group.

**Scoring sources**—NHMUK PV OR 14781 was studied first-hand. The holotype (HLMD V946-948) was examined using high quality photographs provided by S. Sachs, and also discussed at great length with S. Sachs.

**Autapomorphic characters of *Mys. laurillardi***—well-developed and extensive ornamentation on the nasals; external nares oriented anteriorly; antorbital fenestra is sub-rectangular in shape; supratemporal fossae form an approximate isosceles trapezoid-shape; medial margin of supratemporal arch relatively straight in dorsal view, with no significant concavity; prominent anterior notch in the dentaries; mandibular fenestra poorly elliptic; large robust teeth with numerous, conspicuous apicobasally aligned enamel ridges and a pointed apex, with more anteriorly-placed tooth crowns being procumbent.

**Emended diagnosis**—mesorostrine skull; well-developed and extensive ornamentation on the premaxillae, maxillae, frontal, prefrontal, lacrimal and postorbital; frontal ornamentation composed of small sub-circular to elongate pits that are closely spaced or, that can fuse and become a ridge-groove pattern (similar to *Mycterosuchus*); slight constriction of the snout anterior to the orbits (similar to *Deslongchampsina*); large and numerous neurovascular foramina on the premaxillae, maxillae and dentaries (shared with Machimosaurini); external nares 8-shaped in dorsal view (shared with the Chinese teleosauroid, *I. potamosiamensis*, *Bathysuchus* and *Aeolodon*); dorsoventrally deep premaxilla (similar to *I. kalasinensis*); anteroposterior premaxilla length less than 25% of total rostral length (shared with the Chinese teleosauroid, *Mac. buffetauti* and *Mac. mosae*); premaxilla anterior and anterolateral margins are orientated anteroventrally and extend ventrally in lateral view (shared with the Chinese teleosauroid, *Indosinosuchus*, *Platysuchus*, *Mycterosuchus*, *Aeolodon*, *Bathysuchus* and *Sericodon*); antorbital fenestrae almost equidistant to orbit and alveolar margin (shared with *Platysuchus*); antorbital fenestra is large relative to orbits, where the anteroposterior length is approximately 25% orbital anteroposterior length (similar to *Plagiophthalmosuchus* and *Deslongchampsina*); anterolateral margin of supratemporal fossae noticeably inclined anterolaterally (shared with the Chinese teleosauroid, *Indosinosuchus*, *Platysuchus*, *Teleosaurus*, *Mycterosuchus*, *Aeolodon*, *Bathysuchus* and *Sericodon*); the anterior region of the supratemporal fenestra has well-rounded lateral and medial margins; frontal width broader than orbital width (shared with *Plagiophthalmosuchus*, *Platysuchus*, *Teleosaurus*, *Mycterosuchus*, *Aeolodon*, *Bathysuchus*, *Sericodon, Pr*. cf. *bouchardi*, *Neosteneosaurus*, *Mac. buffetauti* and *Mac. mosae*); short frontal anteromedial process, (similar to *Clovesuurdameredeor*); orbits subcircular in shape and dorsolaterally orientated; postorbital reaches orbit posteroventral margin (shared with the Chinese teleosauroid, *I. potamosiamensis*, *Platysuchus*, *Teleosaurus* and *Mycterosuchus*); mandibular symphysis slightly less than half the mandibular length, between 45 and 50% (similar to *I. potamosiamensis*, *Deslongchampsina* and *Proexochokefalos*); deep, well-developed reception pits throughout the anterior- to mid-maxilla and gradually disappear (similar to *Charitomenosuchus*, *Deslongchampsina* and *Proexochokefalos*); ventral border of angular horizontal and poorly curved, especially the anterior part (similar to *Plagiophthalmosuchus*); four teeth per premaxilla; maxillary alveolar count at least 29 (modified from Young & Steel, in press) (similar to the Chinese teleosauroid, *I. potamosiamensis*, *Neosteneosaurus*, *Yvridiosuchus* and *Mac. buffetauti*); dentary alveolar count approximately 30 to 33 alveolar pairs; P1 and P2 both oriented anteriorly (shared with *I. potamosiamensis*, *Platysuchus*, *Macrospondylus*, *Deslongchampsina*, *Neosteneosaurus*, *Yvridiosuchus* and *Lemmysuchus*).

*Clovesuurdameredeor* **gen. nov.**

**Type species**—*Steneosaurus stephani* Hulke, 1877. Now referred to as *Clovesuurdameredeor stephani* (Hulke, 1877), **comb. nov**. urn:lsid:zoobank.org:act:B9FC0E91-9153-4F6B-B4B7-817839A9E7DD

**Etymology**—‘Clovesuurda’s sea creature’. *Clovesuurda* was the Medieval Latin name of the village of Closworth (written in the Doomsday Book of 1086), the locality where the holotype was found; *meredēor* is Old English for ‘sea creature’.

**Diagnosis**—same as the only known species (monotypic genus).

*Clovesuurdameredeor stephani* (Hulke, 1877) **comb. nov.**

(Fig. 4)

**Figure 4:**
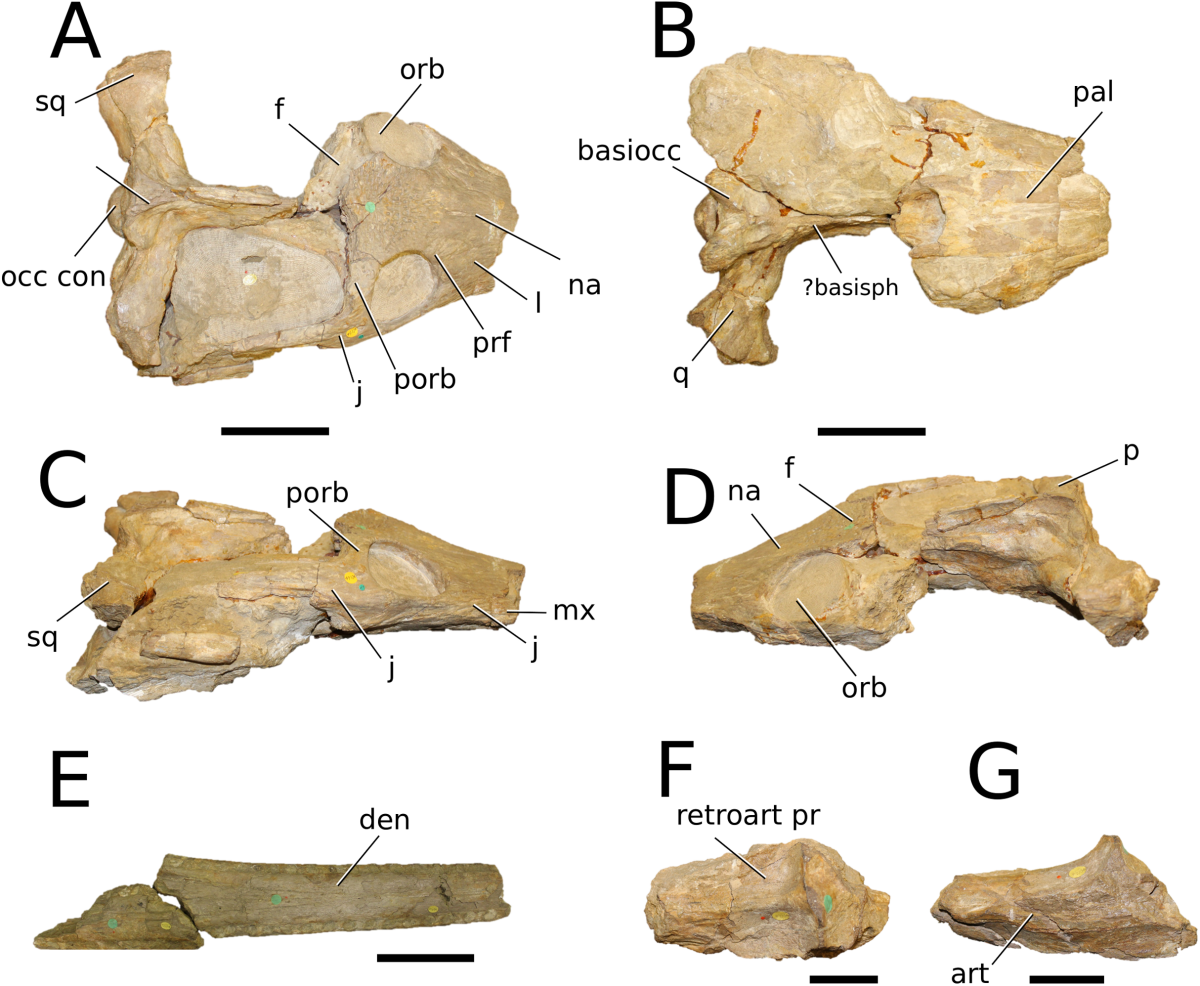
*Clovesuurdameredeor stephani*. *Clovesuurdameredeor stephani* (Hulke, 1877), **comb. nov.**, NHMUK PV OR 49126, holotype. Skull in (A) dorsal, (B) ventral (palatal), (C) right and (D) left lateral views. Partial mandible in (E) dorsal view, and right retroarticular process in (F) dorsal and (G) right lateral views. Refer to abbreviations list. Scale bars: 10 cm (A–C) and 4 cm (E–F).

**Holotype**—NHMUK PV OR 49126, a partial skull and anterior section of mandible.

**Age**—Bathonian, Middle Jurassic.

**Locality**—Closworth, Dorsetshire, UK.

**Stratigraphic horizon**—Great Oolite Group, Cornbrash Formation.

**Scoring sources**—the holotype (NHMUK PV OR 49126) was examined first-hand.

**Autapomorphic characters of *Cl. stephani***—prefrontal is anteroposteriorly short and mediolaterally broadened; posterior projections of the nasals not elongated and level with prefrontal-orbit contact in dorsal view; anteromedial process of the frontal is posterior to the prefrontals; anteromedial process of the frontal is anteroposteriorly short and mediolaterally broad; jugal extends anteriorly to the prefrontal.

**Emended diagnosis**—frontal ornamentation extends from the centre to the lateral- and anterior-most areas (shared with *Plagiophthalmosuchus*, the Chinese teleosauroid, *Indosinosuchus*, *Platysuchus*, *Teleosaurus*, *Mycterosuchus* and *Macrospondylus*); presence of small antorbital fenestrae; no anterolateral expansion or inclination of the supratemporal fenestrae (shared with *Plagiophthalmosuchus*, *Macrospondylus*, *Charitomenosuchus*, *Seldsienean*, *Deslongchampsina*, *Proexochokefalos*, *Neosteneosaurus* and Machimosaurini); frontal subequal to orbital width (shared with the Chinese teleosauroid, *I. kalasinensis*, *Macrospondylus*, *Charitomenosuchus*, *Deslongchampsina*, *Proexochokefalos*, *Yvridiosuchus*, *Mac. hugii* and *Mac. rex*); circular orbits (shared with *Mystriosaurus*, *Indosinosuchus*, *Teleosaurus*, *Mycterosuchus*, *Sericodon*, *Lemmysuchus* and *Machimosaurus*); anterior process of the jugal is slender and elongated (shared with *Charitomenosuchus*, *Proexochokefalos*, *Neosteneosaurus* and Machimosaurini).

The Chinese teleosauroid previously referred to *Peipehsuchus teleorhinus* Young, 1948 (Li, 1993).

(Fig. 5)

**Figure 5:**
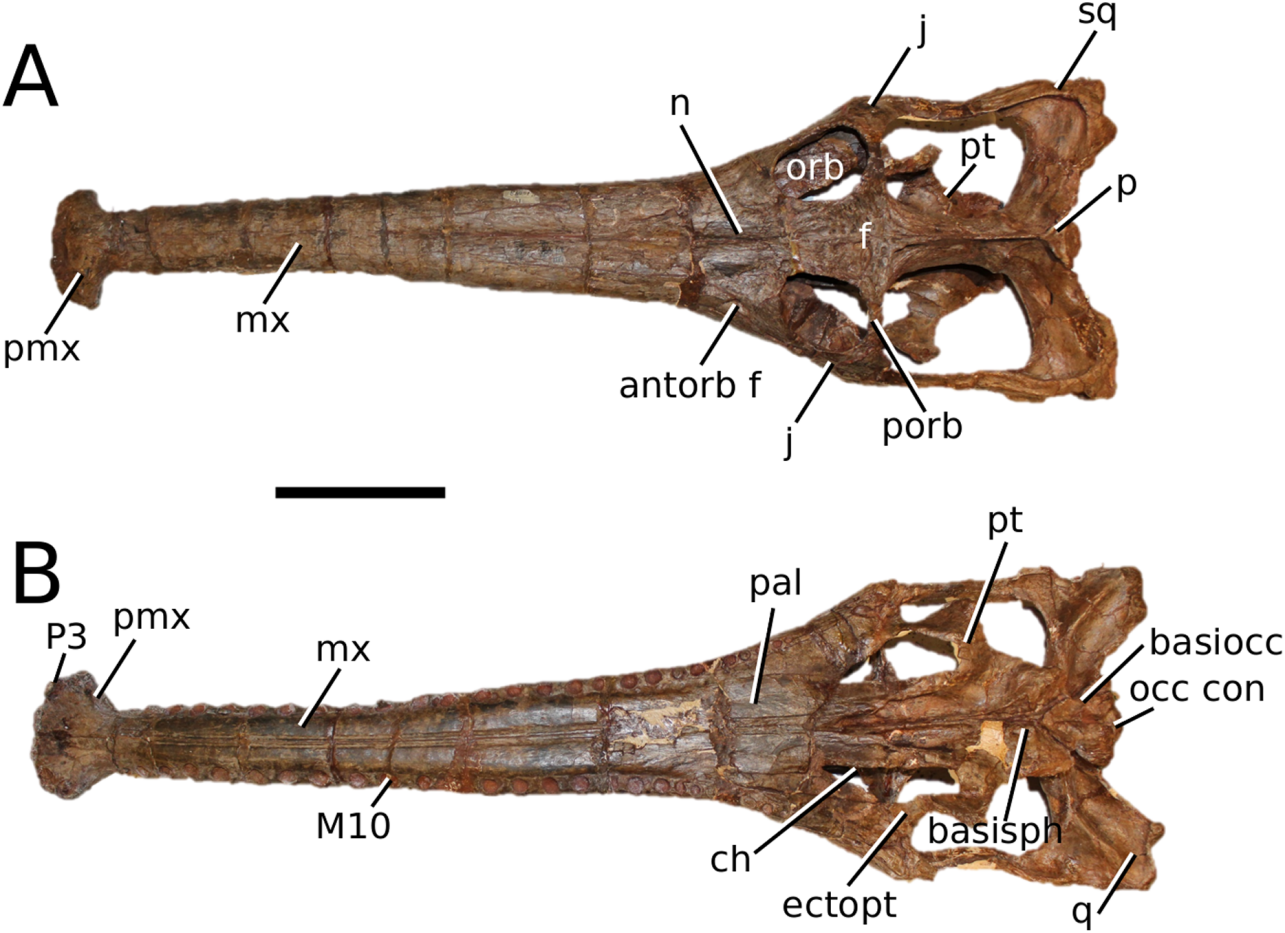
Chinese teleosauroid previously referred to as *Peipehsuchus*. The Chinese teleosauroid previously referred to as *Peipehsuchus* (see Li, 1993), IVPP V 10098, holotype. Skull in (A) dorsal and (B) ventral (palatal) views. Refer to abbreviations list. Scale bars: 10 cm.

**Specimen**—IVPP V 10098, a complete skull.

**Age**—Toarcian, Early Jurassic.

**Locality**—Daxian, Szechuan, China.

**Stratigraphic horizon**—Ziliujing Formation.

**Scoring sources**—IVPP V 10098 was examined first-hand and was also discussed in great length with E. Wilberg.

**Autapomorphic characters of IVPP V 10098**—extreme constriction of premaxillae posterior to external nares (relative to other teleosauroids), creating a laterally expanded, ‘beak-like’ premaxilla; anterior- to mid-maxilla undulates mediolaterally in dorsal view; well-developed palatal canals; the first premaxillary alveolus (P1) and second premaxillary alveolus (P2) oriented immediately laterally to one another, with the anterior-most margins of both alveoli sloping weakly anterolaterally; weak lateral expansion of the premaxilla (the P3 is situated marginally ventrally to the P2); P3 is enlarged relative to the P2 and approximately the same size as the P4.

**Emended diagnosis**—mesorostrine skull; tooth row and occipital condyle aligned, and quadrate condyle at a lower level (shared with *Charitomenosuchus*, *Proexochokefalos*, *Neosteneosaurus* and Machimosaurini); tooth row and occipital condyle aligned on the same plane with quadrate at a slightly lower level (similar to *Charitomenosuchus*, *Proexochokefalos*, *Neosteneosaurus* and Machimosaurini); shallow ornamentation of the premaxillae and maxillae (similar to *Indosinosuchus*, *Aeolodon*, *Bathysuchus* and *Sericodon*); frontal ornamentation extends from the centre to the lateral- and anterior-most areas (shared with *Plagiophthalmosuchus*, *Indosinosuchus*, *Platysuchus*, *Teleosaurus*, *Mycterosuchus*, *Macrospondylus* and *Clovesuurdameredeor*); external nares oriented anterodorsally (shared with *Plagiophthalmosuchus*, *Indosinosuchus*, *Platysuchus*, *Teleosaurus*, *Mycterosuchus*, *Aeolodon*, *Bathysuchus* and *Sericodon*); external nares ‘8-shaped’ in anterior view (shared with *Mystriosaurus*, *I. potamosiamensis*, *Bathysuchus* and *Aeolodon*); premaxilla anteroposterior length less than 25% of total rostrum length (shared with *Mystriosaurus*, *Mac. buffetauti* and *Mac. mosae*); premaxilla anterior and anterolateral margins are orientated anteroventrally and extend ventrally (shared with *Indosinosuchus*, *Platysuchus*, *Mycterosuchus*, *Aeolodon*, *Bathysuchus* and *Sericodon*); over 67% of total premaxilla length posterior to the external nares (shared with *Plagiophthalmosuchus*, *I. potamosiamensis*, *Mycterosuchus*, *Aeolodon*, *Bathysuchus* and *Sericodon*); small antorbital fenestrae present; supratemporal fenestrae sub-rectangular in shape; anterolateral margin of supratemporal fossae noticeably inclined anterolaterally (shared with *Mystriosaurus*, *Indosinosuchus*, *Teleosaurus*, *Platysuchus*, *Mycterosuchus*, *Aeolodon*, *Bathysuchus* and *Sericodon*); frontal width subequal with orbital width (shared with *I. kalasinensis*, *Macrospondylus*, *Clovesuurdameredeor*, *Charitomenosuchus*, *Proexochokefalos*, *Yvridiosuchus*, *Mac. hugii* and *Mac. rex*); squamosal project further posteriorly than occipital condyle (shared with *Plagiophthalmosuchus*, *Neosteneosaurus*, *Yvridiosuchus*, *Lemmysuchus* and *Mac. mosae*); orbit anteroposteriorly elongated and ellipsoid in shape (similar to *Plagiophthalmosuchus*, *Platysuchus*, *Aeolodon*, *Macrospondylus*, *Charitomenosuchus*, *Seldsienean*, *Deslongchampsina*, *Proexochokefalos* and *Neosteneosaurus*); postorbital reaches the orbit posteroventral margin (shared with *Mystriosaurus*, *I. potamosiamensis*, *Platysuchus*, *Teleosaurus* and *Mycterosuchus*); pterygoid flange oriented horizontally (shared with *Teleosaurus*); four premaxillary alveolar pairs; 27 maxillary alveolar pairs; P3 and P4 do not form a couple (shared with *Bathysuchus*); small P1 compared to the P2 (similar to *Macrospondylus*).

**Remarks**—this taxon, along with the holotype of *Peipehsuchus teleorhinus* (IVPP RV 48001), is currently being re-described by MM Johnson and colleagues.

*Platysuchus* Westphal, 1961

**Type species**—*Mystriosaurus multiscrobiculatus* Berckhemer, 1929. Now referred to as *Platysuchus multiscrobiculatus* (Berckhemer, 1929), Westphal, 1961.

**Etymology**—‘Wide crocodile’. *Platys* comes from the Greek *platýs* (πλατύς) meaning wide (referring to the flattened, expanded osteoderms and dermal shield), and *suchus* is the Latinized form of the Greek *soukhos* (σοῦχος), meaning crocodile.

**Diagnosis**—same as the only known species (monotypic genus).

*Platysuchus multiscrobiculatus* (Berckhemer, 1929) Westphal, 1961

(Fig. 6)

**Figure 6:**
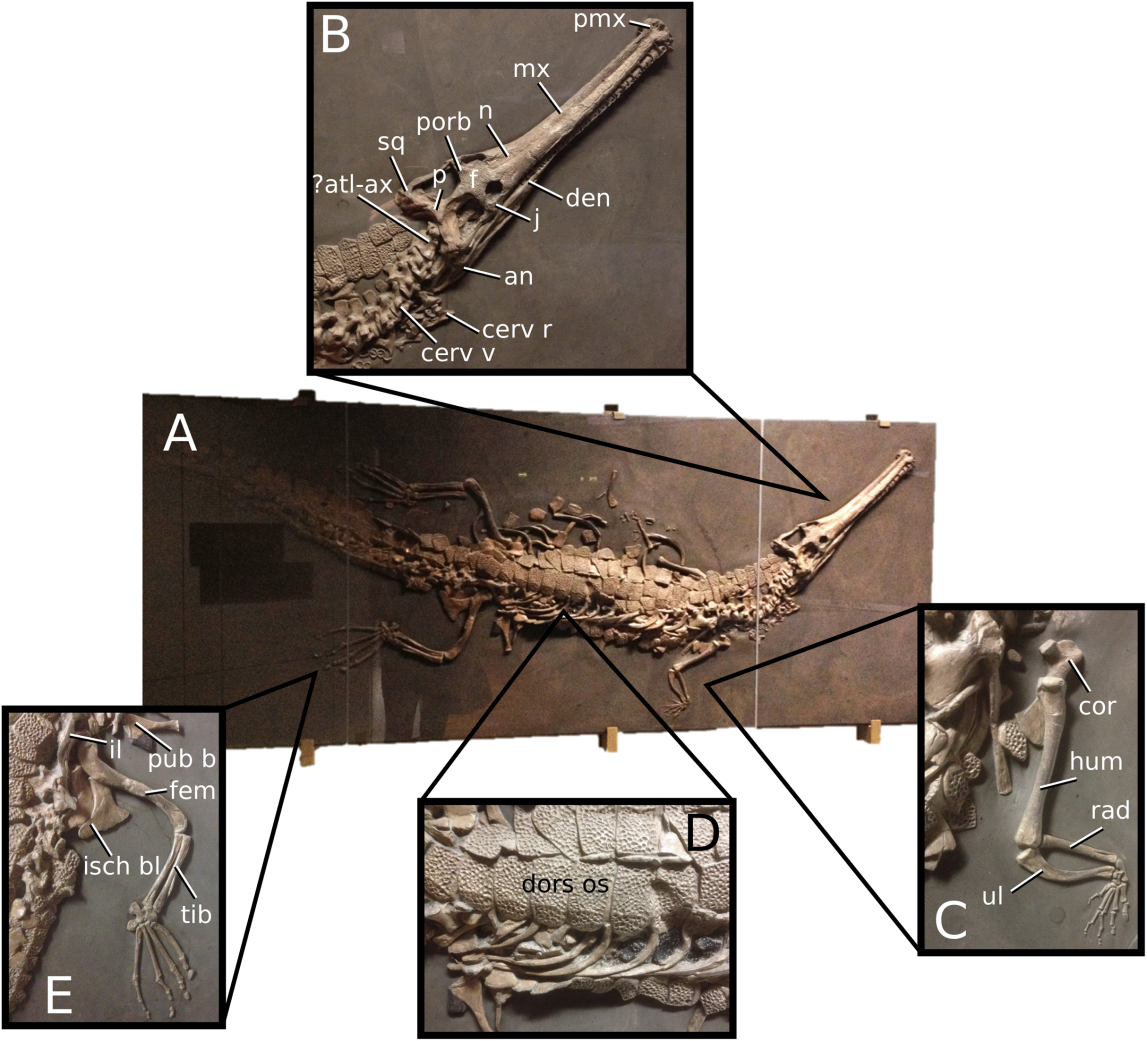
*Platysuchus multiscrobiculatus*. *Platysuchus multiscrobiculatus* (Berckhemer, 1929) Westphal, 1961, SMNS 9930, holotype. (A) Nearly complete skeleton, with close-up views of (B) the skull, (C) forelimb, (D) trunk region and (E) hindlimb. Refer to abbreviations list. Not to scale.

**Holotype**—SMNS 9930, a nearly complete skeleton.

**Referred material**—MNHNL TU895 (a partial rostrum); UH 1 (complete skeleton).

**Age**—lower Toarcian, Early Jurassic.

**Localities**—Holzmaden, Baden-Württemberg, Germany; Foetz, Luxembourg.

**Stratigraphic horizons**—Posidonia Shale Formation; *Harpoceras serpentinum* ammonite Zone (‘*schistes bitumineux*’).

**Scoring sources**—the holotype (SMNS 9930) and MNHNL TU895 were examined first-hand. Additional information was taken from Westphal (1961, 1962).

**Autapomorphic characters of *Pl. multiscrobiculatus***—prefrontal and lacrimal both ornamented with meandering, elongated grooves; mid- and posterior squamosal well ornamented with small, circular, closely packed pits; frontal contribution to the intertemporal bar frontal wider than the parietal in dorsal view; jugal excluded from the orbit by lacrimal-postorbital contact; P1 and P2 do not form a couplet and are not oriented on the anterior margin of the premaxilla; tuberculum of the dorsal rib medium-sized; ischium with thickened, robust ischial neck; shortened, stocky pubis with a relatively subcircular proximal rim.

**Emended diagnosis**—longirostrine snout; tooth row and quadrate condyle unaligned with the tooth row at a lower level, and both below the occipital condyle (shared with *Teleosaurus*); tooth row at a lower level than the quadrate (shared with *Plagiophthalmosuchus*, *Indosinosuchus*, *Teleosaurus*, *Mycterosuchus* and *Macrospondylus*); frontal ornamentation extends from the centre to lateral- and anterior-most regions (shared with *Plagiophthalmosuchus*, the Chinese teleosauroid, *Indosinosuchus*, *Teleosaurus*, *Mycterosuchus*, *Macrospondylus* and *Clovesuurdameredeor*); external nares oriented anterodorsally (shared with *Plagiophthalmosuchus*, the Chinese teleosauroid, *Indosinosuchus*, *Teleosaurus*, *Mycterosuchus*, *Aeolodon*, *Bathysuchus* and *Sericodon*); the premaxilla anterior and anterolateral margins are orientated anteroventrally and extend ventrally (shared with *Mystriosaurus*, the Chinese teleosauroid, *Indosinosuchus*, *Teleosaurus*, *Mycterosuchus*, *Aeolodon*, *Bathysuchus* and *Sericodon*); presence of small, mediolaterally thin antorbital fenestrae; anterior margin of the supratemporal fossae are noticeably inclined anterolaterally (shared with *Mystriosaurus*, the Chinese teleosauroid, *Indosinosuchus*, *Teleosaurus*, *Mycterosuchus*, *Aeolodon*, *Bathysuchus* and *Sericodon*); frontal width is broader than orbital width (shared with *Plagiophthalmosuchus*, *Mystriosaurus*, *Teleosaurus*, *Mycterosuchus*, *Bathysuchus*, *Aeolodon*, *Pr*. cf. *bouchardi*, *Neosteneosaurus*, *Mac. buffetauti* and *Mac. mosae*); frontal-postorbital suture is lower than the intertemporal bar (shared with *Teleosaurus*); orbits are longitudinal ellipsoid in shape (shared with *Plagiophthalmosuchus*, the Chinese teleosauroid, *Aeolodon*, *Macrospondylus*, *Charitomenosuchus*, *Seldsienean*, *Proexochokefalos*, *Deslongchampsina* and *Neosteneosaurus*); postorbital reaches the orbit posteroventral margin and forms an extensive area of the orbit ventral margin (shared with *Mystriosaurus*, *Indosinosuchus*, the Chinese teleosauroid, *Teleosaurus* and *Mycterosuchus*); five premaxillary alveoli (shared with *Teleosaurus*, *Bathysuchus* and *Sericodon*); interalveolar spacing between P1-P2 and P3-P4 relatively the same size (shared with *Mycterosuchus*, *Bathysuchus* and *Sericodon*); anterior maxillary teeth procumbent (shared with *Plagiophthalmosuchus*, *I. kalasinensis*, *Teleosaurus*, *Sericodon*, *Aeolodon*, *Macrospondylus* and *Charitomenosuchus*);neural spine height is greater than centrum height (similar to *Neosteneosaurus*); tuberculum of dorsal rib situated on the medial edge (shared with *Aeolodon*, *Macrospondylus* and *Lemmysuchus*); shortened and squat scapula (similar to *Macrospondylus*); proximal humerus posteriorly expanded and weakly hooked (shared with *Teleosaurus*); forelimb relatively shorter than hindlimb by approximately 22% (similar to *Macrospondylus*); tibia shorter than the femur by approximately 25% (similar to *Macrospondylus*); small round to ellipsoid pits on all osteoderms that are very densely distributed, with a ‘honeycomb’ pattern (shared with *Teleosaurus*); presacral osteoderms are strongly curved and closely locked together, forming a dorsal ‘shield’ (shared with *Teleosaurus*).

*Teleosaurus* Geoffroy Saint-Hilaire, 1825

**Type species**—*Crocodilus cadomensis* Lamouroux, 1820. Now referred to as *Teleosaurus cadomensis* (Lamouroux, 1820), Geoffroy Saint-Hilaire, 1825.

**Etymology**—‘Perfect lizard’. *Teleo* is from the Anceint Greek *téleios* (τέλειος) meaning perfect, and *saurus* is the Latinized version of *saûros* (σαυρoς), which is Ancient Greek for lizard or reptile.

*Teleosaurus cadomensis* Lamouroux, 1820

(Fig. 7)

**Figure 7:**
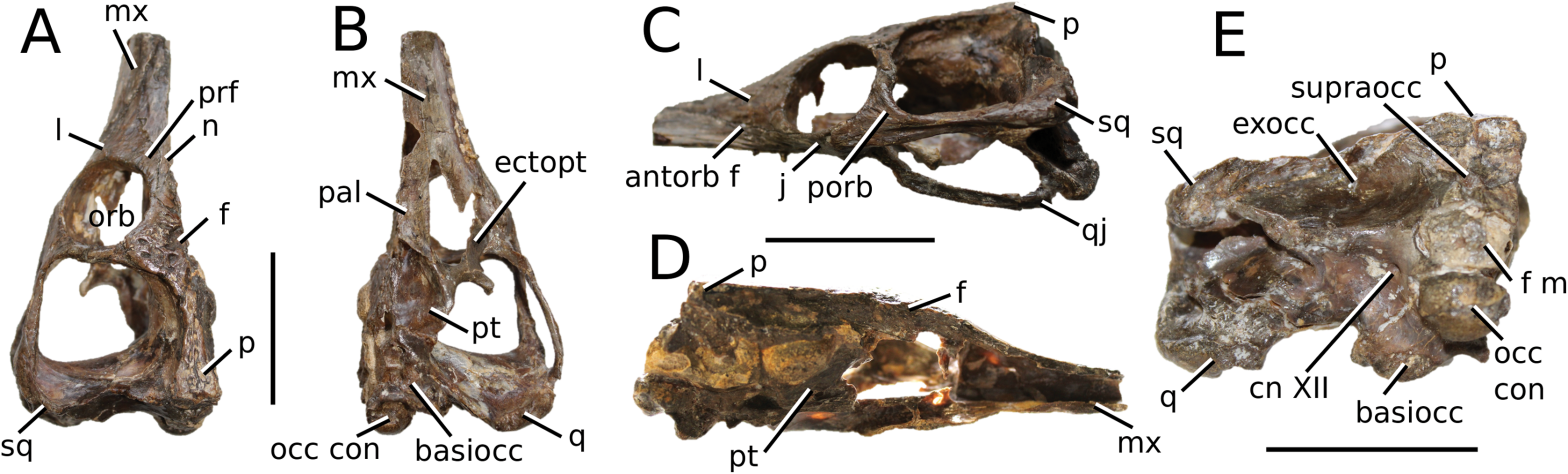
*Teleosaurus cadomensis*. *Teleosaurus cadomensis* (Lamouroux, 1820), MNHN AC 8746, neotype. Partial skull in (A) dorsal, (B) ventral (palatal), (C) left lateral, (D) right lateral and (E) occipital views. Refer to abbreviations list. Scale bars: 5 cm.

**Holotype**—MNHN.F AC 8746, a partially complete skull, with associated postcranial material. The specimen was initially found by Pierre Tesson, who traded it to Lamouroux. Lamouroux briefly noted it (1820) and then sent the specimen to Georges Cuvier. It was fully described by Cuvier (1824) and Geoffroy Saint-Hilaire (1825). See Brignon (2018a) for more details.

**Referred material**—NHMUK PV OR 119a (dorsal osteoderms); NHMUK PV R 4207 (dorsal osteoderms); NHMUK PV OR 32588 (dorsal, sacral and caudal vertebrae); NHMUK PV OR 32657 (femur); NHMUK PV OR 32680 (ischium); NHMUK PV OR 33124 (mandibular symphysis); NHMUK PV OR 39788 (partial rostrum); and additional casts (e.g. NHMUK PV R 880; NHMUK PV R 880a).

**Age**—Bathonian, Middle Jurassic.

**Locality**—Allemagne, 3 km south of Caen, Calvados, Normandy, France.

**Stratigraphic horizon**—‘*Calcaire de Caen*’.

**Scoring sources**—the neotype and all referred material mentioned above was studied first-hand. Lamouroux (1820), Geoffroy Saint-Hilaire (1825), Eudes-Deslongchamps (1867), Vignaud (1995) and Jouve (2009) provided additional information.

**Autapomorphic characters of *T. cadomensis***—small, subcircular, shallow antorbital fenestrae; supratemporal fenestrae box- or square-shaped; postorbital and squamosal are relatively the same length, with the squamosal being slightly longer (~10%); choanae mediolaterally wider than palatines.

**Emended diagnosis**—longirostrine, gracile snout; tooth row and quadrate condyle unaligned with the tooth row at a lower level, and both below the occipital condyle (shared with *Platysuchus*); tooth row at a lower level than the quadrate (shared with *Plagiophthalmosuchus*, *Indosinosuchus*, *Platysuchus*, *Mycterosuchus* and *Macrospondylus*); rostrum narrows immediately anterior to the orbits (shared with *I. potamosiamensis*, *Mycterosuchus*, *Aeolodon*, *Bathysuchus*, *Sericodon* and *Seldsienean*); frontal ornamentation extends from the centre to lateral- and anterior-most regions (shared with *Plagiophthalmosuchus*, the Chinese teleosauroid, *Indosinosuchus*, *Platysuchus*, *Mycterosuchus*, *Macrospondylus* and *Clovesuurdameredeor*); external nares oriented anterodorsally (shared with *Plagiophthalmosuchus*, the Chinese teleosauroid, *Indosinosuchus*, *Platysuchus*, *Mycterosuchus*, *Aeolodon*, *Bathysuchus* and *Sericodon*); premaxilla anterior and anterolateral margins of are orientated anteroventrally and extend ventrally (shared with *Mystriosaurus*, the Chinese teleosauroid, *Indosinosuchus*, *Platysuchus*, *Mycterosuchus*, *Aeolodon*, *Bathysuchus* and *Sericodon*); anterior margin of the supratemporal fossae are noticeably inclined anterolaterally (shared with *Mystriosaurus*, the Chinese teleosauroid, *Indosinosuchus*, *Platysuchus*, *Mycterosuchus*, *Bathysuchus* and *Aeolodon*); anteromedial projection of the frontal is relatively broad but becomes instantly mediolaterally thin at the anterior-most part (shared with *Sericodon*); frontal width is broader than orbital width (shared with *Plagiophthalmosuchus*, *Mystriosaurus*, *Platysuchus*, *Mycterosuchus*, *Bathysuchus*, *Aeolodon*, *Pr*. cf. *bouchardi*, *Neosteneosaurus*, *Mac. buffetauti* and *Mac. mosae*); frontal-postorbital suture is lower than the intertemporal bar (shared with *Platysuchus*); dorsal margins of orbits upturned (shared with *I. potamosiamensis*, *Mycterosuchus* and *Aeolodon*); postorbital reaches the orbit posteroventral margin and forms an extensive area of the orbit ventral margin (shared with *Mystriosaurus*, *Indosinosuchus*, the Chinese teleosauroid, *Platysuchus* and *Mycterosuchus*); pterygoid flange oriented horizontally (shared with the Chinese teleosauroid); five premaxillary alveolar pairs (shared with *Platysuchus*, *Bathysuchus* and *Sericodon*); anterior maxillary teeth procumbent (shared with *Indosinosuchus*, *Platysuchus*, *Aeolodon*, *Sericodon*, *Macrospondylus* and *Charitomenosuchus*); proximal humerus posteriorly expanded and weakly hooked (shared with *Platysuchus*); small round to ellipsoid pits that are very densely distributed, with a ‘honeycomb’ pattern (shared with *Platysuchus*); presacral osteoderms are strongly curved and closely locked together, forming a dorsal ‘shield’ (shared with *Platysuchus*).

**Remarks**—the genus *Teleosaurus*, initially defined by Geoffroy Saint-Hilaire (1825), has encompassed numerous species throughout its long history, such as *T. gladius*, *T. subulidens*, *T. geoffroyi*, *T. minimus* and *T. eucephalus* (Quenstedt, 1852; Phillips, 1871; Seeley, 1880; Eudes-Deslongchamps, 1868c). However, the majority of these historic *Teleosaurus* species are currently considered invalid due to the following propositions: (1) thought to be juveniles or sub-adults, and therefore subjective junior synonyms of *T. cadomensis* (e.g. Jouve, 2009); (2) uncertainty of teleosauroid ontogenetic stages and sexual dimorphism (see Johnson, Young & Brusatte, 2020); and (3) loss of original material. Therefore, we currently only recognize *T. cadomensis* as a valid species; the issue regarding the validity of other ‘*Teleosaurus*’ species is beyond the scope of this manuscript.

*Mycterosuchus* Andrews, 1913

**Type species**—*Steneosaurus nasutus* Andrews, 1909. Now referred to as *Mycterosuchus nasutus* (Andrews, 1909), Andrews, 1913.

**Etymology**—‘(Long) Nose crocodile’. *Myctero* comes from the Latin *mycto* meaning nose, referring to the elongated rostrum of this taxon; *suchus* is the Latinized form of the Greek *soukhos* (σοῦχος), meaning crocodile.

**Diagnosis**—same as the only known species (monotypic genus).

*Mycterosuchus nasutus* (Andrews, 1909) Andrews, 1913

(Fig. 8)

**Figure 8:**
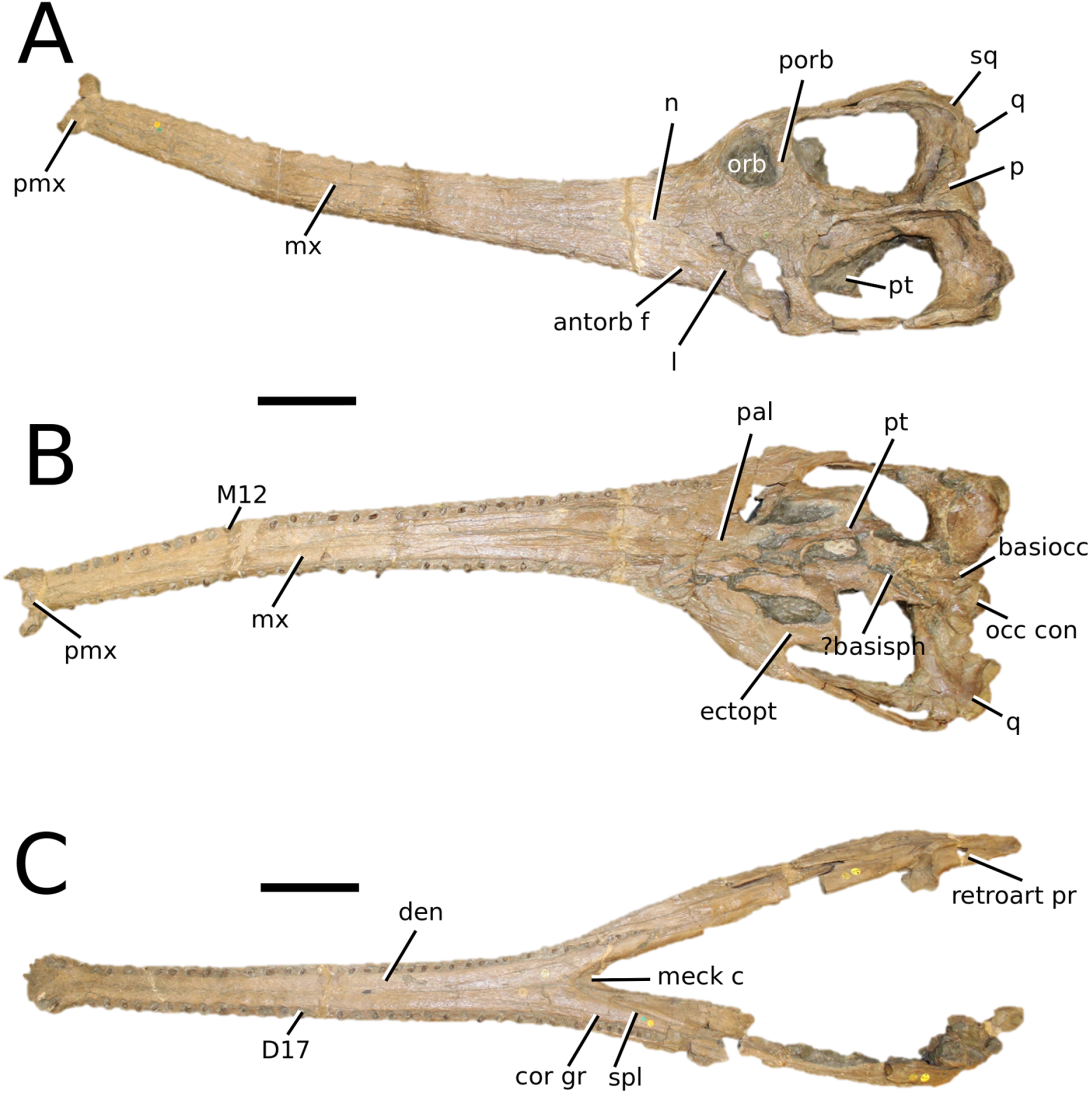
*Mycterosuchus nasutus*. *Mycterosuchus nasutus* Andrews, 1913, NHMUK PV R 2617, holotype. Skull in (A) dorsal and (B) ventral (palatal) views, and dentary in (C) dorsal view. Note the extremely rugose dorsal cranium. Refer to abbreviations list. Scale bars: 10 cm.

**Holotype**—NHMUK PV R 2167, a complete skull and mandible, with additional material (including vertebrae (cervical, dorsal, sacral and caudal), cervical and dorsal ribs, scapulocoracoid, two partial femora, one radius, one ulna, multiple phalanges and tarsals, isolated teeth and multiple dorsal osteoderms).

**Referred material**—CAMSM J.1420 (nearly complete skeleton); NHMUK PV R 3892 (dorsal and sacral vertebrae); NHMUK PV R 4059 (partial skull); unnumbered GZG specimen (complete skull). Possible NM partial skeleton (catalogue number unknown, photographs provided by B. Ekrt).

**Age**—Middle Callovian, Middle Jurassic.

**Locality**—Peterborough, UK.

**Stratigraphic horizon**—Peterborough Member, Oxford Clay Formation, Ancholme Group.

**Scoring sources**—the holotype (NHMUK PV R 2167) and all referred material (excluding the NM skeleton) mentioned above were studied first-hand.

**Autapomorphic characters of *Myc. nasutus***—overall cranium and mandible extremely rugose; elongate, slender rostrum (approximately 73% of total skull length); maxilla ornamented with an array of irregular patterns of deep rugosities and anastomosing grooves; reduced quadrate condyles; palatine anterior margin terminates level to 29th maxillary alveoli, or more distal alveoli; curvature of the angular is gradual in the anterior region, but more abrupt in the posterior-most region; on the retroarticular process, the length of the attachment surface for the adductor muscles is more than twice its width; D1 strongly anteriorly oriented; the neural arches of the posterior cervical vertebrae are taller than the vertebral centra; the posterior edge of the scapula is more strongly concave than the anterior edge; the humeral head is weakly posteriorly expanded and hooked with a club-like shape; the ulna is more than 25% longer than the radius; the pubic shaft is over 50% length of the pubic plate; anteromedial tuber of the femur is the largest of the proximal tubera; size of calcaneal tuber approximately 25% of total astragalus size; large, heavyset dorsal osteoderms with large, round-to-ellipsoid (D-shaped) irregular pits that are well separated from one another.

**Emended diagnosis**—longirostrine snout; tooth row and quadrate condyle unaligned and quadrate at a lower level, but both below the occipital condyle (shared with *Indosinosuchus* taxa); well-developed and extensive ornamentation on the premaxillae, maxillae, frontal, prefrontal, lacrimal and postorbital; frontal ornamentation composed of small sub-circular to elongate pits that are closely spaced or, that can fuse and become a ridge-groove pattern (similar to *Mystriosaurus*); rostrum narrows immediately anterior to the orbits (shared with *I. potamosiamensis*, *Teleosaurus, Aeolodon*, *Bathysuchus*, *Sericodon* and *Seldsienean*); premaxilla anterior and anterolateral margins are strongly anteroventrally deflected and extend ventrally (shared with *Mystriosaurus*, the Chinese teleosauroid, *Indosinosuchus*, *Platysuchus*, *Teleosaurus*, *Aeolodon*, *Bathysuchus* and *Sericodon*); more than 67% of total premaxilla length is posterior to the external nares (shared with *Plagiophthalmosuchus*, *I. potamosiamensis*, the Chinese teleosauroid, *Aeolodon*, *Bathysuchus* and *Sericodon*); external nares are ‘8’ shaped in dorsal view due to enlarged anterior and posterior projections of the premaxilla (shared with *Bathysuchus*); external nares are anterodorsally oriented (shared with *Mystriosaurus*, the Chinese teleosauroid, *Platysuchus* and *Bathysuchus*); clustering of large, circular foramina along lateral margin of external nares (similar to *Mystriosaurus*, *I. kalasinensis* and Machimosaurini); small, subcircular antorbital fenestrae; the anterior margin of the supratemporal fossae are noticeably inclined anterolaterally (shared with *Mystriosaurus*, the Chinese teleosauroid, *Indosinosuchus*, *Platysuchus*, *Teleosaurus*, *Aeolodon*, *Bathysuchus* and *Sericodon*); frontal width broader than orbital width (shared with *Plagiophthalmosuchus*, *Mystriosaurus*, *Platysuchus*, *Teleosaurus*, *Bathysuchus*, *Aeolodon*, *Neosteneosaurus*, *Mac. buffetauti* and *Mac. mosae*); circular orbits (shared with *Mystriosaurus*, *Teleosaurus*, *Indosinosuchus*, *Clovesuurdameredeor* and Machimosaurini); dorsal margins of orbits are upturned (shared with *I. potamosiamensis*, *Teleosaurus* and *Aeolodon*); postorbital reaches the orbit posteroventral margin and extensively forms part of the orbit ventral margin (shared with *Mystriosaurus*, the Chinese teleosauroid, *I. potamosiamensis*, *Platysuchus* and *Teleosaurus*); reduced basioccipital tubera (similar to *Plagiophthalmosuchus*, *Bathysuchus* and *Sericodon*); mandibular symphysis over 50% of mandible length (shared with *Bathysuchus*, *Aeolodon*, *Macrospondylus*, *Seldsienean* and *Charitomenosuchus*); mandibular symphysis depth is very narrow, approximately 4–4.5% of the mandible length (shared with *Charitomenosuchus*); the P1 and P2 do not form a couplet, and the interalveolar spacing between the P1-P2 and P3-P4 are relatively the same size (shared with *Platysuchus*, *Bathysuchus* and *Sericodon*); both the P1 and P2 alveoli are oriented laterally (shared with *Bathysuchus* and *Sericodon*); the P1 and P2 do not form a couplet but are still oriented on the anterior margin of the premaxilla (shared with *Bathysuchus* and *Sericodon*); P1 and P2 are on the same transvers plane (shared with *Aeolodon*, *Bathysuchus* and *Sericodon*); teeth slender, pointed and weekly mediolaterally compressed (shared with *Bathysuchus* and *Aeolodon*); the tubercula and articular facets in the dorsal ribs are positioned directly in the middle (shared with *Charitomenosuchus*); the tubercula in the dorsal ribs are large and pronounced (shared with *Neosteneosaurus* and Machimosaurini); tibia approximately 40–50% shorter than the femur (shared with *Charitomenosuchus*, *Neosteneosaurus* and Machimosaurini); the medial femoral condyle is noticeably larger than the lateral femoral condyle (shared with *Charitomenosuchus* and *Neosteneosaurus*).

**Remarks**—the skull and mandible of the NHMUK holotype was originally numbered PV R 2617, along with the associated postcranial material. The skull and mandible were then reregistered PV R 3577 in error (what year and by whom is unknown). *Mycterosuchus* has also been considered as a synonym of *Steneosaurus leedsi* (= *Charitomenosuchus leedsi*) in certain studies (Vignaud, 1995).

*Aeolodon* Von Meyer, 1832

**Type species**—*Crocodilus priscus* Von Sömmerring, 1814. Now referred to as *Aeolodon priscus* (Von Sömmerring, 1814), Von Meyer, 1832.

**Etymology**—‘Changeful tooth’. *Aeolo* comes from the Ancient Greek *aiólos* (αἰόλος) meaning changeful, and *don* from the Greek *dónti* (δόντι) meaning tooth. Von Meyer (1832) wrote that he used this name based on the holotype’s “heterodont teeth”.

**Diagnosis**—same as the only known species (monotypic genus).

*Aeolodon priscus* (Von Sömmerring, 1814) Von Meyer, 1832

(Fig. 9)

**Figure 9:**
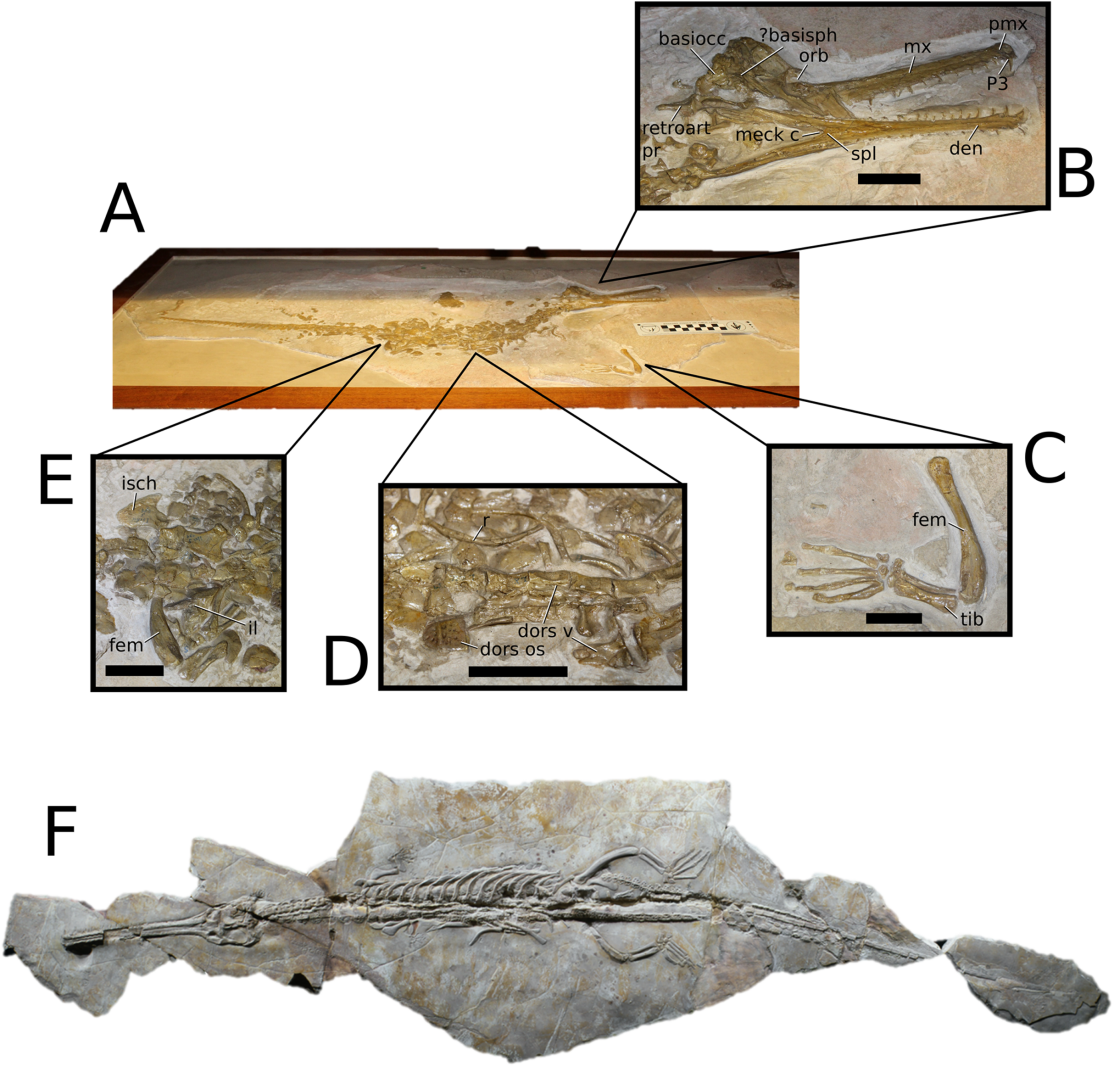
*Aeolodon priscus*. *Aeolodon priscus* (Von Sömmerring, 1814), (A–E) NHMUK PV R 1086, holotype and (F) MNHN.F.CNJ 78, referred specimen (modified from Fig. 10 in Foffa et al. (2019)). (A) Partial skeleton with close-ups of (B) the skull, (C) hindlimb, (D) trunk region and (E) pelvic area. (F) Nearly complete skeleton. Scale bars: 10 cm (A) and 3 cm (B–E), (F) not to scale.

**Figure 10:**
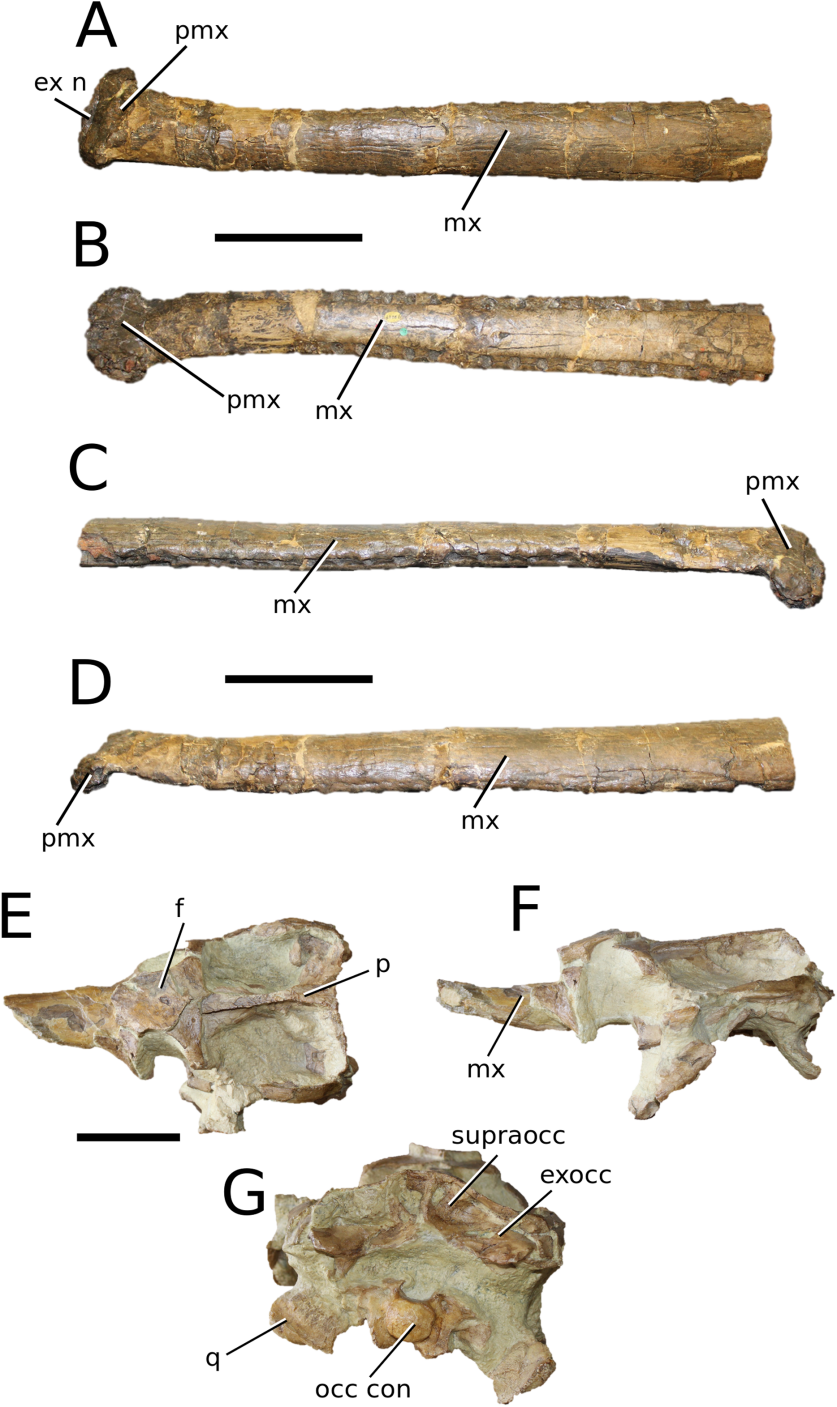
*Bathysuchus megarhinus*. *Bathysuchus megarhinus* (Hulke, 1871) Foffa et al., 2019. (A–D) NHMUK PV OR 43086, holotype; (E–G) unnumbered LPP specimen. In (A and E) dorsal, (B) ventral, (C) right lateral, (D and F) left lateral and (G) occipital views. Refer to abbreviations list. Scale bars: 10 cm.

**Holotype**—NMHUK PV R 1086, a nearly complete skeleton.

**Referred material**—MNHN.F.CNJ 78 (nearly complete skeleton).

**Age**—lower Tithonian, Late Jurassic.

**Localities**—Daiting, southern Germany; Canjuers, Var, France.

**Stratigraphic horizons**—Mörnsheim Formation; Canjuers conservation Lagerstätte.

**Scoring sources**—the holotype (NMHUK PV R 1086) and referred specimen (MNHN.F.CNJ 78a) were both studied first-hand.

**Autapomorphic characters of *A. priscus***—shallow elliptical pits on the frontal; length of the attachment surface for the *m. pterygoideus posterior* on the retroarticular process is short, and subequal to its width; neural spine and centrum heights of the mid-cervical vertebrae are approximately equal; distal coracoid with rounded edges and a deep coracoid foramen; extremely shortened ulna and radius relative to humerus; ulna with little curvature, only in the proximal-most region; metacarpals IV and V are similar in robusticity to II-III ; ischial plate sub-triangular; tibia 30–40% shorter than the femur; dorsal osteoderm ornamentation consists of large, well-spaced circular pits.

**Emended diagnosis**—longirostrine skull; rostrum narrows immediately anterior to the orbits (shared with *I. potamosiamensis*, *Teleosaurus*, *Mycterosuchus*, *Bathysuchus*, *Sericodon* and Seldsienean); shallow, inconspicuous ornamentation of the premaxillae and maxillae (similar to the Chinese teleosauroid, *Indosinosuchus*, *Bathysuchus* and *Sericodon*); no ornamentation on the prefrontal (shared with *Plagiophthalmosuchus*, *I. potamosiamensis*, *Bathysuchus* and *Sericodon*) and lacrimal (shared with *Plagiophthalmosuchus*, *I. potamosiamensis*, *Sericodon*, *Macrospondylus* and *Charitomenosuchus*); frontal ornamentation restricted to centre (shared with *Sericodon*, *Charitomenosuchus*, *Seldsienean*, *Deslongchampsina*, *Proexochokefalos*, *Neosteneosaurus* and Machimosaurini); external nares oriented anterodorsally (shared with the Chinese teleosauroid, *Indosinosuchus*, *Platysuchus*, *Teleosaurus*, *Mycterosuchus*, *Bathysuchus* and *Sericodon*); external nares noticeably ‘8’-shaped in anterior view (shared with *Mystriosaurus*, the Chinese teleosauroid, *I. potamosiamensis* and *Bathysuchus*); the premaxilla anterior and anterolateral margins are orientated anteroventrally and extend ventrally (shared with *Mystriosaurus*, the Chinese teleosauroid, *Indosinosuchus*, *Platysuchus*, *Teleosaurus*, *Mycterosuchus*, *Bathysuchus* and *Sericodon*); sub-rectangular supratemporal fenestrae; the anterior margin of the supratemporal fossae are noticeably inclined anterolaterally (shared with *Mystriosaurus*, the Chinese teleosauroid, *Indosinosuchus*, *Platysuchus*, *Teleosaurus*, *Mycterosuchus*, *Bathysuchus* and *Sericodon*); frontal width is broader than orbital width (shared with *Plagiophthalmosuchus*, *Mystriosaurus*, *Platysuchus*, *Teleosaurus*, *Mycterosuchus*, *Bathysuchus*, *Pr*. cf. *bouchardi*, *Neosteneosaurus*, *Mac. buffetauti* and *Mac. mosae*); orbits are longitudinal ellipsoid in shape (shared with *Plagiophthalmosuchus*, the Chinese teleosauroid, *Platysuchus*, *Macrospondylus*, *Charitomenosuchus*, *Seldsienean*, *Proexochokefalos*, *Deslongchampsina* and *Neosteneosaurus*); the dorsal margins of the orbits are upturned (shared with *I. potamosiamensis*, *Teleosaurus* and *Mycterosuchus*); angular poorly curved (somewhat similar to *Plagiophthalmosuchus* and *Mystriosaurus*); mandibular symphysis is over 50% of the mandible length (shared with *Mycterosuchus*, *Bathysuchus*, *Macrospondylus*, *Charitomenosuchus* and *Seldsienean*); retroarticular width subequal to the glenoid fossa (shared with *Lemmysuchus* and *Mac. buffetauti*); P1 and P2 are both on the same transverse plane (shared with *Mycterosuchus*, *Bathysuchus* and *Sericodon*); the premaxilla lateral margins are sub-rectangular, with the P3 alveoli being clearly lateral to the P2 alveoli (shared with *Mycterosuchus*, *Bathysuchus* and *Sericodon*); at least 22 dentary alveolar pairs; premaxillary and anterior maxillary apicobasal length to basal width ratio of the tooth crown is 3 or greater (shared with *Macrospondylus* and *Charitomenosuchus*); shallow tuberculum on the dorsal ribs (shared with *Macrospondylus* and *Charitomenosuchus*); the proximal region of the humerus is very strongly posteriorly deflected and hooked (shared with *Charitomenosuchus* and *Neosteneosaurus*); femoral condyles are relatively the same size (shared with *Macrospondylus*, *Platysuchus* and *Lemmysuchus*); pits on dorsal osteoderms arranged in alternating rows (similar to *Bathysuchus*); dorsal osteoderms reduced in size and thickness (shared with *Bathysuchus*).

**Remarks**—*Crocodilus priscus* (NHMUK PV R 1086) was the first teleosauroid genus to be scientifically named by von Sömmering in 1814. Von Meyer (1830) initially presented *Aeolodon* **gen. nov.**, and prematurely used this genus for comparison with *Rhacheosaurus* (1831: 176) but did not provide a formal description until his 1832 volume. Comparing the specimen (NHMUK PV R 1086) to the modern gharial, Von Meyer (1832) noted the “heterodont” teeth (which was his basis for the new genus name) and the “limb bones and phalanges […] appear like in whales”. It is also interesting to note that Geoffroy Saint-Hilaire (1831: 48) did not believe that *Aeolodon* (“*le gavial de Sömmering*”: “Sömmering’s gavial”) could be referred to as either *Teleosaurus* or ‘*Steneosaurus*’ (mainly due to the fact that it was not found in the deposits near Caen, which Geoffroy Saint-Hilaire believed these two genera were restricted to).

Despite coming from different localities, the holotype (NHMUK PV R 1086) and referred specimen (MNHN.F.CNJ 78) share the following combination of features:

1. A longirostrine, weakly ornamented skull;
2. Protruding orbits;
3. Neural spine and centrum of the mid-cervical vertebrae are approximately equal in height;
4. Distal coracoid with rounded edges and deep coracoid foramen;
5. An elongated ilial process, more so than other teleosauroids (e.g. *Charitomenosuchus* NHMUK PV R 3806);
6. A sub-triangular ischial blade; and
7. Reduced dorsal ornamentation on osteoderms, with large, shallow, well-spaced pits.

*Bathysuchus* Foffa et al., 2019

**Type species**—*Teleosaurus megahinus* Hulke, 1871. Now referred to as *Bathysuchus megarhinus* (Hulke, 1871), Foffa et al., 2019.

**Etymology**—‘Deep water crocodile’. *Bathys*, or *vathys* (βαθυς) is Ancient Greek for deep, and *suchus* is the Latinized form of the Greek *soukhos* (σοῦχος), meaning crocodile.

**Diagnosis**—same as the only known species (monotypic genus).

*Bathysuchus megarhinus* (Hulke, 1871) Foffa et al., 2019

(Fig. 10)

**Holotype**—NHMUK PV OR 43086, a partial rostrum.

**Referred material**—DORCM G.05067i-v (premaxillae, isolated tooth and partial osteoderm), LPP unnumbered specimen (a partial rostrum, mandible and skull).

**Age**—*Aulacostephanus autissiodorensis* Sub-Boreal ammonite Zone and *Au. eudoxus* ammonite Zone, late Kimmeridgian, Late Jurassic.

**Locality**—Kimmeridge, Dorset, UK; Francoulés, Quercy, France.

**Stratigraphic horizon**—Dorset succession, lower Kimmeridge Clay Formation, Ancholme Group; between the *Quercynum* Horizon and the *Contejeani* Horizon (Hantzpergue & Lafaurie, 1983).

**Scoring sources**—the holotype (NHMUK PV OR 43086) and the unnumbered LPP specimen were studied first-hand. D. Foffa provided high quality photographs of DORCM G.05067i-v, and *B. megarhinus* was also discussed at great length with D. Foffa.

**Autapomorphic characters of *B. megarhinus***—shallow, minor ornamentation on the parietal (nearly imperceptible); extremely pronounced lateral expansion of the premaxilla with rounded, straightened lateral margins; the fifth dentary alveolar pair is posterolaterally oriented and on the posterior end of the mandibular spatula

**Emended diagnosis**—longirostrine snout; rostrum narrows immediately anterior to the orbits (shared with *I. potamosiamensis*, *Teleosaurus*, *Mycterosuchus*, *Sericodon*, *Aeolodon* and *Seldsienean*); shallow, inconspicuous ornamentation of the premaxillae and maxillae (similar to the Chinese teleosauroid, *Indosinosuchus*, *Sericodon* and *Aeolodon*); no ornamentation on the prefrontal (shared with *Plagiophthalmosuchus*, *I. potamosiamensis*, *Sericodon* and *Aeolodon*); external nares are ‘8’ shaped in dorsal view (shared with *Mystriosaurus*, the Chinese teleosauroid, *I. potamosiamensis*, *Mycterosuchus* and *Aeolodon*) and in anterior view (shared with *Mystriosaurus*, the Chinese teleosauroid, *I. potamosiamensis* and *Aeolodon*); external nares are anterodorsally oriented (shared with *Plagiophthalmosuchus*, the Chinese teleosauroid, *Indosinosuchus*, *Platysuchus*, *Mycterosuchus*, *Aeolodon*, *Bathysuchus* and *Sericodon*); reduced anteroposterior length of the external nares; more than 67% of total premaxilla length is posterior to the external nares (shared with *Plagiophthalmosuchus*, the Chinese teleosauroid, *I. potamosiamensis*, *Mycterosuchus*, *Sericodon* and *Aeolodon*); premaxillary anterior and posterior medial margin of external nares formed by two bulbous projections (shared with *Mycterosuchus*); the anterior and anterolateral margins of the premaxillae are strongly anteroventrally deflected and extend ventrally (shared with *Mystriosaurus*, the Chinese teleosauroid, *Mycterosuchus* and *Platysuchus*); inconspicuously ornamented maxillary dorsal surface (shared with the Chinese teleosauroid and *Aeolodon*), consisting of a shallow irregular pattern of ridges and anastomosing grooves; nasal, prefrontal, lacrimal are also inconspicuously ornamented; absence/extremely reduced frontal ornamentation (shared with *Aeolodon*); the rostrum narrows markedly immediately anterior to the orbits (shared with *I. potamosiamensis*, *Teleosaurus* and *Mycterosuchus*); frontal width is broader than the orbital width (shared with *Plagiophthalmosuchus*, *Mystriosaurus*, *Platysuchus*, *Teleosaurus*, *Mycterosuchus*, *Aeolodon*, *Pr*. cf. *bouchardi*, *Neosteneosaurus*, *Mac. buffetauti* and *Mac. mosae*); palatine anterior margin terminates distal to the 20th maxillary alveoli (shared with *Mycterosuchus*); basioccipital tubera reduced (shared with *Plagiophthalmosuchus*, *Mycterosuchus* and *Sericodon*); mandibular symphysis over 50% of mandible length (shared with *Mycterosuchus*, *Aeolodon*, *Macrospondylus*, *Seldsienean* and *Charitomenosuchus*); premaxillae with five alveoli (shared with *Platysuchus*, *Teleosaurus* and *Sericodon*); the P1–P2 do not form a couplet (shared with *Platysuchus*, *Mycterosuchus* and *Sericodon*); the P3–P4 do not form a couple (shared with the Chinese teleosauroid); the P1 and P2 alveoli are lateral to each other at the anterior margin of the premaxilla (shared with *Mycterosuchus*, *Sericodon* and possibly *Aeolodon*); the P3 and P4 are aligned on the lateral plane of the external margin more so than P2 (shared with *Sericodon*); the P1 and P2 are on the same transverse plane, and the lateral margin between the P2 and P3 is sub-rectangular (shared with *Mycterosuchus*, *Sericodon* and *Aeolodon*); anterior maxillary interalveolar spacing is sub-equal to longer than adjacent alveoli; lack of apical tooth carinae (shared with *Sericodon*); the pits on the dorsal osteoderms are circular and regularly organised in alternate rows (similar with *Aeolodon*); dorsal osteoderms reduced in size and thickness (shared with *Aeolodon*).

**Remarks**—*Steneosaurus megarhinus* was initially named and described by Hulke (1871) and was recently re-described within a new monotypic genus, *Bathysuchus*, by Foffa et al. (2019). Due to similar anatomical features of the cranium, stratigraphic horizons, and comparative measurements of the humerus and femur with *Aeolodon*, Foffa et al. (2019) concluded that these two genera were evidence of the first deep water, more pelagic teleosauroids.

*Sericodon* Von Meyer, 1845

**Type species**—*Sericodon jugleri* Von Meyer, 1845.

**Etymology**—‘Silk toothed’, *Serico* comes from the Latin *sēricus* (Ancient Greek: *Sêres* [Σῆρες], possibly from Ancient Chinese 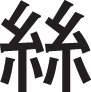) meaning silk, and *don* from the Greek *dónti* (δόντι) meaning tooth. Refers to the slender, poorly ornamented dentition of this taxon.

**Diagnosis**—same as the only known species (monotypic genus).

*Sericodon jugleri* Von Meyer, 1845

(Fig. 11)

**Figure 11:**
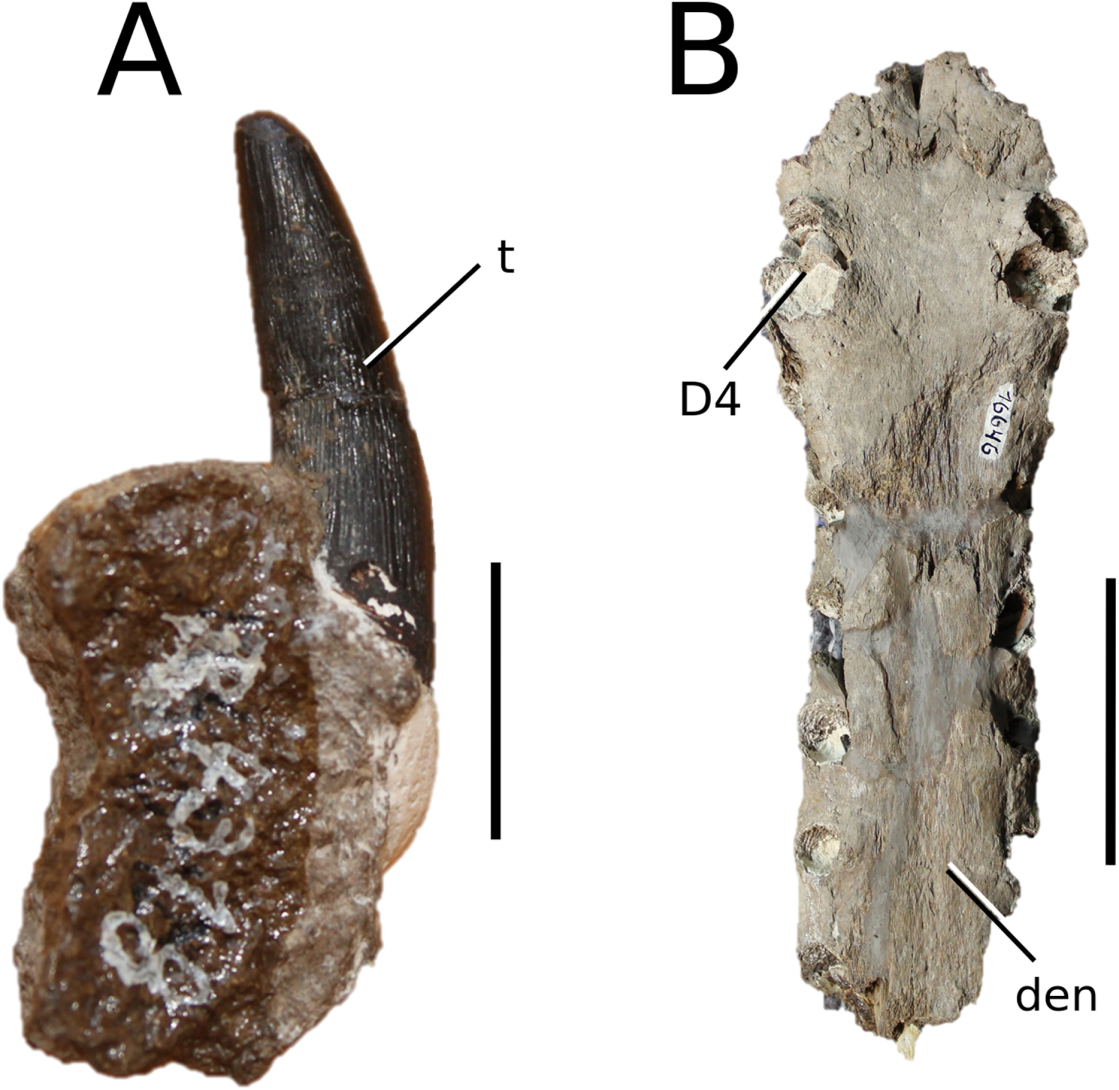
*Sericodon jugleri*. *Sericodon jugleri* Von Meyer, 1845, referred specimens. (A) Tooth in lingual view (SMF R 4318) and (B) anterior rostrum in dorsal view (LMH 16646). Refer to abbreviations list. Scale bars: 1 cm (A) and 5 cm (B).

**Type series**—Isolated teeth from Hannover (Germany) and Solothurn (Switzerland). Catalogue numbers currently unknown.

**Taxonomic note**—Von Meyer (1845) initially diagnosed a series of teeth from the Kimmeridgian of Solothurn and Hannover as the type series of *Sericodon*; however, it is unknown if this material is still available, and Von Meyer did not designate a holotype. A lectotype can be proposed for one of the NMS (Switzerland) specimens, but this needs further clarification. The authors and colleagues plan a thorough description of this specimen, as well as additional *Sericodon* material, to allow for a formal designation of a lectotype.

**Referred material**—BSY006-348, BSY007-134, BSY008-622, SCR010-312, SCR010-1184, SCR011-2460, SCR011-406, TCH005-151 TCH007-215, VTT006-171 (see Schaefer, Püntener & Billon-Bruyat, 2018), as well as LM 16645-46 (anterior mandible), NHMUK PV R 1752, NZM-PZ R2337, SMF R 431a-b, SMF R 4318 (isolated teeth), unnumbered Göttingen specimen (partial skull).

**Age**—late Kimmeridgian to early Tithonian, Late Jurassic.

**Localities**—Courtedoux-Bois de Sylleux, Courtedoux-sur Combe Ronde, Courtedoux-Tchâfouè and Courtedoux-Vâ Tche Tchâ, northwestern Switzerland; Hannover, Germany.

**Stratigraphic horizon**—Reuchenette Formation.

**Scoring sources**—Majority of material was scored using Schaefer, Püntener & Billon-Bruyat (2018). Additional specimens (LM 16645-46, NHMUK PV R 1752, NRM-PZ R2337, SMF R 431a-b, SMF R 4318, unnumbered Göttingen specimen) were examined first-hand.

**Autapomorphic characters of *Ser. jugleri***—unornamented intertemporal bar; external nares weakly subcircular in dorsal view; palatal canals extremely shallow; lack of apical enamel ridges; tuberculum and articular facet of dorsal rib situated close to the lateromedial edge; posteromedial tuber of femur reduced.

**Emended diagnosis**—longirostrine snout; rostrum narrows immediately anterior to orbits (shared with *I. potamosiamensis*, *Teleosaurus*, *Bathysuchus*, *Mycterosuchus* and *Aeolodon*); no conspicuous ornamentation on both the prefrontal (shared with *Plagiophthalmosuchus*, *I. potamosiamensis*, *Bathysuchus* and *Aeolodon*) and lacrimal (shared with *Plagiophthalmosuchus*, *I. potamosiamensis*, *Aeolodon* and *Macrospondylus*); frontal ornamentation restricted to centre (shared with *Aeolodon*, *Charitomenosuchus*, *Seldsienean*, *Deslongchampsina*, *Proexochokefalos*, *Neosteneosaurus* and Machimosaurini); external nares oriented anterodorsally (shared with *Plagiophthalmosuchus*, the Chinese teleosauroid, *Indosinosuchus*, *Platysuchus*, *Mycterosuchus*, *Aeolodon* and *Bathysuchus*); over 67% of total premaxilla length is posterior to the external nares (shared with *Plagiophthalmosuchus*, the Chinese teleosauroid, *I. potamosiamensis*, *Mycterosuchus*, *Bathysuchus* and *Aeolodon*); anteromedial projection of the frontal is relatively broad but becomes immediately mediolaterally thin at the anterior-most part (shared with *Teleosaurus*); basioccipital tubera reduced (shared with *Plagiophthalmosuchus*, *Mycterosuchus* and *Bathysuchus*); five premaxillary alveolar pairs (shared with *Platysuchus*, *Teleosaurus* and *Bathysuchus*); the P1 and P2 alveoli are lateral to each other at the anterior margin of the premaxilla (shared with *Mycterosuchus*, *Bathysuchus* and possibly *Aeolodon*); the P3 and P4 are aligned on the lateral plane of the external margin more so than P2 (shared with *Bathysuchus*); the P1 and P2 are on the same transverse plane, and the lateral margin between the P2 and P3 is sub-rectangular (shared with *Mycterosuchus*, *Bathysuchus* and *Aeolodon*); lack of apical carinae (shared with *Bathysuchus*); shallow tuberculum (shared with *Aeolodon*, *Macrospondylus* and *Charitomenosuchus*); postacetabular iliac process elongated (shared with *Plagiophthalmosuchus*, *Platysuchus*, *Teleosaurus* and *Macrospondylus*); dorsal osteoderm pits are subcircular and organised in sub-parallel rows.

**Remarks**—*Sericodon* was initially diagnosed by Von Meyer (1845) but since the late 1800s has been considered a subjective junior synonym of ‘*Steneosaurus*’ (Sauvage, 1896; Sauvage, 1897; Von Huene, 1926; Kuhn, 1936; Steel, 1973; Buffetaut et al., 1985). *Sericodon* differs from *Bathysuchus* in the following characteristics:

1. *Sericodon* (TCH005-151; Schaefer, Püntener & Billon-Bruyat, 2018) lacks enamel ridges on the apices of the dentition, whereas *Bathysuchus* possesses faint but present enamel ridges (DORCM G.05067iv);
2. The lateral margins of the premaxillae are more expanded and sub-rectangular in *Bathysuchus* (NHMUK PV OR 43086; unnumbered LPP specimen). In *Sericodon* (SCR011-406; Schaefer, Püntener & Billon-Bruyat, 2018) they are less laterally expanded with more rounded margins;
3. Frontal ornamentation is present in *Sericodon* (SCR010-312; Schaefer, Püntener & Billon-Bruyat, 2018) but is absent in *Bathysuchus* (unnumbered LPP specimen), in specimens of approximately equal size;
4. A distinct groove between the two distinct quadrate condyles is present in *Sericodon* (SCR010-312; Schaefer, Püntener & Billon-Bruyat, 2018), whereas in *Bathysuchus* (unnumbered LPP specimen) the groove is nearly non-existent (although this may be due to preservation); and
5. The P3 alveoli is substantially larger than both the P1 and P2 in *Sericodon* (SCR011-406; Schaefer, Püntener & Billon-Bruyat, 2018). In *Bathysuchus* (DORCM G.05067i), the P3 is relatively the same size as the P2 and slightly larger than the P1.
6. Finally, *Sericodon* and *Bathysuchus* are always stable sister taxa in the phylogeny (see below), regardless of teleosauroid taxa and/or characters added or removed.

*Indosinosuchus* Martin et al., 2019

**Type species**—*Indosinosuchus potamosiamensis* Martin et al., 2019.

**Etymology**—‘Indochinese crocodile’. Refers to the Indochinese micro-tectonic block where the fossil was discovered, and *suchus* is the Latinized form of the Greek *soukhos* (σοῦχος), meaning crocodile.

**Diagnosis**—tooth row and quadrate condyle are unaligned with quadrate at a lower level, but both are below the occipital condyle; faint to no conspicuous maxillary ornamentation; approximately 30 alveoli per dentary.

*Indosinosuchus potamosiamensis* Martin et al., 2019

(Fig. 12)

**Figure 12:**
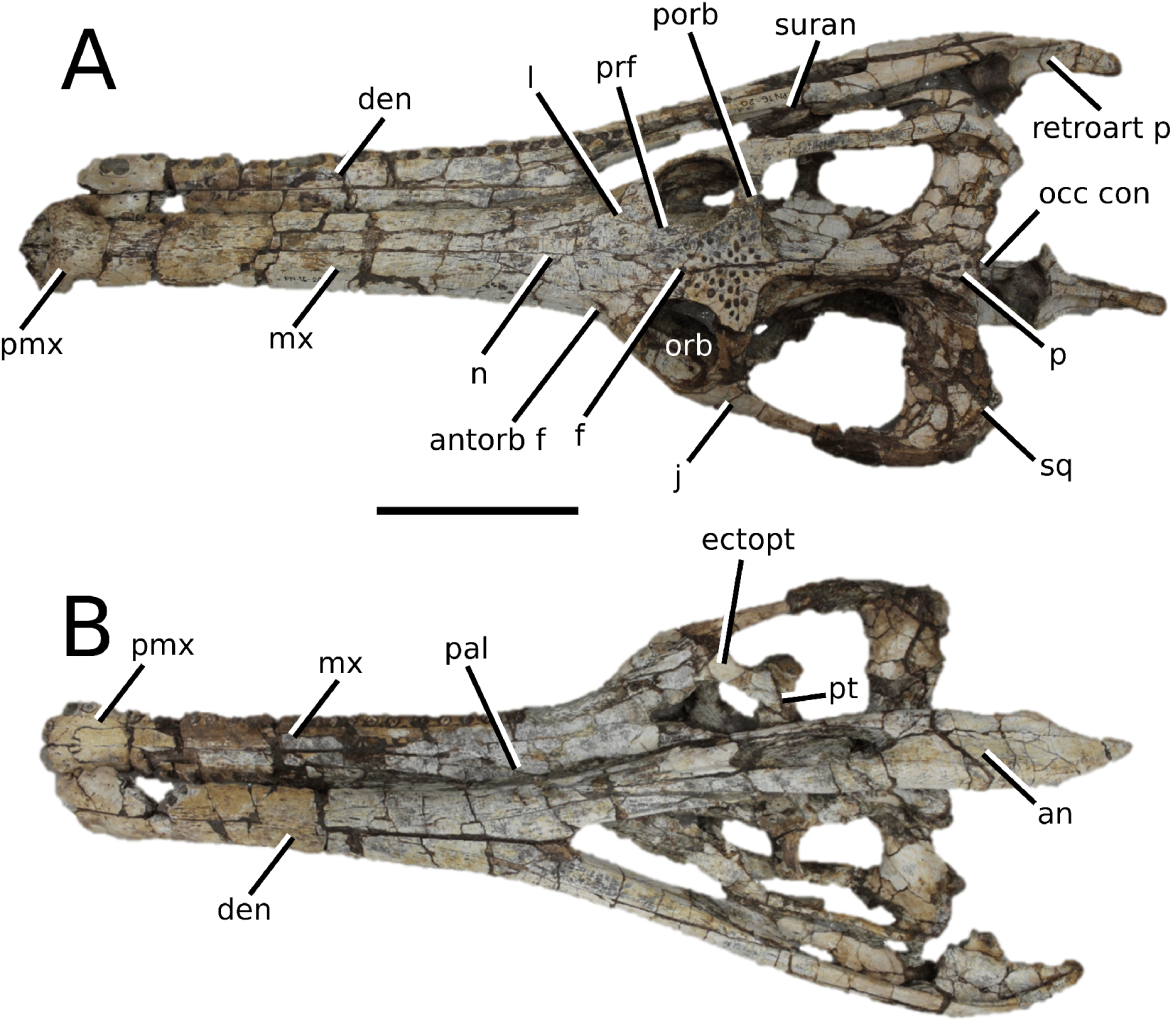
*Indosinosuchus potamosiamensis*. *Indosinosuchus potamosiamensis* Martin et al., 2019, PRC-11, holotype. Skull and attached mandible in (A) dorsal and (B) ventral (palatal) views. Refer to abbreviations list. Scale bar: 10 cm.

**Holotype**—PRC-11, a complete skull and mandible.

**Referred material**—PRC-238

**Age**—Late Jurassic (exact age is unknown, hypothesised to be Tithonian).

**Locality**—Pho Noi, Phu Phan range, Kham Muang District, Kalasin Province, northeastern Thailand.

**Stratigraphic horizon**—lower part of the Phu Kradung Formation, Khorat Group.

**Scoring sources**—the holotype (PRC-11) as well as PRC-238 were examined first-hand. Additional information was gleaned from Martin et al. (2019).

**Autapomorphic characters of *I. potamosiamensis***—extremely anteroposteriorly elongated posterior nasal processes (reaching the medial margin of the orbit); substantially elongated anterior process of the nasal, near-parallel to the posterior margin of the antorbital fenestra; the D2–D3 interalveolar space is longer than that between the D1 and D2.

**Emended diagnosis**—mesorostrine snout; tooth row and quadrate condyle unaligned with quadrate at a lower level, and both below the occipital condyle (shared with *I. kalasinensis* and *Mycterosuchus*); tooth row at a lower level than occipital condyle (shared with *Plagiophthalmosuchus*, *I. kalasinensis*, *Platysuchus*, *Teleosaurus*, *Mycterosuchus* and *Macrospondylus*); rostrum narrows immediately anterior to orbits (shared with *Teleosaurus*, *Mycterosuchus*, *Aeolodon*, *Bathysuchus* and *Sericodon*); shallow, irregular maxillary ornamentation consisting of grooves (similar to the Chinese teleosauroid, *Bathysuchus* and *Aeolodon*); no conspicuous ornamentation on both the prefrontal and lacrimal (similar to *Plagiophthalmosuchus*, *Aeolodon* and *Sericodon*); frontal ornamentation extends from the centre to lateral- and anterior-most regions (shared with *Plagiophthalmosuchus*, the Chinese teleosauroid, *I. kalasinensis*, *Platysuchus*, *Teleosaurus*, *Mycterosuchus*, *Macrospondylus* and *Clovesuurdameredeor*); external nares oriented anterodorsally (shared with the Chinese teleosauroid, *I. kalasinensis*, *Platysuchus*, *Mycterosuchus*, *Aeolodon*, *Bathysuchus* and *Sericodon*); over 67% of premaxilla total length is posterior to the external nares (shared with *Plagiophthalmosuchus*, the Chinese teleosauroid, *Mycterosuchus*, *Bathysuchus*, *Sericodon* and *Aeolodon*); presence of small, oval-shaped antorbital fenestrae; anterior margin of the supratemporal fossae are noticeably inclined anterolaterally (shared with *Mystriosaurus*, the Chinese teleosauroid, *I. kalasinensis*, *Platysuchus*, *Teleosaurus*, *Mycterosuchus*, *Bathysuchus* and *Aeolodon*); frontal width narrower than orbital width (shared with *Charitomenosuchus*); dorsal margins of orbits upturned (shared with *Teleosaurus*, *Mycterosuchus* and *Aeolodon*); postorbital reaches the orbit posteroventral margin and forms an extensive area of the orbit ventral margin (shared with *Mystriosaurus*, the Chinese teleosauroid, *Platysuchus*, *Teleosaurus* and *Mycterosuchus*); palatine anterior margin terminates level to 17th or 18th maxillary alveoli (similar to *Charitomenosuchus* and *Mac. buffetauti*); symphysis under half of mandible length, between 0.45 and 0.5 (shared with *Mystriosaurus*, *Deslongchampsina* and *Proexochokefalos*); mandibular fenestra anteroposteriorly small and poorly elliptic (similar to *Mystriosaurus*); at least 27 maxillary alveolar pairs; third premaxillary alveolus are enlarged relative to adjacent alveoli (shared with the Chinese teleosauroid); at least 30 dentary alveoli.

*Indosinosuchus kalasinensis* **sp. nov.**

(Fig. 13)

**Figure 13:**
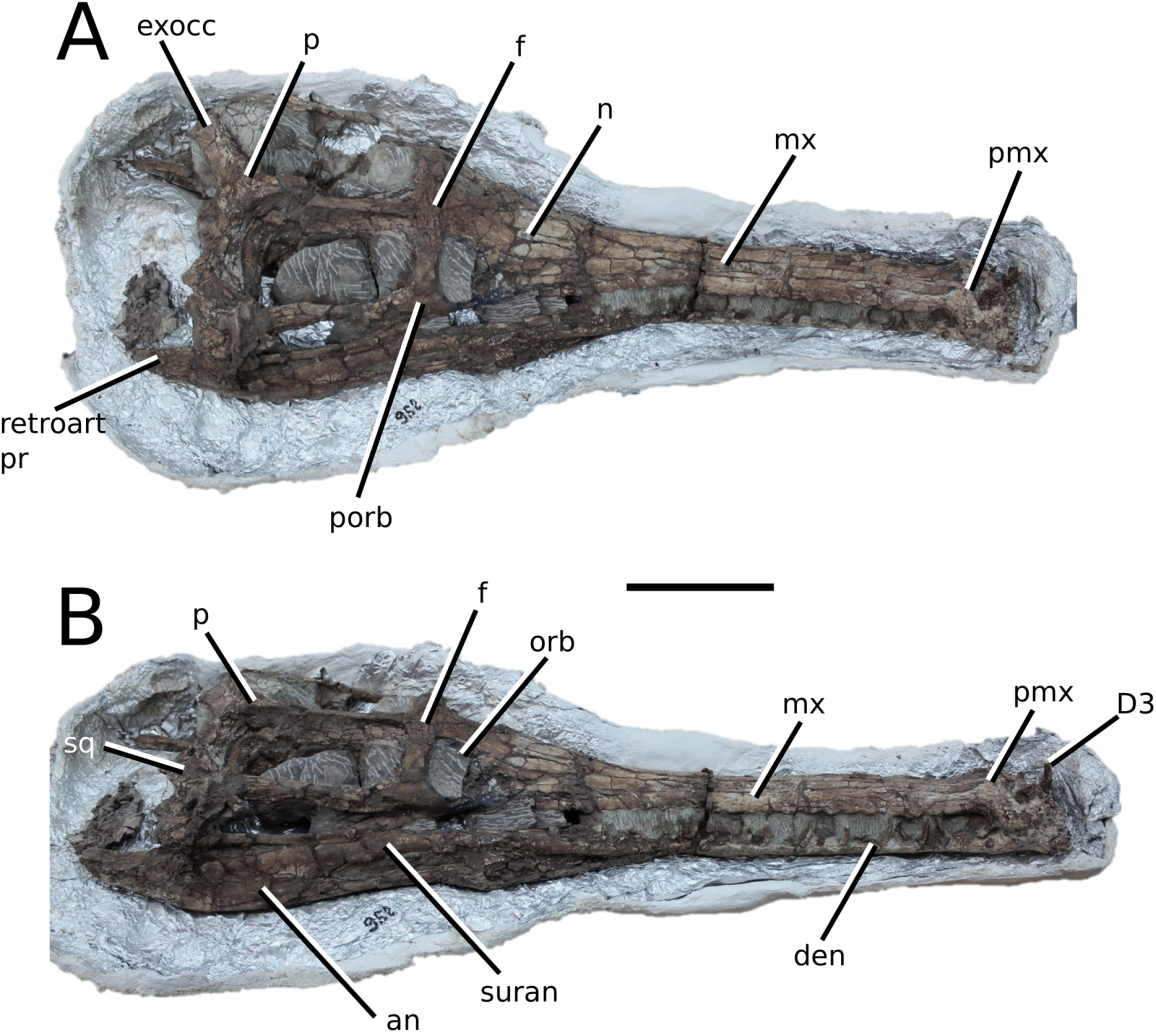
*Indosinosuchus kalasinensis*. *Indosinosuchus kalasinensis*, sp. nov., PRC-239. Skull and mandible in (A) dorsal and (B) right lateral views. Refer to abbreviations list. Scale bar: 10 cm.

**Holotype**—PRC-239, a nearly complete skull and mandible.

**Etymology**—the specific epithet refers to the Kalasin Province in northeastern Thailand where the holotype was found. urn:lsid:zoobank.org:act:2B7DB5BB-1F93-457F-A295-0409ECCD3998

**Age**—Late Jurassic (exact age is unknown, hypothesised to be Tithonian).

**Locality**—Pho Noi, Phu Phan range, Kham Muang District, Kalasin Province, northeastern Thailand.

**Stratigraphic horizon**—lower part of the Phu Kradung Formation, Khorat Group.

**Scoring Sources**—PRC-239 was examined first-hand.

**Autapomorphic characters of *I. kalasinensis***—approximately 64% of total premaxilla length is posterior to the external nares; anteroposteriorly thickened postorbital bar.

**Emended diagnosis**—mesorostrine snout; tooth row and quadrate condyle unaligned with quadrate at a lower level, and both below the occipital condyle (shared with *I. potamosiamensis* and *Mycterosuchus*); tooth row at a lower level than occipital condyle (shared with *Plagiophthalmosuchus*, *I. potamosiamensis*, *Platysuchus*, *Teleosaurus*, *Mycterosuchus* and *Macrospondylus*); premaxilla and maxilla ornamented with shallow ridges (similar to the Chinese teleosauroid, *I. potamosiamensis*, *Bathysuchus*, *Sericodon* and *Aeolodon*); frontal ornamentation extends from the centre to lateral- and anterior-most regions (shared with *Plagiophthalmosuchus*, the Chinese teleosauroid, *I. potamosiamensis*, *Platysuchus*, *Teleosaurus*, *Mycterosuchus*, *Macrospondylus* and *Clovesuurdameredeor*); enlarged premaxillary foramina lateral to the external nares (similar to *Mystriosaurus* and *Yvridiosuchus*); external nares oriented anterodorsally (shared with *Plagiophthalmosuchus*, the Chinese teleosauroid, *I. potamosiamensis*, *Platysuchus*, *Mycterosuchus*, *Aeolodon*, *Bathysuchus* and *Sericodon*); dorsoventrally deep premaxilla (similar to *Mystriosaurus*); the anterior and anterolateral premaxillary margins are orientated anteroventrally and extend ventrally (shared with *Mystriosaurus*, the Chinese teleosauroid, *I. potamosiamensis*, *Platysuchus*, *Teleosaurus*, *Mycterosuchus*, *Bathysuchus* and *Aeolodon*); anterior margin of the supratemporal fossae are noticeably inclined anterolaterally (shared with *Mystriosaurus*, the Chinese teleosauroid, *I. potamosiamensis*, *Platysuchus*, *Teleosaurus*, *Mycterosuchus*, *Bathysuchus* and *Aeolodon*); frontal width subequal to orbital width (shared with the Chinese teleosauroid, *Macrospondylus*, *Clovesuurdameredeor*, *Seldsienean*, *Yvridiosuchus*, *Deslongchampsina*, *Proexochokefalos*, *Mac. hugii* and *Mac. rex*); large, slightly robust teeth (most notably in the posterior dental region) with a pointed apex (most similar to *Mystriosaurus*).

**Remarks**—Martin et al. (2019) initially referred PRC-239 to *Indosinosuchus potamosiamensis*; however, we designate PRC-239 as a separate species, *I. kalasinensis*, as it differentiates from the holotype (PRC-11) of *I. potamosiamensis* in several features:

1. Rostrum does not narrow immediately anterior to the orbits in PRC-239, whereas there is a noticeable narrowing of the rostrum in PRC-11;
2. Premaxillary and maxillary neurovascular foramina are nearly 2x larger in PRC-239 than PRC-11, notably in the premaxillae;
3. External nares ‘B’-shaped in anterior view in PRC-239, whereas in PRC-11 they are somewhat‘8-shaped’;
4. Premaxillary length posterior to the external nares is between 50-65% in PRC-239, whereas in PRC-11 the premaxilla length posterior to the external nares is over 67%;
5. Minimum width of the frontal is subequal to orbital width in PRC-239, whereas in PRC-11 the frontal width is noticeably narrower than the orbital width;
6. Dorsal margin of the orbit flush with the skull dorsal surface in PRC-239 (although this may be due to dorsoventral crushing) whereas in PRC-11 the dorsal margins of the orbits are prominently upturned; and
7. Poorly elliptic external mandibular fenestra in PRC-239, whereas in *I. potamosiamensis* the mandibular fenestra is highly elliptic (anteroposteriorly elongated).

In addition, *I. kalasinensis* is never recovered as sister taxon to *I. potamosiamensis* in the phylogenetic analyses conducted below, and *I. kalasinensis* lacks all autapomorphies seen in *I. potamosiamensis*.

*Macrospondylus* Von Meyer, 1831

**Type species**—*Crocodilus bollensis* Jäger, 1828. Now referred to as *Macrospondylus bollensis* (Jäger, 1828), Von Meyer, 1831.

**Etymology**— ‘Large vertebra.’ *Macro* is from the Greek *makrýs* (μάκρος) meaning long, and *spondylus* is from the Ancient Greek *spóndylos* (σπόνδυλος) meaning vertebra. Refers to the long, amphicoelous vertebrae.

**Diagnosis**—same as the only known species (monotypic genus).

*Macrospondylus bollensis* (Jäger, 1828) Jäger, 1831

(Fig. 14)

**Figure 14:**
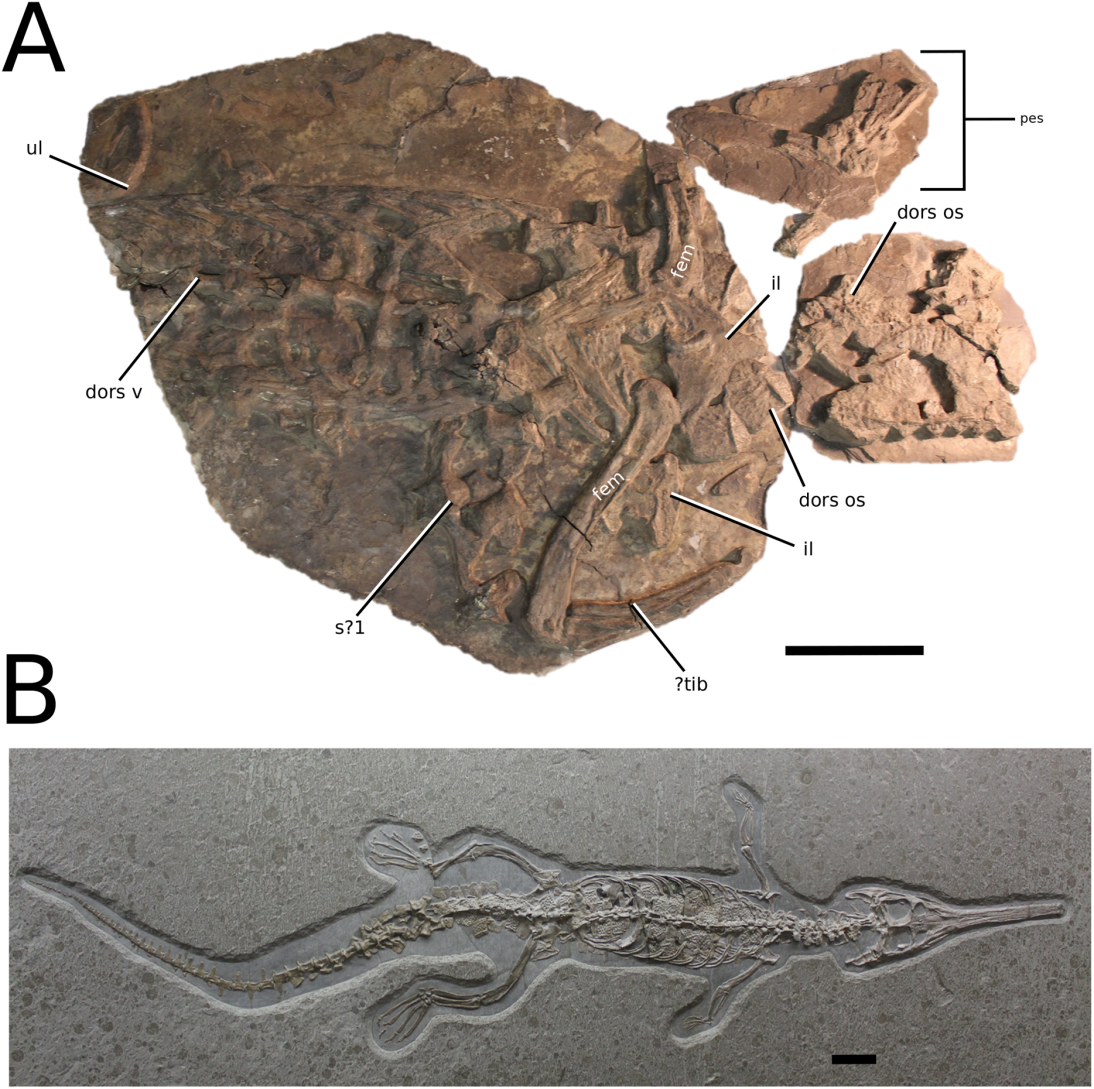
*Macrospondylus bollensis*. *Macrospondylus bollensis* (Jäger, 1828). (A) MMG BwJ 595, holotype, partial postcranial skeleton. (B) Complete skeleton MMG BwJ 565. Refer to abbreviations list. Scale bars: 10 cm.

**Holotype**—MMG BwJ 595, a partial postcranial skeleton, including dorsal, sacral and anterior caudal vertebrae, femora, one tibia, one fibula, one pes and disarticulated osteoderms.

**Referred material**—GPIT-RE-9427; MMG BwJ 565; MMG BwJ 689; NHMUK PV R 324; NHMUK PV R 756; NHMUK PV R 1088; NHMUK PV R 5703; NHMUK PV OR 14436; NHMUK PV OR 14438; NHMW-1848-0031-0001; NHMW-1878-0047-0001; NHMW-1882-0026-4082; PMU R161; SMNS 18672; SMNS 20280; SMNS 20283; SMNS 51555; SMNS 51563; SMNS 51753; SMNS 51957; SMNS 51984; SMNS 53422; SMNS 58876; SMNS 81699; SMNS 10 000 (all representing partial skulls, complete or near-complete skeletons); unnumbered OUMNH partial skull.

**Age**—early Toarcian, Early Jurassic.

**Localities**—Baden-Württemberg, Germany; Yorkshire, UK; Sanem, Luxembourg.

**Stratigraphic horizons**—Posidonia Shale Formation; Whitby Mudstone Formation; *Harpoceras serpentinum* ammonite Zone (‘*schistes bitumineux*’).

**Scoring sources**—the holotype (MMG BwJ 595), as well as a multitude of specimens from Germany, England and Luxembourg, were studied first-hand. Additional photographs were provided by B. Kear (PMU), M. Manabe (NMNSJ), U. Menkveld-Gfeller (NMBE), L. Schöllmann (LWL), A. Sennikov (PIN), W. Simpson (FMNH) and G. Wahlefeld (NMR).

**Autapomorphic characters of *Ma. bollensis***—the proximal region of the humerus is strongly proximodistally elongated and weakly posteriorly hooked; ulna with a well-developed distal curvature.

**Emended diagnosis**—longirostrine skull; tooth row at a lower level than the quadrate (shared with *Plagiophthalmosuchus*, *Platysuchus*, *Indosinosuchus*, *Teleosaurus* and *Mycterosuchus*); no conspicuous ornamentation on the lacrimal (shared with *Plagiophthalmosuchus*, *I. potamosiamensis*, *Bathysuchus*, *Aeolodon* and *Charitomenosuchus*); frontal ornamentation extends from the centre to lateral- and anterior-most regions (shared with *Plagiophthalmosuchus*, the Chinese teleosauroid, *Indosinosuchus*, *Platysuchus*, *Teleosaurus*, *Mycterosuchus* and *Clovesuurdameredeor*); external nares oriented dorsally (shared with *Plagiophthalmosuchus*, *Sericodon*, *Charitomenosuchus*, *Proexochokefalos*, *Deslongchampsina*, *Neosteneosaurus* and Machimosaurini); presence of shallow, slightly anteroposteriorly elongated antorbital fenestrae; no anterolateral expansion or inclination of the supratemporal fenestrae (shared with *Plagiophthalmosuchus*, *Clovesuurdameredeor*, *Charitomenosuchus*, *Seldsienean*, *Deslongchampsina*, *Proexochokefalos*, *Neosteneosaurus* and Machimosaurini); frontal width subequal to orbital width (shared with the Chinese teleosauroid, *I. kalasinensis*, *Clovesuurdameredeor*, *Seldsienean*, *Deslongchampsina*, *Proexochokefalos*, *Yvridiosuchus*, *Mac. hugii* and *Mac. rex*); orbit is longitudinal ellipsoid in shape (shared with *Plagiophthalmosuchus*, the Chinese teleosauroid, *Platysuchus*, *Aeolodon*, *Charitomenosuchus*, *Seldsienean*, *Proexochokefalos*, *Deslongchampsina* and *Neosteneosaurus*); basisphenoid exposed along the palatal surface, bifurcating the pterygoids (shared with *Charitomenosuchus*, *Deslongchampsina*, *Proexochokefalos*, *Neosteneosaurus*, *Yvridiosuchus* and *Lemmysuchus*); mandibular symphysis over 50% of mandible length (shared with *Mycterosuchus*, *Bathysuchus*, *Aeolodon*, *Seldsienean* and *Charitomenosuchus*); anterior maxillary teeth procumbent (shared with *I. kalasinensis*, *Platysuchus*, *Teleosaurus*, *Sericodon*, *Aeolodon* and *Charitomenosuchus*); tuberculum of dorsal rib situated on the medial edge (shared with *Platysuchus*, *Aeolodon* and *Lemmysuchus*); shallow tuberculum on the dorsal ribs (shared with *Sericodon*, *Aeolodon* and *Charitomenosuchus*); forelimb shorter than hindlimb by approximately 22-23% (similar to *Platysuchus*); tibia shorter than the femur by approximately 25% (similar to *Platysuchus*); femoral condyles are relatively the same size (shared with *Platysuchus*, *Aeolodon* and *Lemmysuchus*).

**Remarks**—the holotype of *Macrospondylus bollensis* (MMG BwJ 595) was one of the first well preserved vertebrate fossils housed in a scientific institution, dating back to 1755 (Von Meyer, 1831: 196). Johann Georg Gmelin, a chemist and pharmacist for the Royal Churfurstliche Naturaliengalerie Dresden, acquired it at the beginning of the 18th century. Von Meyer initially presented the holotype in an 1830 public talk (S. Sachs, 2019, personal communication), and both Dassdorff (1782) and Walch (1796) briefly noted it to be a crocodile skeleton (Von Meyer, 1831); it was then described by Cuvier (1812, 1824) as the iconic “*Gavial de Boll*” (“Boll gavial”). Jäger (1828) then named the specimen *Crocodilus bollensis*, and Von Meyer (1831, 1832) defined and described it as a new genus *Macrospondylus*. The holotype was badly burned in the Zwinger fire of May 1849 (during the Burgerliche revolution) but survived. Due to this damage, it has been suggested that it cannot be referable to other *Macrospondylus* specimens (M. Wilmsen, 2017, personal communication). However, MMG BwJ 595 displays a combination of postcranial features unique to *Macrospondylus* (e.g. SMNS 18672; SMNS 51563; SMNS 51753; SMNS 51957):

1. Large, anteroposteriorly elongated and dorsoventrally thin cervical ribs (most posteriorly placed);
2. Shallow tuberculum on dorsal ribs;
3. Ulna with well-developed, pronounced distal curvature that is noticeably larger than the distal part;
4. Anteroposteriorly short anterior iliac process;
5. Femoral condyles of relatively same size; and
6. Dorsal osteoderms with a pronounced keel and subcircular, numerous, separated pits.

*Seldsienean* **gen. nov.**

**Type species**—*Steneosaurus megistorhynchus* Eudes-Deslongchamps, 1866. Now referred to as *Seldsienean megistorhynchus* (Eudes-Deslongchamps, 1866) **comb. nov**. urn:lsid:zoobank.org:act:A5177ED2-1416-4C54-A169-05591DA55D80

**Etymology**— ‘Rare one’. *Seldsīene* is Old English for ‘rare’ or ‘seldom seen’ and ‘*-an*’ is Old English for ‘one’. Refers to the rarity of this taxon compared to other Bathonian teleosauroids.

**Diagnosis**—same as the only known species (monotypic genus).

*Seldsienean megistorhynchus* (Eudes-Deslongchamps, 1866) **comb. nov.**

(Fig. 15)

**Figure 15:**
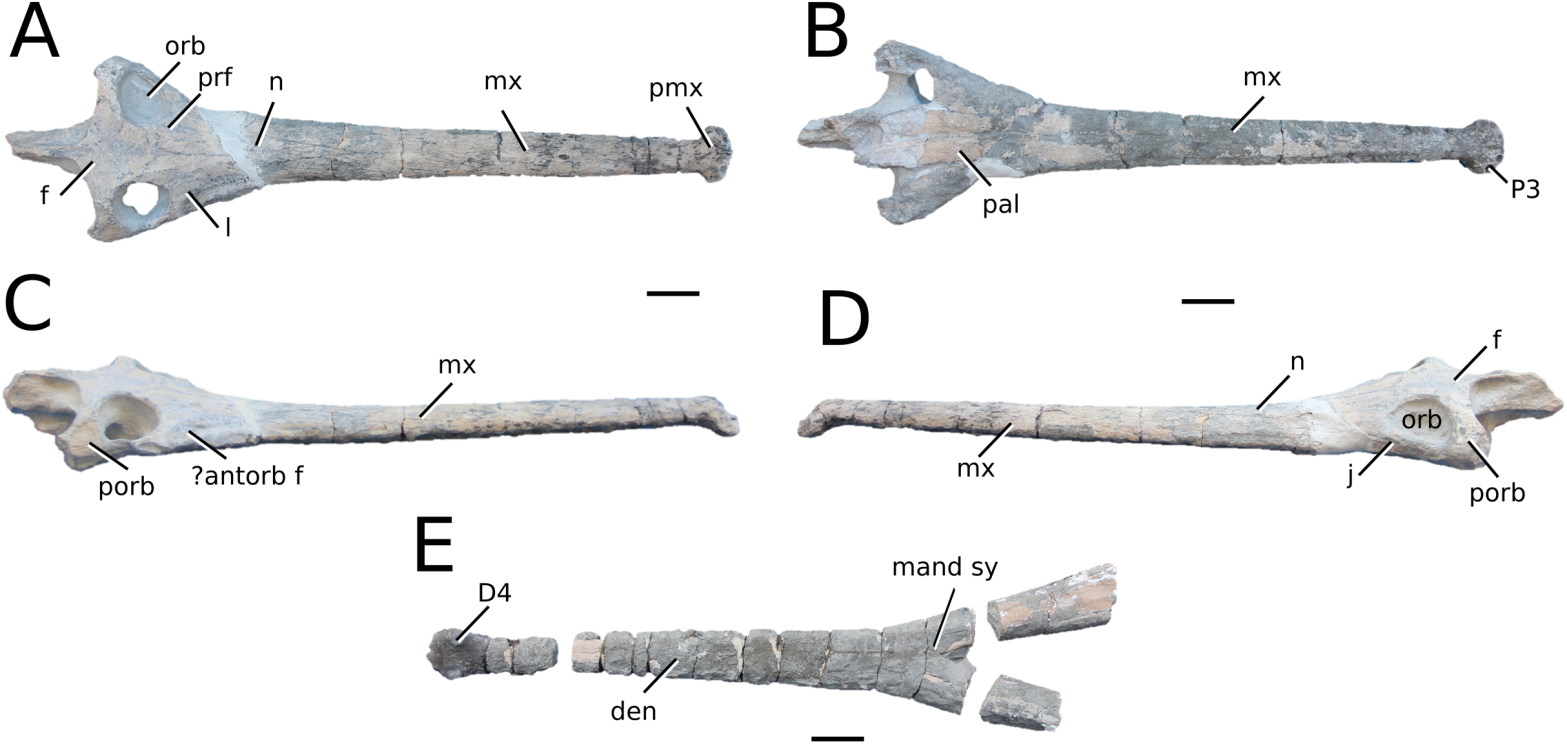
*Seldsienean megistorhynchus*. *Seldsienean megistorhynchus* (Eudes-Deslongchamps, 1866), **comb. nov.**, MMT P28-1, neotype. Skull in (A) dorsal, (B) ventral (palatal), (C) right lateral and (D) left lateral views. Mandible in (E) dorsal view. Refer to abbreviations list. Scale bars: 10 cm. Photographs provided by V. Lamarque.

**Holotype**—A partial skull and complete mandible initially described by Cuvier (1824), re-described by Eudes-Deslongchamps (1866, 1867), and presumed destroyed in 1944.

**Neotype**—MMT P28-1 (a partial skull and mandible, as well as isolated vertebrae, fragmented elements, and three osteoderms and teeth) (see Godefroit, Vignaud & Lieger, 1995 for additional information).

**Designation of neotype**—herein we formally designate MMT P28-1 as the neotype of *Se. megistorhynchus*. In order to be in full accordance of Article 75 of the ICZN Code, specifically Article 75.3, we make the following statements:

1. This designation is made with the objective of clarifying the taxonomic status of *Se. megistorhynchus*.
2. Our assertion of the characters that we regard as distinguishing *Se. megistorhynchus* from other teleosauroid taxa is listed in the species diagnosis below.
3. The neotype can be recognized through both the following diagnosis and Fig. 15.
4. The holotype is presumed destroyed in 1944 during the bombing of Caen.
5. The holotype, in addition to a partial skull, included a complete mandible; E. Eudes-Deslongchamps (1867: 217) stated that the holotype of *Se. megistorhynchus* consisted of a “*Museau très-allonge’, grêle, étroit et aplati dans toute sa longueur*” (“Very elongated muzzle, slender, narrow and flattened along its entire length”). As such, the neotype is consistent with what is known of the former name-bearing type.
6. Unfortunately, the locality of the neotype is not known. However, it and the holotype are from the same age (Bathonian) and country (France), and have been referred to as the same species.
7. *Se. megistorhynchus* is a slender, longirostrine form, which differs from the genera *Deslongchampsina* (mesorostrine) and *Yvridiosuchus* (durophagous), which are found in the same stratigraphic horizon and location. In addition, the neotype displays has several distinct features that differ from *Deslongchampsina* and *Yvridiosuchus* (e.g. telescopic orbits).
8. The neotype is the property of an internationally recognized scientific institution at the Musée d’art et d’histoire de Toul (MMT), which maintains a research collection with suitable facilities for preserving name-bearing types and is accessible for study.

**Referred material**— OUMNH J.1414 (near-complete mandible); LPP.T.1 (partial mandible).

**Age**—Bathonian, Middle Jurassic.

**Localities**—unspecified location in France; Enslow Bridge, Oxfordshire, UK.

**Stratigraphic horizons**— ‘*Calcaire de Caen*’; Cornbrash Formation, Great Oolite Group.

**Scoring Sources**—the referred specimens (LPP.T.1 and OUMNH J.1415) were studied first-hand. Additional information was taken from Eudes-Deslongchamps (1866, 1867).

**Autapomorphic characters of *Se. megistorhynchus***— small, circular, noticeably spaced ornamentation on prefrontal and lacrimal; extremely interdigitated anterior margin of the palatines; relatively deep, subcircular neurovascular foramina in the posterior region of the dentary, seen in lateral view; deep coronoid groove; dorsal osteoderms with large, irregularly shaped and elongated pits with raised areas in between pits, and a small yet well-developed keel situated in the middle of the osteoderm.

**Emended diagnosis**—longirostrine skull; rostrum narrows immediately anterior to the orbits (shared with *I. potamosiamensis*, *Teleosaurus*, *Mycterosuchus*, *Aeolodon*, *Bathysuchus* and *Sericodon*); frontal ornamentation restricted to centre (shared with *Sericodon*, *Aeolodon*, *Charitomenosuchus*, *Deslongchampsina*, *Proexochokefalos*, *Neosteneosaurus* and Machimosaurini); no anterolateral expansion or inclination of the supratemporal fenestrae (shared with *Plagiophthalmosuchus*, *Clovesuurdameredeor*, *Macrospondylus*, *Charitomenosuchus*, *Deslongchampsina, Proexochokefalos*, *Neosteneosaurus* and Machimosaurini); antorbital fenestra present; frontal width subequal to orbital width (shared with the Chinese teleosauroid, *I. kalasinensis*, *Clovesuurdameredeor*, *Macrospondylus*, *Deslongchampsina*, *Proexochokefalos*, *Yvridiosuchus*, *Mac. hugii* and *Mac. rex*); orbit is longitudinal ellipsoid in shape (shared with *Plagiophthalmosuchus*, the Chinese teleosauroid, *Platysuchus*, *Aeolodon*, *Macrospondylus*, *Charitomenosuchus*, *Proexochokefalos*, *Deslongchampsina* and *Neosteneosaurus*); mandibular symphysis over 50% of mandible length (shared with *Mycterosuchus*, *Bathysuchus*, *Aeolodon*, *Macrospondylus* and *Charitomenosuchus*); over 30 dentary alveoli per side (shared with *Plagiophthalmosuchus*, *Platysuchus*, *Bathysuchus*, *Mycterosuchus* and *Charitomenosuchus*).

**Remarks**—despite fragmentary material, we consider *Seldsienean* as a distinct taxon because it is the only longirostrine form present in the Great Oolite Group (UK) during the Bathonian.

*Charitomenosuchus* **gen. nov.**

**Type species**—*Steneosaurus leedsi* Andrews, 1909. Now referred to as *Charitomenosuchus leedsi* (Andrews, 1909), **comb. nov**. urn:lsid:zoobank.org:act:DE54456D-A305-4A5D-8209-A987982B200C

**Etymology**— ‘Graceful crocodile’. *Charitoménos* (χαριτωμένος) is Greek for ‘graceful’ (referring to the slender, elegant skull of this taxon) and *suchus* is the Latinized form of the Greek *soukhos* (σοῦχος), meaning crocodile.

**Diagnosis**—same as the only known species (monotypic genus).

*Charitomenosuchus leedsi* (Andrews, 1909) **comb. nov.**

(Fig. 16)

**Figure 16:**
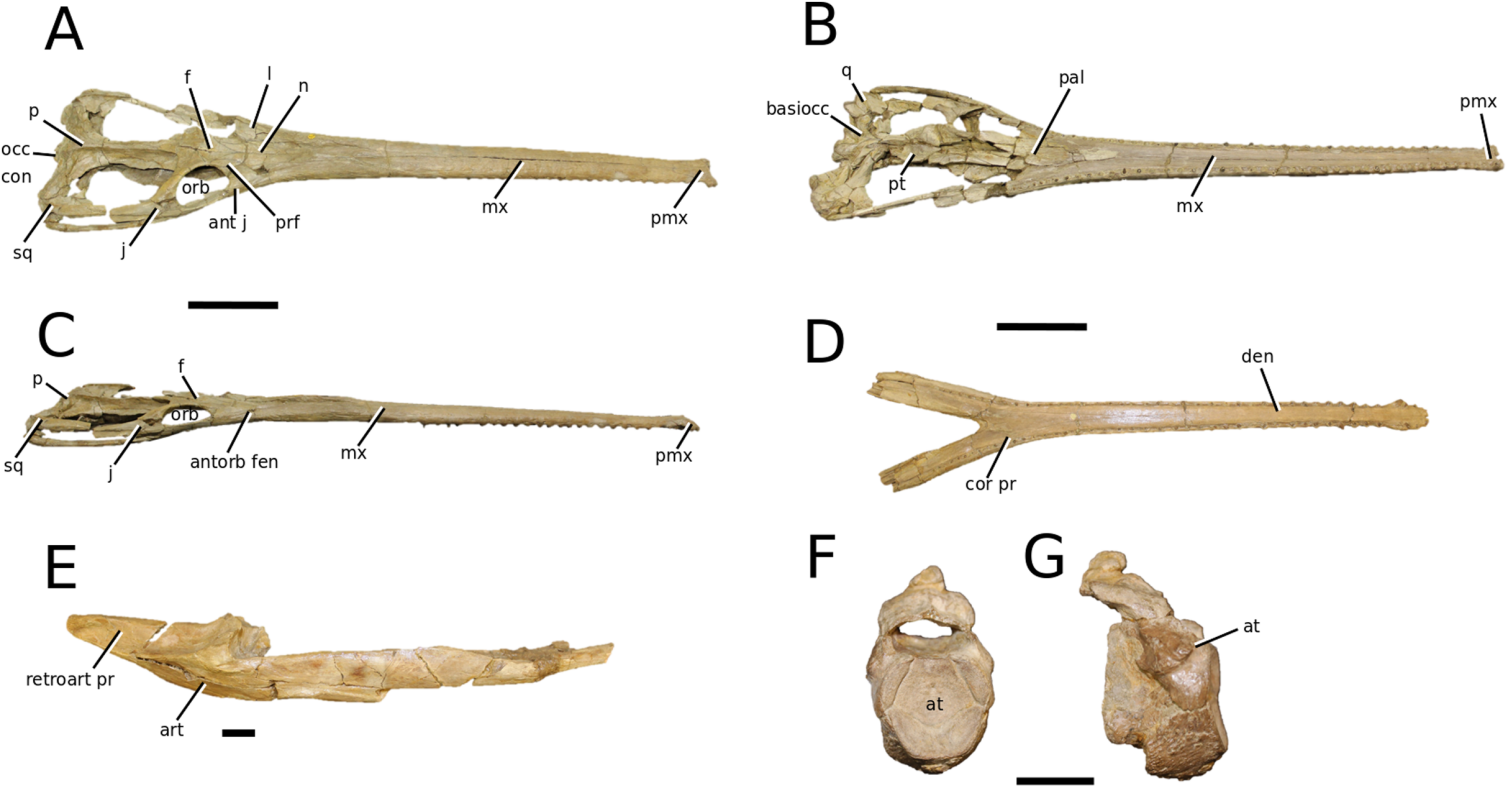
*Charitomenosuchus leedsi*. *Charitomenosuchus leedsi* (Andrews, 1913), **comb. nov.**, NHMUK PV R 3320, holotype. Skull in (A) dorsal, (B) ventral (palatal) and (C) right lateral views; partial mandible in (D) dorsal view. (E) Posterior section of the mandible in right lateral view; atlas in (F) anterior and (G) right lateral view. Refer to abbreviations list. Scale bars: 10 cm (A–D) and 2 cm (E–G).

**Holotype**—NHMUK PV R 3320, a nearly complete skull.

**Referred material**—BRLSI GP1770a-e (a complete skull and mandible); NHMUK PV R 2619 (a complete mandible and additional femora, ilia, ischia, pubes, tibiae, humeri, ulnae, radiae, ribs [cervical, dorsal], partially preserved vertebrae [two cervical, two dorsal, two sacral] and dorsal osteoderms); NHMUK PV R 3806 (a nearly complete skeleton); PETMG R179 (complete skull).

**Age**—Middle Callovian, Middle Jurassic.

**Locality**—Peterborough, UK.

**Stratigraphic horizon**—Peterborough Member, Oxford Clay Formation, Ancholme Group.

**Scoring Sources**—the holotype (NHMUK PV R 3320) as well as all referred specimens mentioned above were examined first-hand.

**Autapomorphic characters of *C. leedsi***—frontal ornamentation consists of circular, spaced apart pits limited to the centre-most and posterior frontal; strongly interdigitating premaxilla-maxilla suture; narrow mediolateral supratemporal fenestra width (relative to other teleosauroids); supratemporal arch dorsal margin subtly concave in lateral view; neural spine height of anterior thoracic vertebrae is less than centrum height; dorsal osteoderms with large, subcircular well-spaced pits arranged in a semi-parallel pattern; mediolaterally thickened keel on sacral osteoderms.

**Emended diagnosis**—longirostrine, gracile skull; tooth row and occipital condyle aligned, and quadrate condyle at a lower level (shared with the Chinese teleosauroid, *Proexochokefalos*, *Neosteneosaurus* and Machimosaurini); skull width less than 26% of skull length (shared with *Plagiophthalmosuchus*, *Mycterosuchus*, *Bathysuchus* and *Aeolodon*); no ornamentation on the lacrimal (shared with *Plagiophthalmosuchus*, *I. potamosiamensis*, *Aeolodon* and *Macrospondylus*); external nares oriented dorsally (shared with *Plagiophthalmosuchus*, *Macrospondylus*, *Deslongchampsina*, *Proexochokefalos*, *Neosteneosaurus* and Machimosaurini); premaxilla anterior and anterolateral margins are not subvertical (shared with *Plagiophthalmosuchus*, *Macrospondylus*, *Andrianavoay*, *Deslongchampsina*, *Proexochokefalos*, *Neosteneosaurus* and Machimosaurini); frontal width narrower than orbital width (shared with *I. potamosiamensis*); orbit is longitudinal ellipsoid in shape (shared with *Plagiophthalmosuchus*, the Chinese teleosauroid, *Platysuchus*, *Aeolodon*, *Macrospondylus*, *Seldsienean*, *Proexochokefalos*, *Deslongchampsina* and *Neosteneosaurus*); the anterior process of the jugal is slender, elongated and extends anteriorly (shared with *Clovesuurdameredeor*, *Proexochokefalos*, *Neosteneosaurus* and Machimosaurini); palatine anterior margin terminates level to 15th to 19th maxillary alveoli (shared with *I. potamosiamensis* and *Mac. buffetauti*); basisphenoid exposed along the palatal surface, bifurcating the pterygoids (shared with *Macrospondylus*, *Deslongchampsina*, *Proexochokefalos*, *Neosteneosaurus*, *Yvridiosuchus* and *Lemmysuchus*); the mandibular symphysis is over 50% of the mandible length (shared with *Bathysuchus*, *Mycterosuchus*, *Macrospondylus*, *Aeolodon* and *Seldsienean*); mandibular symphysis depth is very narrow, approximately 4–4.5% of the mandible length (shared with *Mycterosuchus*); the P1 is oriented anteriorly whereas the P2 is oriented slightly medially (shared with *Proexochokefalos*); over 30 dentary alveoli per side (shared with *Plagiophthalmosuchus*, *Platysuchus*, *Bathysuchus*, *Mycterosuchus* and *Seldsienean*); slender teeth with weak mediolateral compression (shared with *Macrospondylus*); neural spine height of mid-cervical vertebrae is approximately equal to centrum height (similar to *Aeolodon*); the tuberculum and articular facet are situated directly in the dorsal rib (shared with *Mycterosuchus*); the dorsal rib tuberculum is shallow (shared with *Sericodon*, *Aeolodon* and *Macrospondylus*); proximal humerus strongly posteriorly deflected and hooked (similar to *Aeolodon*, *Macrospondylus* and *Neosteneosaurus*); supraacetabular iliac crest is shallow and poorly pronounced (shared with *Neosteneosaurus*, *Lemmysuchus* and *Mac. mosae*); postacetabular iliac process is fan-shaped (shared with *Neosteneosaurus*, *Lemmysuchus* and *Mac. mosae*); tibia approximately 40–50% shorter than the femur (shared with *Mycterosuchus*, *Neosteneosaurus*, *Lemmysuchus* and *Mac. mosae*); medial femoral condyle larger than lateral femoral condyle (shared with *Mycterosuchus*, *Neosteneosaurus* and *Machimosaurus*).

**Remarks**—Both Vignaud (1995) and Mueller-Töwe (2006) considered *Mycterosuchus nasutus* to be a synonym of *Steneosaurus leedsi* (= *Charitomenosuchus leedsi*).

*Deslongchampsina* Johnson, Young & Brusatte, 2019

**Type species**—*Steneosaurus larteti* Eudes-Deslongchamps, 1866. Now referred to as *Deslongchampsina larteti* (Eudes-Deslongchamps, 1866) Johnson, Young & Brusatte, 2019.

**Etymology**—Named after Jacques Amand and Eugène Eudes-Deslongchamps, father and son French naturalists who thoroughly described the holotype specimen and additional teleosauroid material.

**Diagnosis**—same as the only known species (monotypic genus).

*Deslongchampsina larteti* (Eudes-Deslongchamps, 1866) Johnson, Young & Brusatte, 2019

(Fig. 17)

**Figure 17:**
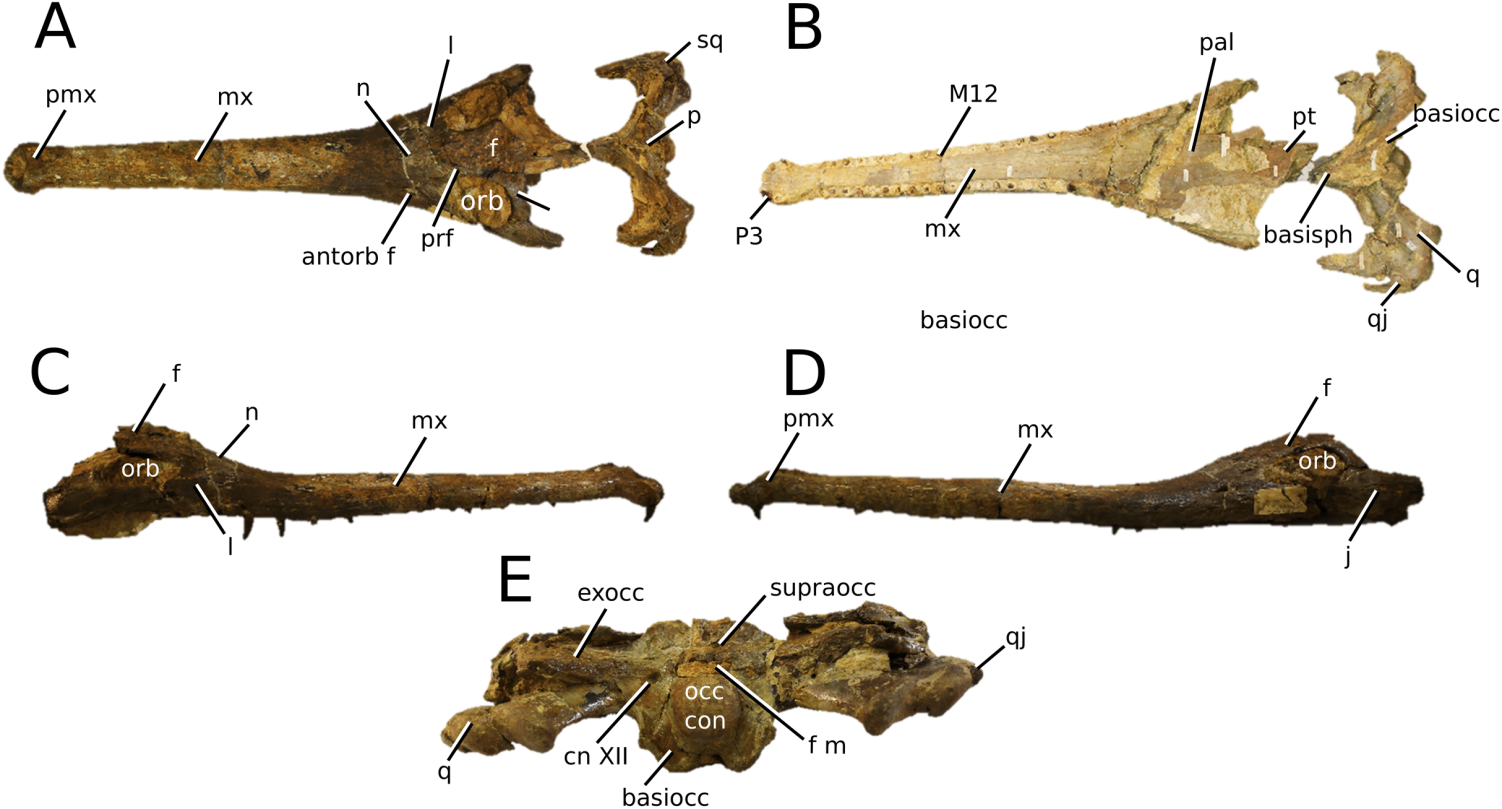
*Deslongchampsina larteti*. *Deslongchampsina larteti* (Eudes-Deslongchamps, 1866) Johnson, Young & Brusatte, 2019, OUMNH J.29851, neotype. Skull in (A) dorsal, (B) ventral (palatal), (C) right lateral, (D) left lateral and (E) occipital views. Refer to abbreviations list. Scale bars: 5 cm.

**Holotype**—A partial skull associated with a partial symphyseal section of the mandible, pelvis, hindlimb, two vertebrae and dorsal osteoderms. Destroyed in 1944.

**Neotype**—OUMNH J.29851, a partial skull broken into two pieces. Neotype designation by Johnson, Young & Brusatte (2019).

**Age**—Bathonian, Middle Jurassic.

**Localities**—Calvados, France; Enslow Bridge, Oxfordshire, UK.

**Stratigraphic horizons**— ‘*Fuller’s Earth inférieur*’; Cornbrash Formation, Great Oolite Group.

**Scoring Sources**—the neotype (OUMNH J.29851) was studied first-hand.

**Autapomorphic characters of *D. larteti***—feeble constriction of the premaxillae posterior to the external nares, giving the premaxillae a more rounded, ‘globular’ appearance in dorsal and ventral views; posterior processes of the nasals are mediolaterally thin; gradual and well-developed anteroventral sloping of the nasals. See Johnson, Young & Brusatte (2019) for more detail.

**Emended diagnosis**—mesorostrine snout; frontal ornamentation restricted to the centre (shared with *Sericodon*, *Aeolodon*, *Seldsienean*, *Charitomenosuchus*, *Proexochokefalos*, *Neosteneosaurus* and Machimosaurini); external nares oriented dorsally (shared with *Plagiophthalmosuchus*, *Macrospondylus*, *Charitomenosuchus*, *Proexochokefalos*, *Neosteneosaurus* and Machimosaurini); premaxilla anterior and anterolateral margins are not sub-vertical (shared with *Plagiophthalmosuchus*, *Macrospondylus*, *Andrianavoay*, *Charitomenosuchus*, *Proexochokefalos*, *Neosteneosaurus* and Machimosaurini); presence of large, anteroposteriorly elongated antorbital fenestrae, and internal antorbital fenestra over 25% of the length of the orbit (shared with *Plagiophthalmosuchus*); orbit is longitudinal ellipsoid in shape (shared with *Plagiophthalmosuchus*, the Chinese teleosauroid, *Platysuchus*, *Aeolodon*, *Macrospondylus*, *Charitomenosuchus*, *Seldsienean*, *Proexochokefalos* and *Neosteneosaurus*); frontal width subequal with orbital width (shared with the Chinese teleosauroid, *Mycterosuchus*, *Proexochokefalos*, *Yvridiosuchus*, *Mac. hugii* and *Mac. rex*); small basioccipital tuberosities (similar to *Bathysuchus*); palatine anterior margin terminates distal to the 20th maxillary alveoli (shared with *Charitomenosuchus*, *Mycterosuchus* and *Bathysuchus*); mandibular symphysis slightly less than half the mandibular length, between 45 and 50% (shared with *Mystriosaurus*, *I. potamosiamensis* and *Proexochokefalos*); deep, well-developed reception pits throughout the anterior- to mid-maxilla and gradually disappear (similar to *Mystriosaurus*, *Charitomenosuchus* and *Proexochokefalos*); teeth are robust, slightly curved and weakly-compressed, with pointed apices and high relief enamel ridges (similar to *Neosteneosaurus*).

*Proexochokefalos* **gen. nov.**

**Type species**—*Steneosaurus heberti* Morel de Glasville, 1876. Now referred to as *Proexochokefalos heberti* (Morel de Glasville, 1876), **comb. nov.** urn:lsid:zoobank.org:act:FC885641-54CC-421D-84E7-0341140EB704

**Etymology**— ‘Big head with big tuberosities’. *Proexochi* (προεξοχή) is Greek for projection/tuberosity (in an anatomical sense), referring to the large occipital tuberosities that are characteristic of this taxon, and *kefálo[s]* (κεφάλι) is Greek meaning head.

**Diagnosis**—mesorostrine snout; lack of a midline cavity (= trench) on the nasals; well-developed occipital tuberosities.

*Proexochokefalos heberti* (Morel de Glasville, 1876) **comb. nov.**

(Fig. 18)

**Figure 18:**
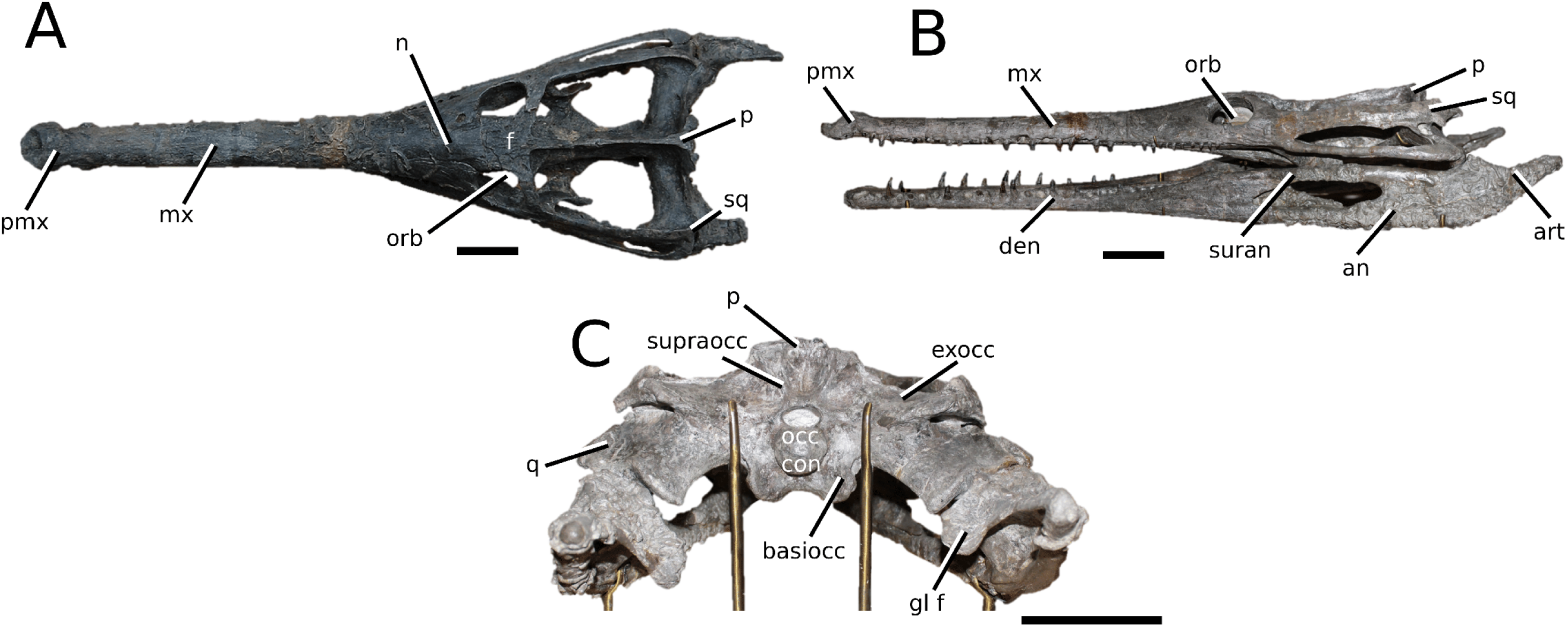
*Proexochokefalos heberti*. *Proexochokefalos heberti* (Morel de Glasville, 1876), **comb. nov.**, MNHN.F 1890-13, holotype. Skull in (A) dorsal, (B) left lateral and (C) occipital views. Refer to abbreviations list. Scale bars: 10 cm.

**Holotype**—MNHN.F 1890-13, a complete skull and mandible.

**Age**—upper Callovian, Middle Jurassic.

**Locality**—Villers-sur-mer, Calvados, France.

**Stratigraphic horizon**—Marnes de Dives Formation.

**Scoring sources**—the holotype (MNHN.F 1890-13) was studied first-hand.

**Autapomorphic characters of *Pr. heberti***—premaxillae dorsoventrally high in lateral view (approximately 38 mm dorsoventral length, from dorsal-most area to tooth row); occipital tuberosities large and well-developed; slightly mediolaterally compressed teeth with pointed apices throughout the dentary series; faint enamel ridges on apical third of teeth; 79-80° posterior curvature of the teeth throughout the entire dental series.

**Emended diagnosis**—mesorostrine skull; tooth row and occipital condyle aligned, and quadrate condyle at a lower level (shared with the Chinese teleosauroid, *Charitomenosuchus*, *Pr*. cf. *bouchardi*, *Neosteneosaurus* and Machimosaurini); frontal ornamentation restricted to centre (shared with *Sericodon*, *Aeolodon*, *Charitomenosuchus*, *Seldsienean*, *Deslongchampsina*, *Neosteneosaurus* and Machimosaurini); external nares oriented dorsally (shared with *Plagiophthalmosuchus*, *Macrospondylus*, *Charitomenosuchus*, *Deslongchampsina*, *Neosteneosaurus* and Machimosaurini); anterior and anterolateral margins of the supratemporal fenestrae are not sub-vertical (shared with *Plagiophthalmosuchus*, *Macrospondylus*, *Andrianavoay*, *Charitomenosuchus*, *Deslongchampsina*, *Neosteneosaurus* and Machimosaurini); flat nasals with no evidence of a midline concavity (shared with *Pr*. cf. *bouchardi*); absence of antorbital fenestrae (shared with *Neosteneosaurus* and Machimosaurini excluding *Yvridiosuchus*); supratemporal fenestra length is twice as long as the anterior width (shared with *Pr*. cf. *bouchardi* and *Neosteneosaurus*, and somewhat similar to Machimosaurini); orbit is longitudinal ellipsoid in shape (shared with *Plagiophthalmosuchus*, the Chinese teleosauroid, *Platysuchus*, *Aeolodon*, *Macrospondylus*, *Charitomenosuchus*, *Seldsienean*, *Pr*. cf. *bouchardi*, *Deslongchampsina* and *Neosteneosaurus*); frontal width sub-equal to orbital width (shared with the Chinese teleosauroid, *I. kalasinensis*, *Macrospondylus*, *Clovesuurdameredeor*, *Seldsienean*, *Deslongchampsina*, *Yvridiosuchus*, *Mac. hugii* and *Mac. rex*); anterior process of the jugal is slender and anteriorly elongated (shared with *Clovesuurdameredeor*, *Charitomenosuchus*, *Neosteneosaurus* and Machimosaurini); mandibular symphysis slightly less than half the mandibular length, between 45 and 50% (shared with *Mystriosaurus*, *I. potamosiamensis* and *Deslongchampsina*); deep, well-developed reception pits throughout the anterior- to mid-maxilla and gradually disappear (similar to *Mystriosaurus*, *Charitomenosuchus* and *Deslongchampsina*); shallow Meckelian groove (shared with *Neosteneosaurus* and Machimosaurini); sharp dorsal curvature of the angular (shared with *Neosteneosaurus* and Machimosaurini); the P1 is oriented anteriorly whereas the P2 is oriented slightly medially (shared with *Proexochokefalos*).

*Proexochokefalos* cf. *bouchardi* (Sauvage, 1872) **comb. nov.**

(Fig. 19)

**Figure 19:**
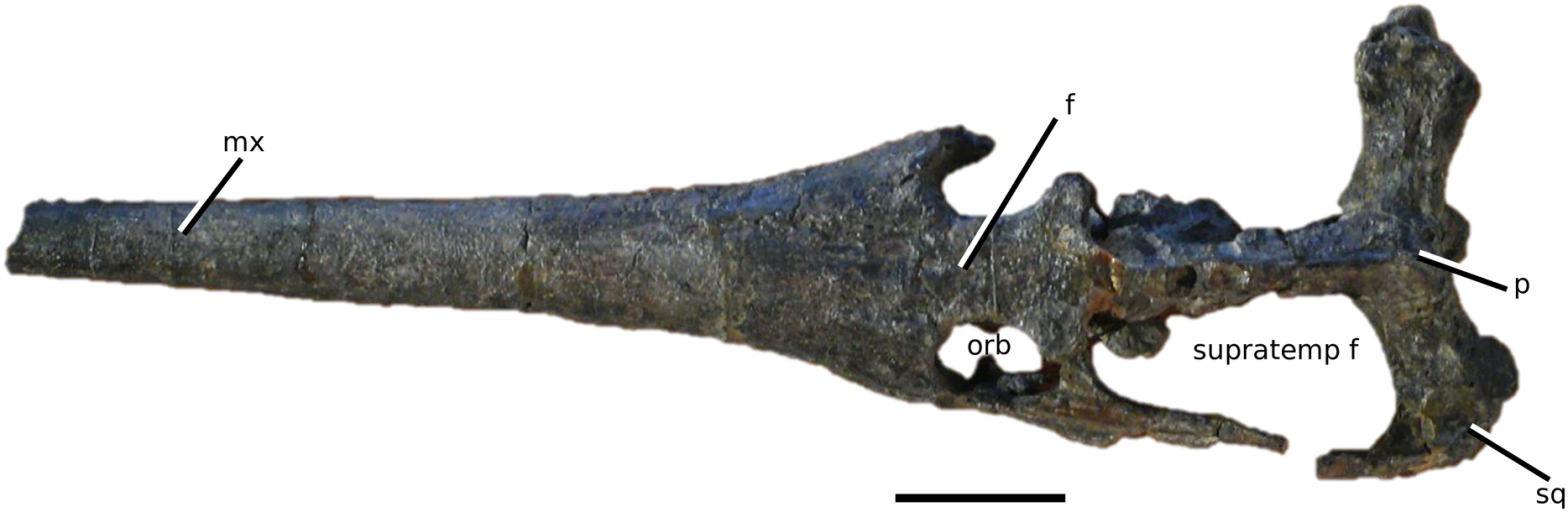
*Proexochokefalos* cf. *bouchardi*. *Proexochokefalos* cf. *bouchardi* (Sauvage, 1872), **comb. nov.** Unknown specimen number, photo provided by Y. Lepage (from Lepage et al., 2008). Skull in dorsal view. Refer to abbreviations list. Scale bar: 10 cm.

**Holotype**—A partial specimen initially composed of a skull, mandible and assorted vertebrae (Vignaud, 1995). Currently missing and/or destroyed.

**Referred material**—Sauvage (1872); Buffetaut & Makinsky (1984); Lepage et al. (2008); SCR010-374 (Schaefer, Püntener & Billon-Bruyat, 2018).

**Age**—Kimmeridgian, Late Jurassic.

**Localities**—Villerville, Calvados, France; Courtedoux-sur Combe Ronde, northwestern Switzerland.

**Stratigraphic horizons**— ‘*Calcaire de Caen*’; Reuchenette Formation.

**Scoring sources**—Scores were based on specimen photographs from Lepage et al. (2008) and Schaefer, Püntener & Billon-Bruyat (2018). Additional information was read from Joleaud (1928) and Buffetaut & Makinsky (1984).

**Emended diagnosis**—mesorostrine skull; tooth row and occipital condyle aligned in the same plane (similar to the Chinese teleosauroid, *Charitomenosuchus*, *Pr. heberti*, *Neosteneosaurus* and Machimosaurini); flat nasals with no evidence of a midline concavity (shared with *Pr. heberti*); supratemporal fenestrae length is twice as long as width (shared with *Pr. heberti* and *Neosteneosaurus*, and somewhat similar to Machimosaurini); frontal width broader than orbital width (shared with *Plagiophthalmosuchus*, *Mystriosaurus*, *Platysuchus*, *Teleosaurus*, *Mycterosuchus*, *Aeolodon*, *Bathysuchus*, *Neosteneosaurus*, *Mac. buffetauti* and *Mac. mosae*); orbit is ellipsoid in shape (shared with *Plagiophthalmosuchus*, the Chinese teleosauroid, *Platysuchus*, *Aeolodon*, *Macrospondylus*, *Charitomenosuchus*, *Seldsienean*, *Deslongchampsina*, *Pr. heberti* and *Neosteneosaurus*).

**Remarks**—the mandible of the holotype disappeared, while remnants of the skull material were initially sent to BHN2 (and was considered the lectotype (presumably BHN2 R 59)). However, this museum was closed in 2003 and the current whereabouts of the material is unknown. In addition, Vignaud (1995) considered the remaining vertebrae of the holotype (location also unknown) as the paralectotype, with no formal explanation as to why. In 1892, M. Makinsky discovered the skull figured in Lepage et al. (2008) in the *Pictonia baylei* ammonite zone (lower Kimmeridgian) near Villerville (Calvados, France). Buffetaut & Makinsky (1984) described it as ‘*Steneosaurus*’ cf. *bouchardi*; currently the location of this skull, as with all holotype material, is not known (Y. Lepage, 2018, personal communication). Due to the close phylogenetic placement of this taxon to *Proexochokefalos heberti*, it is currently considered to be in the same genus.

*Steneosaurus* Geoffroy Saint-Hilaire, 1825

**Type species**—*Steneosaurus rostromajor* Geoffroy Saint-Hilaire, 1825. Type by subsequent designation (see Johnson, Young & Brusatte, 2020).

**Etymology**— ‘Narrow lizard.’ *Steneo* is from the Greek *sténos* (στενός) meaning narrowness (presumably referring to the elongated maxillae), and *saurus* is Latin meaning lizard.

**Diagnosis**—nomen dubium, undiagnostic.

*Steneosaurus rostromajor* Geoffroy Saint-Hilaire, 1825

(Fig. 20)

**Figure 20:**
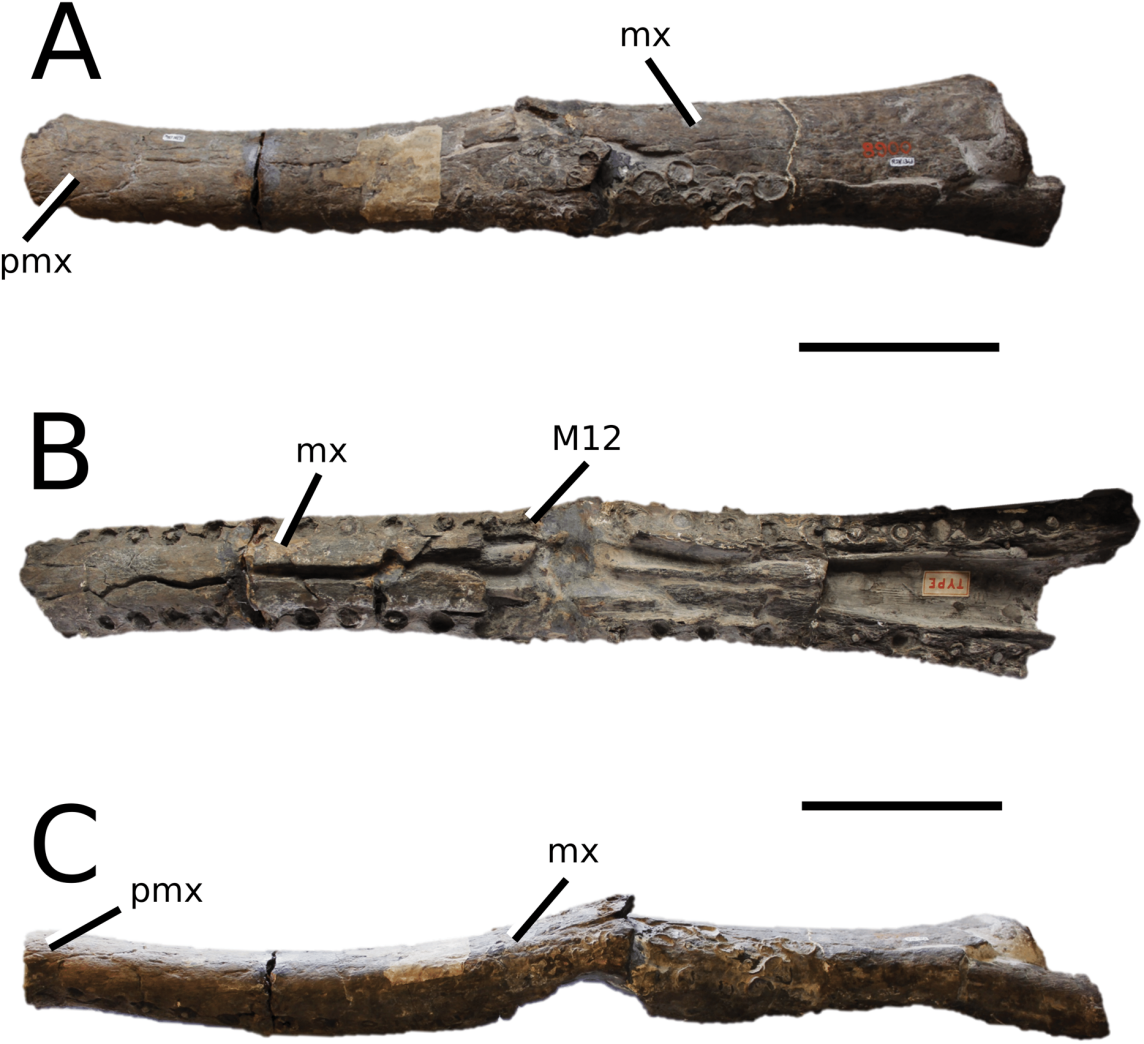
*Steneosaurus rostromajor*. *Steneosaurus rostromajor* (Geoffroy Saint-Hilaire, 1825), MNHN.RJN 134c-d, nomen dubium. Partial rostrum in (A) dorsal, (B) ventral and (C) left lateral views. Refer to abbreviations list. Scale bar: 10 cm.

**Lectotype**—MNHN.RJN 134, a partial rostrum. Designated by Johnson, Young & Brusatte (2020).

**Age**—lower Oxfordian, Late Jurassic (Bacheley (1778a, 1778b) and Cuvier (1808, 1812)).

**Locality**—Vaches Noires, Calvados, France.

**Stratigraphic horizon**—Marnes de Villiers Formation (hypothesized by Bacheley (1778a, 1778b) and Cuvier (1808, 1812)).

**Scoring sources**—the lectotype (MNHN.RJN 134c-d) was examined first-hand.

**Description**—maxillae ornamented with numerous, weakly- to strongly developed grooves; moderately interdigitating premaxilla-maxilla dorsal suture (shared with *Mystriosaurus*, *Proexochokefalos*, *Andrianavoay*, *Neosteneosaurus* and Machimosaurini); deep, pronounced reception pits throughout the entirety of the maxilla (shared with *Andrianavoay*, *Neosteneosaurus*, and Machimosaurini); at least 27 maxillary alveoli; mainly circular, well-spaced maxillary alveoli throughout the entirety of the rostrum; posterior maxillary alveoli slightly smaller than anterior maxillary alveoli (similar to *Yvridiosuchus*); well-developed, pronounced enamel ridges near the base of the tooth. See Johnson, Young & Brusatte (2020) for more detail.

**Remarks**—initially, the type species of the genus *Steneosaurus* (MNHN.RJN 134), *Steneosaurus rostromajor* Geoffroy Saint-Hilaire, 1825, was composed of a rostrum (MNHN.RJN 134c-d) and orbital region (MNHN.RJN 134a-b); however, the orbital section comes from a metriorhynchid. The validity of this taxon has been called into question due to its fragmentary nature (Eudes-Deslongchamps, 1867) and paraphyletic or polyphyletic nature of *Steneosaurus* in phylogenetic studies (Mueller-Töwe, 2006; Ősi et al., 2018; Foffa et al., 2019; Johnson, Young & Brusatte, 2019). Currently, only one taxon can hypothetically be referable to *S*. *rostromajor*, *Neosteneosaurus*; however, due to lack of autapomorphic features, uncertainty of teleosauroid ontogenetic and sexual dimorphic stages, a generic concept that has changed multiple times, and poor preservation, *S. rostromajor* is currently regarded as a nomen dubium (Johnson, Young & Brusatte, 2020).

*Andrianavoay* **gen. nov.**

**Type species**—*Steneosaurus baroni* Newton, 1893. Now referred to as *Andrianavoay baroni* (Newton, 1893), **comb. nov**. urn:lsid:zoobank.org:act:90C7838E-BE28-4615-BB85-BB04B67F1304

**Etymology**— ‘Noble crocodile’. *Andrian’* and *voay* are Malagasy meaning noble (usually referring to a prince) and crocodile, respectively.

**Diagnosis**—same as the only known species (monotypic genus).

*Andrianavoay baroni* (Newton, 1893) **comb. nov.**

(Fig. 21)

**Figure 21:**
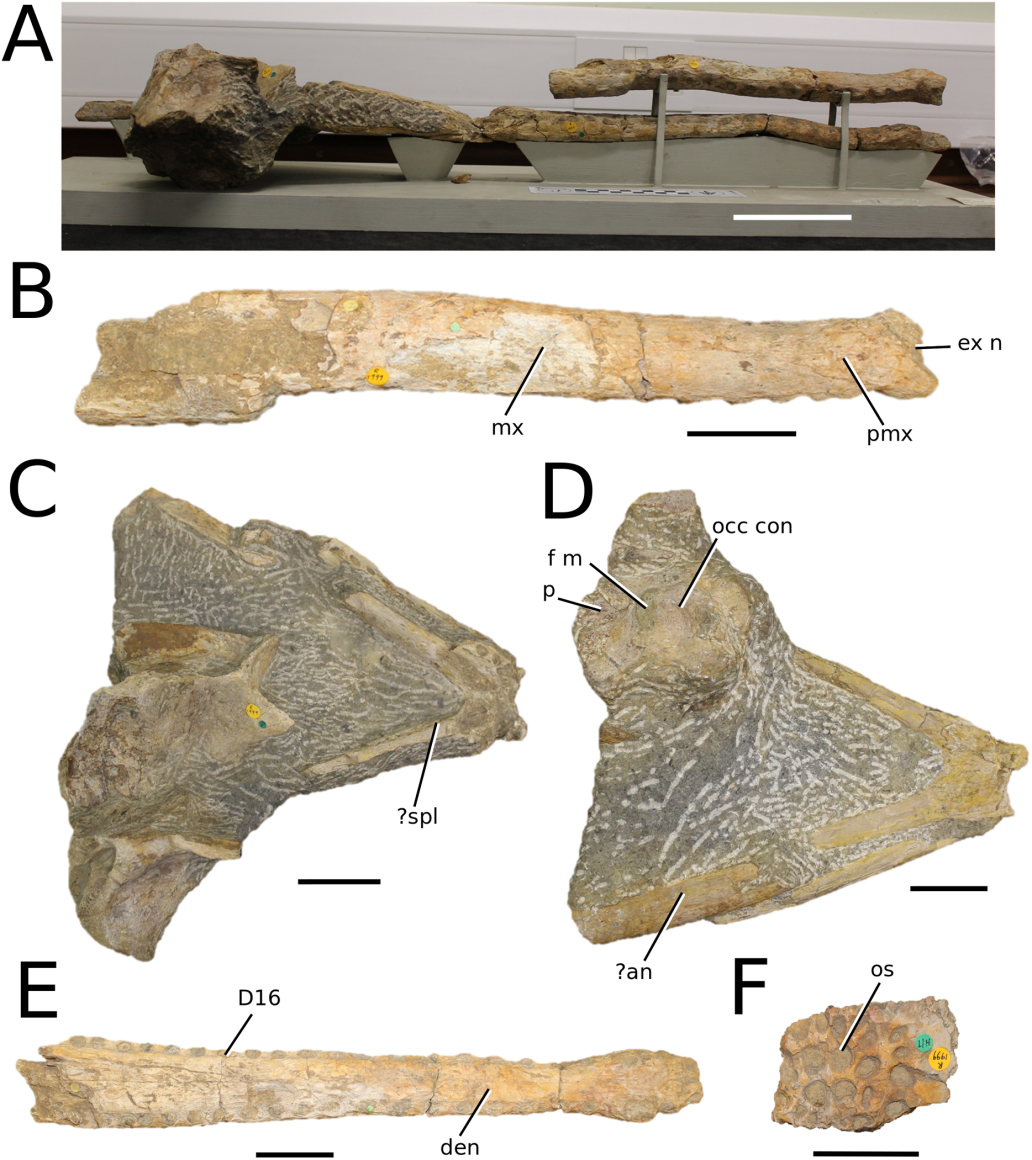
*Andrianavoay baroni*. *Andrianavoay baroni* (Newton, 1893), **comb. nov.**, NHMUK PV R 1999, holotype. Photograph of the partial skull and mandible in (A) right lateral view, as well as (B) partial rostrum in dorsal view; posterior skull in (C) dorsal and (D) ventral views; (E) partial mandible in dorsal view; and (F) fragment of osteoderm in dorsal view. Refer to abbreviations list. Scale bars: 10 cm (A), 5 cm (B–E) and 3 cm (F).

**Holotype**—NHMUK PV R 1999, a partial skull and mandible with one associated osteoderm.

**Age**—Lower Oolite, Bathonian, Middle Jurassic, based on association with *Mytilus*, *Modiola*, *Perna* and *Trochactmonina* shells (Newton, 1893).

**Locality**—Andranosamonta, northwestern Madagascar.

**Stratigraphic horizon**—Unknown.

**Scoring sources**—the holotype (NHMUK PV R 1999) was examined first-hand.

**Autapomorphic characters of *A. baroni***—sparse, small, deep subcircular foramina on the posterior and lateral margins of the external nares; anteroposteriorly thin posterior-most parietal.

**Emended diagnosis**—maxilla ornamented with numerous, shallow to deep grooves; premaxilla anterior and anterolateral margins are not sub-vertical (shared with *Plagiophthalmosuchus*, *Macrospondylus*, *Charitomenosuchus*, *Deslongchampsina*, *Proexochokefalos*, *Neosteneosaurus* and Machimosaurini); moderately interdigitating premaxilla-maxilla dorsal suture (shared with *Mystriosaurus*, *Proexochokefalos*, *Neosteneosaurus*, S. *rostromajor* and Machimosaurini); dorsoventrally deep posterior premaxilla (shared with *Proexochokefalos*); dorsoventrally tall supraoccipital (shared with *Plagiophthalmosuchus*, *Clovesuurdameredeor* and *Lemmysuchus*); deep, pronounced reception pits throughout the entirety of the maxilla (shared with S. *rostromajor*, *Neosteneosaurus* and Machimosaurini); osteoderm fragment with large, circular pits that are well separated from one another.

*Neosteneosaurus* **gen. nov.**

**Type species**—*Steneosaurus edwardsi* Eudes-Deslongchamps, 1868a. Now referred to as *Neosteneosaurus edwardsi* (Eudes-Deslongchamps, 1868a), **comb. nov**. urn:lsid:zoobank.org:act:09ADDEA4-AB2B-40A4-AAFF-19819898532F

**Etymology**— ‘New *Steneosaurus*’. ‘*Neo-*’ is from the Greek *neos* (νέος) meaning ‘new’. Refers to the genus this species previously belonged to, *Steneosaurus*.

**Diagnosis**—same as the only known species (monotypic genus).

*Neosteneosaurus edwardsi* (Eudes-Deslongchamps, 1868a) **comb. nov.**

(Fig. 22)

**Figure 22:**
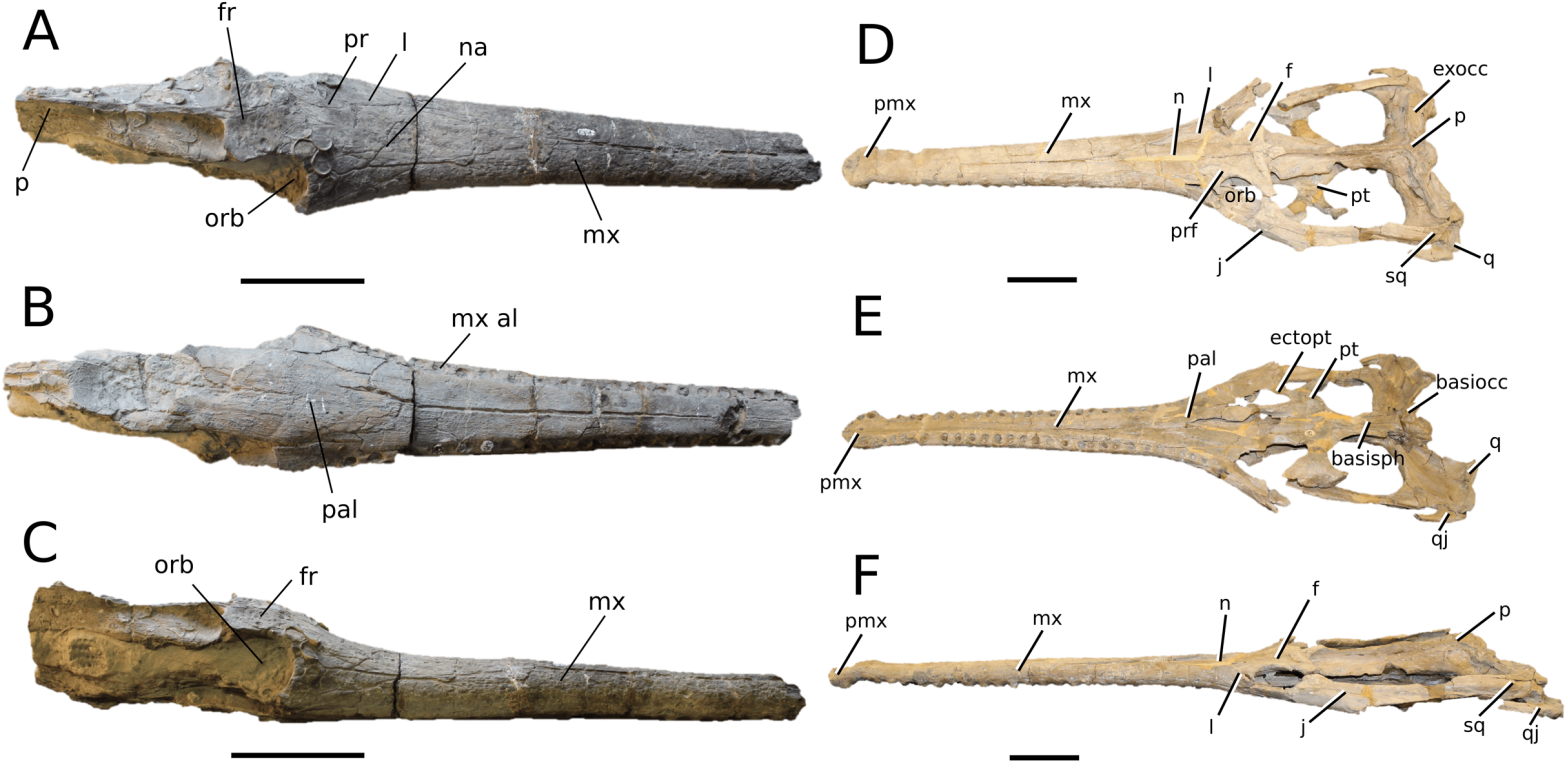
*Neosteneosaurus edwardsi*. *Neosteneosaurus edwardsi* (Eudes-Deslongchamps, 1868a), **comb. nov.** (A–C) MNHN.RJN 118, holotype and (D–F) NHMUK PV R 2865, referred specimen. Partial skull in (A) dorsal, (B) ventral (palatal) and (C) right lateral views. Refer to abbreviations list. Scale bars: 10 cm.

**Holotype**—While Eugène Eudes-Deslongchamps (1867) described and figured MNHN.RJN 118, he did not formally designate it as the holotype, and included other specimens (syntypes) in his original description (Brignon, 2018b).

**Lectotype**—MNHN.RJN 118, a partial skull (see Brignon, 2018b).

**Referred material**—GPIT-RE-7286 (complete skeleton); NHMUK PV R 2075 (partial skull, mandible and associated postcrania); NHMUK PV R 2076 (partial mandible and femora, ilia, tibia, ulna, dorsal and sacral osteoderms); NHMUK PV R 2865 (complete skull, assorted vertebrae and isolated teeth); NHMUK PV R 3701 (nearly complete skull and mandible, and partial skeleton); NHMUK PV R 3898 (femur, ilium and ischium); NRM-PZ R.144 (a partial sacral vertebra); NRM-PZ R.2053 (tibia); NRM-PZ R.2074 (femur); OUMNH J.29815 (partial skull); PETMG R175 (complete skeleton); PETMG R178 (nearly complete skeleton); SMF R 123 (complete skull and nearly complete mandible).

**Age**—Middle Callovian, Middle Jurassic.

**Locality**—Peterborough, UK.

**Stratigraphic horizon**—Peterborough Member, Oxford Clay Formation, Ancholme Group.

**Scoring sources**—the holotype (MNHN.RJN 118), as well as all additional referred specimens, were examined first-hand.

**Autapomorphic characters of *N. edwardsi***—posterior (distal) teeth with sub-pointed apices (are not blunt and rounded but significantly less pointed than in anterior [mesial] and middle teeth); tuberculum and articular facet of the dorsal rib positioned on the lateromedial edge.

**Emended diagnosis**—mesorostrine snout; tooth row and occipital condyle aligned, and quadrate condyle at a lower level (shared with the Chinese teleosauroid, *Charitomenosuchus*, *Proexochokefalos* and Machimosaurini); frontal ornamentation restricted to centre (shared with *Sericodon*, *Aeolodon*, *Charitomenosuchus*, *Seldsienean*, *Deslongchampsina*, *Proexochokefalos* and Machimosaurini); external nares oriented dorsally (shared with *Plagiophthalmosuchus*, *Macrospondylus*, *Charitomenosuchus*, *Deslongchampsina*, *Proexochokefalos*, and Machimosaurini); premaxilla anterior and anterolateral margins are not sub-vertical (shared with *Plagiophthalmosuchus*, *Macrospondylus*, *Andrianavoay*, *Charitomenosuchus*, *Deslongchampsina*, *Proexochokefalos* and Machimosaurini); moderately interdigitating premaxilla-maxilla suture, appearing subcircular in shape (shared with *Mystriosaurus*, *Andrianavoay*, *S*. *rostromajor*, *Lemmysuchus* and *Machimosaurus*); absence of antorbital fenestrae (shared with *Proexochokefalos* and Machimosaurini excluding *Yvridiosuchus*); supratemporal fenestrae length is twice as long as wide (shared with *Proexochokefalos*, and somewhat similar to Machimosaurini); the anterior process of the jugal is slender, elongated and extends anteriorly (shared with *Clovesuurdameredeor*, *Proexochokefalos* and Machimosaurini); orbit is longitudinal ellipsoid in shape (shared with *Plagiophthalmosuchus*, the Chinese teleosauroid, *Platysuchus*, *Aeolodon*, *Macrospondylus*, *Charitomenosuchus*, *Seldsienean*, *Proexochokefalos* and *Deslongchampsina*); frontal width broader than orbital width (shared with *Plagiophthalmosuchus*, *Mystriosaurus*, *Platysuchus*, *Teleosaurus*, *Mycterosuchus*, *Bathysuchus*, *Aeolodon*, *Pr*. cf. *bouchardi*, *Mac. buffetauti* and *Mac. mosae*); squamosal projects further posteriorly than occipital condyle (shared with the Chinese teleosauroid and Machimosaurini); shallow Meckelian groove (shared with *Proexochokefalos* and Machimosaurini); mandibular symphysis between 30 to 45% of the mandibular length; (shared with Machimosaurini); deep, pronounced reception pits throughout the entirety of the maxilla (shared with *Andrianavoay*, *Neosteneosaurus*, and Machimosaurini); maxillary teeth not procumbent (shared with *Proexochokefalos* and Machimosaurini); large, robust, weakly-compressed teeth with a pointed apex and high relief enamel ridges (similar to *Deslongchampsina*); postacetabular iliac process is fan-shaped (shared with *Charitomenosuchus*, *Lemmysuchus* and *Mac. mosae*); tibia approximately 40-50% shorter than the femur (shared with *Mycterosuchus*, *Charitomenosuchus*, *Lemmysuchus* and *Mac. mosae*); medial femoral condyle larger than lateral femoral condyle (shared with *Mycterosuchus*, *Charitomenosuchus* and *Machimosaurus*); elongated and pronounced keel across the entirety of the sacral dorsal osteoderms (shared with *Lemmysuchus*).

TRIBE Machimosaurini (Jouve et al., 2016)

*Yvridiosuchus* Johnson, Young & Brusatte, 2019

**Type species**—*Steneosaurus boutilieri* Eudes-Deslongchamps, 1868b. Now referred to as *Yvridiosuchus boutilieri* (Eudes-Deslongchamps, 1868b), Johnson, Young & Brusatte, 2019.

**Etymology**— ‘Hybrid crocodile’. *Yvrídio* (υβρίδιο) is Ancient Greek for ‘hybrid’ (refers to a unique combination of non-machimosaurin and machimosaurin teleosauroid symplesiomorphies observed in this genus), and *suchus* is the Latinized form of the Greek *soukhos* (σοῦχος), meaning crocodile.

**Diagnosis**—same as the only known species (monotypic genus).

*Yvridiosuchus boutilieri* (Eudes-Deslongchamps, 1868b) Johnson, Young & Brusatte, 2019

(Fig. 23)

**Figure 23:**
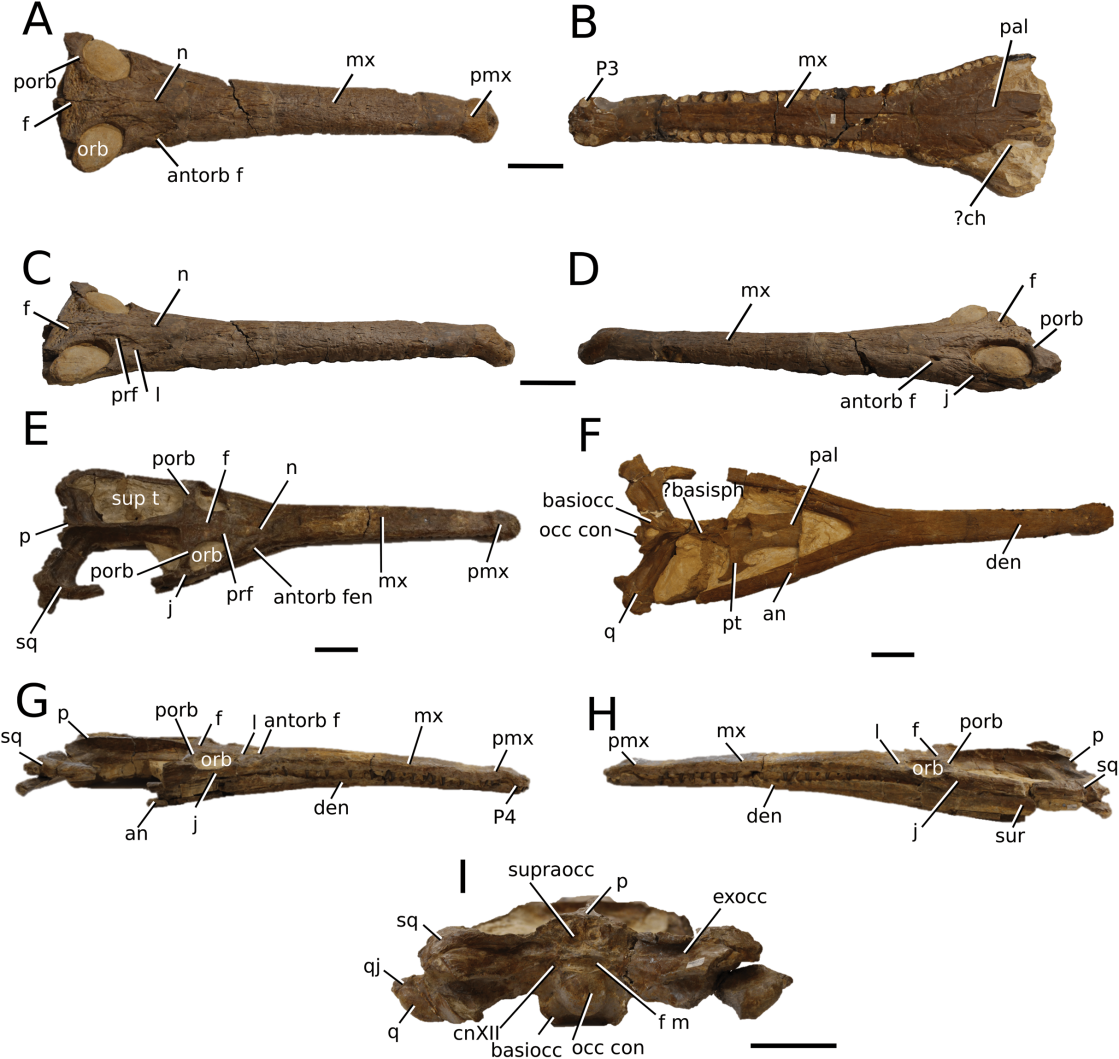
*Yvridiosuchus boutilieri*. *Yvridiosuchus boutilieri* (Eudes-Deslongchamps, 1868c) Johnson, Young & Brusatte, 2019. (A–D) OUMNH J.1401, holotype and (E–I) OUMNH J.29850, referred specimen. Skull in (A and E) dorsal, (B and F) ventral (palatal), (C and G) right lateral, (D and H) left lateral and (I) occipital views. Refer to abbreviations list. Scale bars: 5 cm.

**Holotype**—A skull fragment, figured by Eudes-Deslongchamps (1867) and presumed lost or destroyed (Vignaud, 1995; Johnson, Young & Brusatte, 2019).

**Neotype**—OUMNH J.1401, a partial skull. Neotype designation by Johnson, Young & Brusatte (2019).

**Referred material**—OUMNH J.29850 (nearly complete skull and mandible); OUMNH J.1403 (nearly complete skull); OUMNH J.1404 (partial mandible); OUMNH J.1417 (partial mandible) (see Johnson, Young & Brusatte, 2019).

**Age**—Bathonian, Middle Jurassic.

**Localities**—Calvados, France; Enslow Bridge, Oxfordshire, UK.

**Stratigraphic horizons**— ‘*Sommet de la Grande Oolithe*, France; Great Oolite Group, UK.

**Scoring sources**—the neotype (OUMNH J.1401), as well as all referred specimens mentioned above, were studied first-hand.

**Autapomorphic characters of *Y. boutilieri***—heavily ornamented lacrimal, appearing perforated in lateral view; extreme elongation of the anterior jugal, so that it participates in the posterior margin of the antorbital fenestra; orbit subcircular in shape; anterior process of palatine U-shaped; width of retroarticular process is narrower than the glenoid fossa. See Johnson, Young & Brusatte (2019) for more detail.

**Emended diagnosis**—mesorostrine skull; skull ornamented with numerous conspicuous pits and grooves (differs from that seen in *Mycterosuchus* and *Mystriosaurus*); large and numerous neurovascular foramina on the premaxillae, maxillae and dentaries (shared with *Mystriosaurus* and Machimosaurini); external nares oriented dorsally (shared with *Plagiophthalmosuchus*, *Macrospondylus*, *Charitomenosuchus*, *Proexochokefalos*, *Deslongchampsina*, *Neosteneosaurus* and other members of Machimosaurini); premaxilla anterior and anterolateral margins are not sub-vertical (shared with *Plagiophthalmosuchus*, *Macrospondylus*, *Andrianavoay*, *Charitomenosuchus*, *Deslongchampsina*, *Proexochokefalos*, *Neosteneosaurus* and other members of Machimosaurini); presence of small, deep antorbital fenestrae; frontal width subequal with orbital width (shared with the Chinese teleosauroid, *Mycterosuchus*, *Proexochokefalos*, *Deslongchampsina*, *Mac. hugii*, and *Mac. rex*); squamosal projects further posteriorly than occipital condyle (shared with the Chinese teleosauroid, *Neosteneosaurus* and other members of Machimosaurini); shallow Meckelian groove (shared with *Proexochokefalos*, *Neosteneosaurus* and other members of Machimosaurini); sharp dorsoposterior curvature of the posterior mandibular rami (shared with *Proexochokefalos* and *Lemmysuchus*); teeth large and conical with blunt apices (shared with other members of Machimosaurini); teeth not mediolaterally compressed (shared with *Bathysuchus* and other members of Machimosaurini); carinae heterogeneous with faint denticles (shared with other members of Machimosaurini); teeth with anastomosing pattern on the apical surface (shared with other members of Machimosaurini); maxillary teeth not procumbent (shared with *Proexochokefalos*, *Neosteneosaurus* and other members of Machimosaurini).

**Remarks**—*Yvridiosuchus* has a long and complicated taxonomic history, including an invalid species name (*Crocodilus oxoniensis*; following ICZN Code rules), and OUMNH J.1401 (the designated neotype) considered by Eudes-Deslongchamps (1867) as “*appartenant à la même espèce*” (“belonging to the same species”) to the previously destroyed French holotype (Johnson, Young & Brusatte, 2019). In addition, *Teleosaurus* (‘*Steneosaurus*’) *brevidens* Phillips, 1871, and ‘*Steneosaurus*’ *meretrix* Phizackerley, 1951 (the holotype of *T. brevidens*), are subjective junior synonyms of *Yvridiosuchus* (see Johnson, Young & Brusatte, 2019 for more information).

*Lemmysuchus* Johnson et al., 2017

**Type species**—*Steneosaurus obtusidens* Andrews, 1909. Now referred to as *Lemmysuchus obtusidens* (Andrews, 1909) Johnson et al., 2017.

**Etymology**— ‘Lemmy’s crocodile’. *Lemmy* refers to Ian Fraser ‘Lemmy’ Kilmister, the deceased founder, lead singer and bassist of the band Motörhead, and *suchus* is the Latinized form of the Greek *soukhos* (σοῦχος), meaning crocodile.

**Diagnosis**—same as the only known species (monotypic genus).

*Lemmysuchus obtusidens* (Andrews, 1909) Johnson et al., 2017

(Fig. 24)

**Figure 24:**
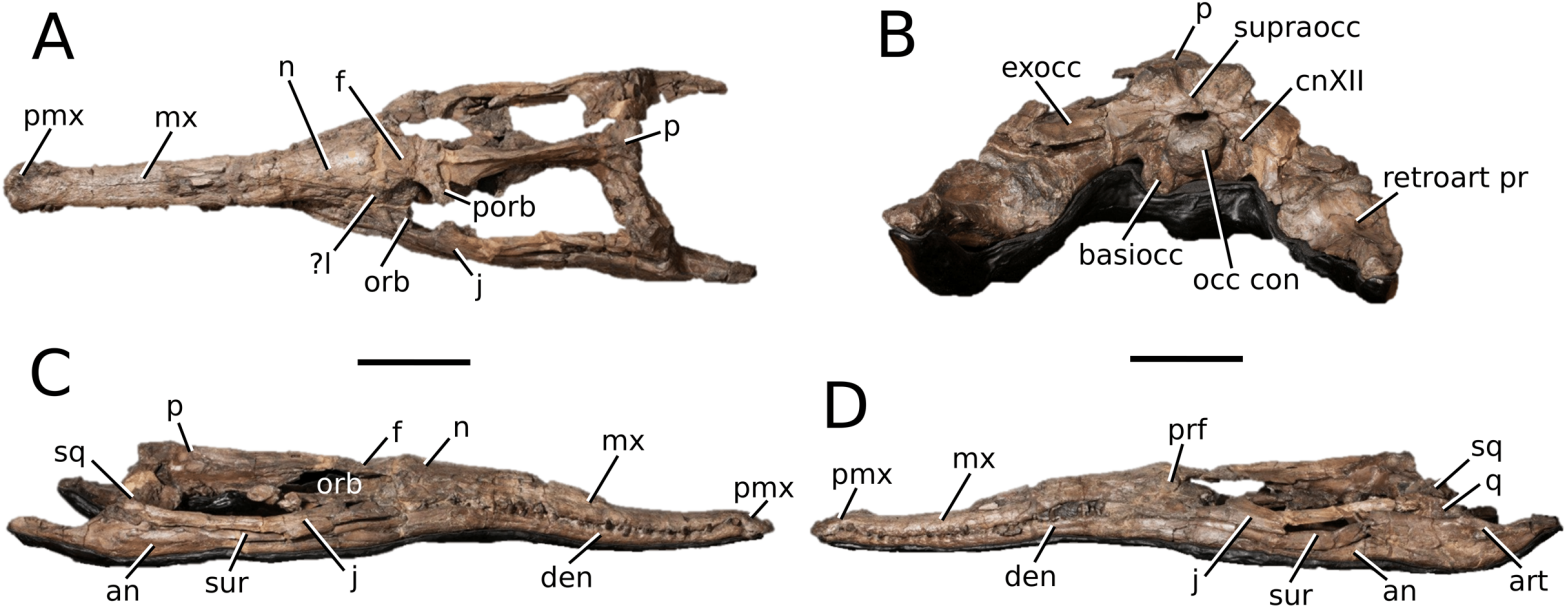
*Lemmysuchus obtusidens*. *Lemmysuchus obtusidens* (Andrews, 1909) Johnson et al., 2017, NHMUK PV R 3168, holotype. Skull in (A) dorsal, (B) occipital, (C) right lateral and (D) left lateral views. Refer to abbreviations list. Scale bars: 20 cm.

**Holotype**—NHMUK PV R 3168, a nearly complete skeleton including the skull, mandible, vertebrae, hindlimbs, and multiple osteoderms.

**Referred material**—LPP.M.21 (a nearly complete skull and mandible); NOTNH FS3361 (a partial rostrum); PETMG R39 (a rostral-orbital section).

**Age**—Middle Callovian, Middle Jurassic.

**Locality**—Peterborough, UK.

**Stratigraphic horizon**—Peterborough Member, Oxford Clay Formation, Ancholme Group.

**Scoring sources**—the holotype (NHMUK PV R 3168) and all referred specimens mentioned above were studied first-hand.

**Autapomorphic characters of *L. obtusidens***—the rostrum external surface is strongly convex, in particular the nasals; partial or complete fusion of the internasal suture; nasal midline cavity poorly developed; eight cervical vertebrae; dorsoventrally curved cervical ribs; anterior process of ilium is anteroposteriorly shortened; acetabulum is shallow and poorly developed; shallow supraacetabular crest on the ilium; anterior ischial process reduced; dorsal osteoderms with small-to-large, irregularly shaped pits that radiate from the centre of the keel and are arranged in a starburst pattern (to a certain extent similar to *Mac. mosae*). See Johnson et al. (2017) for more detail.

**Emended diagnosis**—mesorostrine skull; external nares oriented dorsally (shared with *Plagiophthalmosuchus*, *Macrospondylus*, *Deslongchampsina*, *Proexochokefalos*, *Neosteneosaurus* and other members of Machimosaurini); two parallel lines of large, circular neurovascular foramina on the premaxillae and maxillae, and a clustering of foramina on the lateral surface of the premaxillae (shared with other members of Machimosaurini); premaxilla anterior and anterolateral margins are not sub-vertical (shared with *Plagiophthalmosuchus*, *Macrospondylus*, *Andrianavoay*, *Charitomenosuchus*, *Deslongchampsina*, *Proexochokefalos*, *Neosteneosaurus* and other members of Machimosaurini); moderately interdigitating premaxilla-maxilla suture, appearing subcircular in shape (shared with *Mystriosaurus*, *Andrianavoay*, *Neosteneosaurus*, *S*. *rostromajor*, and *Machimosaurus*); absence of antorbital fenestrae (shared with *Proexochokefalos*, *Neosteneosaurus* and other members of Machimosaurini excluding *Yvridiosuchus*); parallelogram-shaped supratemporal fenestrae (shared with other members of Machimosaurini); the anterior process of the jugal is slender, elongated and extends anteriorly (shared with *Clovesuurdameredeor*, *Proexochokefalos*, *Neosteneosaurus* and other members of Machimosaurini); squamosal project posteriorly to occipital condyle (shared with *Plagiophthalmosuchus*, the Chinese teleosauroid, *Neosteneosaurus* and *Yvridiosuchus*); supraoccipital dorsoventrally tall (shared with *Plagiophthalmosuchus*, *Clovesuurdameredeor* and *Andrianavoay*); shallow Meckelian groove (shared with *Proexochokefalos*, *Neosteneosaurus* and other members of Machimosaurini); retroarticular process subequal to glenoid fossa width (shared with *Aeolodon* and *Mac. buffetauti*); teeth large and conical with blunt apices (shared with other members of Machimosaurini); teeth not mediolaterally compressed (shared with *Bathysuchus* and other members of Machimosaurini); carinae heterogeneous with faint denticles (shared with other members of Machimosaurini); teeth with anastomosing pattern on the apical surface (shared with other members of Machimosaurini); axis lacks diapophyses (shared with *Macrospondylus*); three sacral vertebrae (shared with *Machimosaurus*); dorsal ribs with pronounced tuberculum (shared with *Mycterosuchus*, *Neosteneosaurus* and *Machimosaurus*); postacetabular iliac process is fan-shaped (shared with *Charitomenosuchus*, *Neosteneosaurus* and *Mac. mosae*); posteroventral margin of ischial plate sub-squared (shared with *Mac. mosae*); tibia approximately 40–50% shorter than the femur (shared with *Mycterosuchus*, *Charitomenosuchus*, *Neosteneosaurus* and *Mac. mosae*); tibial tuberosity angled ventrally (shared with *Mac. mosae*); elongate and pronounced keel on sacral osteoderms (shared with *Neosteneosaurus*).

**Remarks**—the exact location of LPP.M.21, which comes from France, is currently unknown.

GENUS *Machimosaurus* (Von Meyer, 1837) emend. Von Meyer, 1838

**Type species**—*Machimosaurus hugii* Von Meyer, 1837 emend. Von Meyer, 1838

**Referred species**—*Machimosaurus buffetauti* Young et al., 2015b; *Machimosaurus mosae* Sauvage & Liénard, 1879; *Machimosaurus rex* Fanti et al., 2016.

**Etymology**— ‘Pugnacious lizard’. *Machimo* is derived from the Greek *machimoi* (μάχιμoι), meaning pugnacious (having a combative nature, presumably referring to the robust dentition), and *saurus* is the Latinized version of *sauros* (σαυρoς), which is Ancient Greek for lizard.

**Age**—middle Oxfordian to upper Hauterivian/lower Barremian.

**Geographical range**—Africa (Ethiopia and Tunisia) and Europe (England, France, Germany, Portugal, Spain and Switzerland).

**Generic diagnosis**—rostrum wider than high; three alveoli per premaxilla; first premaxillary alveoli strongly oriented anteroventrally; 18–22 alveoli per maxilla; 19–25 alveoli per dentary; maximum supratemporal length is greater than 27% relative to maximum basicranial length; extreme elongation of the supratemporal fenestrae, with the anteroposterior length twice the mediolateral length; medial quadrate hemicondyle considerably smaller than the lateral quadrate hemicondyle; presence of carinae on teeth variable; tall axis neural spine terminating on a plane dorsal to the pre- and postzygapophyses in lateral view; axis neural spine posteriorly expanded in lateral view.

*Machimosaurus buffetauti* Young et al., 2015b

(Fig. 25)

**Figure 25:**
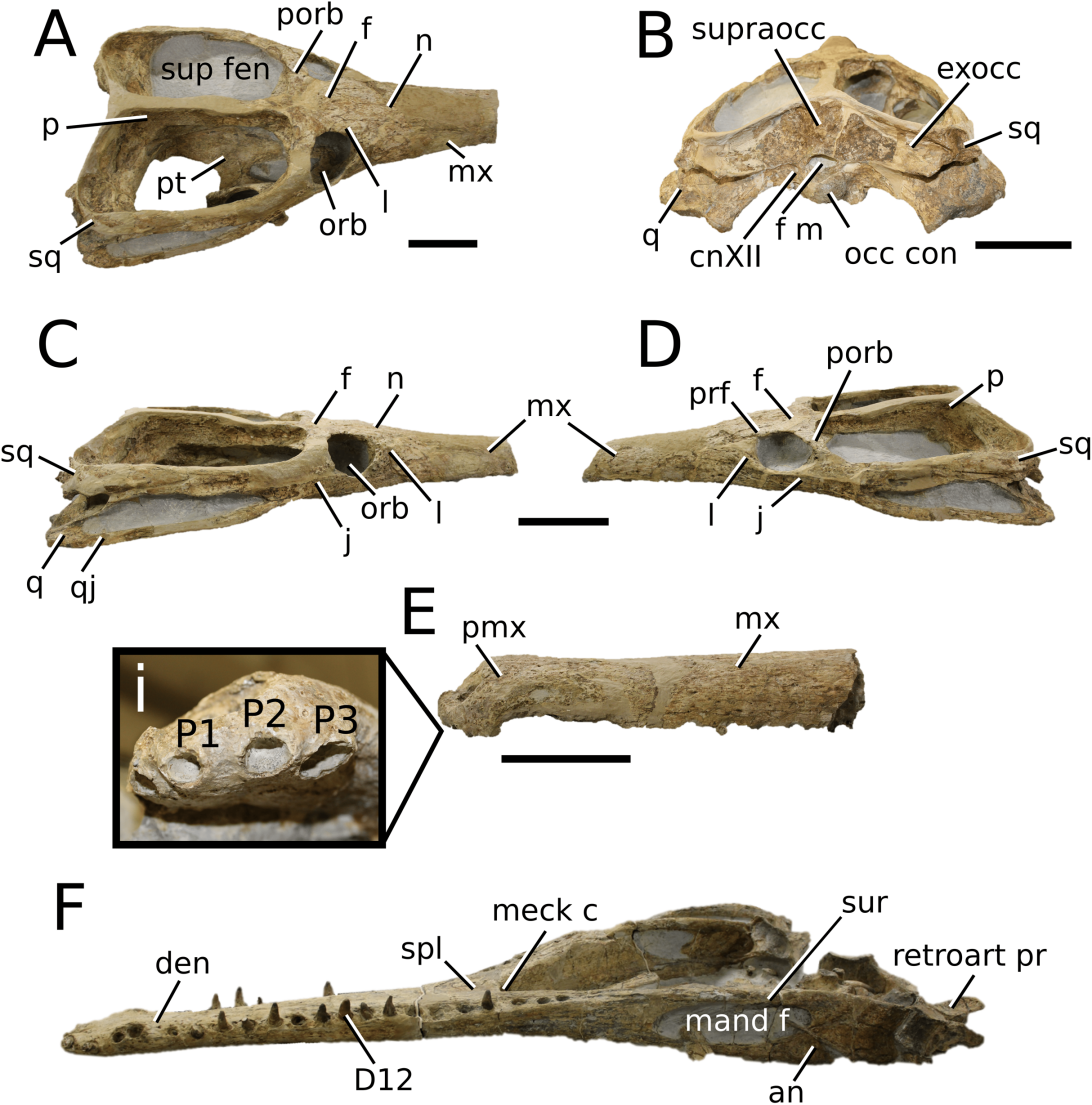
*Machimosaurus buffetauti*. *Machimosaurus buffetauti* Young et al., 2015b, SMNS 91415, holotype. Skull in (A) dorsal, (B) occipital, (C) right lateral and (D) left lateral views. Rostrum in (E) left lateral view, with a close-up of (i) the premaxillary alveoli. (F) Mandible in left lateral view. Refer to abbreviations list. Scale bars: 10 cm.

**Holotype**—SMNS 91415, a complete skull and mandible (as well as in situ teeth) with associated partial postcranial skeleton including cervical and dorsal vertebrae, one coracoid and multiple osteoderms.

**Referred material**—DFMMh FV 330 (isolated tooth crown); DFMMh FV 541 (isolated tooth crown); MPV V1600.Bo (anterior region of rostrum and mandible); MPV V1601.Bo (partial rostrum).

**Age**—*Ataxioceras hypselocyclum* Sub-Mediterranean ammonite Zone (=Weißer Jura gamma 2), Lower Kimmeridgian, Upper Jurassic.

**Localities**—Am Hörnle Quarry, Neuffen, Baden-Württemberg, Germany; lower Saxony, Germany; Cricqueboeuf, Normandy, Northern France

**Stratigraphic horizons**—Lacunosamergel Formation; Langenberg Formation; Calcaires Coquilliers Formation.

**Scoring sources**—the holotype (SMNS 91415) was examined first-hand, and additional information was gleaned from Young et al. (2014, 2015b).

**Autapomorphic characters of *Mac. buffetauti***—anterolateral frontal projections between nasals and prefrontals; squamosal approximately level with occipital condyle; retroarticular process is slightly longer than wide; low post-symphyseal tooth count of the dentary; dorsal margin of the axis neural arch is strongly concave in lateral view; tuberculum and articular facet of dorsal ribs slightly situated on the medial edge; elongated coracoid glenoid process that extends considerably from the proximal coracoid, and sub-isosceles triangle-shaped in lateral view; anterior margin of the coracoid postglenoid process is slightly concave and terminates approximately in the same frontal plane as the glenoid; posterior margin of the coracoid postglenoid process is strongly concave and terminates approximately in the same frontal plane as the posterior end of the glenoid process; dorsal osteoderms with generally small, irregularly shaped pits arranged in a random pattern, with a shallow keel.

**Emended diagnosis**—brevirostrine skull; rostrum wider than high; two parallel lines of large, circular neurovascular foramina on the premaxillae and maxillae, and a clustering of foramina on the lateral surface of the premaxillae (shared with *Mystriosaurus* and members of Machimosaurini); dentary neurovascular foramina form a relatively straight line (shared with *Mac. mosae*); external nares oriented dorsally (shared with *Plagiophthalmosuchus*, *Macrospondylus*, *Deslongchampsina*, *Proexochokefalos*, *Neosteneosaurus* and other members of Machimosaurini); premaxilla anterior and anterolateral margins are not sub-vertical (shared with *Plagiophthalmosuchus*, *Macrospondylus*, *Andrianavoay*, *Charitomenosuchus*, *Deslongchampsina*, *Proexochokefalos*, *Neosteneosaurus* and other members of Machimosaurini); premaxilla less than 25% of rostral length (shared with *Mystriosaurus*, the Chinese teleosauroid and *Mac. mosae*); absence of antorbital fenestrae (shared with *Proexochokefalos*, *Neosteneosaurus*, *Lemmysuchus* and other members of *Machimosaurus*); parallelogram-shaped supratemporal fenestrae (shared with other members of Machimosaurini); frontal width broader than orbital width (shared with *Plagiophthalmosuchus*, *Mystriosaurus*, *Platysuchus*, *Teleosaurus*, *Mycterosuchus*, *Bathysuchus*, *Aeolodon*, *Pr*. cf. *bouchardi*, *Neosteneosaurus* and *Mac. mosae*); circular orbits (shared with *Mystriosaurus*, *Indosinosuchus*, *Teleosaurus*, *Mycterosuchus*, *Clovesuurdameredeor*, *Lemmysuchus* and other members of *Machimosaurus*); the anterior process of the jugal is slender, elongated and extends anteriorly (shared with *Clovesuurdameredeor*, *Proexochokefalos*, *Neosteneosaurus* and Machimosaurini); quadrates with a single large, circular depression on the dorsal surface close to the hemicondyles; shallow Meckelian groove (shared with *Proexochokefalos*, *Neosteneosaurus* and other members of Machimosaurini); retroarticular width is subequal to the glenoid fossa (shared with *Aeolodon* and *Lemmysuchus*); 21–28 maxillary alveolar pairs; deep, pronounced reception pits throughout the entirety of the maxilla (shared with *Andrianavoay*, S. *rostromajor*, *Neosteneosaurus* and other members of Machimosaurini); teeth large and conical with blunt apices (shared with other members of Machimosaurini); teeth not mediolaterally compressed (shared with *Bathysuchus* and other members of Machimosaurini); carinae heterogeneous with faint denticles (shared with other members of Machimosaurini); presence of keeled carinae variable (shared with *Mac. hugii* and *Mac. rex*); teeth with anastomosing pattern on the apical surface (shared with other members of Machimosaurini).

**Remarks**—the correct nominal authority is found in the short taxonomic note in Young et al., 2015b, not Young et al., 2014 (where the new taxon was described).

*Machimosaurus mosae* Sauvage & Liénard, 1879

(Fig. 26)

**Figure 26:**
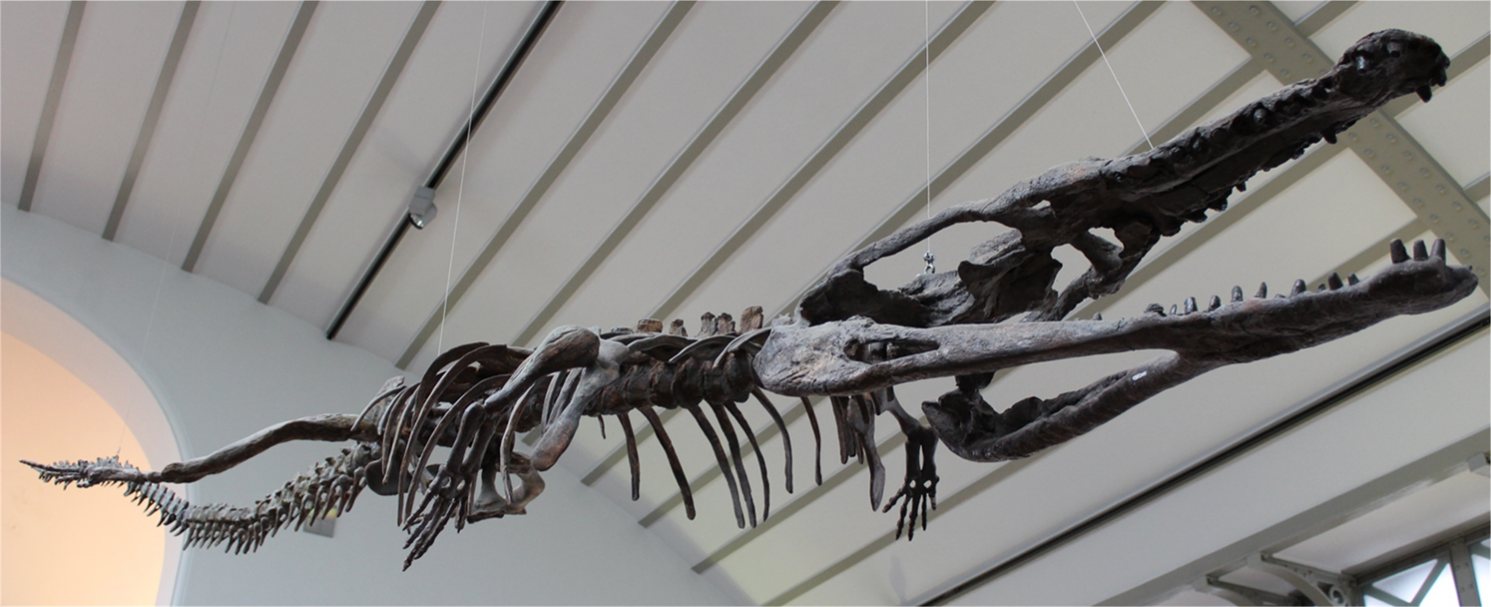
*Machimosaurus mosae*. *Machimosaurus mosae* Sauvage & Liénard, 1879, IRSNB cast. Not to scale.

**Holotype**—A skull, destroyed during the First World War. Location and horizon unknown.

**Neotype**—A partially complete skeleton, labelled as MHNB 1100. Current location unknown.

**Referred material**—IRSNB (cast of neotype with reconstructed elements added, representing a complete skeleton); Hua (1999); Young et al. (2014).

**Age**—Either the *Aulacostephanus autissiodorensis* Sub-Boreal ammonite Zone, uppermost Kimmeridgian, or the *Gravesia gigas*/*Pectinaties elegans* Sub-Boreal ammonite Zone, lowermost Tithonian; Late Jurassic (neotype locality).

**Neotype locality**—Beach near Ambleteuse, Boulonnais, Département du Pas-de-Calais, Nord Pas-de-Calais, France.

**Neotype stratigraphic horizon**—Argiles de Châtillon Formation.

**Scoring sources**—Young et al. (2014). Additional information was gleaned from examining the large cast of *Mac. mosae* in the IRSNB exhibit.

**Autapomorphic characters of *Mac. mosae***—anterior palatal margin terminates at approximately the 11–14th maxillary alveoli; approximately 17–18 alveoli per maxilla; approximately 19–20 alveoli per dentary; coracoid glenoid process very short; anterior edge of the scapula is strongly concave compared to the posterior edge.

**Emended diagnosis**—brevirostrine skull; conspicuous grooved-ridged ornamentation of maxilla (shared with *Mac. hugii* and *Mac. rex*); two parallel lines of large, circular neurovascular foramina on the premaxillae and maxillae, and a clustering of foramina on the lateral surface of the premaxillae (shared with *Mystriosaurus* and members of Machimosaurini); dentary neurovascular foramina form a relatively straight line (shared with *Mac. buffetauti*); external nares oriented dorsally (shared with *Plagiophthalmosuchus*, *Macrospondylus*, *Deslongchampsina*, *Proexochokefalos*, *Neosteneosaurus* and other members of Machimosaurini); premaxilla anterior and anterolateral margins are not subvertical (shared with *Plagiophthalmosuchus*, *Macrospondylus*, *Andrianavoay*, *Charitomenosuchus*, *Deslongchampsina*, *Proexochokefalos*, *Neosteneosaurus* and other members of Machimosaurini); premaxilla less than 25% of rostral length (shared with *Mystriosaurus*, the Chinese teleosauroid and *Mac. buffetauti*); absence of antorbital fenestrae (shared with *Proexochokefalos*, *Neosteneosaurus*, *Lemmysuchus* and other members of *Machimosaurus*); parallelogram-shaped supratemporal fenestrae (shared with other members of Machimosaurini); frontal width broader than orbital width (shared with *Plagiophthalmosuchus*, *Mystriosaurus*, *Platysuchus*, *Teleosaurus*, *Mycterosuchus*, *Bathysuchus*, *Aeolodon*, *Pr*. cf. *bouchardi*, *Neosteneosaurus* and *Mac. buffetauti*); circular orbits (shared with *Mystriosaurus*, *Indosinosuchus*, *Teleosaurus*, *Mycterosuchus*, *Clovesuurdameredeor*, *Lemmysuchus* and other members of *Machimosaurus*); shallow Meckelian groove (shared with *Proexochokefalos*, *Neosteneosaurus* and other members of Machimosaurini); deep, pronounced reception pits throughout the entirety of the maxilla (shared with *Andrianavoay*, *S*. *rostromajor*, *Neosteneosaurus* and other members of Machimosaurini); teeth large and conical with blunt apices (shared with other members of Machimosaurini); teeth not mediolaterally compressed (shared with *Bathysuchus* and other members of Machimosaurini); carinae heterogeneous with faint denticles (shared with other members of Machimosaurini); teeth with anastomosing pattern on the apical surface (shared with other members of Machimosaurini); three sacral vertebrae (shared with *Lemmysuchus* and potentially other members of *Machimosaurus*); postacetabular iliac process is fan-shaped (shared with *Charitomenosuchus*, *Neosteneosaurus* and *Lemmysuchus*); posteroventral margin of ischial plate is sub-square (shared with *Lemmysuchus*); tibial tuberosity angled ventrally (shared with *Lemmysuchus*); dorsal osteoderms ornamented with small-to-large, irregularly shaped pits that radiate from the centre of the keel and are arranged in a starburst pattern (similar to an extent in *Lemmysuchus*).

**Remarks**—the diagnosis of *Machimosaurus mosae* has until recently been uncertain. Sauvage & Liénard (1879) initially diagnosed this taxon based on an incomplete skull, mandible and postcranial material. However, Krebs (1967) viewed it as a junior synonym of *Machimosaurus hugii*. Hua (1999) then regarded it as a distinct taxon and proposed a new diagnosis for it, based on a new specimen from the Kimmeridgian of Boulonnais (northwestern France) containing the skull, mandible and partial postcranial material. Pierce, Angielczyk & Rayfield (2009) also considered *Mac. mosae* to be distinct from *Mac. hugii*, due to the position of it within their geometric morphometric analysis.

However, Martin & Vincent (2013: 194) criticized Hua (1999) and Pierce, Angielczyk & Rayfield (2009)’s diagnoses, writing ‘most of the content of these diagnoses reveal to be either diagnostic at the genus level or to characterize all Teleosauridae’. Martin & Vincent (2013: 195) then showed that high variation in maxillary and dentary tooth counts among the various Callovian teleosaurids is ‘sufficient difference to discard such an interpretation (the synonymy)’. Martin & Vincent (2013) synonymized *Mac. mosae* with *Mac. hugii*, thus re-opening an old debate as to whether *Machimosaurus* represented a monotypic genus, or if the differences found between *Mac. mosae* and *Mac. hugii* were ontogenetic. However, other subsequent studies by Vignaud (1995), Hua (1999) and Young et al. (2014) all considered *Mac. mosae* to be taxonomically distinct from *Mac. hugii*. Importantly, Young et al. (2014) outlined five distinct points that strengthen the separation of *Mac. mosae* from *Mac. hugii*:

1. The *Mac. mosae* neotype is equivalent in size to *Mac. buffetauti* skulls from France and Germany;
2. Lack of juvenile characteristics in any of the French and German *Mac. buffetauti* skulls;
3. The *Mac. mosae* neotype exhibits exostoses (the formation of new bone) in the femur, right pubis, and some caudal vertebrae;
4. There is a 3- to 5-million-year gap between the *Mac. mosae* neotype and the *Mac. hugii* skulls; and
5. Loss of the prearticulars in *Mac. mosae*, which are present in *Mac. hugii*.

There are also certain postcranial features that differentiate *Mac. mosae* and *Mac. hugii*, including the shape and size of the coracoid postglenoid and glenoid processes (Young et al., 2014).

*Machimosaurus hugii* (Von Meyer, 1837) emend. Von Meyer, 1838

(Fig. 27)

**Figure 27:**
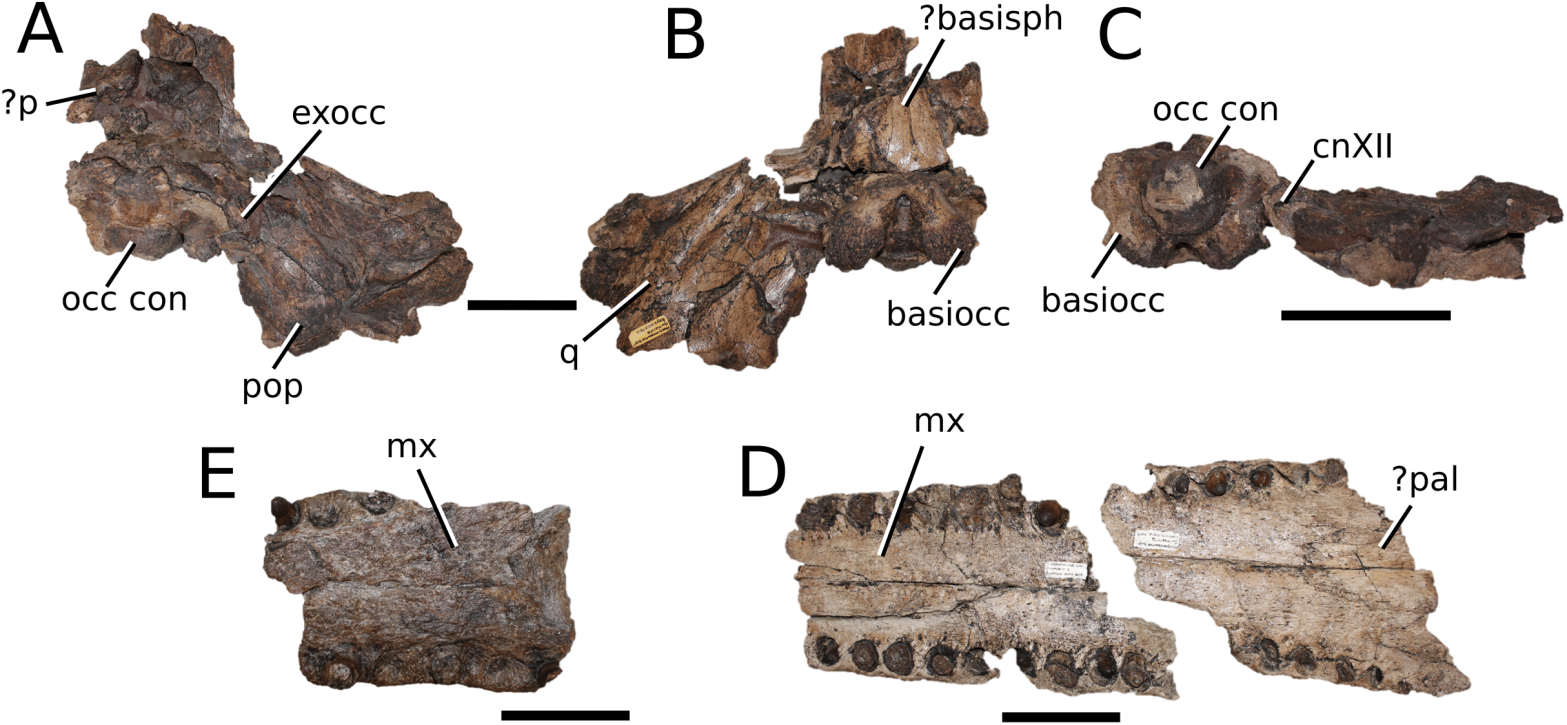
*Machimosaurus hugii*. *Machimosaurus hugii* (Von Meyer, 1837) emend. Von Meyer, 1838, MG-8730, referred specimen. (A–C) MG-8730-2: occipital in (A) dorsal, (B) ventral and (C) occipital views. (D and E) MG-8730-1: partial rostrum in (D and E) palatal view. Refer to abbreviation list. Scale bars: 10 cm.

**Holotype**—Von Meyer (1837, 1838) never designated a holotype; when establishing *Mac. hugii*, he referred to isolated tooth crowns from Solothurn, Switzerland and Kahlenberg, Germany (syntypes).

**Lectotype**—NMS 8342, an isolated tooth crown. Designation by Krebs (1967).

**Referred material**—MCNV-CC-4 (isolated tooth crown); MG-25; MG-8730-1 (two rostral pieces); MG-8730-2 (occipital section); MG unnumbered; ML 647; ML 491; ML 657; ML 658; (isolated teeth); Young et al. (2014).

**Age**—Kimmeridgian, Late Jurassic.

**Localities**—Kreuzen Quarry at St. Verena, near Solothurn, Canton Solothurn, Switzerland; Guimarota coalmine, Leiria, NW Portugal.

**Stratigraphic horizon**— ‘*Rätschenbank der Schildkrötenschichten*’ (“Solothurn Turtle Limestone, Reuchenette Formation”); Guimarota Strata, Alcobaça Formation.

**Scoring sources**—MG-8730-1, MG-8730-2 and MG unnumbered were examined first-hand, along with multiple teeth (e.g. LMH 16386; LMH 16399; MG 25; NZM-PZ R.2358a-g; SMF R 434a-b). Additional information was taken from Young et al. (2014).

**Autapomorphic characters of *Mac. hugii***—external surfaces of the cranial bones are poorly ornamented, particularly the rostrum and near the orbits; paraoccipital processes greatly enlarged, mediolaterally elongated and with expanded lateral ends, and are larger than the exoccipital-opisthotics; in occipital view, the inter-basioccipital tubera notch is a large inverse ‘U’-shape; dentary interalveolar spacing uniformly narrow.

**Emended diagnosis**—mesorostrine skull; groove-ridged ornamentation present along the maxilla (shared with *Mac. mosae* and *Mac. rex*); circular orbits (shared with *Mystriosaurus*, *Indosinosuchus*, *Teleosaurus*, *Mycterosuchus*, *Clovesuurdameredeor*, *Lemmysuchus* and other members of *Machimosaurus*); frontal width sub-equal to orbital width (shared with the Chinese teleosauroid, *I. kalasinensis*, *Macrospondylus*, *Clovesuurdameredeor*, *Seldsienean*, *Deslongchampsina*, *Proexochokefalos*, *Yvridiosuchus* and *Mac. rex*); parallelogram-shaped supratemporal fenestrae (shared with other members of Machimosaurini); circular orbits (shared with *Mystriosaurus*, *Indosinosuchus*, *Teleosaurus*, *Mycterosuchus*, *Clovesuurdameredeor*, *Lemmysuchus* and other members of *Machimosaurus*); shallow Meckelian groove (shared with *Proexochokefalos*, *Neosteneosaurus* and other members of Machimosaurini); deep, pronounced reception pits throughout the entirety of the maxilla (shared with *Andrianavoay*, S. *rostromajor*, *Neosteneosaurus* and other members of Machimosaurini); teeth large and conical with blunt apices (shared with other members of Machimosaurini); teeth not mediolaterally compressed (shared with *Bathysuchus* and other members of Machimosaurini); carinae heterogeneous with faint denticles (shared with other members of Machimosaurini); presence of keeled carinae variable (shared with *Mac. buffetauti* and *Mac. rex*); teeth with anastomosing pattern on the apical surface (shared with other members of Machimosaurini); pseudodenticles present (shared with *Mac. rex*); dorsal osteoderm ornamentation composed of small-to-large, well separated, irregularly shaped, randomly arranged pits.

**Remarks**—In response to Young et al. (2014)’s proposal that the genus *Machimosaurus* consisted of four distinct species, Martin, Vincent & Falconnet (2015) wrote a brief rebuttal, hypothesising that *Machimosaurus* was monospecific and *Mac. hugii* was the only representative of the genus. Foffa, Young & Brusatte (2015) then addressed the rebuttal put forth by Martin, Vincent & Falconnet (2015), noting that the authors did not address the monospecifity of *Machimosaurus* but rather concentrated on the validity of *Mac. buffetauti*, suggesting that it is the same as *Mac. mosae* and that both should be referred to *Mac. hugii* (as proposed by Martin & Vincent (2013)). Martin, Vincent & Falconnet (2015) claimed that intraspecific variation or post-mortem deformation accounted for the diagnoses put forth by Young et al. (2014); however, while acknowledging that the specimens did undergo some deformation, Foffa, Young & Brusatte (2015) argued that Young et al. (2014)’s diagnoses consisted of accurate morphological traits. In addition, both Young et al. (2014) and Foffa, Young & Brusatte (2015) listed six additional factors that differentiated *Machimosaurus* species:

1. Stratigraphy;
2. Basioccipital cross-sections;
3. Comparable size and shape of basioccipital tuberosities;
4. Comparable size and lateral expansion of the paraoccipital processes;
5. Dental morphology, as well as enamel traits; and
6. Tooth counts.

*Machimosaurus rex* Fanti et al., 2016

(Fig. 28)

**Figure 28:**
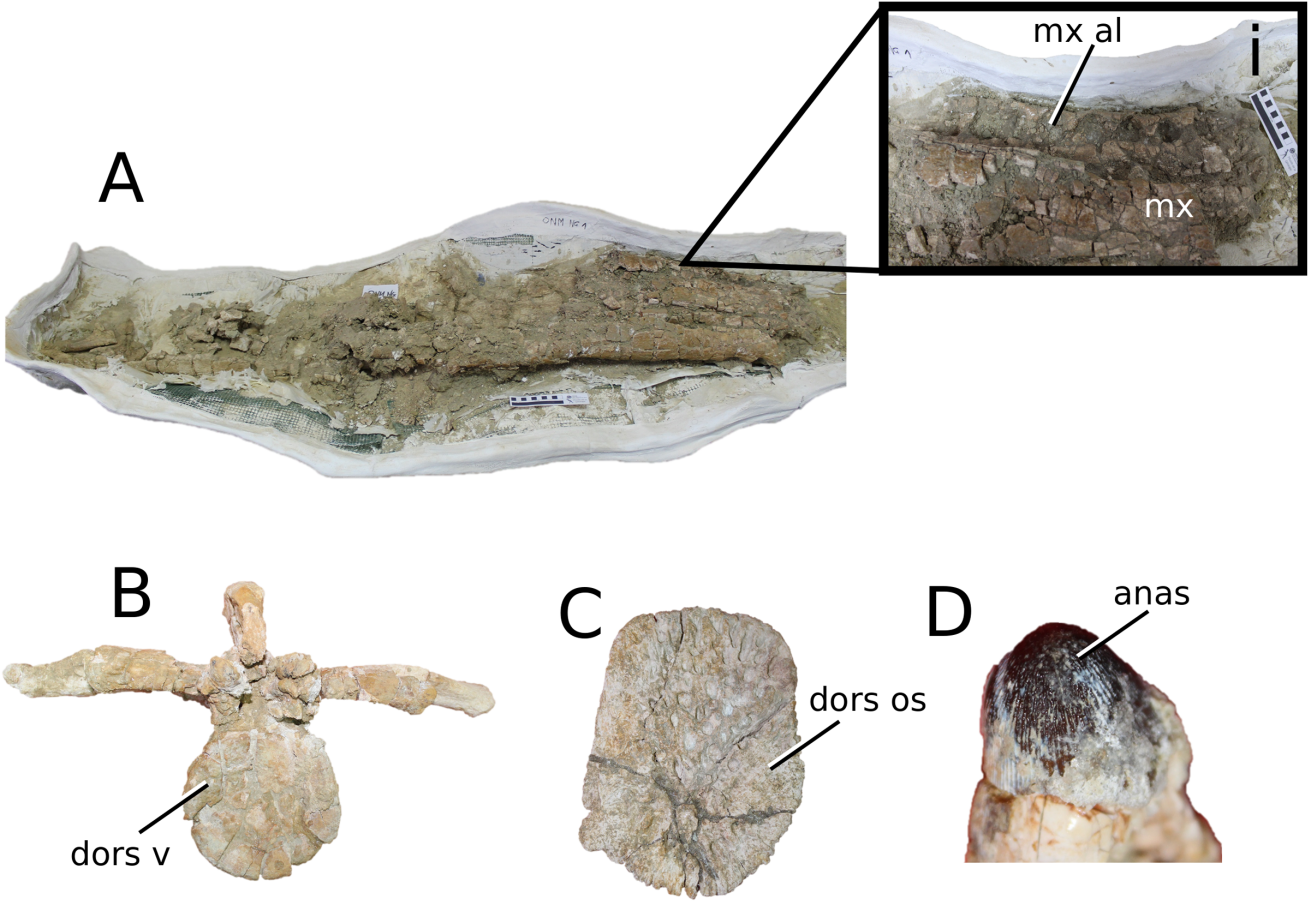
*Machimosaurus rex*. *Machimosaurus rex* Fanti et al., 2016, ONM NG 1-25, holotype. Partial skull in (A) ventral view, with a close-up of the (i) maxillary alveoli. Additional material: (B) dorsal vertebra in anterior view; (C) dorsal osteoderm; and (D) close-up of tooth apex. Refer to abbreviation list. Scale bars: 10 cm (as indicated on A), 5 cm (B and C) and 1 cm (D).

**Holotype**—ONM NG 1–25, 80, 81 and 83–87, comprising a fragmented, partially complete skull in association with pieces of the atlas-axis complex, two complete dorsal vertebrae, multiple fragments, and isolated osteoderms and teeth.

**Age**—late Hauterivian/early Barremian, Early Cretaceous.

**Locality**—Touil el Mhahir, Tataouine Governorate, Tunisia.

**Stratigraphic horizon**—Douiret Sand Member, Douiret Formation.

**Scoring sources**—the holotype was examined first-hand.

**Emended diagnosis**—mesorostrine skull; conspicuous groove-ridged ornamentation along the maxilla (shared with *Mac. mosae* and *Mac. hugii*); frontal width sub-equal to orbital width (shared with the Chinese teleosauroid, *I. kalasinensis*, *Macrospondylus*, *Clovesuurdameredeor*, *Seldsienean*, *Deslongchampsina*, *Proexochokefalos*, *Yvridiosuchus* and *Mac. hugii*); circular orbits (shared with *Mystriosaurus*, *Indosinosuchus*, *Teleosaurus*, *Mycterosuchus*, *Clovesuurdameredeor*, *Lemmysuchus* and other members of *Machimosaurus*); parallelogram-shaped supratemporal fenestrae (shared with other members of Machimosaurini); teeth large and conical with blunt apices (shared with other members of Machimosaurini); teeth not mediolaterally compressed (shared with *Bathysuchus* and other members of Machimosaurini); carinae heterogeneous with faint denticles (shared with other members of Machimosaurini); presence of keeled carinae variable (shared with *Mac. buffetauti* and *Mac. hugii*); teeth with anastomosing pattern on the apical surface (shared with other members of Machimosaurini); pseudodenticles present (shared with *Mac. hugii*); dorsal osteoderm ornamentation consists of pits with variable size, shape and distribution (similar *Lemmysuchus*, *Mac. buffetauti* and *Mac. mosae*).

**Remarks**—While Fanti et al. (2016) described this specimen as being Hauterivian in age, the exact age is unclear, due to uncertainty of the geological age of the area, as well as previously disregarded biostratigraphic invertebrate fauna (Dridi & Johnson, 2019; Dridi, 2020). It is also important to note that *Mac. rex* does not display any autapomorphic characters, given its extremely poor preservation.

## Character Descriptions

### New characters pertaining to teleosauroids

The 38 new characters introduced here were formulated to describe thalattosuchian, specifically teleosauroid, anatomical variation. These characters are relevant to the interrelationships of teleosauroids, and many highlight previously unexamined morphological divergence between two large subclades within the group (see below). These characters are new and are here used in a cladistic analysis for the first time, and all states (indicated by a number in brackets) are subsequently figured. Character numbering follows the numbering used in the full list of characters for the present analysis (see Supplemental Data SD1). More detailed descriptions and comparisons of all following characters have been provided in the Supplemental Data SD4.

**Figure 29:**
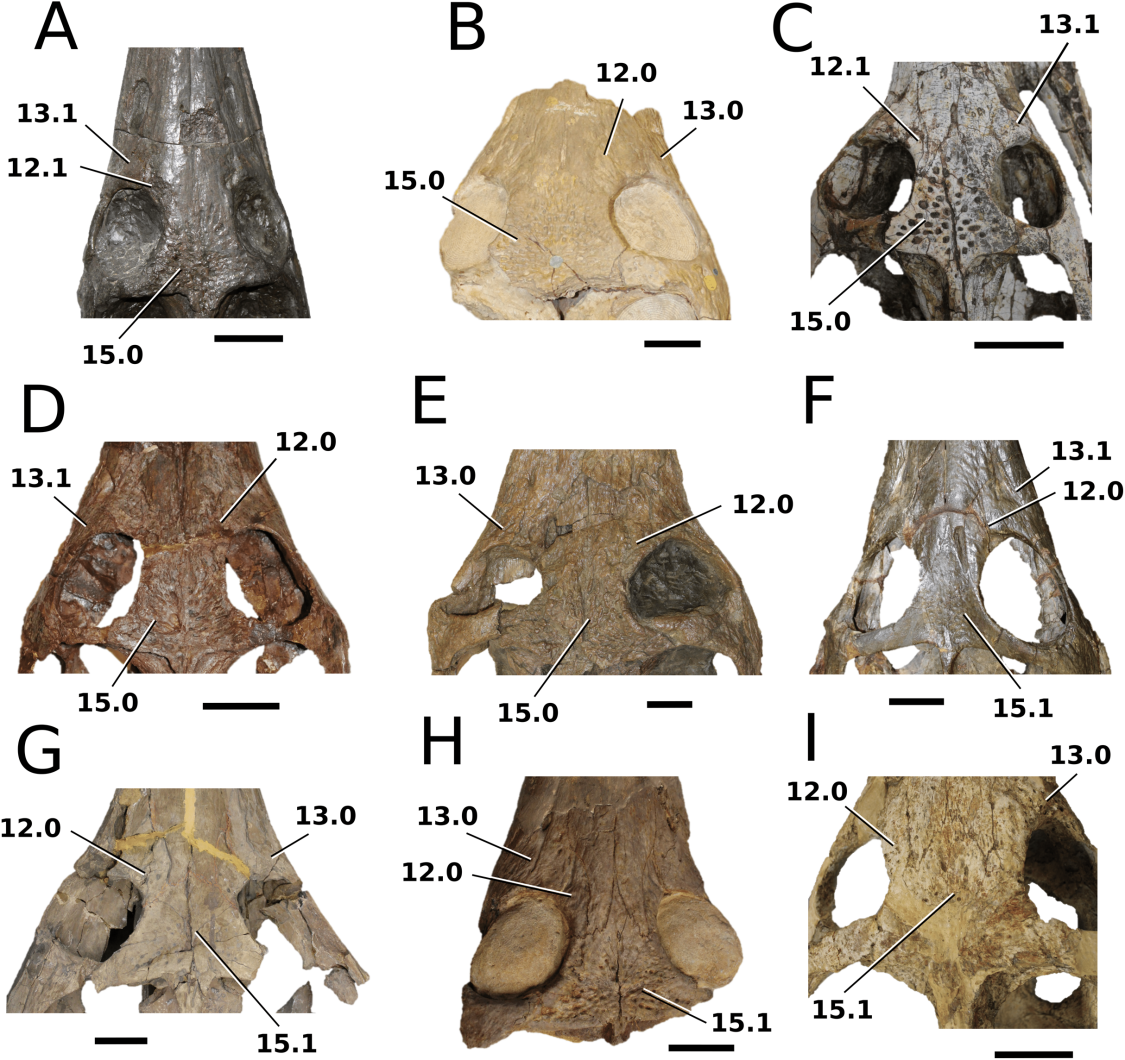
Comparative photographs: ornamentation on the prefrontal, lacrimal and frontal. Comparative photographs displaying ornamentation on the prefrontal (ch. 12), lacrimal (ch. 13) and frontal (ch. 15) in dorsal view. (A) *Plagiophthalmosuchus gracilirostris* (NHMUK PV R 14892); (B) *Clovesuurdameredeor stephani* (NHMUK PV OR 49126); (C) *Indosinosuchus potamosiamensis* (PRC-11); (D) the Chinese teleosauroid (IVPP V 10098); (E) *Mycterosuchus nasutus* (NHMUK PV R 2617); (F) *Charitomenosuchus leedsi* (NHMUK PV R 38060); (G) *Neosteneosaurus edwardsi* (NHMUK PV R 2865); (H) *Yvridiosuchus boutilieri* (OUMNH J.1401); and (I) *Machimosaurus buffetauti* (SMNS 91415). Scale bars: 4 cm.

**12.** Ornamentation on prefrontal in dorsal view: present, with shallow to deep pits and/or grooves (0), or absent (1) (Fig. 29).

This character was inspired by the variety of ornamentation patterns found on the prefrontal of teleosauroid taxa. Ornamentation is either absent (state 1) or comes in the form of shallow to deep pits or shallow to deep, elongated and thin grooves (state 0). State 1 occurs in very few teleosauroids, including the basal teleosauroid *Plagiophthalmosuchus* (NHMUK PV OR 14792), *I. potamosiamensis* (PRC-11), *Aeolodon* (MNHN.F.CNJ 78), *Sericodon* (Schaefer, Püntener & Billon-Bruyat, 2018) and *Bathysuchus* (Foffa et al., 2019). The majority of teleosauroids are scored as state 0, including the Chinese teleosauroid (IVPP V 10098), *Platysuchus* (SMNS 9930), *Mycterosuchus* (NHMUK PV R 2617), *Macrospondylus* (GPIT-RE-9427; MMG BwJ 565; SMNS 51555), *Charitomenosuchus* (NHMUK PV R 3320), *Proexochokefalos* (MNHN.F 1890-13), and machimosaurins (*Yvridiosuchus*: OUMNH J.1401; *Lemmysuchus*: LPP.M.21; *Mac. buffetauti*: SMNS 91415).

**13.** Ornamentation on lacrimal in dorsal view: present (0), with shallow to deep pits and/or grooves, or absent (1) (Fig. 29).

As with the above character, the ornamentation displayed on the lacrimal (=lachrymal) differs between taxa. Ornamentation is either absent (state 1) or comes in the form of shallow to deep pits, as well as shallow to deep, elongated and thin grooves (state 0). The majority of teleosauroids (*Mystriosaurus*: NHMUK PV OR 14781; *Platysuchus*: SMNS 9930; *Mycterosuchus*: NHMUK PV R 2617; *Proexochokefalos*: MNHN.F 1890-13; *Lemmysuchus*: NHMUK PV R 3168; *Mac. buffetauti*: SMNS 91415) exhibit state 0, with some form of ornamentation being present. State 1 (lack of ornamentation) occurs in six taxa: *I. potamosiamensis* (PRC-11), *Aeolodon* (MNHN.F.CNJ 78), *Plagiophthalmosuchus* (NHMUK PV OR 14792), *Macrospondylus* (SMNS 51563), *Charitomenosuchus* (NHMUK PV R 3320) and *Sericodon* (Schaefer, Püntener & Billon-Bruyat, 2018). As discussed in ch. **12**, lack of ornamentation has previously been attributed to juveniles (Vignaud, 1995); however, this character was scored using adult specimens.

**15.** Frontal, extension of ornamentation: extends from the centre of the frontal to lateral- and anterior-most regions (0), restricted to centre of the frontal (1) or no ornamentation (2) (Fig. 29).

The frontal of teleosauroids is a single bone that is consistently ornamented throughout the majority of the group, excluding *Bathysuchus* (unnumbered LPP specimen) and juveniles (e.g. SMNS 10,000). Ornamentation either extends from the centre of the frontal to the anterior- and lateral-most areas (state 0) or is restricted to the midline or centre of the frontal (state 1), with minimal extension.

*Plagiophthalmosuchus* (NHMUK PV OR 14792), *Clovesuurdameredeor* (NHMUK PV OR 49126), *Macrospondylus* (MMG BwJ 565; SMNS 51563) and many basal teleosauroids (e.g. *Mystriosaurus*: NHMUK PV OR 14781; *Platysuchus*: SMNS 9930), display state 0. The majority of more derived teleosauroids (e.g. *Charitomenosuchus*: NHMUK PV R 3320; *Proexochokefalos*: MNHN.F 1890-13; *Lemmysuchus*: LPP.M.21; *Mac. buffetauti*: SMNS 91415), along with *Sericodon* (SCR010312 in Schaefer, Püntener & Billon-Bruyat (2018)) and *Aeolodon* (MNHN.F.CNJ 78), share state 1.

It has been suggested that *Bathysuchus* lacks any frontal ornamentation (Vignaud, 1995), similar to juvenile individuals. However, there may possibly be weak, nearly unnoticeable pits and grooves restricted to the midline of the frontal in this taxon (Fig.), in an LPP unnumbered specimen (Foffa et al., 2019). Due to this uncertainty, this taxon was scored as (?).

**Figure 30:**
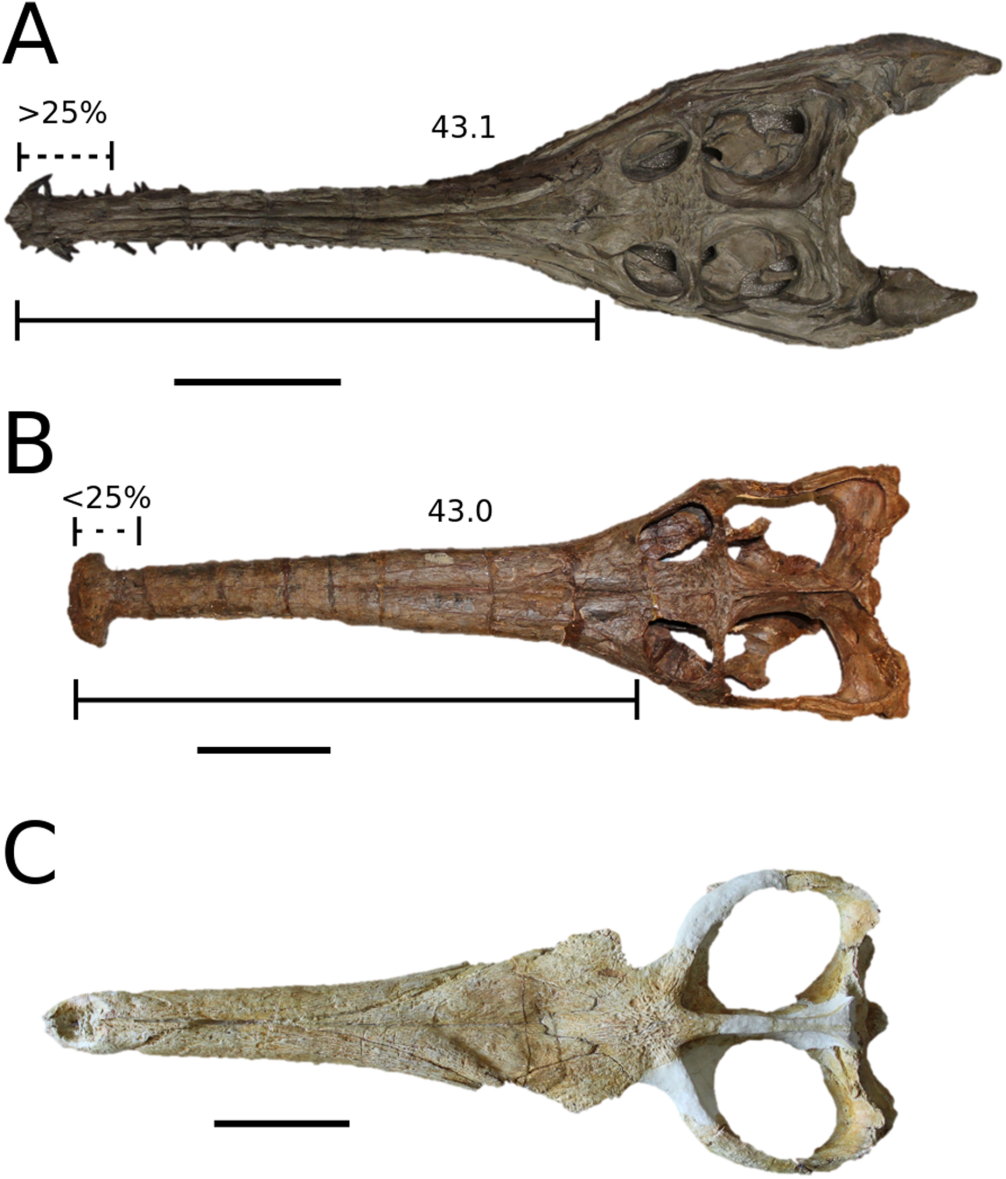
Comparative photographs: premaxillary anteroposterior length relative to rostrum length. Comparative photographs displaying premaxillary anteroposterior length relative to rostrum length (ch. 43): (A) *Macrospondylus bollensis* (SMNS 81672) and (B) the Chinese teleosauroid (IVPP V 10098), as well as (C) *Metriorhynchus superciliosus* (LPP.M.48). Dashed lines (- - - -) represent anteroposterior premaxillary length, while solid lines (^___^) represent total rostral length. Scale bars: 10 cm.

**43.** Premaxilla in dorsal view, the total anteroposterior length relative to total rostrum length is less than 25% (0) or approximately 25% or greater (1) (Fig. 30).

This character focuses on the total anteroposterior premaxillary length in relation to the total anteroposterior rostrum length of a cranium. When defining the rostral length, this refers to the length between the anterior-most premaxillae to the anterior orbital margin.

In the majority of teleosauroids, the premaxillary anteroposterior length is greater than 25% relative to the rostral length (state 1). This condition is observed in the basal teleosauroid *Plagiophthalmosuchus* (NHMUK PV OR 14792), as well as many longirostrine taxa that are (e.g. *Indosinosuchus*: PRC239; *Mycterosuchus*: NHMUK PV R 2617; *Macrospondylus*: SMNS 18672; *Proexochokefalos*: MNHN.F 1890-13; *Lemmysuchus*: NMHUK PV R 3168). Few teleosauroids have a premaxillary anteroposteriorly length that is less than 25% of the rostral length (state 0). This is seen in *Mac. buffetauti* (SMNS 91415) and *Mac. mosae* (IRSNB cast; Hua, 1999) as well as *Mystriosaurus* (NHMUK PV OR 14781) and the Chinese teleosauroid (IVPP V 10098).

**Figure 31:**
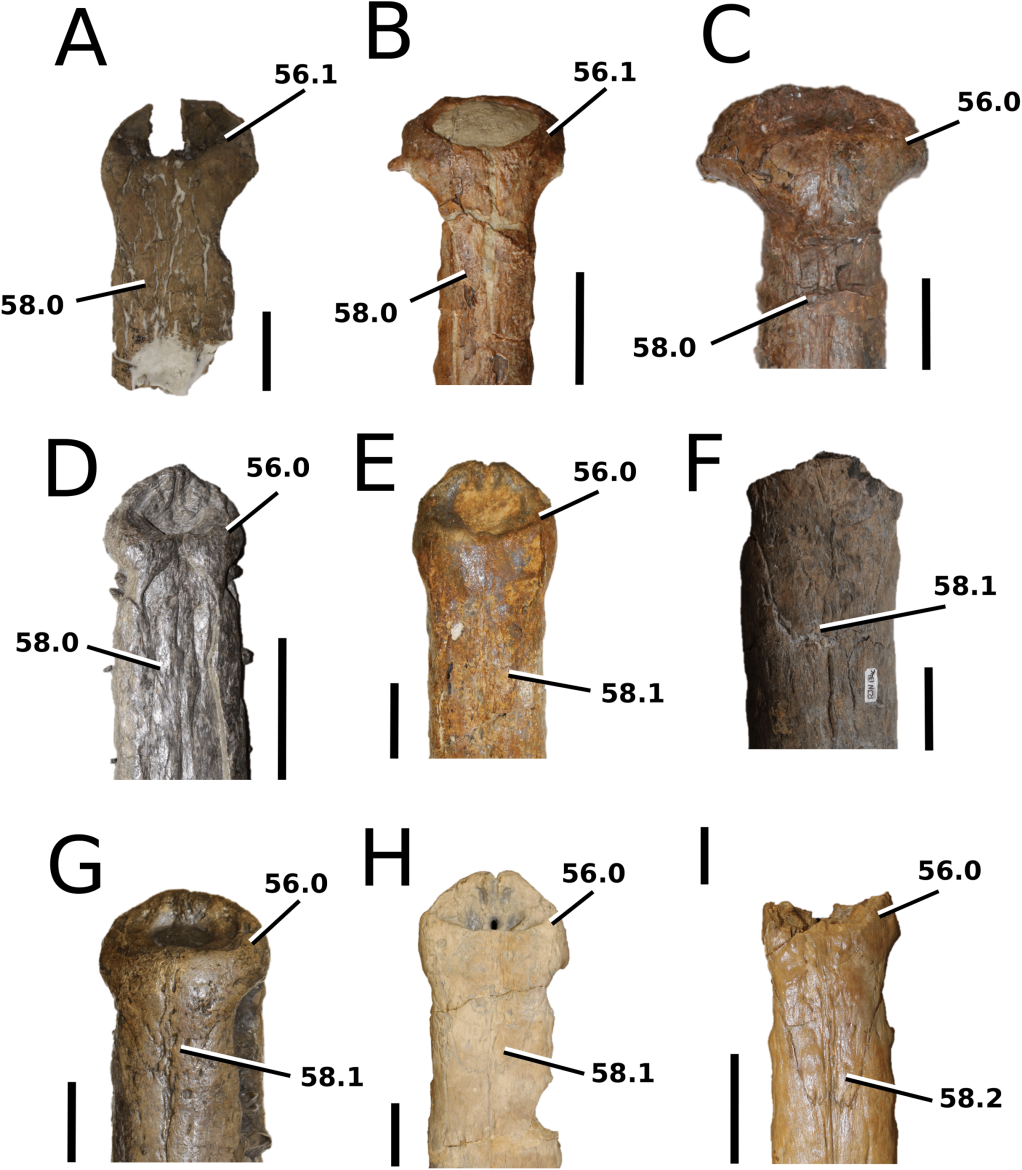
Comparative photographs: medial margins of the external nares, and the premaxilla-maxilla suture. Comparative photographs displaying medial margins of the external nares (ch. 56) and the premaxilla-maxilla suture (ch. 58): (A) *Mycterosuchus nasutus* (CAMSM J.1420), (B) *Bathysuchus megarhinus* (unnumbered LPP specimen), (C) the Chinese teleosauroid (IVPP V 10098), (D) *Macrospondylus bollensis* (MMG BwJ 565), (E) *Deslongchampsina larteti* (OUMNH J.29851), (F) *Steneosaurus rostromajor* (MNHN.RJN 134c-d), (G) *Mystriosaurus laurillardi* (NHMUK PV OR 14781), (H) *Neosteneosaurus edwardsi* (NHMUK PV R 2685) and (I) *Charitomenosuchus leedsi* (NHMUK PV R 3320). Scale bars: 3 cm.

**56.** Premaxilla in dorsal view, the anterior and posterior medial margins of the external nares are formed by two bulbous projections, which are either absent (0) or present (1) (Fig. 31).

In most teleosauroids, the medial margins of the external nares are minimally convex (state 0), causing the external nares to appear D-shaped in dorsal view. This is the condition seen in the basal *Plagiophthalmosuchus* (NHMUK PV OR 14792) in addition to *Mystriosaurus* (NHMUK PV R OR 14781), *Indosinosuchus* (PRC11; PRC-239), the Chinese teleosauroid (IVPP V 10098), *Platysuchus* (SMNS 9930), *Macrospondylus* (MMG BwJ 565), *Charitomenosuchus* (NHMUK PV R 3806), *Proexochokefalos* (MNHN.F 1890-13), *Neosteneosaurus* (NHMUK PV R 2865) and Machimosaurini (e.g. *Lemmysuchus*: NHMUK PV R 3168).

In certain taxa, however, both the anterior and posterior margins are strongly convex, and appear ‘bulging’ in dorsal view. This condition (state 1) is synapomorphic in a unique clade containing *Mycterosuchus* (NHMUK PV R 2617), *Bathysuchus* (unnumbered LPP specimen) (Foffa et al., 2019), and possibly *Aeolodon* (MNHN.F.CNJ 78) (however, specimens of this taxon are dorsoventrally crushed and slightly distorted, so it is difficult to say with certainty if it is present).

**58.** Premaxilla in dorsal view, the shape of the anteroposterior premaxilla-maxilla contact is triangular (0), subcircular (1) or ‘ragged’ (2) (Fig. 31).

In the basal-most form (*Plagiophthalmosuchus*: NHMUK PV OR 14792), as well as the Chinese teleosauroid (IVPP V 10098); *Indosinosuchus* (PRC-11; PRC-239); *Platysuchus* (SMNS 9930); *Aeolodon* (MNHN.F.CNJ 78), *Mycterosuchus* (NHMUK PV R 2617), *Bathysuchus* (unnumbered LPP specimen) and *Macrospondylus* (SMNS 51753; SMNS 51984), the contact is triangular with slight or no interdigitating areas (state 0). An intermediate condition (state 1) shows the contact to be anteroposteriorly short and subcircular in shape (more posteromedially horizontally oriented than state 0), with a weak to moderate degree of interdigitating regions, generally close to the midline of the rostrum. This occurs in *S*. *rostromajor* (MNHN.RJN 134c-d) as well as *Mystriosaurus* (NHMUK PV OR 14781), *Andrianavoay* (NHMUK PV R 1999), *Proexochokefalos* (MNHN.F 1890-13), *Neosteneosaurus* (NHMUK PV R 2865) and Machimosaurini (e.g. *Lemmysuchus*: NHMUK PV R 3168, LPP.M.21). A third condition (state 2) is autapomorphic to *Charitomenosuchus* (NHMUK PV R 3320, NHMUK PV R 3806): the premaxilla-maxilla suture is anteroposteriorly elongated, sub-rectangular and highly interdigitating, giving it a ‘ragged’-like appearance.

**Figure 32:**
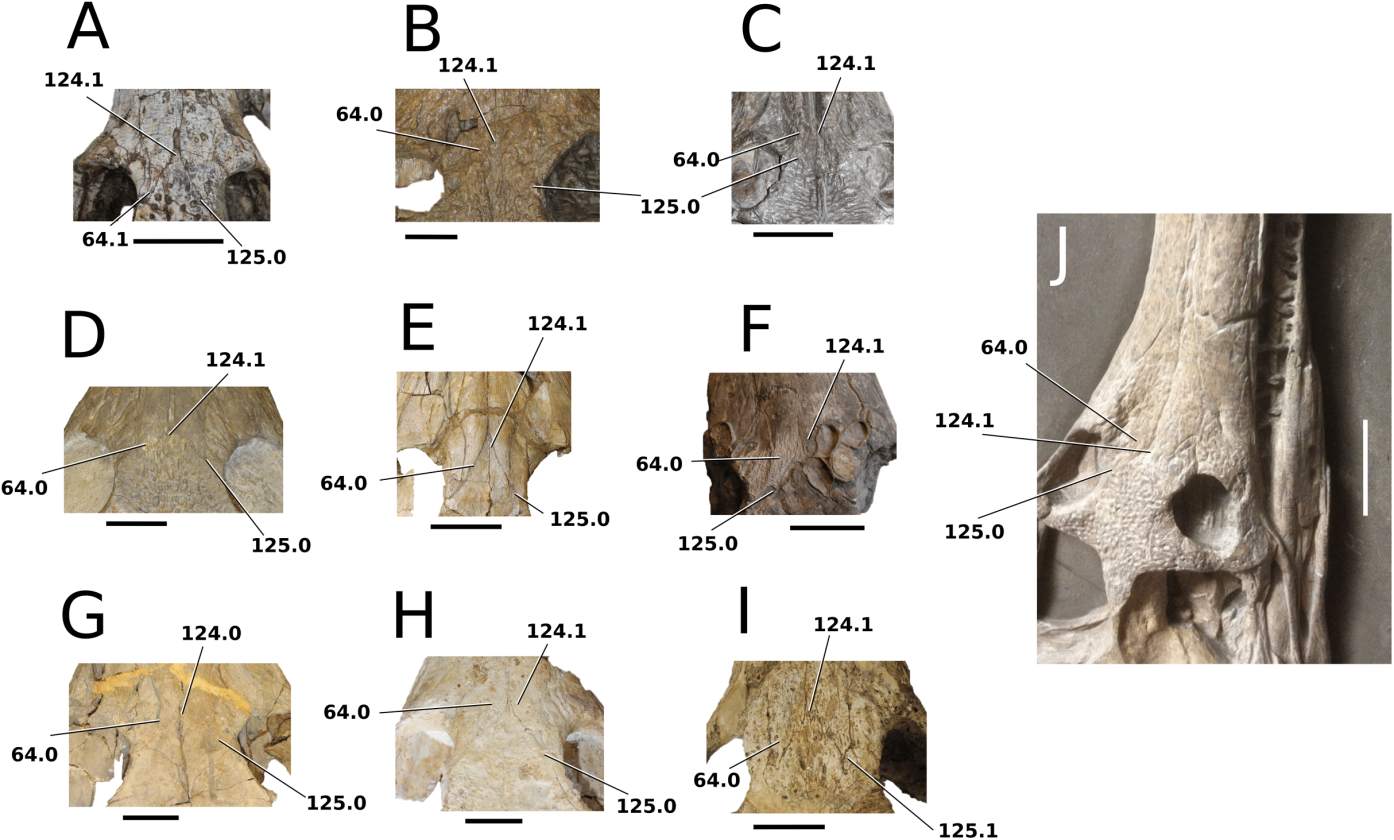
Comparative photographs: elongated posterior nasal processes, anteromedial frontal process, and additional anterolateral frontal projections. Comparative photographs displaying the presence/absence of elongated posterior nasal processes (ch. 64), anteromedial frontal process (ch. 124) and additional anterolateral frontal projections (ch.125): (A) *Indosinosuchus potamosiamensis* (PRC-11), (B) *Mycterosuchus nasutus* (NHMUK PV R 2617), (C) *Macrospondylus bollensis* (NHMW-1878-0047-0001), (D) *Clovesuurdameredeor stephani* (NHMUK PV OR 49126), (E) *Charitomenosuchus leedsi* (NHMUK PV R 3320), *Neosteneosaurus edwardsi* ((F): MNHN.RJN 118; (G) NHMUK PV R 2865), (H) *Lemmysuchus obtusidens* (LPP.M.21), (I) *Machimosaurus buffetauti* (SMNS91415) and (J) *Platysuchus multiscrobiculatus* (SMNS 9930). *Platysuchus* photograph provided by MTY. Scale bars: 4 cm.

**64.** Nasals, elongate posterior process that does not (0) or does (1) contact anterior rim of orbit (Fig. 32).

In the majority of teleosauroids (e.g. the Chinese teleosauroid: IVPP V 10098; *Platysuchus*: SMNS 9930; *Mycterosuchus*: NHMUK PV R 2617; *Lemmysuchus*: LPP.M.21), including the basal-most teleosauroid (*Plagiophthalmosuchus*: NHMUK PV OR 14792), the posterior processes of the nasals reach or extend slightly past the anterior rim of the orbits (state 0). In addition, these processes are positioned medially, slightly mediolaterally thin in the posterior-most area, and do not come into close contact with the medial orbital margin. However, *I. potamosiamensis* (PRC-11) clearly possesses state 1, in which the nasals have extraordinarily anteroposteriorly elongated posterior processes; these are mediolaterally thin and contacts the medial rim of the orbit (see Martin et al., 2019).

**124.** Frontal, anteromedial process shape and length relative to nasals: anterior projection of frontal is mediolaterally broad and does not extend far anteriorly past anterior orbital rim into nasals (0) or anterior projection of frontal is mediolaterally thin and extends anteriorly past anterior orbital rim into nasals (1) (Fig. 32).

In the majority of teleosauroids, this process is triangular, thin and anteromedially elongated, usually extending past the anterior orbital margin (state 1). This is seen in taxa such as the basal-most form *Plagiophthalmosuchus* (NHMUK PV OR 14792) as well as *Mystriosaurus* (NHMUK PV OR 14781), the Chinese teleosauroid (IVPP V 10098), *Indosinosuchus* taxa (PRC 11; PRC 239), *Platysuchus* (SMNS 9930), *Mycterosuchus* (NHMUK PV R 2617), *Aeolodon* (MNHN.F.CNJ 78), *Macrospondylus* (MMG BwJ 565; SMNS 51555), *Charitomenosuchus* (NHMUK PV R 3320), *Deslongchampsina* (OUMNH J.29851), *Proexochokefalos* (MNHN.F 1890-13), *Neosteneosaurus* (MNHN.RJN 118; PETMG R178) and Machimosaurini (*Yvridiosuchus* OUMNH J.1401; *Lemmysuchus* LPP.M.21; *Mac. buffetauti* SMNS 91415). It is interesting to note that the anteromedial frontal processes in *Yvridiosuchus*, *Indosinosuchus*, *Charitomenosuchus* and *Mac. buffetauti* are considerably more elongated and mediolaterally thin than in the other aforementioned taxa.

Only one taxon, *Clovesuurdameredeor* (NHMUK PV OR 49126), expresses state 0, in which the anteromedial frontal process is noticeably mediolaterally broadened (giving it a subcircular appearance in dorsal view) and anteroposteriorly short.

**125.** Frontal in dorsal view, small anterolateral projections between nasals and prefrontals are absent (0) or present (1) (Fig. 32).

Most teleosauroids do not have these extra frontal projections; instead, the frontal suture is flush with that of the posterior nasal processes (state 0). This condition is clearly seen in the basal teleosauroid *Plagiophthalmosuchus* (NHMUK PV OR 14792) and the Chinese teleosauroid (IVPP V 10098), *Indosinosuchus* (PRC-11, PRC-239), *Platysuchus* (SMNS 9930), *Teleosaurus* (MNHN AC 8746), *Mycterosuchus* (NHMUK PV R 2617), *Aeolodon* (MNHN.F.CNJ 78), *Macrospondylus* (MMG BwJ 565), *Clovesuurdameredeor* (NHMUK PV OR 49126), *Charitomenosuchus* (NHMUK PV R 3320), *Deslongchampsina* (OUMNH J.29851), *Proexochokefalos* (MNHN.F 1890-13), *Neosteneosaurus* (NHMUK PV R 2865), *Yvridiosuchus* (OUMNH J.1401) and *Lemmysuchus* (LPP.M.21). The presence of these frontal projections is an apomorphic state, however, in the taxon *Mac. buffetauti* (Martin & Vincent, 2013; SMNS 91415), in which they are large, mediolaterally broadened and clearly noticeable (state 1).

**Figure 33:**
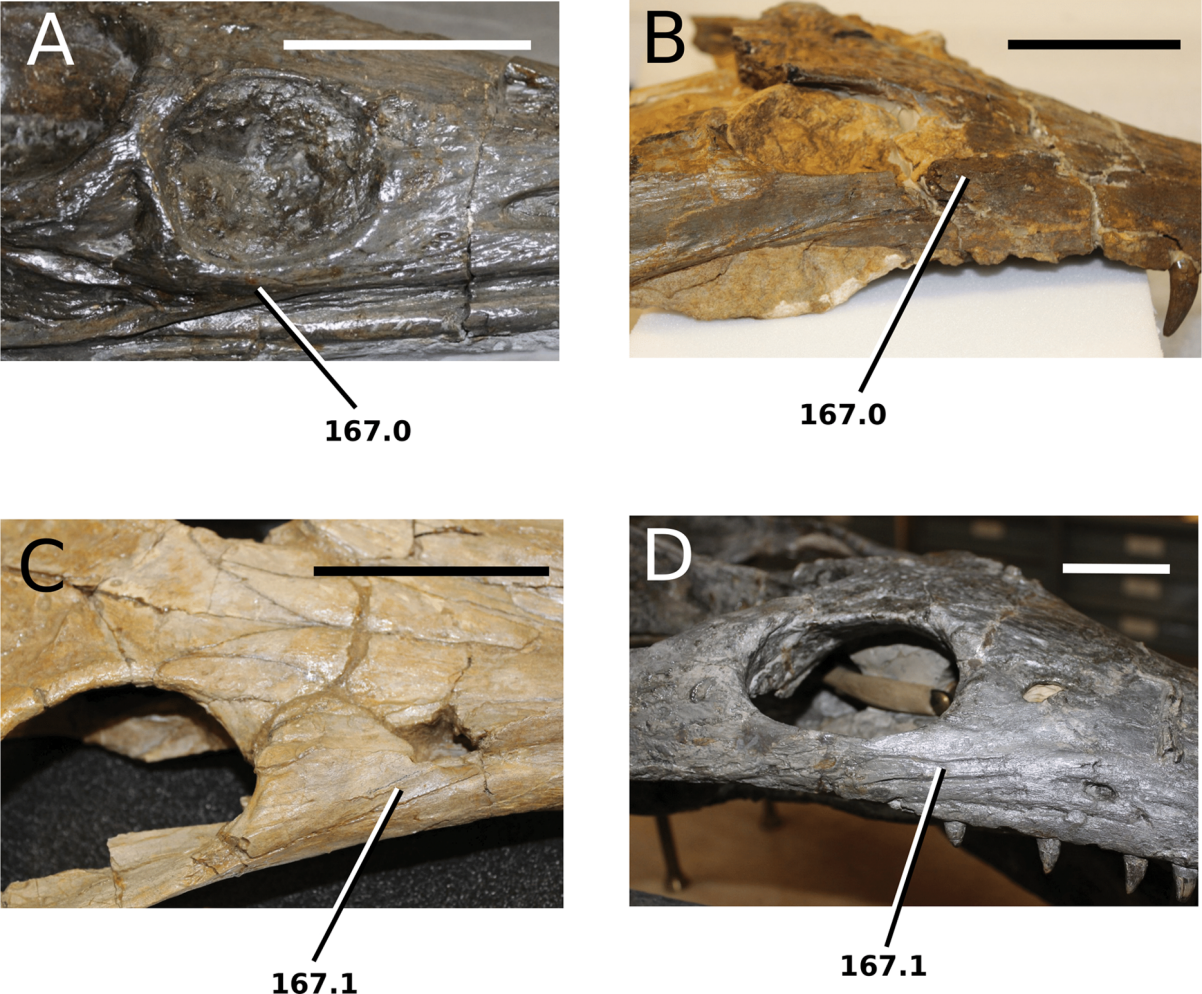
Comparative photographs: anterior elongation of the jugal. Comparative photographs displaying the anterior elongation of the jugal (ch. 167) in (A) *Plagiophthalmosuchus gracilirostris* (NHMUK PV OR 14792); (B) *Deslongchampsina larteti* (OUMNH J.29851); (C) *Charitomenosuchus leedsi* (NHMUK PV R 3320); and (D) *Proexochokefalos heberti* (MNHN.F 1890-13). Scale bars: 5 cm.

**167.** Jugal anterior process is absent (0) or is slender, elongated and extends anteriorly (1) (Fig. 33).

The majority of teleosauroids have a shortened anterior process of the jugal that does not extend past the anterior orbital margin (state 0). This is clearly seen in the basal form *Plagiophthalmosuchus* (MNHNL. TU515) as well as *Mystriosaurus* (NHMUK PV OR 14781), the Chinese teleosauroid (IVPP V 10098), *Platysuchus* (SMNS 9930), *Teleosaurus* (MNHN AC 8746), *Mycterosuchus* (NHMUK PV R 2617), *Macrospondylus* (PMU R161) and *Deslongchampsina* (OUMNH J.29851).

In certain teleosauroids, the anterior jugal becomes dorsoventrally curved, narrow and anteroposteriorly elongated, and extends substantially past the anterior orbital margin, at times nearly to the posterior region of the antorbital fenestra. This condition (state 1) is present in the taxa *Charitomenosuchus* (NHMUK PV R 3320), *Neosteneosaurus* (MNHN.RJN 118; PETMG R178), *Proexochokefalos* (MNHN.F 1890-130) and members of Machimosaurini (e.g. *Yvridiosuchus*: OUMNH J.1401).

**Figure 34:**
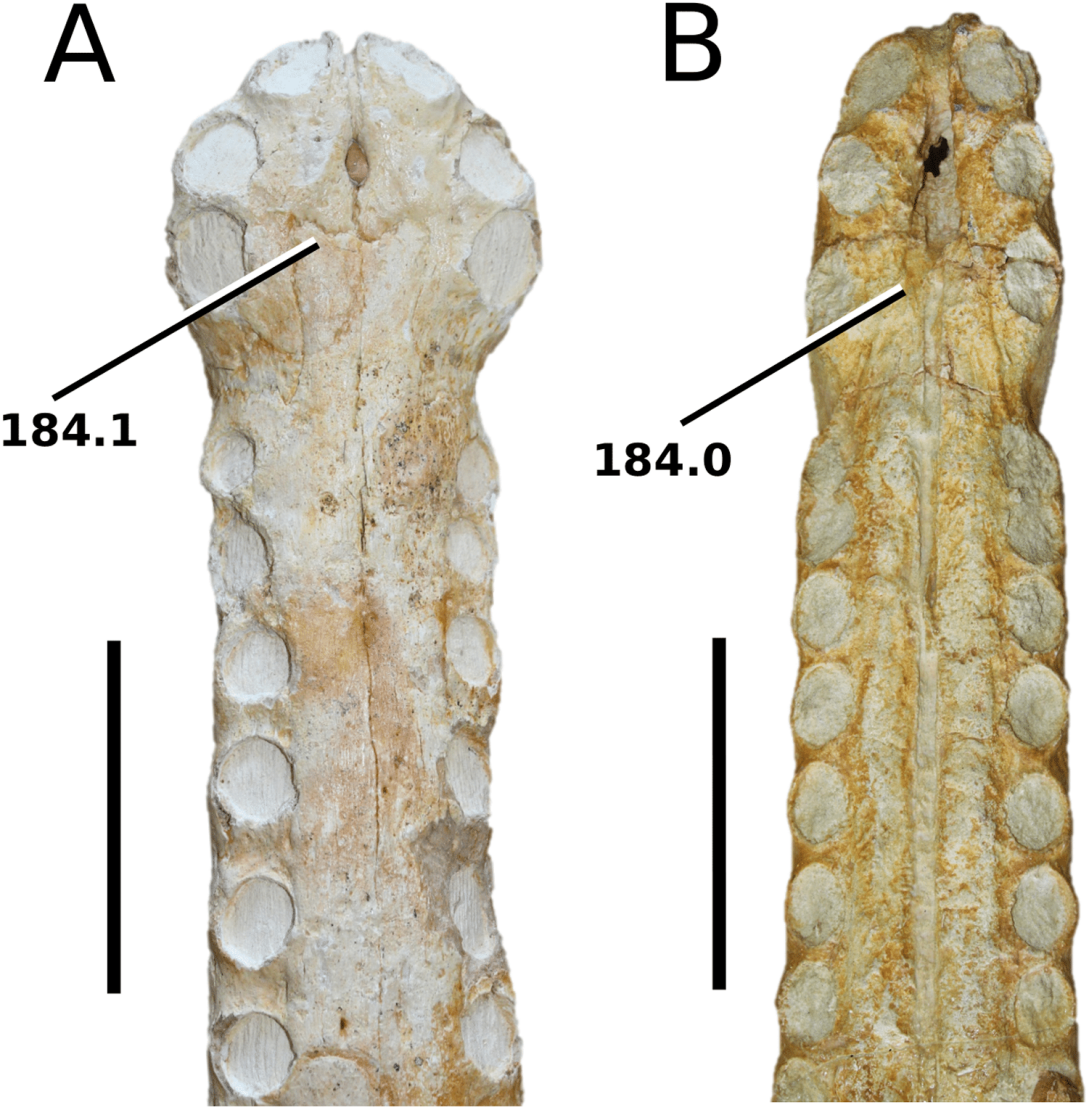
Comparative photographs: premaxillary-maxillary suture in palatal view. Comparative photographs displaying the premaxillary-maxillary suture in palatal view (ch. 184): (A) Teleosauroidea (*Lemmysuchus obtusidens* LPP.M.21) and (B) Metriorhynchoidea (*Metriorhynchus supercilious* LPP.M.48). Scale bars: 7 cm.

**184.** Maxilla in palatal view, shape of anterior maxilla is tapering (subtriangular) (0) or straightened (sub-rectangular) (1) (Fig. 34).

This character focuses on the anterior premaxilla-maxilla contact in palatal view, which is positioned parallel to the fourth premaxillary alveolus. State 1 is a synapomorphic character for members of Teleosauroidea (e.g. the Chinese teleosauroid: IVPP V 10098; *Yvridiosuchus*: OUMNH J.1401); the contact is horizontal and straight, and sub-rectangular in shape. This character is one key difference from Metriorhynchoidea, in which the contact is subtriangular and anteriorly directed (state 0) (e.g. *Metriorhynchus superciliosus*: LPP.M.48).

**Figure 35:**
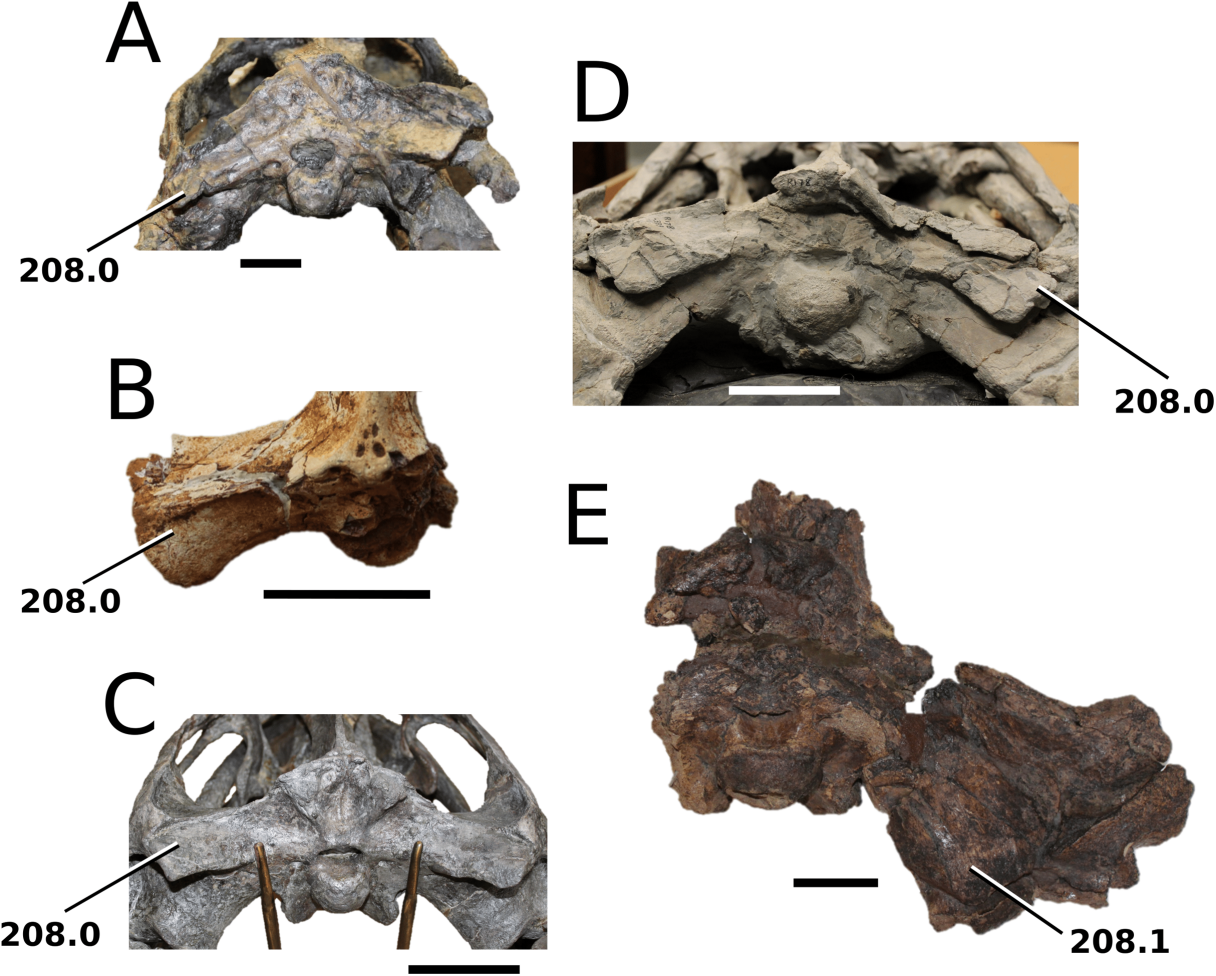
Comparative photographs: exoccipital and paraoccipital processes. Comparative photographs displaying the exoccipital and paraoccipital processes (ch. 208): (A) *Plagiophthalmosuchus gracilirostris* (MNHNL TU515), (B) ‘*Steneosaurus*’ sp. (IRSNB R 0140), (C) *Proexochokefalos heberti* (MNHN.F 1890-13), (D) *Neosteneosaurus edwardsi* (PETMG R178) and (E) *Machimosaurus hugii* (MG 8730). Scale bars: 5 cm.

**208.** Paraoccipital process approximately the same size (0) or substantially larger than the remainder of the exoccipital-opisthotic (1) (Fig. 35).

Generally, the paraoccipital processes (the posterior-most part of the exoccipital-opisthotics) are approximately the same size as the rest of the exoccipital-opisthotic (state 0). This is seen in the basal form *Plagiophthalmosuchus* (MNHNL TU515) as well as most teleosauroids (e.g. the Chinese teleosauroid: IVPP V 10098; *Platysuchus*: SMNS 9930; *Mycterosuchus*: NHMUK PV R 2617; *Macrospondylus*: SMNS 81699; *Charitomenosuchus*: NHMUK PV R 3320; *Proexochokefalos*: MNHN.F 1890-13; *Lemmysuchus*: NHMUK PV R 3168). In *Mac. hugii* (MG-8730-2), the paraoccipital processes are noticeably and substantially larger than the remaining exoccipital-opisthotics; this condition (state 1) is autapomorphic for this taxon.

**Figure 36:**
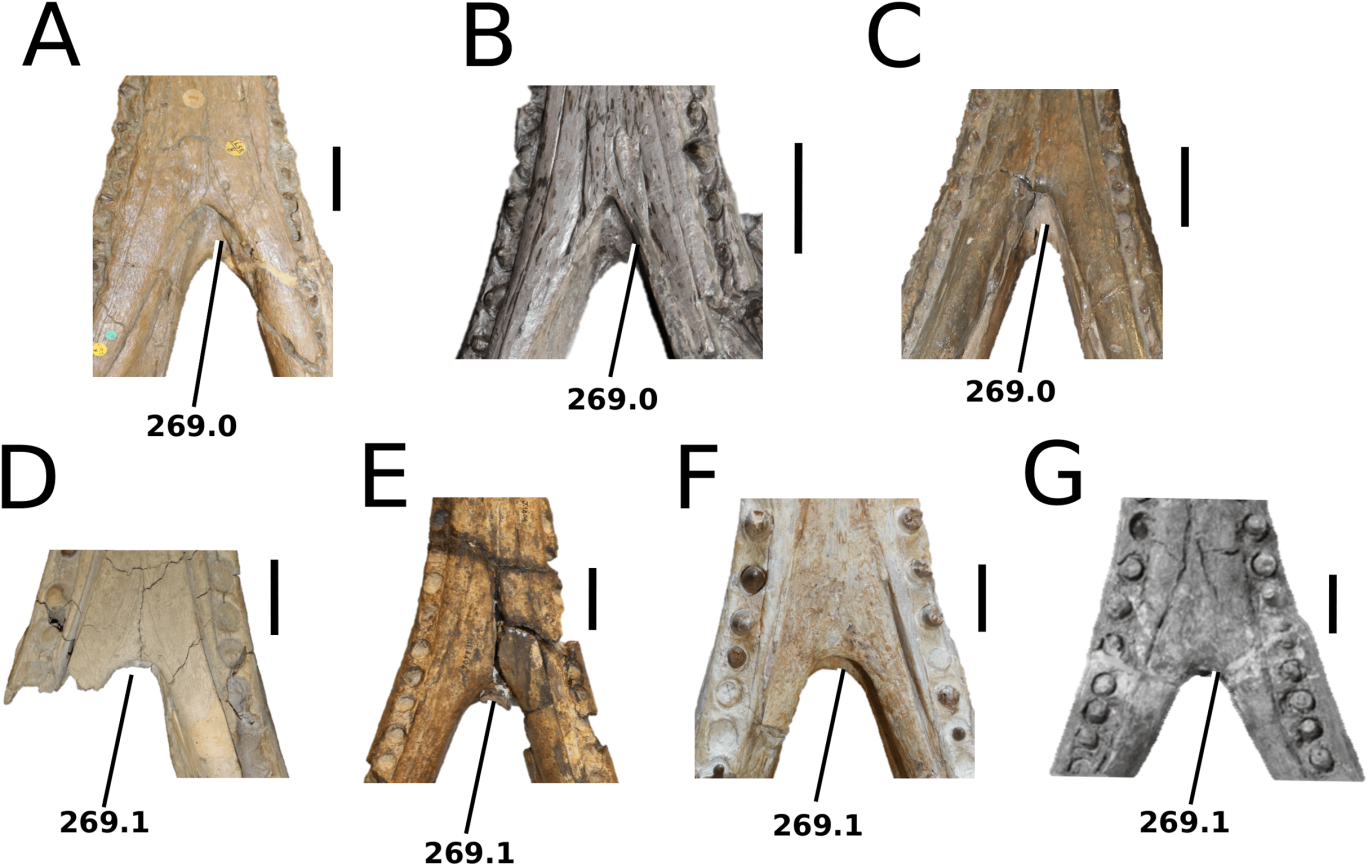
Comparative photographs: meckelian groove (= canal). Comparative photographs displaying the Meckelian groove (canal) (ch. 269) in (A) *Mycterosuchus nasutus* (NHMUK PV R 2617), (B) *Macrospondylus bollensis* (53422), (C) *Charitomenosuchus leedsi* (NHMUK PV R 3806), (D) *Steneosaurus hulkei* (= *Neosteneosaurus edwardsi*) (NHMUK PV R 2074), (E) *Yvridiosuchus boutilieri* (OUMNH J.1404), (F) *Lemmysuchus obtusidens* (LPP.M.21), and (G) *Machimosaurus mosae* (Young et al., 2014). Scale bars: 3 cm.

**269.** Splenials in dorsal view, the excavation of Meckelian groove on the dorsal surface of symphyseal splenials is deep (0) or shallow (1) (Fig. 36).

This character focuses on the excavation of the Meckelian groove (=canal) seen on the dorsal surface of the symphyseal splenials. In more basal and longirostrine teleosauroids (e.g. *Mycterosuchus*: NHMUK PV R 2617; *Macrospondylus*: SMNS 53422; *Seldsienean*: OUMNH J.1414; *Charitomenosuchus*: NHMUK PV R 3806), the Meckelian groove is anteroposteriorly long relative to jaw length and deeply excavated (state 1). In the taxa *Proexochokefalos* (MNHN.F 1890-13), *Neosteneosaurus* (NHMUK PV R 3701) and Machimosaurini (e.g. *Lemmysuchus*: LPP.M.21), the Meckelian groove is shallow with little to no excavation (state 0).

**Figure 37:**
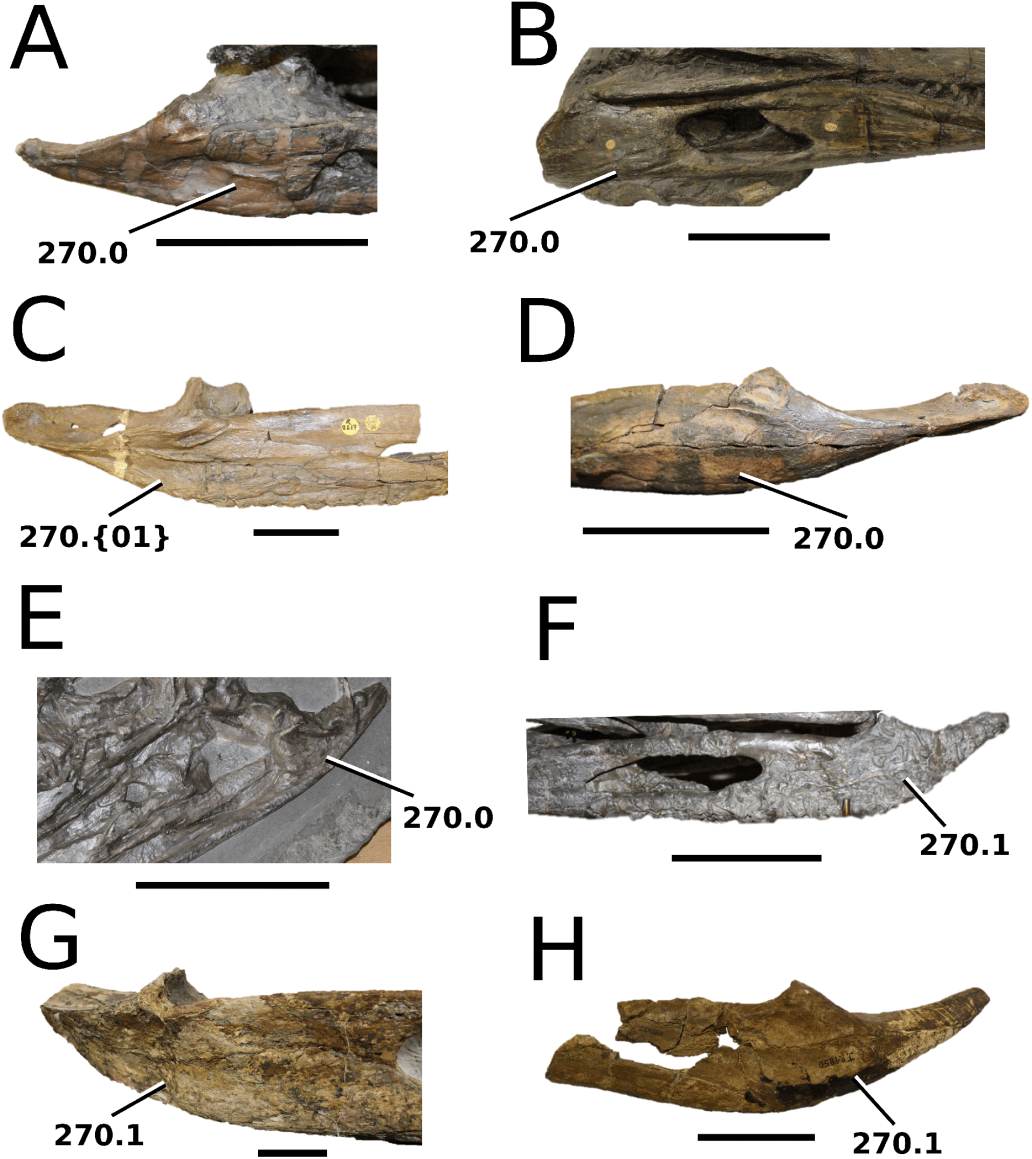
Comparative photographs: curvature of the retroarticular process. Comparative photographs displaying the curvature of the retroarticular process (ch. 270) (in lateral view). (A) *Plagiophthalmosuchus gracilirostris* (MNHNL TU515), (B) *Mystriosaurus laurillardi* (NHMUK PV OR 14781), (C) *Mycterosuchus nasutus* (NHMUK PV R 2617), (D) *Charitomenosuchus leedsi* (NHMUK PV R 3806), (E) *Macrospondylus bollensis* (SMNS 58876), (F) *Proexochokefalos heberti* (MNHN.F 1890-13), (G) *Machimosaurus buffetauti* (SMNS 91415) and (H) *Yvridiosuchus boutilieri* (OUMNH J.29850). Scale bars: 15 cm (B, E, and F) and 5 cm (A, C, D, G and H).

**270.** Angular dorsal curvature is gradual (0) or sharp and abrupt (1) (Fig. 37).

In most teleosauroids, the ventral margin of the angular gradually curves posterodorsally (state 0). This condition is seen in *Indosinosuchus* (PRC-11; PRC-239), *Platysuchus* (SMNS 9930), *Sericodon* (SCR010-1184 in Schaefer, Püntener & Billon-Bruyat, 2018), *Aeolodon* (MNHN.F.CNJ 78), *Macrospondylus* (SMNS 51753), *Charitomenosuchus* (NHMUK PV R 3806) and *Seldsienean* (OUMNH J.1414). Both *Plagiophthalmosuchus* (MNHNL TU515; NHMUK PV OR 15500) and *Mystriosaurus* (NHMUK PV OR 14781) also display state 0; however, the anterior-most angular is straight (horizontally directed), and the dorsoposterior curvature is poor and limited to the posterior area.

The curvature of the angular differs in *Proexochokefalos* (MNHN.F 1890-13), *Neosteneosaurus* (PETMG R178) and Machimosaurini (*Yvridiosuchus*: OUMNH J.29850; *Lemmysuchus*: NHMUK PV R 3168; *Machimosaurus*: IRSNB cast, SMNS 91415), in which the dorsoposterior curvature is immediate, sharp and abrupt (state 1).

**Figure 38:**
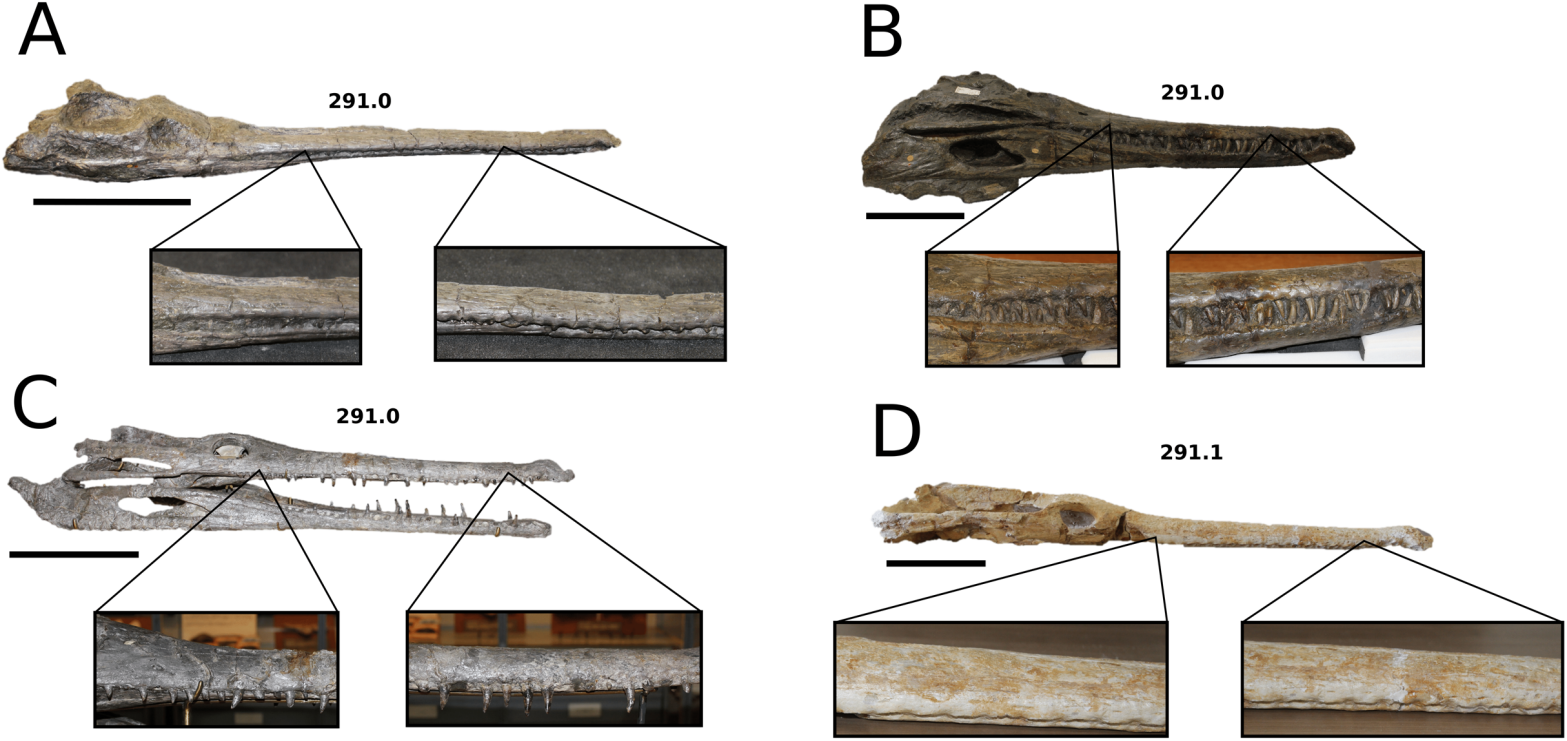
Comparative photographs: reception pits. Comparative photographs displaying the reception pits (in right lateral view) (ch. 291). (A) *Plagiophthalmosuchus gracilirostris* (NHMUK PV OR 15500), (B) *Mystriosaurus laurillardi* (NHMUK PV OR 14781), (C) *Proexochokefalos heberti* (MNHN.F 1890-13) and (D) *Lemmysuchus obtusidens* (LPP.M.21). Scale bars: 17 cm.

**291.** Maxilla, reception pits are either absent, shallow throughout, or conspicuous only in the anterior maxilla (0) or pronounced and deep throughout the entirety of the maxilla (1) (Fig. 38).

State 0 includes taxa that have either shallow or absent reception pits on the maxillae; however, it is important to note that reception pits are present in all teleosauroids, so for the purposes of this analysis, state 0 of character **291** focuses purely on taxa with shallow reception pits. These may vary substantially in terms of noticeability; for example, they are present but near invisible in the basal taxon *Plagiophthalmosuchus* (MNHNL TU515) and are relatively small and shallow, disappearing gradually, in most taxa (e.g. *Mystriosaurus*: NHMUK PV OR 14781; *Platysuchus*: SMNS 9930; *Mycterosuchus*: NHMUK PV R 2617;).

In some taxa, the reception pits are deep and noticeable throughout the near-entirety or entirety of the maxilla, notably so in the anterior and middle regions, although they do become smaller when progressing posteriorly (state 1). This condition is seen in machimosaurins (e.g. *Lemmysuchus*: NHMUK PV R 3618) as well as *Andrianavoay* (NHMUK PV R 1999), *S*. *rostromajor* (MNHN.RJN 134c-d, to some extent) and large individuals of *Neosteneosaurus* (PETMG R178).

**Figure 39:**
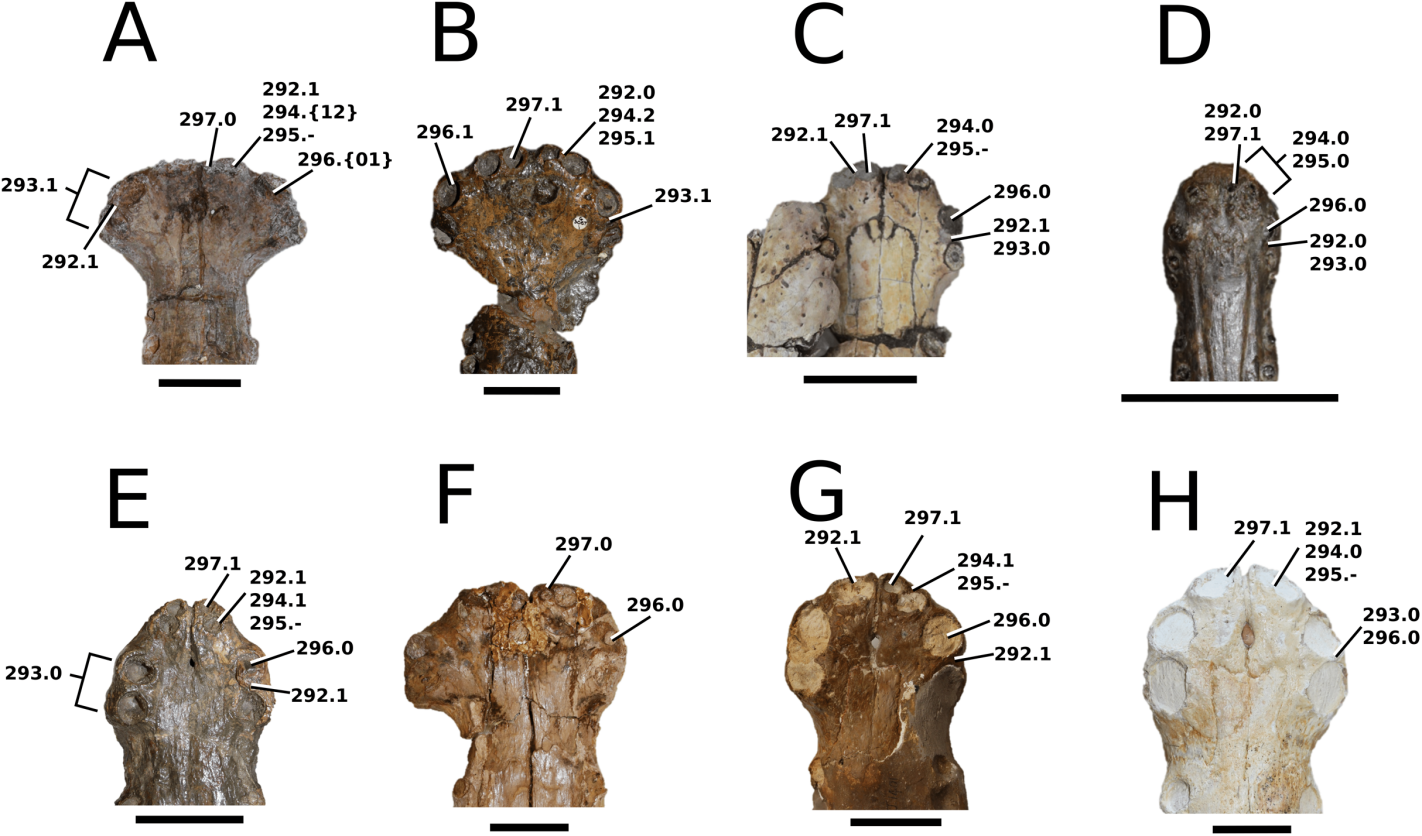
Comparative photographs: characteristic features of the premaxillary alveoli. Comparative photographs displaying characteristic features of the premaxillary alveoli (ch. 292 to 297), in: (A) the Chinese teleosauroid (IVPP V 10098), (B) *Bathysuchus megarhinus* (DORCM G.05067i; Foffa et al., 2019), (C) *Indosinosuchus potamosiamensis* (PRC-11), (D) *Platysuchus multiscrobiculatus* (MNHNL. TU895), (E) *Charitomenosuchus leedsi* (NHMUK PV R 3806), (F) *Mystriosaurus* sp. (SNHM-IG-008-R), (G) *Yvridiosuchus boutilieri* (OUMNH J.1401) and (H) *Lemmysuchus obtusidens* (LPP.M.21). Note that character 294 and 295 are inapplicable for the Chinese teleosauroid (IVPP V 10098). Scale bars: 3 cm.

**292.** Premaxilla, P1-P2 either does not form a couplet and the interalveolar spacing between P1-P2 and P3-P4 relatively the same size (0) or forms a couplet with the interalveolar spacing between P1-P2 and P3-P4, with P1-P2 being separated by a thin lamina and P3-P4 being well separated (1) (Fig. 39).

The first (P1) and second (P2) premaxillary alveoli are situated anterior to the third (P3) and fourth (P4), which are positioned posterolaterally. The fifth (P5) premaxillary alveolus (present in *Bathysuchus*, *Sericodon* and *Platysuchus*) is positioned dorsally in comparison to the P1 to P4 (Foffa et al., 2019). As such, the interalveolar distance varies between these alveoli. The P1 and P2 can be well separated in a way similar to that between the P3 and P4; the interalveolar spacing is large and noticeable, with the adjacent alveoli at a further distance from one another. This condition (state 0) occurs in *Platysuchus* (MNHNL TU895), *Sericodon* (SCR011-406 in Schaefer, Püntener & Billon-Bruyat, 2018), *Bathysuchus* (DORCM G.05067i) and *Mycterosuchus* (CAMSM J.1420).

In contrast, in the majority of teleosauroids the P3 and P4 remain separate, but the P1 and P2 are situated closely together and are either separated by a small, thin interalveolar lamina, or appear slightly merged together, thereby creating a P1-P2 ‘couplet’ (state 1). This state is seen in *Mystriosaurus* (NHMUK PV OR 14781), the Chinese teleosauroid (IVPP V 10098), *I. potamosiamensis* (PRC-11) and one subclade of teleosauroids (e.g. *Macrospondylus* SMNS 18672; *Charitomenosuchus*: NHMUK PV R 3806; *Proexochokefalos*: MNHN.F 1890-13; *Lemmysuchus*: NOTNH FS3361). Note that this character is not applicable for taxa that have fewer than four premaxillary alveoli (*Machimosaurus*).

**293.** Premaxilla, P3-P4 couplet is present (0) or absent (1) (Fig. 39).

In most teleosauroids, the interalveolar spacing is generally noticeable and well-developed between the P3 and the P4, but it is usually small (possibly due to both alveoli being quite large); the alveoli are therefore closely spaced together, forming a couplet (state 0). This is present in most teleosauroids (e.g. *Mystriosaurus*: NHMUK PV OR 14781; *Platysuchus*: MNHNL TU895; *Mycterosuchus*: CAMSM J.1420; *Macrospondylus* SMNS 81699; *Proexochokefalos*: MNHN.F 1890-13; *Lemmysuchus*: NOTNH FS3361). State 1 is found in both *Bathysuchus* (NHMUK PV OR 43086, DORCM G.05067i) and the Chinese teleosauroid (IVPP V 10098), in which the P3-P4 are widely spaced apart from one another, and therefore do not form a couplet. Note that this character is not applicable for taxa that have fewer than four premaxillary alveoli *(Machimosaurus*).

**294.** Premaxilla in palatal view, both P1 and P2 are oriented anteriorly (0), P1 is oriented anteriorly and P2 slightly medially (1), or both P1 and P2 are oriented laterally (2) (Fig. 39).

In many teleosauroids, both the P1 and P2 are oriented anteriorly (state 0). This occurs in *Mystriosaurus* (NHMUK PV OR 14781), *I. potamosiamensis* (PRC11), *Platysuchus* (MNHNL TU895), *Macrospondylus* (SMNS 18672), *Deslongchampsina* (OUMNH J.29851), *Neosteneosaurus* (NHMUK PV R 28650), *Yvridiosuchus* (OUMNH J.1401) and *Lemmysuchus* (NOTNH FS3361). In a second condition (state 1), the P1 is oriented anteriorly, but the P2 is oriented slightly medially. This is seen in *Charitomenosuchus* (NHMUK PV R 3806) and *Proexochokefalos* (MNHN.F 1890-13). A third condition (state 2), which occurs in *Bathysuchus* (Foffa et al., 2019), *Sericodon* (SCR011-406 in Schaefer, Püntener & Billon-Bruyat, 2018) and *Mycterosuchus* (CAMSM J.1420), is that the P1 and P2 are both strongly oriented laterally, appearing almost horizontally placed. Note that this character is not applicable for taxa that have fewer than four premaxillary alveoli (*Machimosaurus*).

**295.** Premaxilla, both P1 and P2 do not form a couplet and are either not oriented on the anterior margin of the premaxilla (0) or are oriented on the anterior margin of the premaxilla (1) (Fig. 39).

In certain teleosauroids, if the P1-P2 alveolar complex does not form a couplet, these two alveoli are positioned either on or slightly ventral to the anterior margin of the premaxilla. In *Platysuchus* (SMNS 9930), the P1 and P2 do not form such a couplet and both alveoli are not oriented on the anterior margin of the premaxilla (state 0). However, in the genera *Bathysuchus* (DORCM G.05067i, unnumbered LPP specimen), *Sericodon* (SCR011-406 in Schaefer, Püntener & Billon-Bruyat, 2018) and *Mycterosuchus* (CAMSM J.1420), the P1 and P2 do not form a couplet but are noticeably oriented on the anterior margin of the premaxilla (state 1). Note that this character is not applicable for taxa that have fewer than four premaxillary alveoli (*Machimosaurus*).

**296.** Premaxilla with no strong lateral expansion (0) or strong lateral expansion so that P3 and P4 are aligned on the lateral plane of the external margin, more so than P2 (1) (Fig. 39).

In most teleosauroids, the P3 and P4 are positioned posteriorly to the P1 and P2 and are aligned on a vertical plane of the lateral margin, whereas the P1 and P2 are aligned more laterally,, due to little or no lateral expansion of the premaxillae (state 0). This condition can be clearly seen in *Plagiophthalmosuchus* (NHMUK PV OR 14792), more basal teleosauroids (e.g. *Mystriosaurus*: NHMUK PV OR 14781; *Platysuchus*: MNHNL TU895), and in more derived teleosauroids (e.g. *Charitomenosuchus*: NHMUK PV R 3806; *Proexochokefalos*: MNHN.F 1890-13; *Lemmysuchus*: LPP.M.21). In select taxa, the premaxillae are laterally expanded, with the P3 and P4 aligned on a different plane (state 1). This occurs in *Bathysuchus* (DORCM G.05067i; unnumbered LPP specimen) and *Sericodon* (Schaefer, Püntener & Billon-Bruyat, 2018).

**297.** Premaxilla, very small first premaxillary alveolus with the second premaxillary alveolus being much larger (0) or the first and second premaxillary alveoli are relatively the same size (1) (Fig. 39).

In most teleosauroids, the size of the P1 and P2 are relatively the same, with both being slightly smaller than the P3 and P4 (which is often the largest, as it houses the large fourth premaxillary tooth) (state 1). This condition is observed in *I. potamosiamensis* (PRC-11), *Mycterosuchus* (CAMSM J.1420), *Bathysuchus* (DORCM G.05067i), *Deslongchampsina* (OUMNH J.29851), *Charitomenosuchus* (NHMUK PV R 3806), *Proexochokefalos* (MNHN.F 1890-13), *Neosteneosaurus* (NHMUK PV R 2865), *Yvridiosuchus* (OUMNH J.1401) and *Lemmysuchus* (LPP.M.21). In certain teleosauroids, the P1 is considerably smaller than the P2, with the P1 being 25% or less the size of the P2 (state 0). This condition is observed in the Chinese teleosauroid (IVPP V 10098) and *Macrospondylus* (SMNS 81699).

**Figure 40:**
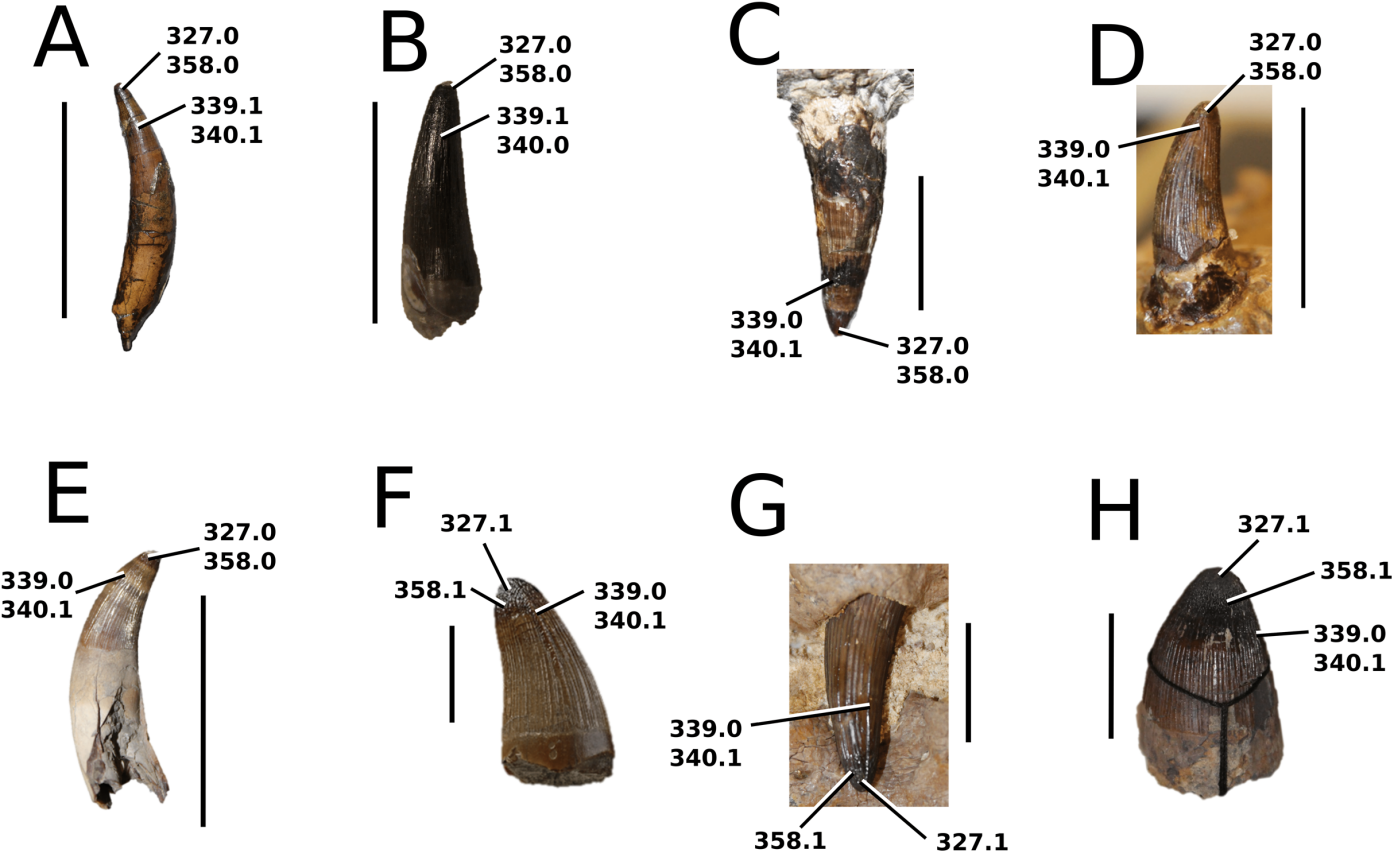
Comparative photographs: teleosauroid teeth. Comparative photographs of teleosauroid teeth, highlighting the carinae (ch. 339–340), apices (ch. 327) and anastomosing pattern (ch. 358): (A) *Bathysuchus megarhinus* (DORCM G.05067iv; Foffa et al., 2019), (B) *Sericodon jugleri* (NRM-PZ 2337), (C) *Proexochokefalos heberti* (MNHN.F 1890-13), (D) *Deslongchampsina larteti* (OUMNH J.29851), (E) *Neosteneosaurus edwardsi* (NHMUK PV R 2865), (F) Machimosaurini indeterminate (GPIT-RE-301), (G) *Yvridiosuchus boutilieri* (OUMNH J.29850), and (H) *Machimosaurus hugii* (MG 25). Scale bars: 3 cm (A, B and E) and 1 cm (C, D, F–H).

**339.** Dentition, carinae on the apical third of a tooth are present and well pronounced (0) or absent/weakly pronounced (1) (Fig. 40).

All known teleosauroids possess carinae (excluding the Chinese teleosauroid IVPP V 10098, *Andrianavoay* NHMUK PV R 1999, *Clovesuurdameredeor* NHMUK PV OR 49126 and *P*. cf. *bouchardi* (Lepage et al., 2008), as none have any teeth preserved); in addition, most teleosauroids have carinae that extend the entire apicobasal length of the tooth, (state 0). These is seen in the basal form *Plagiophthalmosuchus* (MNHNL TU515) and *Mystriosaurus* (NHMUK PV OR 14781), *I. kalasinensis* (PRC-239), *Mycterosuchus* (NHMUK PV R 2617), *Aeolodon* (MNHN.F.CNJ 78) *Charitomenosuchus* (NHMUK PV R 3806), *Proexochokefalos* (MNHN.F 1890-13) *Seldsienean* (OUMNH J.1414), *Neosteneosaurus* (PETMG R178), *Lemmysuchus* (NHMUK PV R 3168) and *Mac. hugii* (MG8730-1). However, two taxa (*Bathysuchus*: DORCM G.05067iv; *Sericodon*: TCH005-151 in Schaefer, Püntener & Billon-Bruyat, 2018) have carinae that only extend two-thirds the apicobasal length of the tooth, from the base to the apex and are absent at the apex (state 1).

**340.** Dentition, enamel ridges on the apical third of a tooth are absent (0) or present (1) (Fig. 40).

In teleosauroids, the enamel ridges are either faint and/or difficult to see (e.g. *Plagiophthalmosuchus*: MNHNL TU515), or noticeable and well-developed (e.g. *Mycterosuchus*: NHMUK PV R 2617). Enamel ridges are present on the entirety of the crown, including the apex (state 1) in the basal-most form *Plagiophthalmosuchus* (MNHNL TU515), along with most teleosauroids (e.g. *Mystriosaurus*: NHMUK PV OR 14781; *Mycterosuchus*: NHMUK PV R 2617; *Bathysuchus*: DORCM G.05067iv; 53422; *Charitomenosuchus*: NHMUK PV R 3806; *Seldsienean*: OUMNH J.1414; *Deslongchampsina*: OUMNH J.29851; machimosaurins: NHMUK PV R 3168; NHMW 1846.III.208). Only in one confirmed taxon, *Sericodon* (TCH005-151 in Schaefer, Püntener & Billon-Bruyat (2018)), are the enamel ridges absent from the apex (state 0).

**Figure 41:**
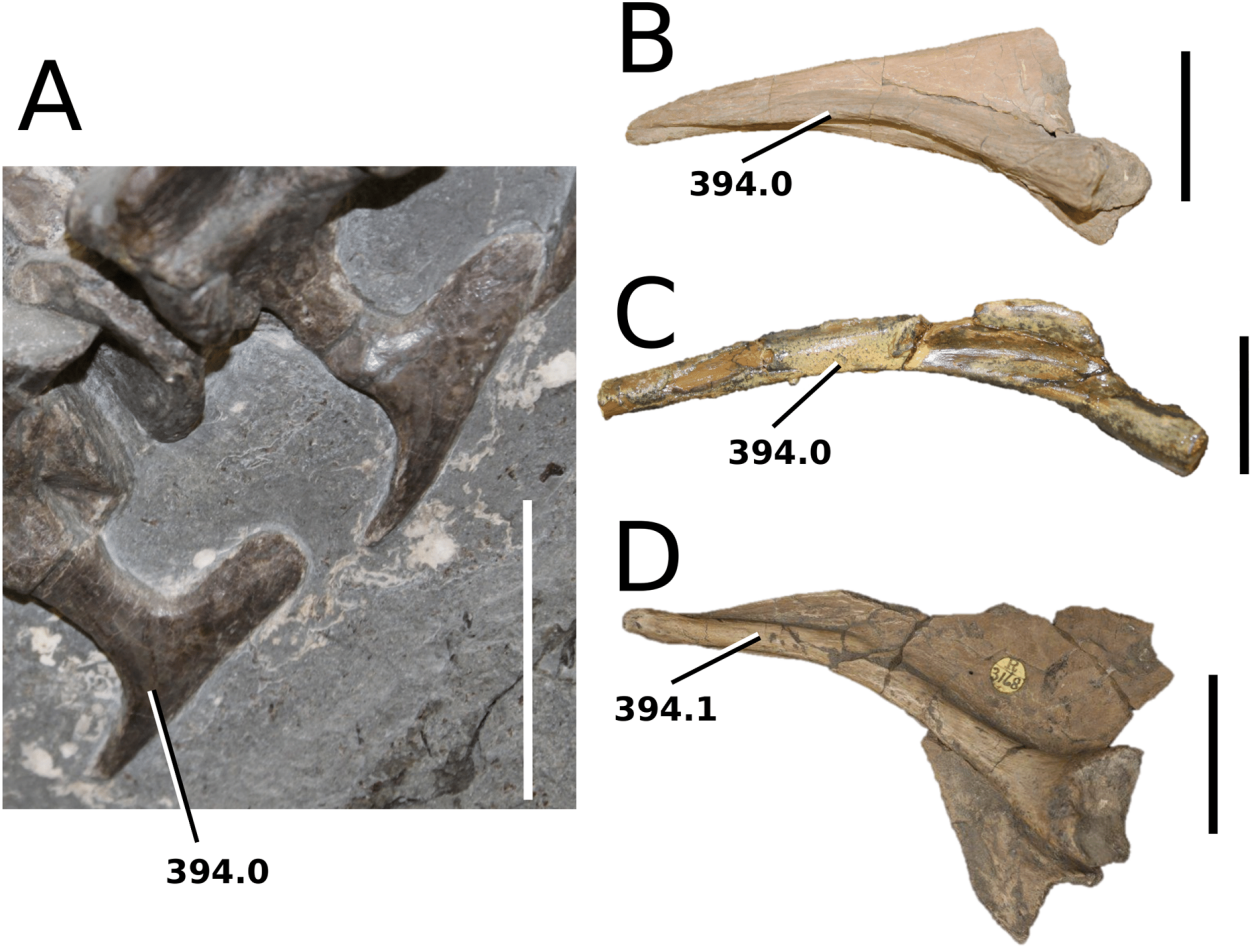
Comparative photographs: teleosauroid cervical ribs. Comparative photographs of teleosauroid cervical ribs (ch. 394): (A) *Macrospondylus bollensis* (SMNS 51984), (B) *Mycterosuchus nasutus* (NHMUK PV R 2617), (C) *Neosteneosaurus edwardsi* (NHMUK PV R 3701) and (D) *Lemmysuchus obtusidens* (NHMUK PV R 3168). Scale bars: 3 cm.

**394.** Cervical ribs in lateral view, the anteroposterior ridge of large, more posteriorly placed cervical ribs is straight (0) or dorsoventrally curved (1) (Fig. 41).

Most teleosauroids that can be scored for this character exhibit T-shaped (in dorsal view) cervical ribs where the anteroposterior ridge is horizontal or straightened (state 0)(*Platysuchus* : SMNS 9930); *Mycterosuchus*: NHMUK PV R 2617; *Charitomenosuchus*: NHMUK PV R 3806. However, in *Lemmysuchus* (NHMUK PV R 3168), the largest, most posteriorly placed cervical ribs have a distinct dorsomedial curvature along the anteroposterior ridge, appearing slightly concave in lateral view (state 1).

**Figure 42:**
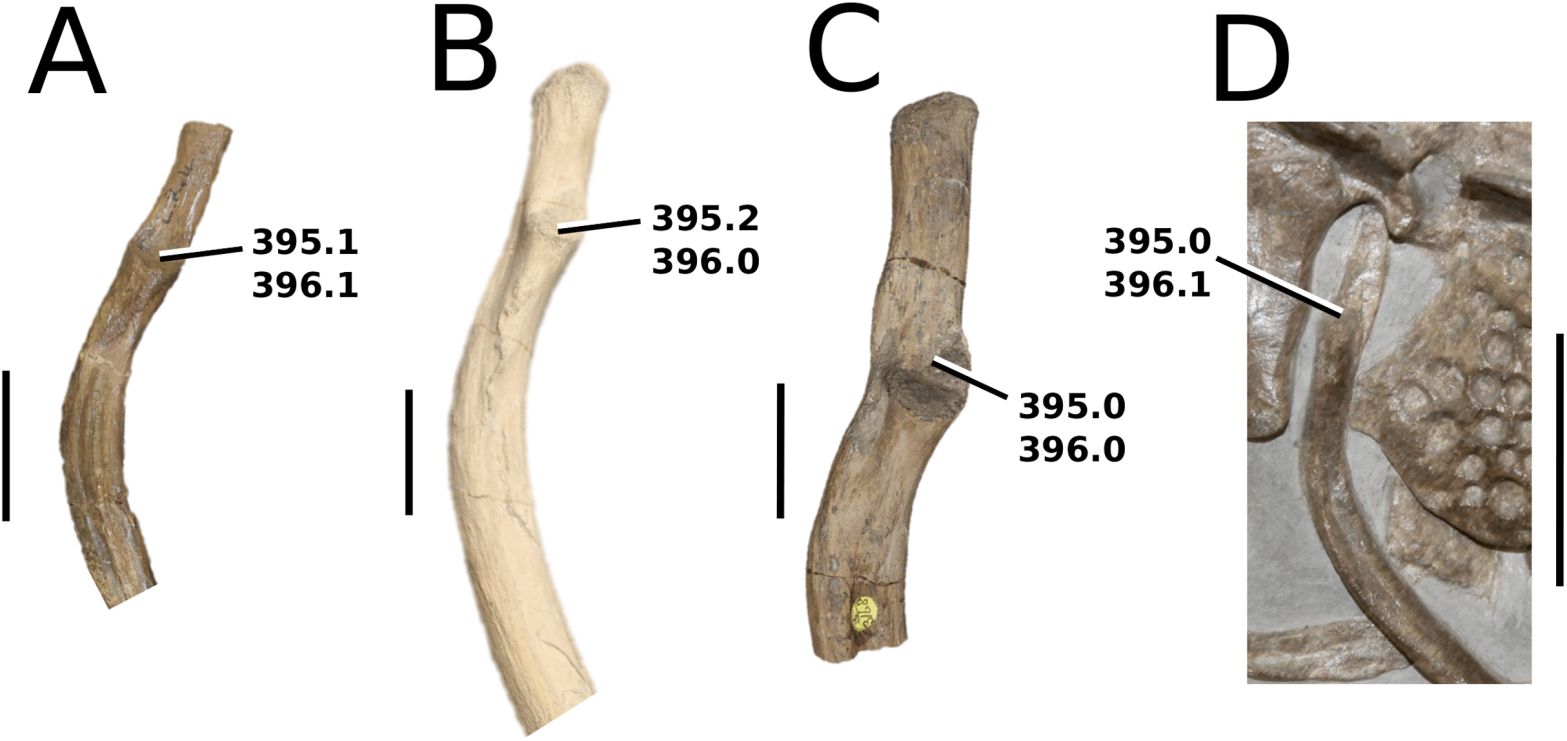
Comparative photographs: teleosauroid dorsal ribs. Comparative photographs of teleosauroid dorsal ribs (ch. 395 and 396) (from the middle of the ribcage); (A) *Charitomenosuchus leedsi* (NHMUK PV R 3806), (B) *Neosteneosaurus edwardsi* (PETMG R178), (C) *Lemmysuchus obtusidens* (NHMUK PV R 3168) and (D) *Macrospondylus bollensis* (SMNS 52034). Scale bars: 3 cm.

**395.** Dorsal ribs, the positioning of both the tuberculum and articular facet is on the medial edge (0), directly in the middle (1), or on the lateromedial edge (2) (Fig. 42).

In most teleosauroids with preserved dorsal ribs, both the tuberculum and articular facet are positioned on the medial edge of the rib (state 0). This is observed in *Platysuchus* (SMNS 9930), *Macrospondylus* (SMNS 51753, SMNS 18672), *Aeolodon* (MNHN.F.CNJ 78) and *Lemmysuchus* (NHMUK PV R 3168). In two taxa (*Mycterosuchus*: NHMUK PV R 2617; *Charitomenosuchus*: NHMUK PV R 3806), the tuberculum and articular facets have shifted laterally and are placed directly in the middle of the rib (state 1). In *Neosteneosaurus* (NHMUK PV R 3701, PETMG R178), the tuberculum and articular facets have shifted even further laterally so that they are positioned on the lateromedial edge of the rib (state 2).

**396.** Dorsal ribs in lateral view, the tuberculum is pronounced (0) or weak (1) (Fig. 42).

In *Mycterosuchus* (NHMUK PV R 2617), *Neosteneosaurus* (PETMG R178), *Lemmysuchus* (NHMUK PV R 3168) and *Mac. buffetauti* (SMNS 91415), the tuberculum is well-developed and pronounced, as large as the capitulum and anteroposteriorly elongated, giving it an oval shape (state 0). In certain taxa (*Sericodon*: Schaefer, Püntener & Billon-Bruyat, 2018; *Aeolodon*: MNHN.F.CNJ 78; *Macrospondylus*: SMNS 51753; *Charitomenosuchus*: NHMUK PV R 3806), the tuberculum is reduced, small and circular in shape (state 1).

**Figure 43:**
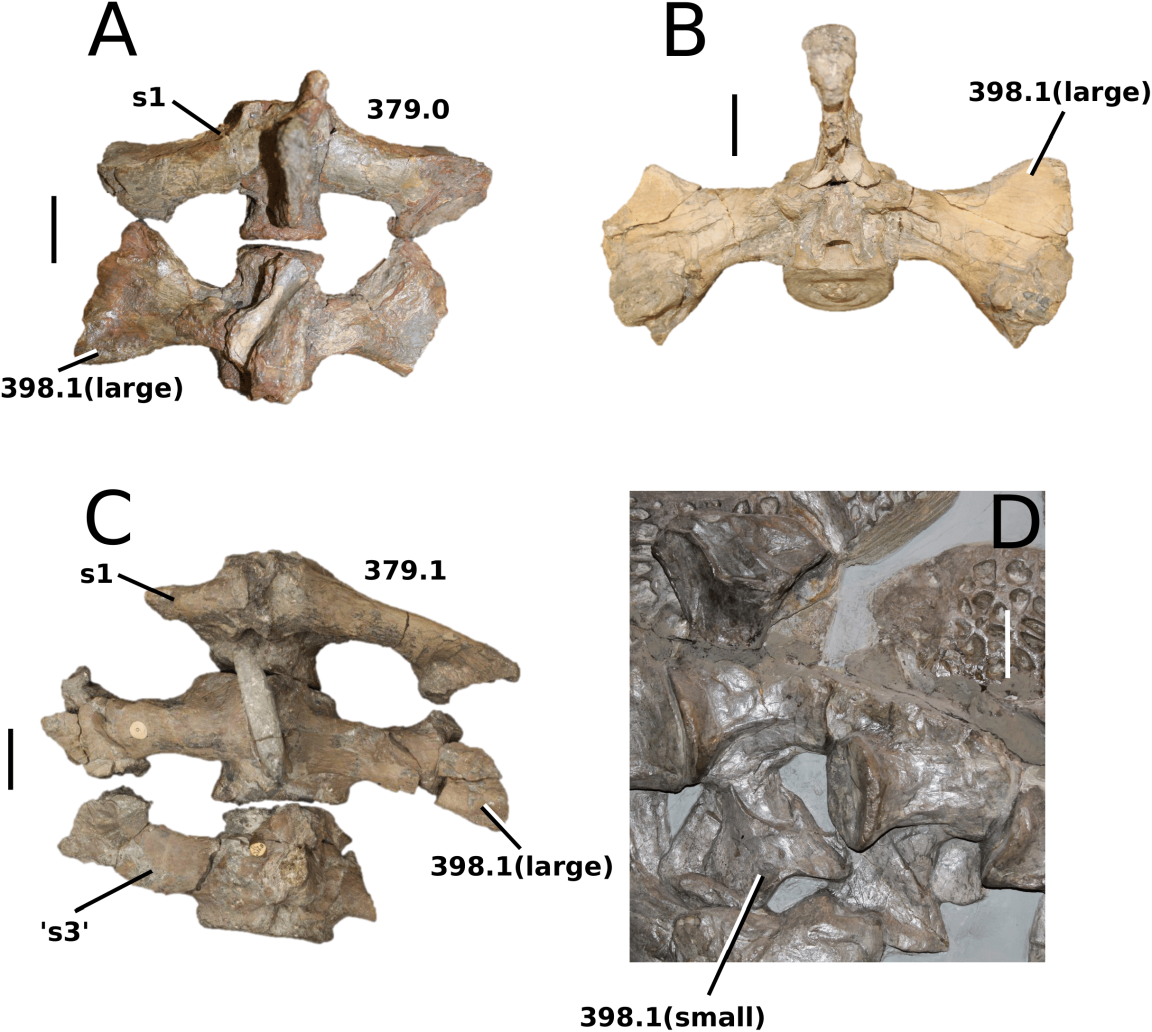
Comparative photographs: teleosauroid sacral vertebrae. Comparative photographs of teleosauroid sacral vertebrae, with special attention to the number (ch. 379) and flange of the second sacral (ch. 398): (A) *Charitomenosuchus leedsi* (NHMUK PV R 3806), (B) *Lemmysuchus obtusidens* (NHMUK PV R 3168), (C) *Mycterosuchus nasutus* (NHMUK PV R 2617) and (D) *Macrospondylus bollensis* (GPIT-RE-9427).

**398.** Second sacral vertebrae, the anterior margin of the posterior area of the second sacral vertebra has either a small, non-expanding flange (0) or a large, expanded and projecting flange (1) (Fig. 43).

In crocodylomorphs, the posterior area of the second sacral vertebra has an anterior margin that is both anteroposteriorly and dorsoventrally expanded into a projection or ‘flange’ of bone, which allows for a secure attachment to the ilium, thus influencing body movement. This ‘flange’ is either small and non-expanding (state 0), or noticeably expanded and anteroposteriorly protruding (state 1). All scored teleosauroids exhibit state 1, as there is always an expanded flange present on the anterior margin; however, the size and development differ. In the taxa *Mycterosuchus* (NHMUK PV R 2617), *Charitomenosuchus* (NHMUK PV R 3806), *Lemmysuchus* (NHMUK PV R 3168) and *Mac. mosae* (Hua, 1999; Young et al., 2014), the flange is considerably larger, more pronounced and well-developed. In *Macrospondylus* (MMG BwJ 595) and *Neosteneosaurus* (NHMUK PV R 3701) the flange is still present, but it is much smaller and less obvious.

**Figure 44:**
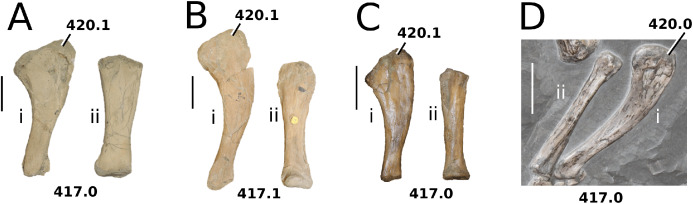
Comparative photographs: teleosauroid ulnae and radiae. Comparative photographs of teleosauroid ulnae and radiae, with special attention to relative size (ch. 417) and proximal ulna (ch. 420): (A) *Neosteneosaurus edwardsi* (PETMG R178) (i) ulna and (ii) radius; (B) *Mycterosuchus nasutus* (NHMUK PV R 2617) (i) ulna and (ii) radius; (C) *Charitomenosuchus leedsi* (NHMUK PV R 3806) (i) ulna and (ii) radius; and (D) *Macrospondylus bollensis* (SMNS 53422) (i) ulna and (ii) radius. Scale bars: 3 cm.

**417.** Radius and ulna, the same length (0) or the ulna is longer (1) (Fig. 44).

In the majority of teleosauroids, the radius and ulna are approximately the same size (Andrews, 1913), with the ulna being marginally longer (state 0); this is seen in taxa such as *Platysuchus* (SMNS 9930), *Aeolodon* (MNHN.F.CNJ 78), *Macrospondylus* (SMNS 51563, SMNS 53422), *Charitomenosuchus* (NHMUK PV R 3608), *Neosteneosaurus* (PETMG R178) and *Lemmysuchus* (NHMUK PV R 3168). However, in the genus *Mycterosuchus* (NHMUK PV R 2617) the ulna is roughly 18% longer than the radius (state 1), which is unusual.

**Figure 45:**
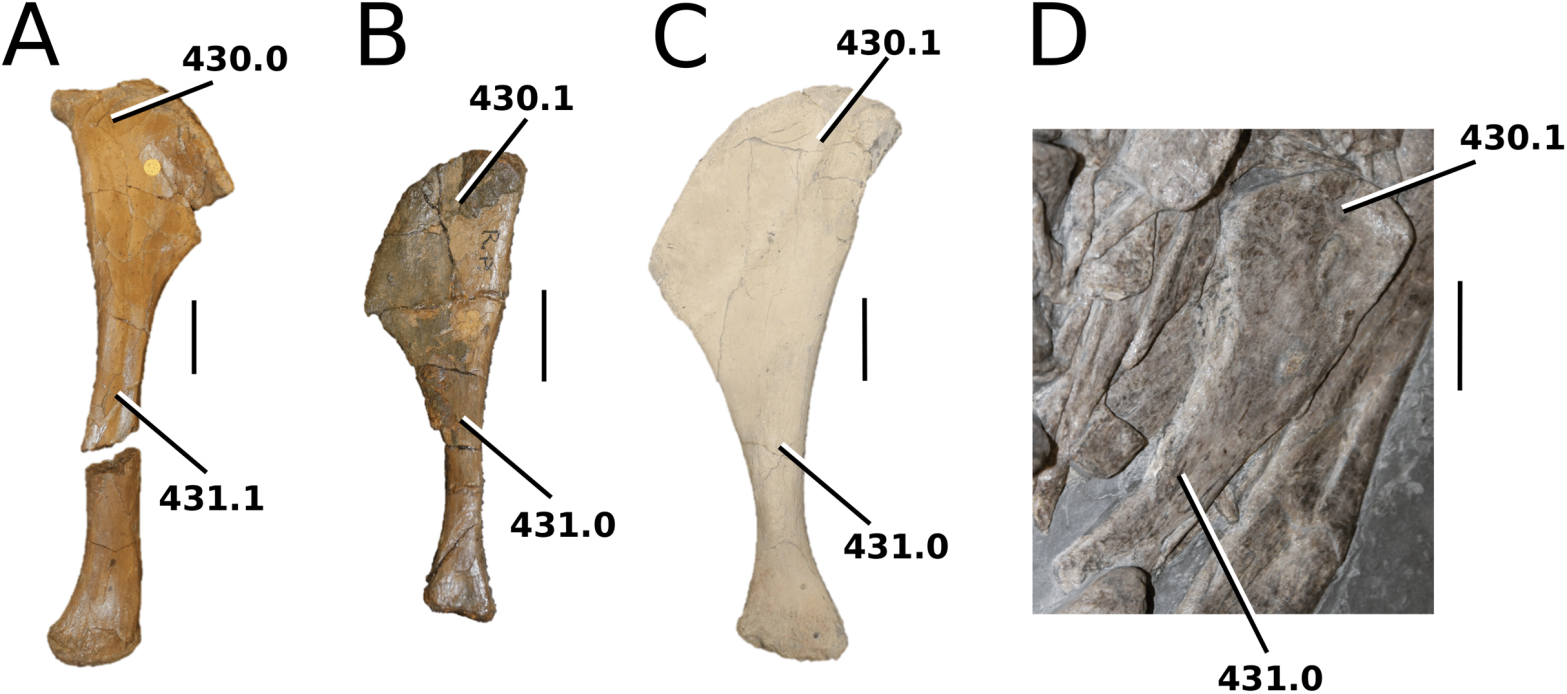
Comparative photographs: teleosauroid pubes. Comparative photographs of teleosauroid pubes, highlighting the pubic blade (ch. 430) and elongation (ch. 431): (A) *Mycterosuchus nasutus* (NHMUK PV R 2617), (B) *Charitomenosuchus leedsi* (NHMUK PV R 3806), (C) *Neosteneosaurus edwardsi* (PETMG R178) and (D) *Macrospondylus bollensis* (SMNS 51957). Scale bars: 3 cm.

**430.** Pubis, the shape of distal rim of distal pubic blade is straight and square-like (0) or curved and rounded (1) (Fig. 45).

In most scored teleosauroids, the ventral (distal) margin of the pubic blade is anteriorly curved and rounded in lateral view (state 1). This is the case in *Charitomenosuchus* (NHMUK PV R 3806), *Macrospondylus* (SMNS 51957), *Neosteneosaurus* (PETMG R178), *Lemmysuchus* (NHMUK PV R 3168) and *Mac. mosae* (Hua, 1999; Young et al., 2014). However, in two taxa the distal rim of the pubic blade is straightened and relatively square-like (state 0): *Mycterosuchus* (NHMUK PV R 2617) and *Platysuchus* (SMNS 9930).

**431.** Pubis, the pubic shaft is shorter (0) or longer (1) than the pubic blade (Fig. 45).

In most teleosauroid taxa, the pubic shaft is either approximately the same length or slightly anteroposteriorly shorter than the pubic blade (state 0). This is the condition seen in six scored teleosauroids: *Macrospondylus* (SMNS 51957), *Charitomenosuchus* (NHMUK PV R 3806), *Lemmysuchus* (NHMUK PV R 3168), *Mac. mosae* (Hua, 1999), *Platysuchus* (SMNS 9930) and *Sericodon* (SCR010-312 in Schaefer, Püntener & Billon-Bruyat, 2018). However, the pubic shaft is significantly longer (over 50%) than the pubic blade (state 1) in one taxon (*Mycterosuchus*: NHMUK PV R 2617) and represents an apomorphic trait of this genus.

**Figure 46:**
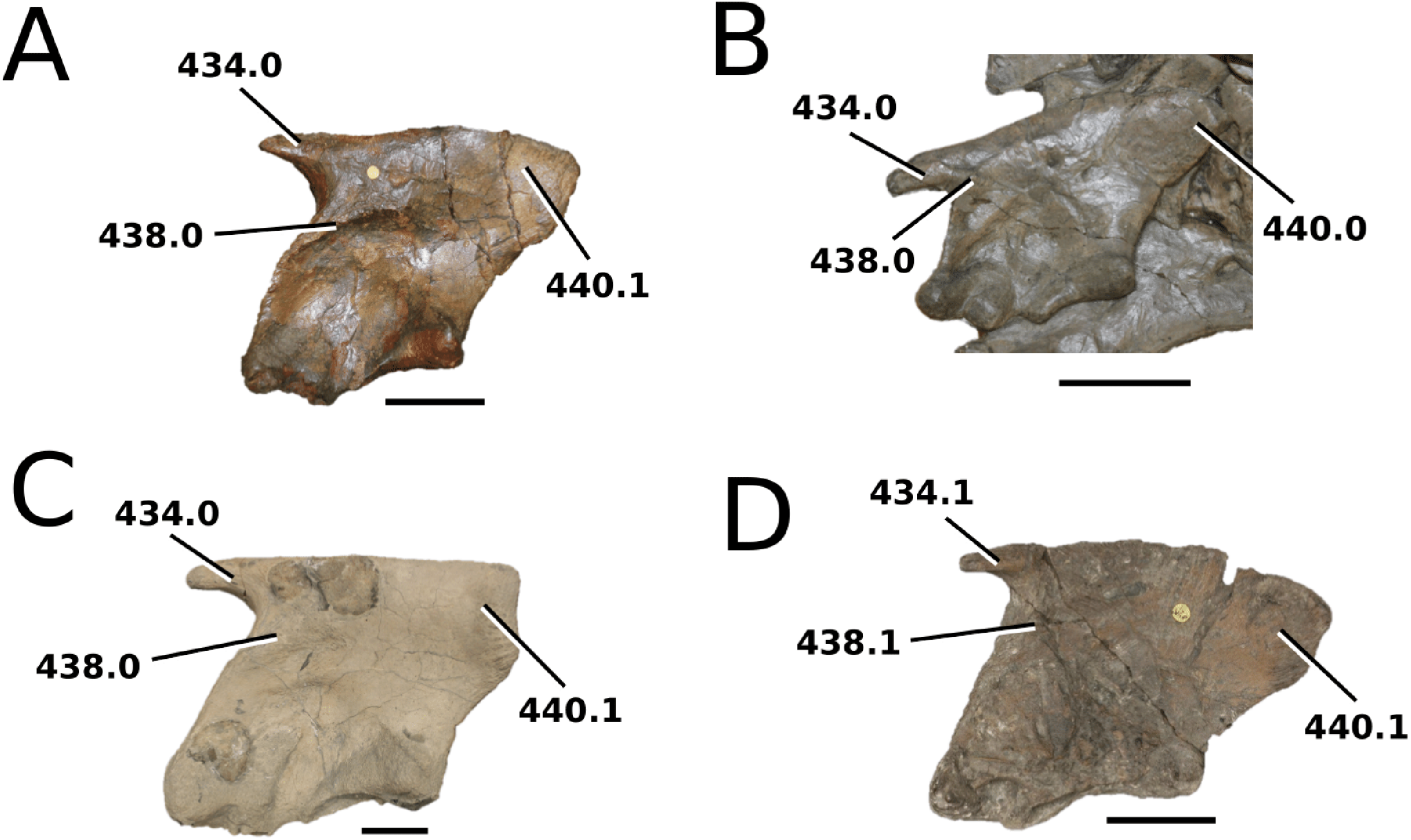
Comparative photographs: teleosauroid ilia. Comparative photographs of teleosauroid ilia with attention to the anterior process (ch. 434), supraacetabular crest (ch. 438) and postacetabular process (ch. 440): (A) *Charitomenosuchus leedsi* (NHMUK PV R 3806), (B) *Macrospondylus bollensis* (SMNS 18672), (C) *Neosteneosaurus edwardsi* (PETMG R178) and (D) *Lemmysuchus obtusidens* (NHMUK PV R 3168). Scale bars: 5 cm.

**434.** Ilium, the anterior iliac process is long and slender (0), or short and robust (1) (Fig. 46).

In most teleosauroids, the anterior iliac process is anteroposteriorly elongated, mediolaterally slender, and straight with little to no curvature (state 0). This is seen in *Platysuchus* (SMNS 9930), *Teleosaurus* (NHMUK PV R 1782a), *Sericodon* (SCR010-312 in Schaefer, Püntener & Billon-Bruyat, 2018), *Aeolodon* (MNHN.F.CNJ 78), *Macrospondylus* (MMG BwJ 565), *Charitomenosuchus* (NHMUK PV R 3806; Andrews, 1913) and *Neosteneosaurus* (PETMG R178). In contrast, state 1 describes the anterior process as anteroposteriorly shortened, robust and chunky in appearance, with a slight lateral curvature. This morphology is present in the machimosaurins *Lemmysuchus* (NHMUK PV R 3168) and *Mac. mosae* (Hua, 1999; Young et al., 2014), as well as the basal metriorhynchoid *Pelagosaurus* (MNHN.RJN 463) and members of Metriorhynchidae (e.g. *Tyrannoneustes lythrodectikos* Young et al., 2013; *Cricosaurus lithographicus*; *Cricosaurus araucanensis* (Herrera, Fernández & Gasparini, 2013); Fraas, 1902; Andrews, 1913).

**438.** Supraacetabular iliac crest is pronounced (0) or shallow and poorly developed (1) in medial view (Fig. 46).

In non-machimosaurins (e.g. *Plagiophthalmosuchus*: NHMUK PV OR 14792; *Platysuchus*: SMNS 9930; *Charitomenosuchus*: NHMUK PV R 3806; *Neosteneosaurus*: NHMUK PV R 3701, PETMG R178) the supraacetabular crest is enlarged and pronounced, jutting out laterally and slightly overhanging the acetabulum (state 0). In state 1, the supraacetabular crest is poorly developed, with either shallow or no outward projection. This is the case in the machimosaurins *Lemmysuchus* (NHMUK PV R 3168; Johnson et al., 2017) and *Mac. mosae* (Hua, 1999).

**Figure 47:**
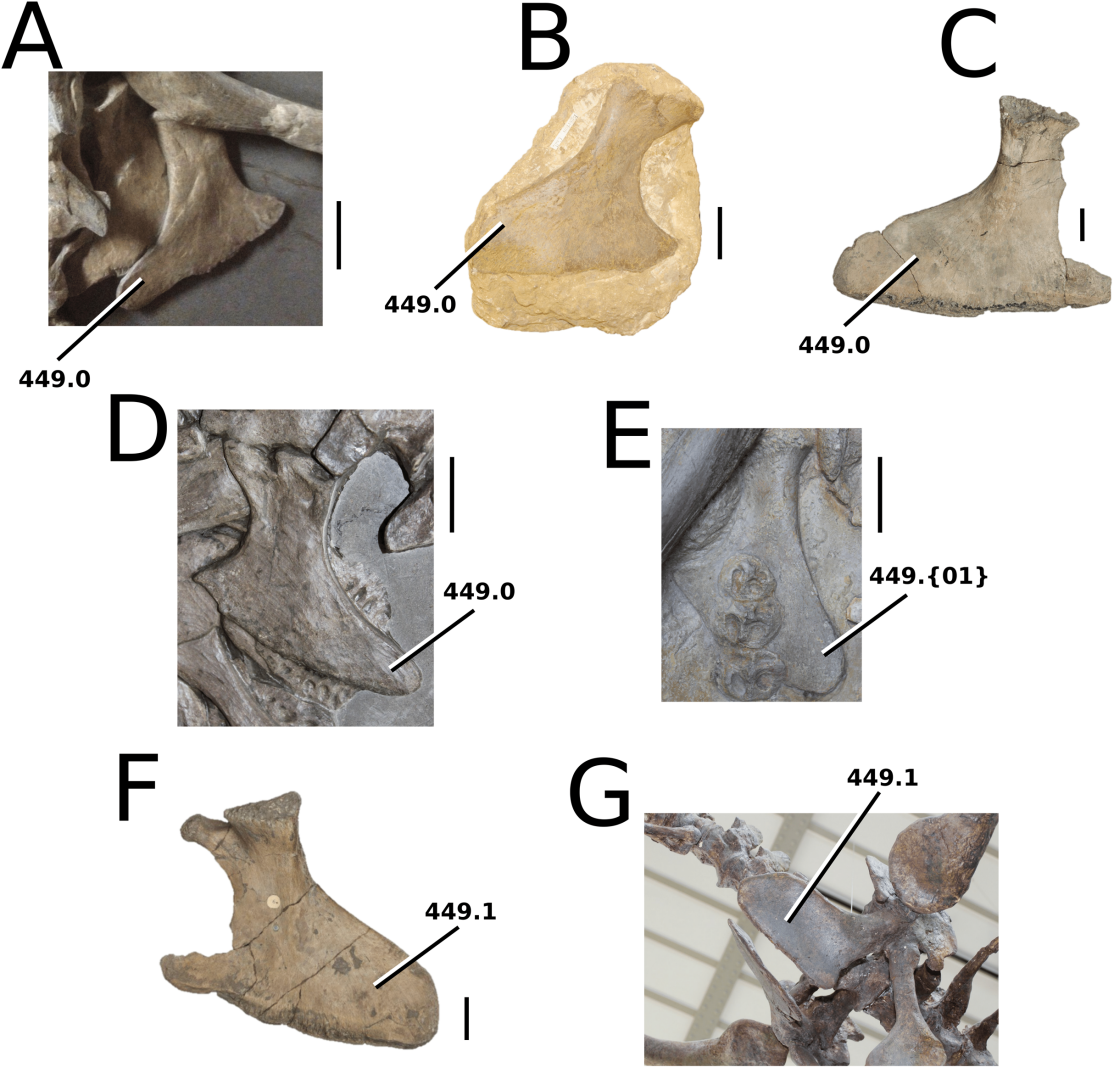
Comparative photographs: teleosauroid ischia. Comparative photographs of teleosauroid ischia with emphasis on the ischial blade (ch. 449): (A) *Platysuchus multiscrobiculatus* (SMNS 9930), (B) *Teleosaurus* sp. (NHMUK PV 238), (C) *Neosteneosaurus edwardsi* (NHMUK PV R 3898), (D) *Macrospondylus bollensis* (SMNS 58876), (E) *Aeolodon priscus* (MNHN.F.CNJ 78), (F) *Lemmysuchus obtusidens* (NHMUK PV R 3168) and (G) *Machimosaurus mosae* (IRSNB cast). Scale bars: 3 cm, (H) not to scale.

**449.** Ischium, the posteroventral margin of ischial blade is triangular (0) or sub-square (1) (Fig. 47).

In most teleosauroids, the ischial blade is gracile, mediolaterally thin and anteroposteriorly elongated, with the posteroventral margin having a triangular-like shape (state 0). This morphology is present in *Platysuchus* (SMNS 9930), *Teleosaurus* (NHMUK PV R 1638), *Mycterosuchus* (CAMSM J.1420), *Macrospondylus* (SMNS 51957), *Charitomenosuchus* (NHMUK PV R 3806) and *Neosteneosaurus* (NHMUK PV R 3701, PETMG R178). A second condition (state 1) is that the posteroventral margin is noticeably anteroposteriorly shortened and dorsoventrally broad, giving it a sub-square shape. This state is unique to machimosaurins (*Lemmysuchus*: NHMUK PV R 3168; *Mac. mosae*: ISRNB cast; Hua, 1999; Young et al., 2014).

**Figure 48:**
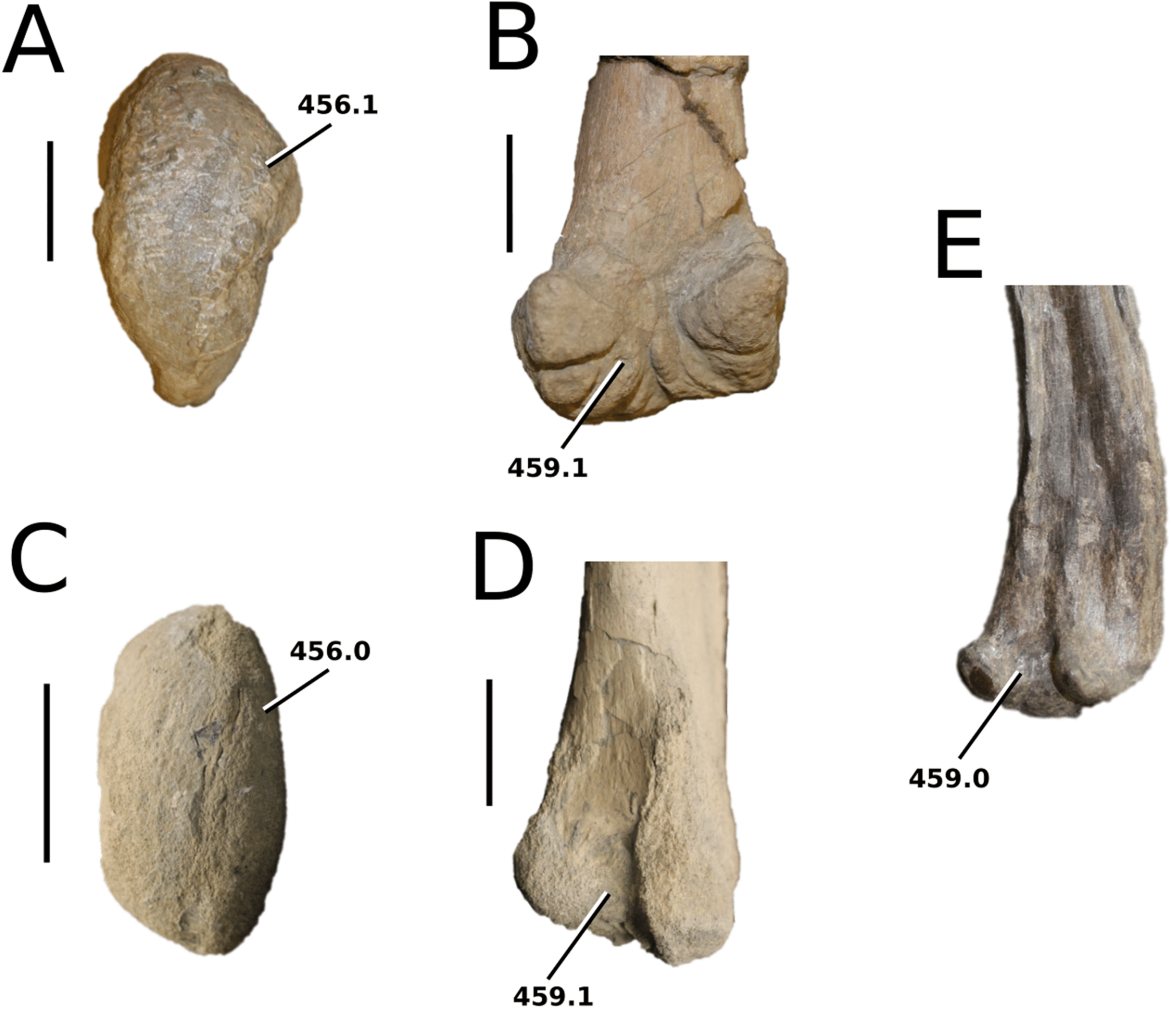
Comparative photographs: teleosauroid femora. Comparative photographs of teleosauroid femora (ch. 456 and 459): *Mycterosuchus nasutus* (NHMUK PV R 2617) ((A) femoral head dorsal view; (B) femoral condyles posterior view), *Neosteneosaurus edwardsi* (PETMG R178) ((C) femoral head dorsal view; (D) femoral condyles posterior view) and *Macrospondylus bollensis* (SMNS 51555) ((E) femoral condyles posterior view). Scale bars: 3 cm, (E) not to scale.

**456.** Femur in dorsal view, the anteromedial tuber is present and small (0), or the largest of the proximal tubera (1) (Fig. 48).

In most teleosauroids, the posteromedial tuber is the largest of the three femoral tubera, and the anteromedial tuber is present but relatively small (state 0). This is the condition seen in *Platysuchus* (SMNS 9930), *Sericodon* (SCR010-312 in Schaefer, Püntener & Billon-Bruyat, 2018), *Aeolodon* (MNHN.F.CNJ 78), *Macrospondylus* (SMNS 18672), *Charitomenosuchus* (NHMUK PV R 3806), *Neosteneosaurus* (PETMG R178) and machimosaurins (*Lemmysuchus*: NHMUK PV R 3168; *Machimosaurus*: Hua, 1999) The genus *Mycterosuchus* (NHMUK PV R 2617), however, has an anteromedial tuber that is noticeably well pronounced and well-developed, and it is the largest of all proximal tubera (state 1).

**459.** Femur, the distal medial and lateral condyles are the same size (0), or the medial condyle is larger than the lateral condyle (1) (Fig. 48).

In most teleosauroids, the medial and lateral condyles of the femur are approximately the same size (state 0). This condition is seen in the basal form *Plagiophthalmosuchus* (NHMUK PV OR 14792), as well as *Platysuchus* (SMNS 9930), *Aeolodon* (MNHN.F.CNJ 78), *Macrospondylus* (SMNS 51555) and *Lemmysuchus* (NHMUK PV R 3168). In certain teleosauroid genera, however, the femoral medial condyle is noticeably larger than the femoral lateral condyle (state 1). This is the case in *Mycterosuchus* (NHMUK PV R 2617) and *Neosteneosaurus* (NHMUK PV R 3701, PETMG R178).

**Figure 49:**
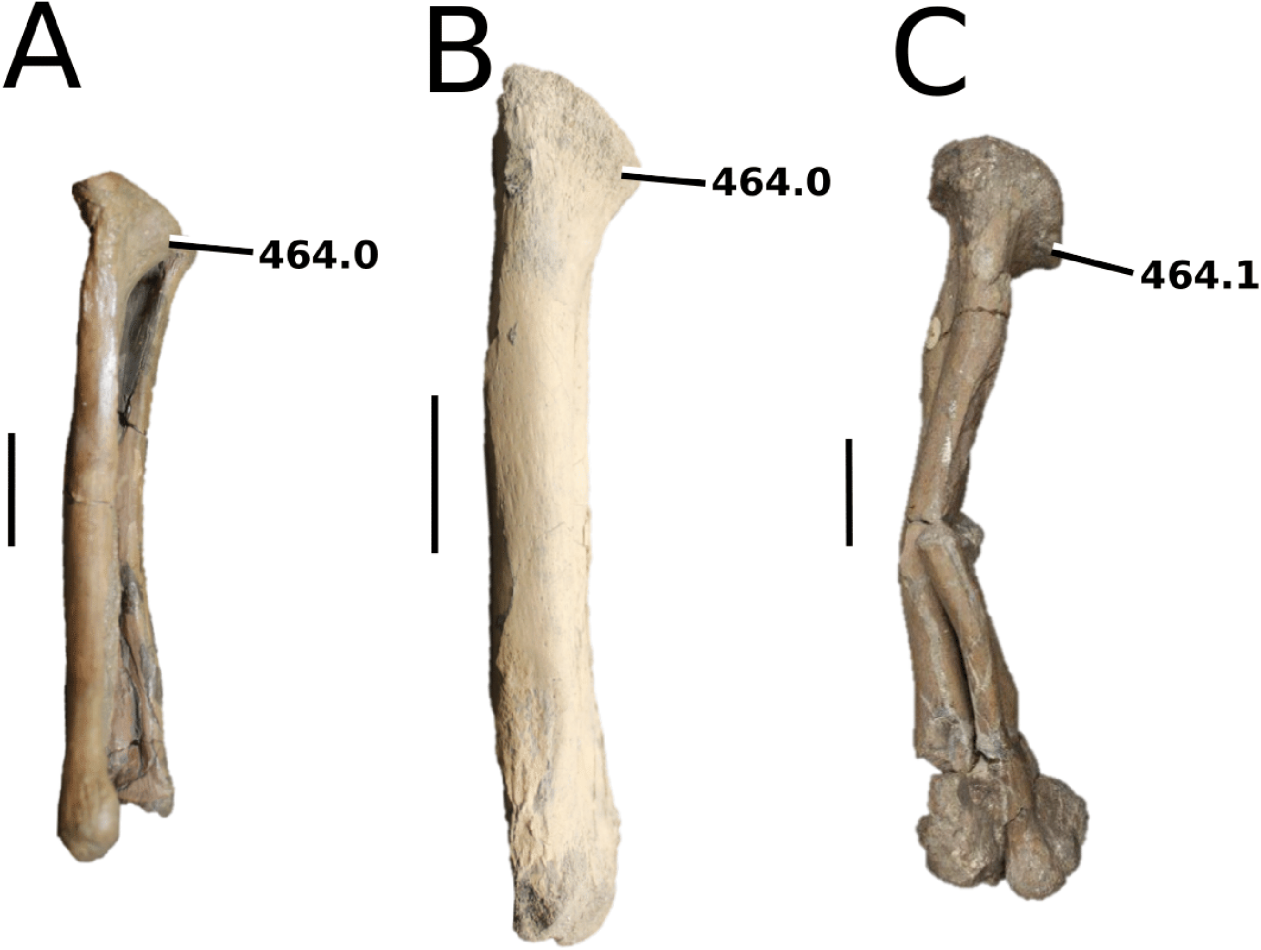
Comparative photographs: teleosauroid tibiae. Comparative photographs of teleosauroid tibiae, focusing on the tibal tuberosity (ch. 464): (A) *Charitomenosuchus leedsi* (NHMUK PV R 3806), (B) *Neosteneosaurus edwardsi* (PETMG R178) and (C) *Lemmysuchus obtusidens* (NHMUK PV R 3168). Scale bars: 3 cm.

**464.** Tibia in lateral view, the angle of tibial tuberosity is horizontal (0) or ventral (1) (Fig. 49).

In most scored teleosauroids, the tibial tuberosity is horizontally placed in lateral view (state 0). This is seen in the basal form *Plagiophthalmosuchus* (NHMUK PV OR 14792) as well as *Platysuchus* (SMNS 9930), *Mycterosuchus* (NHMUK PV R 2617), *Aeolodon* (MNHN.F.CNJ 78), *Macrospondylus* (SMNS 51984), *Charitomenosuchus* (NHMUK PV R 3806) and *Neosteneosaurus* (NHMUK PV R 3701, PETMG R178). In select teleosauroids, the angle of the tibial tuberosity is strongly ventrally displaced. This condition (state 1) is seen in machimosaurins (*Lemmysuchus*: NHMUK PV R 3168; *Machimosaurus*: IRSNB cast; Hua, 1999).

**Figure 50:**
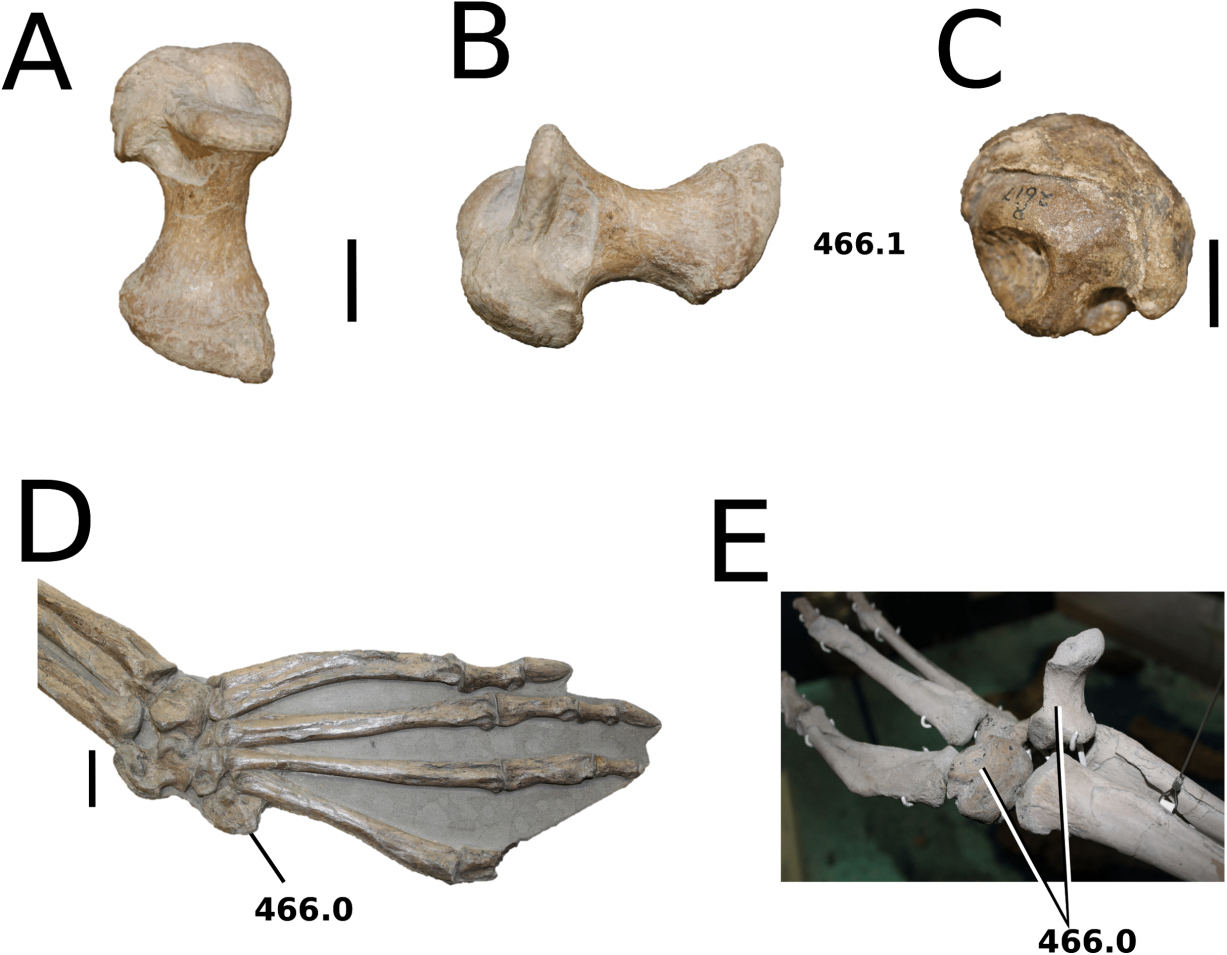
Comparative photographs: teleosauroid calcaneae and astragulae. Comparative photographs of teleosauroid calcaneae and astragulae (ch. 466): *Mycterosuchus nasutus* (NHMUK PV R 2617) ((A and B) calcaneum in (A) dorsal and (B) lateral view; and (C) astragulus), (D) *Macrospondylus bollensis* (SMNS 81699) and (E) *Neosteneosaurus edwardsi* (PETMG R175). Scale bars: 1.5 cm (A–C) and 2.5 cm (D), (E) not to scale.

**466.** Calcaneum, the calcaneum tuber is the same size (0) or larger (1) than the astragalus (Fig. 50).

Both the calcaneum and astragalus are approximately the same shapes in all scored teleosauroids; both tarsal bones are also relatively the same size as one another (state 0), with the calcaneum being marginally larger. This condition is observed in *Platysuchus* (SMNS 9930), *Macrospondylus* (MMG BwJ 565, SMNS 51984), *Charitomenosuchus* (NHMUK PV R 3806), *Neosteneosaurus* (PETMG R178) and *Lemmysuchus* (NHMUK PV R 3168). However, in *Mycterosuchus* (NHMUK PV R 2617) the enlarged calcaneum tuber is noticeably larger than the astragalus (state 1), by approximately 25%. This condition is currently autapomorphic for this genus.

**Figure 51:**
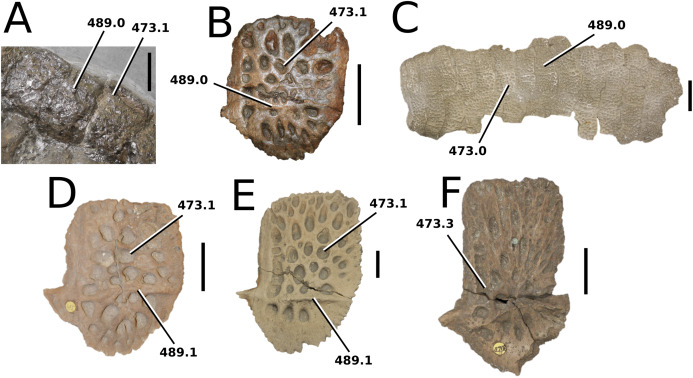
Comparative photographs: teleosauroid dorsal osteoderms. Comparative photographs displaying teleosauroid dorsal sacral osteoderms, with emphasis on ornamentation pattern (ch. 473) and keel presence (ch. 489): (A) *Plagiophthalmosuchus gracilirostris* (NHMUK PV OR 14892), (B) *Charitomenosuchus leedsi* (NHMUK PV R 3806), (C) *Teleosaurus cadomensis* (NHMUK PV R 119a), (D) *Mycterosuchus nasutus* (NHMUK PV R 2617), (E) *Neosteneosaurus edwardsi* (PETMG R178), and (F) *Lemmysuchus obtusidens* (NHMUK PV R 3168). Scale bars: 3 cm, (D) not to scale.

**489.** Sacral dorsal armour (osteoderms), the dorsal keel is elongated and shallow (0) or elongated and pronounced (1) (Fig. 51).

In certain teleosauroids, the longitudinal ridge (or keel) on the dorsal osteoderms is anteroposteriorly elongated but shallow (state 0). This condition is seen in *Plagiophthalmosuchus* (NHMUK PV OR 14792), *Platysuchus* (SMNS 9930), *Teleosaurus* (NHMUK PV R 4207, NHMUK PV OR 32584), *Aeolodon* (NHMUK PV R 1086, MNHN.F.CNJ 78), *Macrospondylus* (SMNS 51563) and *Charitomenosuchus* (NHMUK PV R 3806). In more derived teleosauroids, the keel of the sacral osteoderms is elongated, well-developed and thickened (state 1). State 1 is well exemplified in large specimens of *Neosteneosaurus* (PETMG R178) as well as the machimosaurin *Lemmysuchus* (NHMUK PV R 3168).

### Previous characters pertaining to teleosauroids

**Figure 52:**
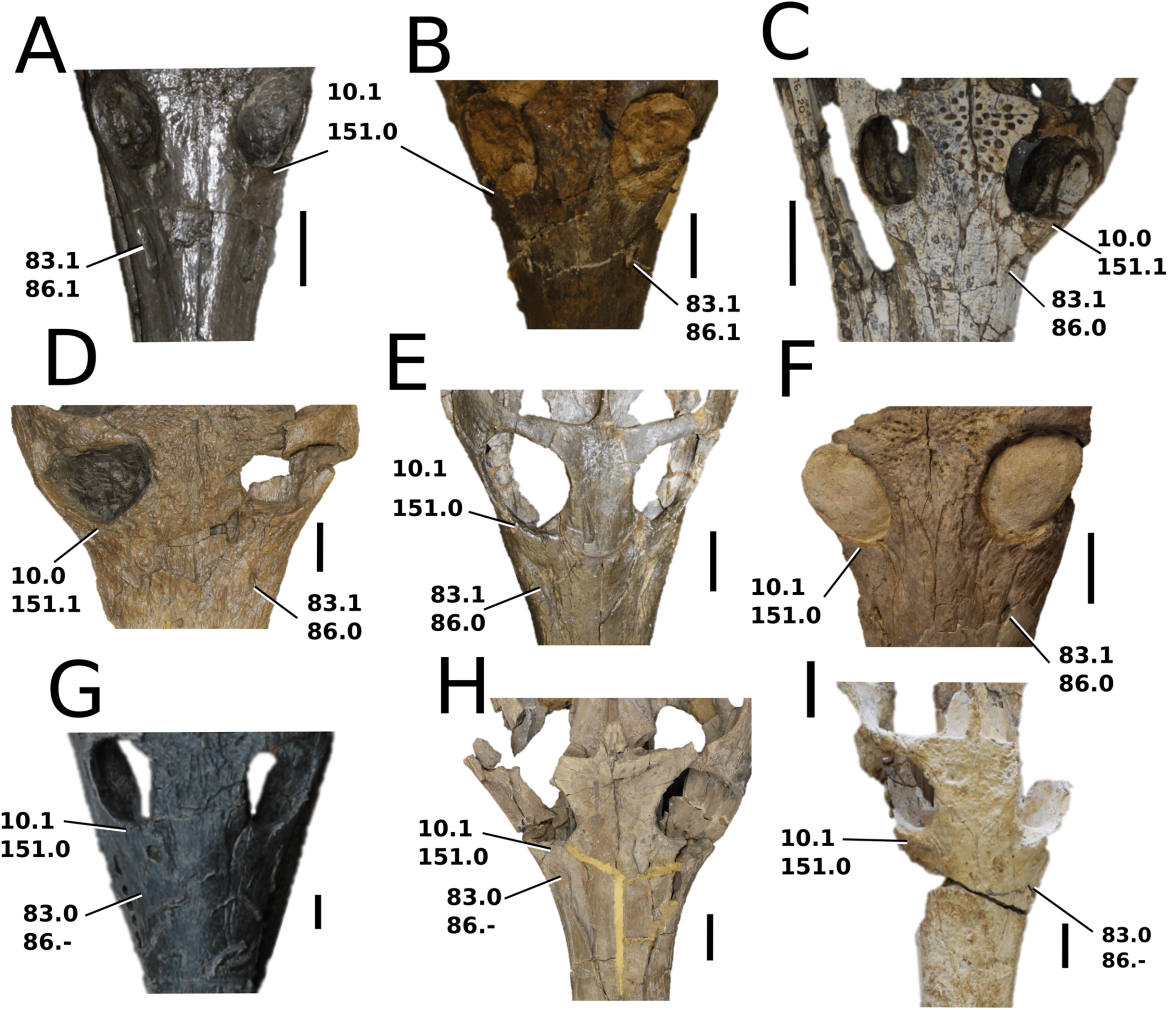
Comparative photographs: telescopic orbits and antorbital fenestrae. Comparative photographs displaying telescopic orbits (ch. 10, 151) as well as presence or absence (ch. 83) and shape of antorbital fenestrae (ch. 86) in dorsal view. (A) *Plagiophthalmosuchus gracilirostris* (NHMUK PV OR 14892); (B) *Deslongchampsina larteti* (OUMNH J.29851); (C) *Indosinosuchus potamosiamensis* (PRC-11); (D) *Mycterosuchus nasutus* (NHMUK PV R 2617); (E) *Charitomenosuchus leedsi* (NHMUK PV R 3806); (F) *Yvridiosuchus boutilieri* (OUMNH J.1401); (G) *Proexochokefalos heberti* (MNHN.F 1890-13); (H) *Neosteneosaurus edwardsi* (PETMG R178); and (I) *Lemmysuchus obtusidens* (LPP.M.21). Note the shallow antorbital fenestrae of *C. leedsi* compared to other taxa with antorbital fenestrae. Scale bars: 4 cm.

In addition to the 38 new characters described above, several original characters from the 2016 H+Y dataset are key in differentiating between various teleosauroid taxa. In particular, 19 characters are anatomically distinct, variant and important in teleosauroids and are described in detail as follows (as mentioned previously, all following characters are thoroughly described in SD4):

**10.** Rostrum narrows markedly in dorsal view immediately in front of the orbits (0), or there is no narrowing (1) (Fig. 52).

In most teleosauroids, the posterior portion of the rostrum will either narrow slightly mediolaterally or not narrow at all, instead becoming flush with the anterior rim of the orbit (state 1). This is seen in *Plagiophthalmosuchus* (NHMUK PV OR 14792), *Mystriosaurus* (NHMUK PV OR 14781), the Chinese teleosauroid (IVPP V 10098), *Platysuchus* (SMNS 9930), and a particular subclade of teleosauroids (e.g. *Macrospondylus* MMG BwJ 565; *Charitomenosuchus*: NHMUK PV R 3806; *Proexochokefalos*: MNHN.F 1890-13; *Mac. buffetauti* SMNS 91415). In certain teleosauroids, however, there is a distinct and pronounced narrowing, or mediolateral compression, of the rostrum immediately anterior to the orbits, causing the dorsal margins of the orbits to become upturned (state 0). This condition is in *Mycterosuchus* (NHMUK PV R 2617), *Aeolodon* (MNHN.F.CNJ 78), *I. potamosiamensis* (PRC-11), *Teleosaurus* (MNHN AC 8746), *Sericodon* (Schaefer, Püntener & Billon-Bruyat, 2018), and *Bathysuchus* (Foffa et al., 2019).

**Figure 53:**
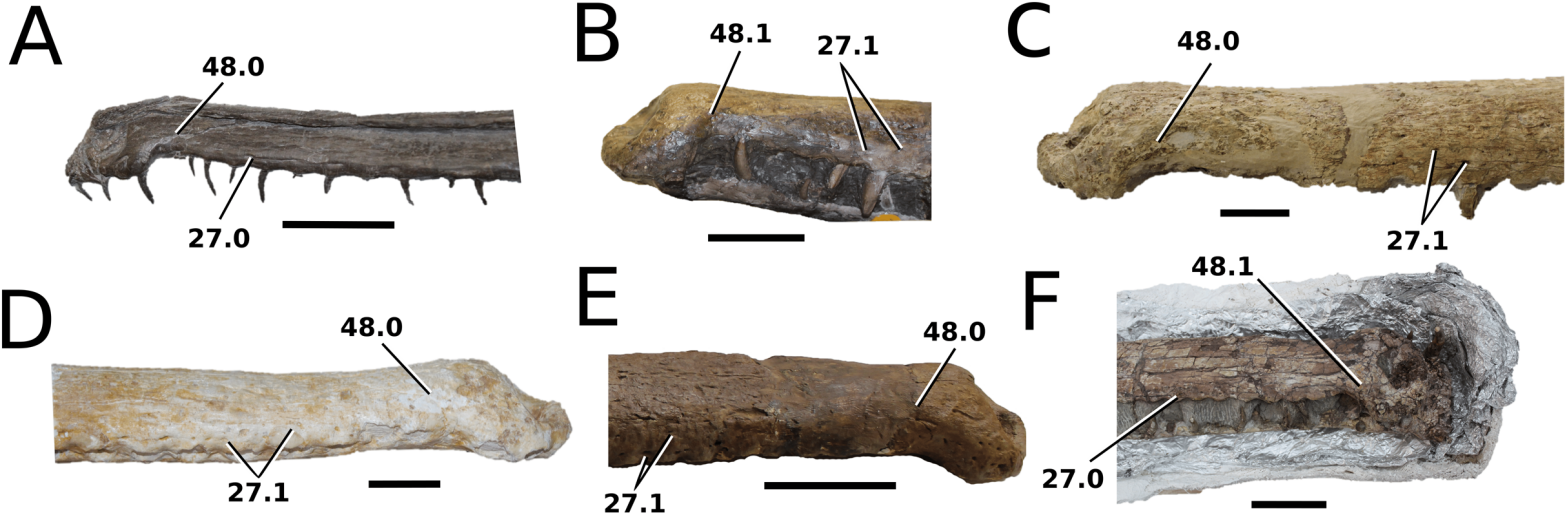
Comparative photographs: anterior and anterolateral premaxillary margins, and neurovascular foramina. Comparative photographs displaying the anterior and anterolateral premaxillary margins (ch. 48) as well as neurovascular foramina (ch. 27), in lateral view: (A) *Macrospondylus bollensis* (SMNS 51563), (B) *Mystriosaurus laurillardi* (NHMUK PV OR 14781), (C) *Machimosaurus buffetauti* (SMNS 91415), (D) *Lemmysuchus obtusidens* (LPP.M.21), (E) *Yvridiosuchus boutilieri* (OUMNH J.1401) and (F) *Indosinosuchus kalasinensis* (PRC-239). Scale bars: 5 cm.

**27.** Neurovascular foramina of the premaxillae/maxillae, represented by a single line of small sub-circular openings (0), or two lines (one dorsal, one ventral) of large, circular openings (1) (Fig. 53).

On the lateral premaxillae and maxillae, teleosauroids possess numerous neurovascular foramina. These openings are possibly involved with multiple mechanoreceptory function such as prey detection, tactile discrimination or disruption in the surrounding water (Soares, 2002; Leitch & Catania, 2012). In most teleosauroids, the neurovascular foramina are small and subcircular in shape on both the premaxilla and maxilla, and are generally consistent in size and number. On the premaxilla, these foramina are restricted to the anteroventral and lateroventral margins of the external nares. On the ventrolateral surface of the maxilla, dorsal to the tooth row, they form a single line and are relatively well spaced. This condition (state 0) is seen in taxa such as the basal-most teleosauroid *Plagiophthalmosuchus* (NHMUK PV OR 14792) and *Platysuchus* (SMNS 9930), *Mycterosuchus* (NHMUK PV R 2617), *Macrospondylus* (PMU R161), and *Neosteneosaurus* (NHMUK PV 2865). *Deslongchampsina* (OUMNH J. 29851) also has restricted foramina on the premaxilla as well as a single line on the maxilla; however, the foramina are larger than those seen in other taxa with state 0, and are slightly anteroposteriorly elongated on the maxilla (most notably at the anterior and middle areas of the rostrum).

State 1 is seen in the genus *Mystriosaurus* (NHMUK PV R 14781) along with members of Machimosaurini (*Yvridiosuchus*: OUMNH J.1401, OUMNH J.29850; *Lemmysuchus*: NHMUK PV R 3168; *Mac. buffetauti*: SMNS 91415; *Mac. mosae*: Young et al., 2014): these taxa display large, deep, numerous, sub-circular neurovascular foramina (although the foramina in *Mystriosaurus* are smaller than in machimosaurins). The premaxillary openings are generally circular in shape, located around the ventral, lateral and anteroventral margins of the external nares and cluster together (especially around the external nares’ lateral margins). On the maxilla, the foramina are more anteroposteriorly elongated and situated in two parallel lines, one dorsal to the tooth row with an additional line above it (state 1). The foramina are closely spaced together at the anterior part of the maxilla, but they gradually become more distanced from one another further posteriorly. In addition, it is interesting to note that the premaxillary foramina are exceptionally large in *Yvridiosuchus* (OUMNH J.29850) as well as only around the anteroventral margin of the external nares in *I. kalasinensis* (PRC-239).

**Figure 54:**
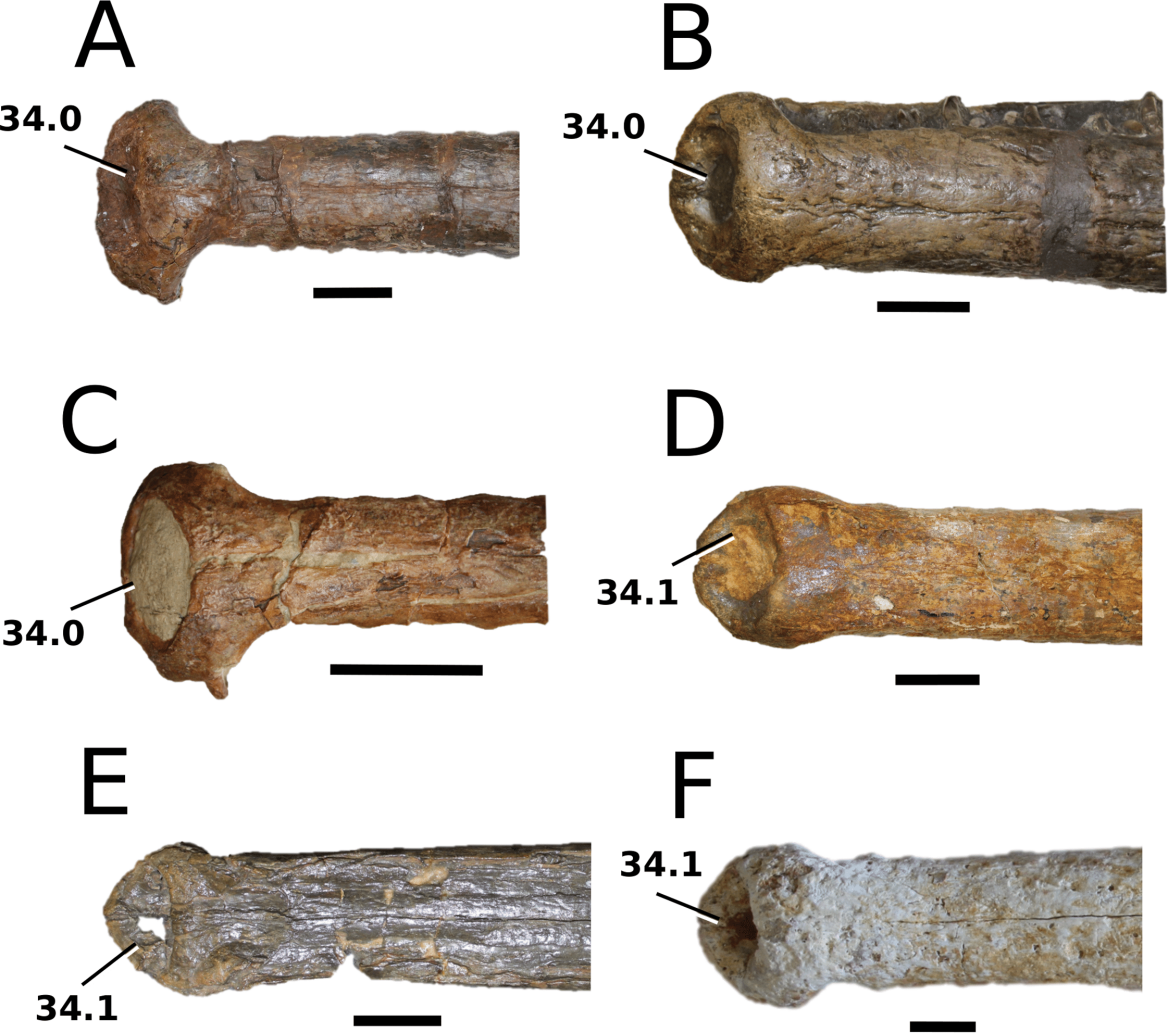
Comparative photographs: orientation of teleosauroid external nares. Comparative photographs displaying the external nares, in dorsal view (ch. 34): (A) the Chinese teleosauroid (IVPP V 10098), (B) *Mystriosaurus laurillardi* (HLMD V946-948), (C) *Bathysuchus megarhinus* (unnumbered LPP specimen), (D) *Deslongchampsina larteti* (OUMNH J.29851), (E) *Neosteneosaurus edwardsi* (NHMUK PV R 3701) and (F) *Lemmysuchus obtusidens* (LPP.M.21). Scale bars 3 cm.

**34.** External nares oriented anteriorly or anterodorsally (0), or dorsally (1) (Fig. 54).

In a certain group of predominately Laurasian teleosauroids, the external nares face either anteriorly or anterodorsally (state 0). This condition occurs in *Mystriosaurus* (NHMUK PV OR 14781), the Chinese teleosauroid (IVPP V 1009), *Mycterosuchus* (NHMUK PV R 2617), *Teleosaurus* (Eudes-Deslongchamps, 1867), *Platysuchus* (SMNS 9930), *Aeolodon* (MNHN.F.CNJ 78), *Sericodon* (SCR011-406 in Schaefer, Püntener & Billon-Bruyat, 2018) and *Bathysuchus* (unnumbered LPP specimen). In predominately Sub-Boreal/Gondwanan teleosauroids, the external nares are oriented dorsally (state 1). This is seen in *Macrospondylus* (PMU R161), *Charitomenosuchus* (NHMUK PV R 3806), *Deslongchampsina* (OUMNH J.29851), *Proexochokefalos* (MNHN.F 1890-13), *Neosteneosaurus* (NHMUK PV R 2865) and machimosaurins (*Yvridiosuchus*: OUMNH J.1401; *Lemmysuchus*: LPP.M.21; *Machimosaurus*: SMNS 91415).

**48.** Premaxilla in lateral view, the anterior and anterolateral premaxillary margins are not sub-vertical, or do not extend ventrally (0), or the anterior and anterolateral margins are orientated anteroventrally and extend ventrally (1) (Fig. 53).

In one teleosauroid subclade, the anterior and anterolateral margins of the premaxilla are not sub-vertical and do not extend ventrally (state 0) when compared to the rest of the premaxilla; rather, they are anterodorsally curved in a continuous arc throughout. This condition is seen in the basal teleosauroid *Plagiophthalmosuchus* (NHMUK PV OR 14792) as well as *Macrospondylus* (PMU R161), *Charitomenosuchus* (NHMUK PV R 3806), *Deslongchampsina* (OUMNH J.29851), *Proexochokefalos* (MNHN.F 1890-13), *Andrianavoay* (NHMUK PV R 1999), *Neosteneosaurus* (NHMUK PV R 2865) and Machimosaurini (e.g. *Lemmysuchus*: NHMUK PV R 3168). In the second teleosauroid subclade, the anterior and anterolateral premaxillary margins are strongly oriented anteroventrally and extend ventrally in lateral view, giving these margins a near-vertical appearance. This condition (state 1) occurs in *Mystriosaurus* (NHMUK PV OR 14781), the Chinese teleosauroid (IVPP V 10098), *Platysuchus* (SMNS 9930), *Mycterosuchus* (NHMUK PV R 2617), *I. potamosiamensis* (PRC-11), *Bathysuchus* (unnumbered LPP specimen) and *Aeolodon* (MNHN.F.CNJ 78). It is particularly well-developed in *Mystriosaurus* (NHMUK PV OR 14781) and the Chinese teleosauroid (IVPP V 10098).

**83.** Antorbital fenestrae/cavity, absent (0) or present (1) (Fig. 52).

In most teleosauroids, a small, slit-like or subcircular antorbital fenestra is present (state 1). This condition is seen in taxa such as *Mycterosuchus* (NHMUK PV R 2617), *Indosinosuchus* (PRC-11, PRC-239), *Teleosaurus* (MNHN AC 8746), *Charitomenosuchus* (NHMUK PV R 3806), *Macrospondylus* (MMG BwJ 565) and *Yvridiosuchus* (OUMNH J.1401). However, in *Proexochokefalos* (MNHN.F 1890-13), *Neosteneosaurus* (PETMG R178) and select members of Machimosaurini (*Lemmysuchus*: LPP.M.21; *Machimosaurus*: SMNS 91415; Young et al., 2014) the antorbital fenestrae (and internal antorbital fossae) are absent (state 0).

**86.** Antorbital fenestrae/cavity sub-circular (0) or anteroposteriorly elongated (1) in shape (Fig. 52).

In most teleosauroid taxa, the antorbital fenestra openings are subcircular or sub-oval in shape (state 0). This condition is seen in *Mystriosaurus* (NHMUK PV OR 14781), the Chinese teleosauroid (IVPP V 10098), *Indosinosuchus* (PRC-11; PRC-239), *Platysuchus* (SMNS 9930), *Teleosaurus* (MNHN AC 8746), *Mycterosuchus* (NHMUK PV R 2617), *Macrospondylus* (SMNS 51555), *Charitomenosuchus* (NHMUK PV R 3320) and *Yvridiosuchus* (OUMNH J.1401). Most notably, in *Plagiophthalmosuchus* (NHMUK PV OR 14792) and *Deslongchampsina* (OUMNH J.29851: Johnson, Young & Brusatte, 2019), the antorbital fenestrae are large and anteroposteriorly elongated (state 1), making them appear fully oval- or teardrop-shaped. Note that this character is not applicable for those taxa that lack antorbital fenestrae: *Proexochokefalos* (MNHN.F 1890-13), *Neosteneosaurus* (PETMG R178), *Lemmysuchus* (LPP.M.21) and *Machimosaurus* (SMNS 91415; Young et al., 2014).

**Figure 55:**
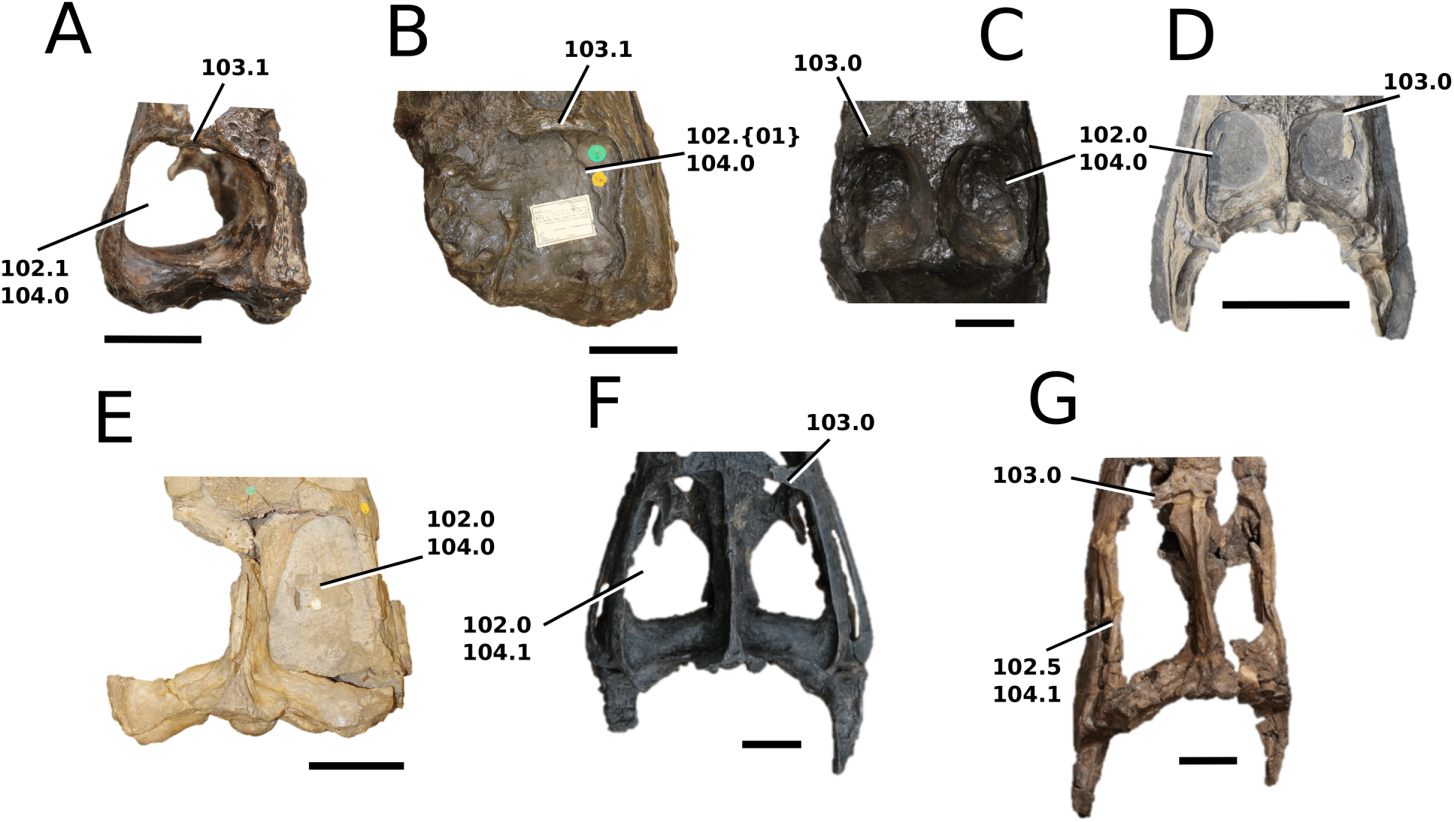
Comparative photographs: teleosauroid supratemporal fenestrae. Comparative photographs displaying the shape of the supratemporal fenestrae (ch. 102), as well as the anterolateral expansion of the anterior portion (ch. 103) and elongation (ch. 104) of these fenestrae in dorsal view. (A) *Teleosaurus cadomensis* (MNHN AC 8746); (B) *Mystriosaurus laurillardi* (NHMUK PV OR 14781); (C) *Plagiophthalmosuchus gracilirostris* (NHMUK PV OR 14892); (D) *Macrospondylus bollensis* (MMG BwJ 565); (E) *Clovesuurdameredeor stephani* (NHMUK PV OR 49126), (F) *Proexochokefalos heberti* (MNHN.F 1890-13); and (G) *Lemmysuchus obtusidens* (NHMUK PV R 3168). Scale bars: 3 cm (A and C) and 10 cm (B, D–F).

**102.** Supratemporal fenestrae, shape is either longitudinal ellipsoid or sub-rectangular (0), square-shaped (regular quadrilateral) (1), transverse (= extended) triangle (2), circular (3), triangle-shaped (three 60° points) (4), or parallelogram (5) (Fig. 55).

Teleosauroids show variance in the shape of the supratemporal fenestrae. Most taxa have a sub-rectangular shaped fenestra, in which the anteroposterior axis is greater than 10% longer than the lateromedial axis (state 0). This is the condition seen in *Plagiophthalmosuchus* (NHMUK PV OR 14792; MNHNL TU515), *Platysuchus* (SMNS 9930), the Chinese teleosauroid (IVPP V 10098), *Mycterosuchus* (NHMUK PV R 2617), *Aeolodon* (MNHN.F.CNJ 78), *Sericodon* (Schaefer, Püntener & Billon-Bruyat, 2018), *Bathysuchus* (unnumbered LPP specimen), *Macrospondylus* (MMG BwJ 565), *Clovesuurdameredeor* (NHMUK PV OR 49126), *Charitomenosuchus* (NHMUK PV R 3320), *Pr*. cf. *bouchardi* (Lepage et al., 2008), *Proexochokefalos* (MNHN.F 1890-13) and *Neosteneosaurus* (NHMUK PV R 2865, PETMG R178). Two teleosauroids, *I. potamosiamensis* (PRC-11) and *Teleosaurus* (MNHN AC 8746), show state 1, which is square-shaped supratemporal fenestrae; as with state 0, the anteroposterior axis is over 10% longer than the lateromedial axis. In Machimosaurini (*Yvridiosuchus*: OUMNH J.29850; *Lemmysuchus*: NHMUK PV R 3168; *Mac. buffetauti*: SMNS 91415; *Mac. mosae*: IRSNB cast, Young et al., 2014; *Mac. hugii*: NMS 7029) the supratemporal fenestrae are extremely elongated and parallelogram-shaped (state 5), with the lateral and medial margins, and anterior and posterior margins being sub-parallel. This state is a putative apomorphy within machimosaurins.

**103.** Anterior margin shape of supratemporal fenestra, no anterolateral expansion of the supratemporal fenestrae/fossae (0), or the anterior margin noticeably inclined anterolaterally (1) (Fig. 55).

In most teleosauroids, the anterior margin of the supratemporal fenestra is not anterolaterally expanded, and the anterolateral corners of the supratemporal fossae are parallel to the anteromedial corners, which makes the anterior margin of the supratemporal fenestrae appear horizontal in dorsal view (state 0). This condition is seen in the basal teleosauroid *Plagiophthalmosuchus* (NHMUK PV OR 17892) as well as one teleosauroid subclade (e.g *Macrospondylus* MMG BwJ 565; *Charitomenosuchus*: NHMUK PV R 3320; *Proexochokefalos*: MNHN.F 1890-13; *Lemmysuchus*: NHMUK PV R 3168; *Mac. buffetauti*: SMNS 91415). However, in the second subclade, the anterolateral corners of the supratemporal fossae are noticeably more inclined anteriorly than the anteromedial corners of the supratemporal fossae (state 1), giving the anterior margin an anteroposteriorly tilted appearance in dorsal view. State 1 is seen in *Mystriosaurus* (NHMUK PV OR 14781), the Chinese teleosauroid (IVPP V 10098), *Platysuchus* (SMNS 9930), *Mycterosuchus* (NHMUK PV R 2617), *Indosinosuchus* (PRC-11, PRC-239) and *Aeolodon* (MNHN.F.CNJ 78).

**104.** Supratemporal fenestrae, overall anteroposterior length is either less than or sub-equal to the anterior width (0), or is twice as long as the anterior width, or more (1) (Fig. 55).

This character is related in part to ch. **102**, specifically regarding the parallelogram-shaped supratemporal fenestrae see in Machimosaurini. In most teleosauroids, the anteroposterior length of the supratemporal fenestrae is approximately the same as the width (state 0). This condition is in the basal-most form *Plagiophthalmosuchus* (NHMUK PV OR 14792) as well as *Mystriosaurus* (NHMUK PV OR 14781), *Indosinosuchus* (PRC-11; PRC-239), *Platysuchus* (SMNS 9930), *Teleosaurus* (MNHN AC 8746), *Mycterosuchus* (NHMUK PV R 2617), *Bathysuchus* (unnumbered LPP specimen), *Aeolodon* (MNHN.F.CNJ 78), *Macrospondylus* (MMG BwJ 565), *Clovesuurdameredeor* (NHMUK PV OR 49126), *Charitomenosuchus* (NHMUK PV R 3806) and *Deslongchampsina* (OUMNH J.29851). In more derived teleosauroids, the anteroposterior width of the supratemporal fenestrae are approximately twice as long as the width (state 1). This condition is in *Proexochokefalos* (MNHN.F 189013), *Pr*. cf. *bouchardi* (Lepage et al., 2008), *Neosteneosaurus* (PETMG R178) and machimosaurins (e.g. *Lemmysuchus*: NHMUK PV R 3168).

**151.** The circumorbital dorsal margins of the orbits are flush with the skull dorsal surface (0), upturned (prominent along the orbital medial margin in dorsal view, with the frontal interorbital margins being upturned) (1), or upturned along with the posterior margins (the frontal lateral process anterior margins are also upturned) (2) (Fig. 52).

In the majority of teleosauroids, the orbital dorsal margins are flush (=flattened) with the skull dorsal surface (state 0) and display no evidence of any dorsal upturn. This condition is seen in the basal teleosauroid *Plagiophthalmosuchus* (NHMUK PV OR 14792) as well as *Mystriosaurus* (NHMUK PV OR 14781), the Chinese teleosauroid (IVPP V 10098), *I. kalasinensis* (PRC-239), *Platysuchus* (SMNS 9930), *Macrospondylus* (MMG BwJ 565), *Clovesuurdameredeor* (NHMUK PV OR 49126), *Charitomenosuchus* (NHMUK PV R 3320), *Deslongchampsina* (OUMNH J.29851), *Proexochokefalos* (MNHN.F 1890-13), *Neosteneosaurus* (NHMUK PV R 2865) and Machimosaurini (e.g. *Lemmysuchus*: LPP.M.21). Four teleosauroid taxa (*I. potamosiamensis*: PRC-11; *Mycterosuchus*: NHMUK PV R 2617; *Teleosaurus*: MNHN AC 8746; *Aeolodon*: MNHN.F.CNJ 78) have a definitive upturning of the orbital dorsal margin (state 1), contributing to the protruding appearance of the orbits.

**Figure 56:**
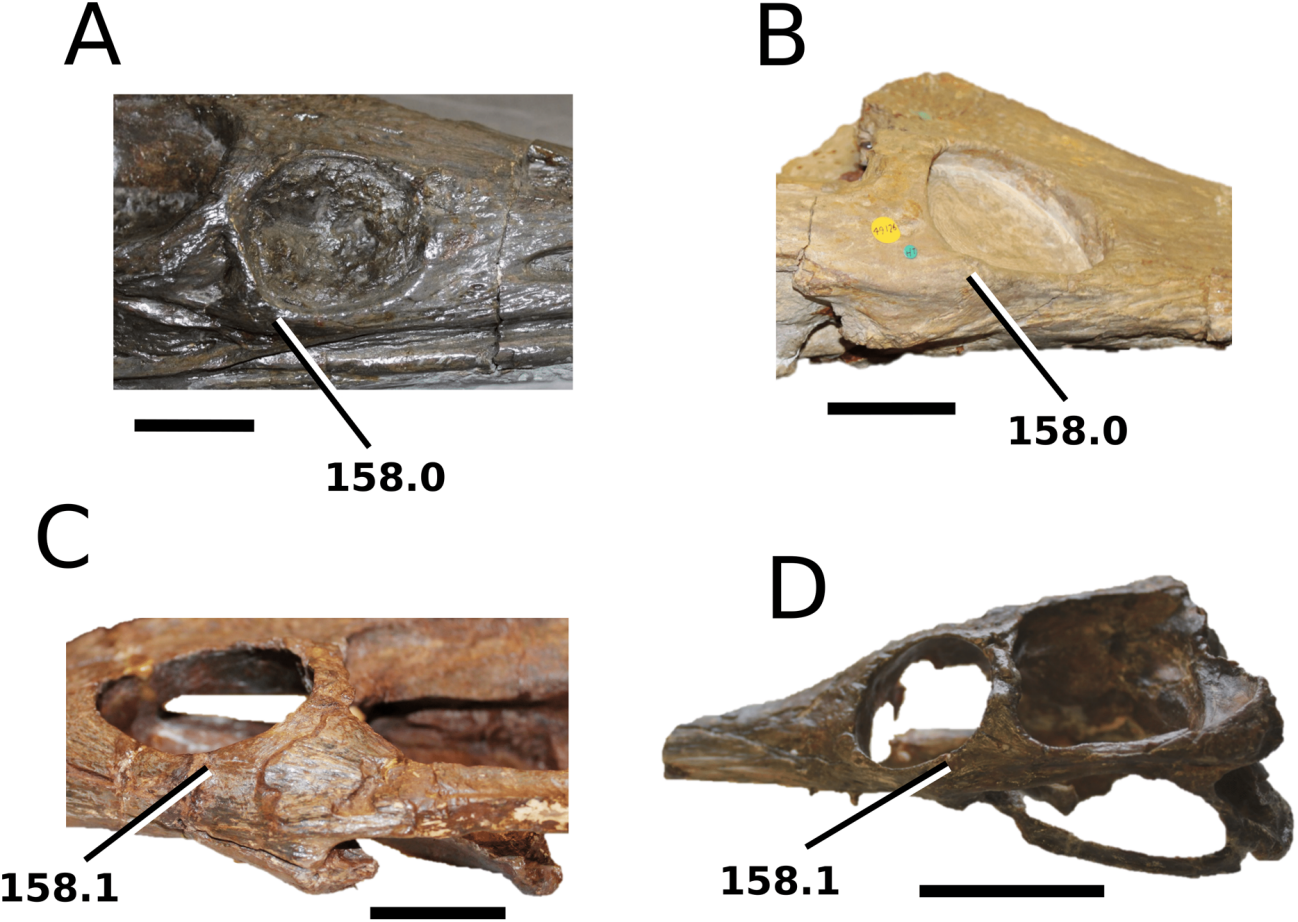
Comparative photographs: teleosauroid orbital margin. Comparative photographs of teleosauroid orbital margin (in lateral view), focusing on the inclusion of the postorbital (ch. 158): (A) *Plagiophthalmosuchus gracilirostris* (NHMUK PV OR 14892), (B) *Clovesuurdameredeor stephani* (NHMUK PV OR 49126), (C) the Chinese teleosauroid (IVPP V 10098) and (D) *Teleosaurus cadomensis* (MNHN AC 8746). Scale bars: 3 cm.

**158.** Orbit, the postorbital is excluded from the orbit posteroventral margin or only present in the posteroventral margin (0), or the postorbital reaches the orbit posteroventral margin and extensively forms part of the orbit ventral margin (1) (Fig. 56).

In most teleosauroids, the postorbital does not contact the posteroventral margin of the orbit (state 0). This is the condition seen in the basal-most teleosauroid (*Plagiophthalmosuchus*: MNHNL TU515, NHMUK PV OR 14792) as well as more derived taxa (e.g. *Charitomenosuchus*: NHMUK PV R 3806; *Proexochokefalos*: MNHN.F 1890-13; *Yvridiosuchus*: OUMNH J.29850; *Mac. mosae*: IRSNB cast). However, in some teleosauroid taxa, the postorbital contacts the posteroventral margin of the orbit, forming a substantial proportion of the orbital ventral margin. Due to this extension, the postorbital often overlaps the posterior part of the jugal. This condition (state 1) is found in basal teleosauroids (*Mystriosaurus*: NHMUK PV OR 14781; the Chinese teleosauroid: IVPP V 10098; *I. potamosiamensis*: PRC-11; *Platysuchus*: SMNS 9930; *Teleosaurus*: MNHN AC 8746; *Mycterosuchus*: CAMSM J.1420).

**Figure 57:**
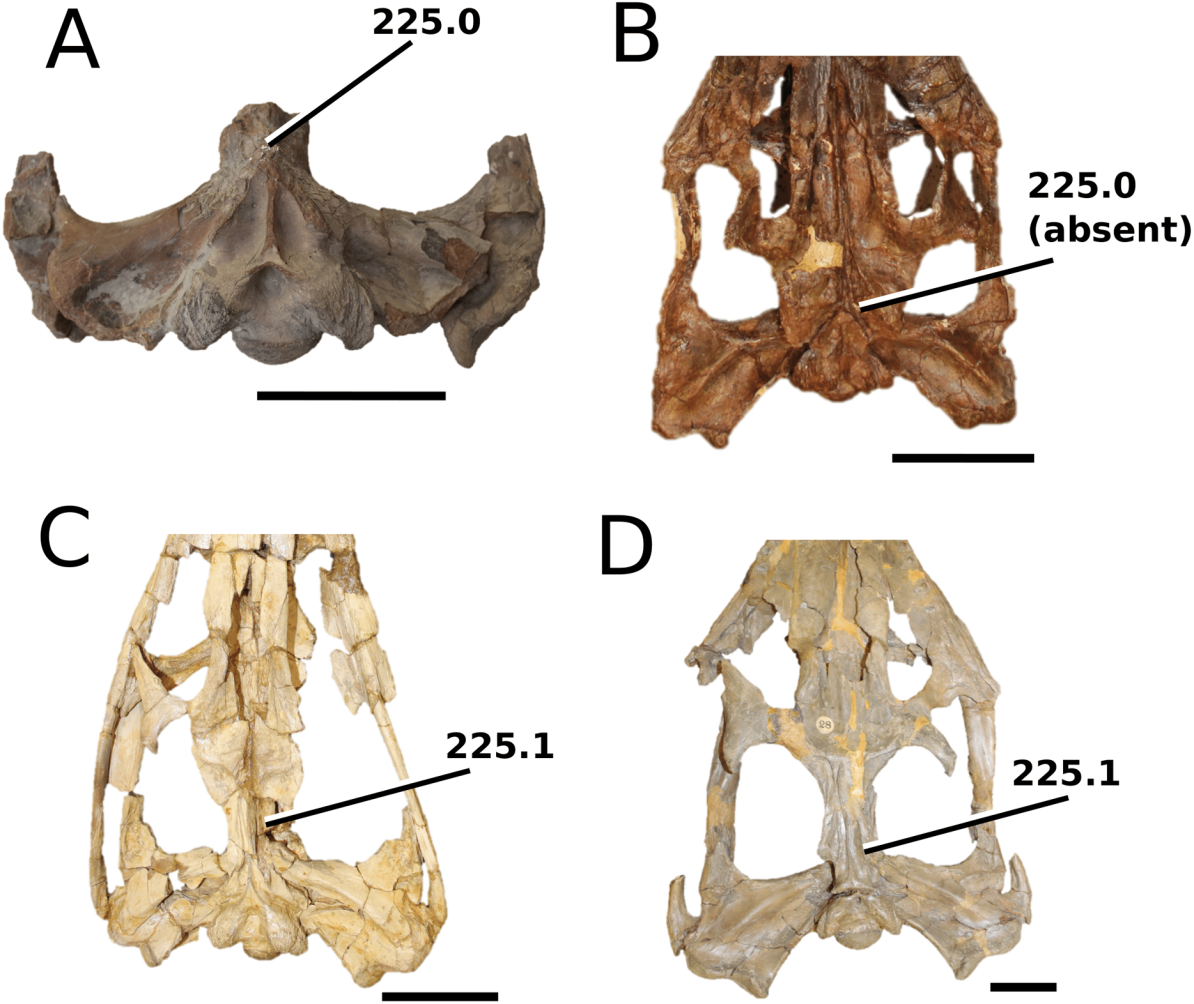
Comparative photographs: exposure of the teleosauroid basioccipital. Comparative photographs exhibiting exposure of the teleosauroid basioccipital (ch. 225): (A) *Mycterosuchus nasutus* (CAMSM J.1420), (B) the Chinese teleosauroid (IVPP V 10098), (C) *Charitomenosuchus leedsi* (NHMUK PV R 3320) and (D) *Neosteneosaurus edwardsi* (NHMUK PV R 2865). Scale bars: 7 cm.

**225.** Basisphenoid, exposure anterior to the quadrates in palatal view: absent or basisphenoid terminates approximately level to the anterior extent of the quadrates (0), or basisphenoid ‘rostrum’ (= cultriform process) is exposed along the palatal surface anterior to the quadrates and continues to bifurcate the pterygoids (1) (Fig. 57).

In certain teleosauroids, when examining the anterior exposure of the basisphenoid in palatal view, this bone is either absent or terminates approximately at the level of the anterior-most quadrates (state 0). This is the condition seen in the Chinese teleosauroid (IVPP V 10098), *I. potamosiamensis* (PRC-11), *Teleosaurus* (MNHN AC 8746) and *Mycterosuchus* (CAMSM J.1420). In the majority of teleosauroids, the basisphenoid is well exposed along the palatal surface anterior to the quadrates and bifurcates the pterygoids (state 1), which is caused by the posterior expansion of the posterior margin of the pterygoid. State 1 is a putative synapomorphy of one teleosauroid subclade and is seen in *Macrospondylus* (SMNS 81699), *Clovesuurdameredeor* (NHMUK PV OR 49126), *Charitomenosuchus* (NHMUK PV R 3320), *Deslongchampsina* (OUMNH J.29851), *Proexochokefalos* (MNHN.F 1890-13), *Neosteneosaurus* (NHMUK PV R 2865), *Yvridiosuchus* (OUMNH J.403) and *Lemmysuchus* (LPP.M.21).

**327.** Teeth along the entirety of the tooth row, with sharp, pointed apices (0) or blunt, round apices (1) (Fig. 40).

Teeth that are elongate and slender with pointed apices (state 0) can clearly be seen in the basal-most form *Plagiophthalmosuchus* (MNHNL TU515) and in most teleosauroids (e.g. *I. kalasinensis*: PRC-238, PRC-239; *Platysuchus*: SMNS 9930; *Mycterosuchus*: NHMUK PV R 2617; *Bathysuchus*: DORCM G.05067iv; *Charitomenosuchus*: NHMUK PV 3806). While the taxa *Mystriosaurus* (HLMD V946-948, NHMUK PV OR 14781), *Proexochokefalos* (MNHN.F 1890-13), *Deslongchampsina* (OUMNH J.29851) and *Neosteneosaurus* (PETMG R178) possess teeth with pointed apices (and are therefore scored as state 0), it is important to note that the overall dentition of these four genera are more robust than in the other aforementioned teleosauroids. In particular, the posterior teeth of *Neosteneosaurus* (PETMG R178) are noticeably more conical but continue to retain a pointed apex. The tribe Machimosaurini (Jouve et al., 2016) is unique in that all members (*Yvridiosuchus*: OUMNH J.29850; *Lemmysuchus*: NHMUK PV R 3618; *Machimosaurus*: LMH 16387, LMH 16405, MG-8730-1, ONM NG 7, SMF 2027, SMNS 91415) have conical teeth with blunt, rounded apices (state 1) throughout the entirety of the dentition.

**358.** Morphology of apical enamel surface ornamentation, macroscopic anastomosed pattern absent (0) or present (1) (Fig. 40).

As with the above character, the apices of the teeth are relatively smooth and unornamented aside from the enamel ridges that reach the tip of the apex (state 0) in most teleosauroids. This is the condition seen in *Plagiophthalmosuchus* (MNHNL TU515), as well as *Mystriosaurus* (NHMUK PV OR 14781); *I. kalasinensis* (PRC-239); *Platysuchus* (SMNS 9930); *Teleosaurus* (Eudes-Deslongchamps, 1867); *Mycterosuchus* (NHMUK PV R 2617); *Bathysuchus* (DORCM G.05067iv); *Sericodon* (TCH005-151 in Schaefer, Püntener & Billon-Bruyat (2018)); *Aeolodon* (NHMUK PV R 1086); *Macrospondylus* (MNHNL TU799); *Charitomenosuchus* (NHMUK PV R 3806); *Seldsienean* (OUMNH J.1414); *Deslongchampsina* (OUMNH J.29851); *Proexochokefalos* (MNHN.F 1890-13); and *Neosteneosaurus* (NHMUK PV R 3701; PETMG R178). However, the tribe Machimosaurini evolved a complex ornamentation pattern (state 1); this pattern is often referred to as ‘anastomosed’, which is a rough, ‘wrinkled’ texture, visible to the naked eye, on the apical third of the tooth. Anastomosed teeth are one of the characteristic features in machimosaurins, present in all members of the group (*Yvridiosuchus*: OUMNH J.29850; *Lemmysuchus*: NHMUK PV R 3168; *Machimosaurus*: SMNS 91415, MG-8730-1, ONM NG 7, SMF 2027).

**379.** Number of sacral vertebrae: two (0) or three (1) (Fig. 43).

In the majority of teleosauroids, there are two sacral vertebrae (state 0). This condition is seen in the basal form *Plagiophthalmosuchus* (NHMUK PV OR 14792) as well as *Platysuchus* (SMNS 9930), *Teleosaurus* (NHMUK PV OR 32588), *Mycterosuchus* (NHMUK PV R 2617), *Aeolodon* (MNHN.F.CNJ 78), *Macrospondylus* (SMNS 52034), *Charitomenosuchus* (NHMK PV R 3806), and *Neosteneosaurus* (NHMUK PV R 3701, PETMG R178). However, in scored members of Machimosaurini (*Lemmysuchus*: NHMUK PV R 3618; *Mac. mosae*: IRSNB cast, Hua, 1999), three sacral vertebrae are present (state 1), which is a unique feature of this clade. The first two vertebrae are true sacrals, with the first caudal vertebra appearing and functioning as a third sacral.

**Figure 58:**
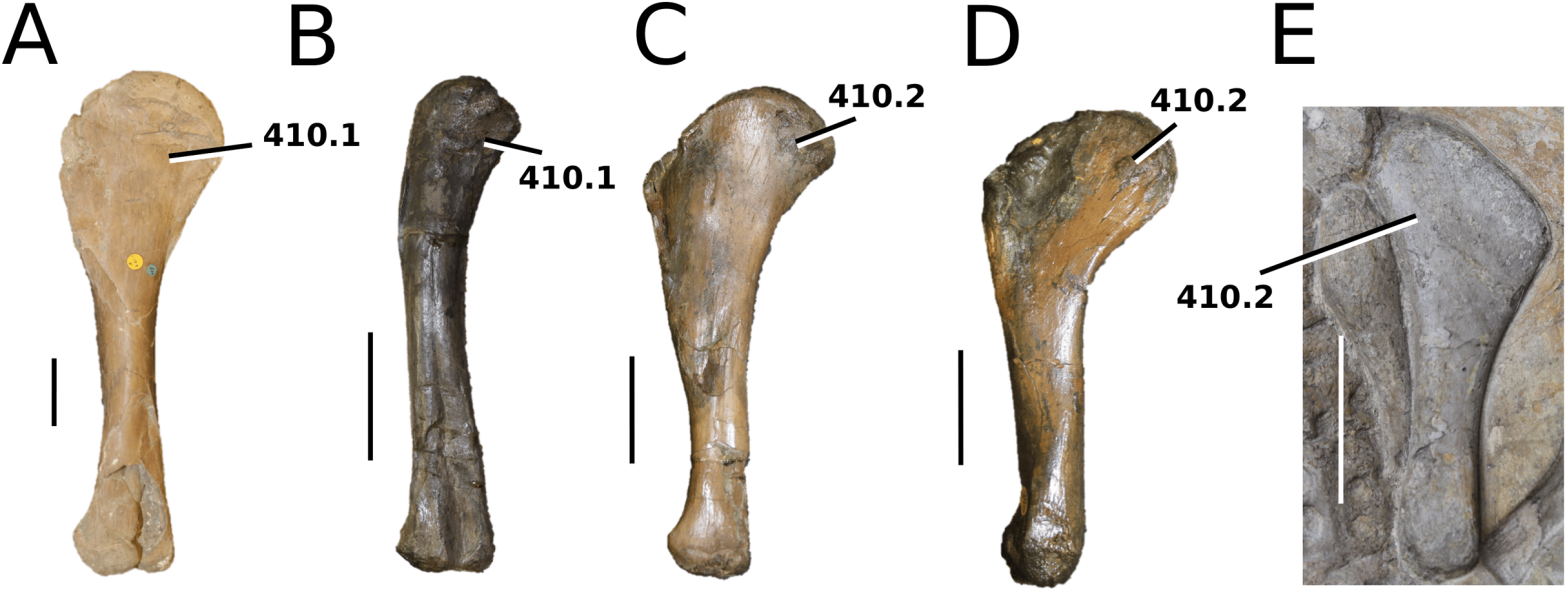
Comparative photographs: teleosauroid humeri. Comparative photographs of teleosauroid humeri (ch. 410): (A) *Mycterosuchus nasutus* (NHMUK PV R 2617), (B) *Macrospondylus bollensis* (SMNS 18672), (C) *Neosteneosaurus edwardsi* (NHMUK PV R 3701), (D) *Charitomenosuchus leedsi* (NHMUK PV R 3806) and (E) *Aeolodon priscus* (MNHN.F.CNJ 78). Scale bars: 3 cm. (ch. 410):

**410.** Humerus, humeral head: confined to the proximal surface (0), gently posteriorly expanded and hooked (1), or very strongly posteriorly deflected and hooked (2) (Fig. 58).

In scored teleosauroids, the proximal area of the humerus is either gently posteriorly expanded and hooked (state 1) or strongly deflected and hooked (state 2); it is never confined to the proximal surface (state 0). In basal teleosauroids such as *Plagiophthalmosuchus* (NHMUK PV OR 14792), *Platysuchus* (SMNS 9930), *Teleosaurus* (OUMNH J.26801), *Macrospondylus* (SMNS 51957) and *Mycterosuchus* (NHMUK PV R 2617), the proximal humerus (or humeral head) is anteroposteriorly elongated and gently but noticeably hooked (state 1). In the teleosauroids *Aeolodon* (MNHN.F.CNJ 78), *Charitomenosuchus* (NHMUK P R 3806) and *Neosteneosaurus* (PETMG R178), the posterior deflection of the proximal humerus is strong, so much so that the proximal epiphysis is noticeably posterior to the distal epiphysis. This posterior deflection is much more pronounced than in any other thalattosuchian taxa.

**420.** Ulna, olecranon process mediolaterally compressed and greatly proximally expanded: no (0), yes (1) (Fig. 44).

Only two basal teleosauroids (*Platysuchus*: SMNS 9930; *Macrospondylus* SMNS 53422) score as 0, in which the olecranon process is neither compressed nor expanded. Interestingly, more derived teleosauroids score as state 1, where the olecranon process is both greatly expanded and mediolaterally compressed. This is seen in *Mycterosuchus* (NHMUK PV R 2617), *Aeolodon* (MNHN.F.CNJ 78), *Charitomenosuchus* (NHMUK PV R 3806), *Neosteneosaurus* (PETMG R178) and *Lemmysuchus* (NHMUK PV R 3168).

**440.** Ilium, postacetabular (= posterior) process expanded into a thin ‘fan’ shape: no (0), yes (1) (Fig. 46).

In most teleosauroids, the postacetabular (=posterior) iliac process is either anteroposteriorly shortened, robust and process-like (state 0) or anteroposteriorly expanded and mediolaterally thin, expanding it into a ‘fanlike’ shape (state 1), and is best seen in either lateral or medial view. In *Charitomenosuchus* (NHMUK PV R 3806), *Neosteneosaurus* (PETMG R178), *Lemmysuchus* (NHMUK PV R 3816) and *Mac. mosae* (Young et al., 2014), state 1 is present, with the postacetabular process lengthened into a mediolaterally thin ‘fan-like’ shape. However, it is important to note that state 1 is a putative apomorphy of derived teleosauroids, and is not seen in basal taxa such as *Plagiophthalmosuchus* (NHMUK PV OR 14792), *Platysuchus* (SMNS 9930), *Teleosaurus* (NHMUK PV OR 32588), *Sericodon* (SCR010-312 in Schaefer, Püntener & Billon-Bruyat, 2018) and *Macrospondylus* (SMNS 18672, SMNS 51753).

**473.** Ornamentation (dorsal osteoderms), the pits are either small round to ellipsoid and very densely distributed (0), large round to ellipsoid and well separated (1), irregularly shaped with an extreme variation in size, with elongate pits present on the ventrolateral surface running from the keel to the lateral margin (2), or variable in both size, shape and length that radiate in a starburst pattern (3) (Fig. 51).

While the overall shape of the dorsal osteoderms is consistent in certain areas of the body across taxa, the ornamentation (or pitting) pattern differs, most notably in the thoracic/sacral osteoderms. In most teleosauroids, the pits are large, subcircular to ellipsoid in shape, and generally well separated from one another. This condition (state 1) is seen in *Plagiophthalmosuchus* (NHMUK PV OR 14792), *Mycterosuchus* (NHMUK PV R 2617), *Charitomenosuchus* (NHMUK PV R 3806) and *Neosteneosaurus* (NHMUK PV R 2865; NHMUK PV R 3701; PETMG R178). In *Charitomenosuchus* (NHMUK PV R 3806), the pits are arranged in a semi-circular pattern, and the larger ones are situated more towards the lateral margins of the osteoderm. In *Neosteneosaurus* (NHMUK PV R 2865), most pits are exceptionally large (especially situated in the centre of the osteoderm), subcircular and fewer in number. While the osteoderm ornamentation in the holotype of *Macrospondylus* (MMG BwJ 595) is poorly preserved, the pits appear to be large and semi-ellipsoid with a strong anteroposterior keel. The pits also appear to be more closely placed to one another, which is observed in other *Macrospondylus* specimens (e.g. MMG BwJ 565; SMNS 51563; SMNS 51753), with a thin ridge separating them. In two teleosauroid taxa, the ornamental pits are small, round, and extremely densely distributed throughout the entirety of the dorsal osteoderms (state 0). This is seen in *Platysuchus* (SMNS 9930) and *Teleosaurus* (NHMUK PV R 119a). Certain teleosauroids, however, possess thoracic/sacral osteoderms with exceptionally enlarged, elongated pits; due to this elongation and large size, these pits merge with one another and become elongated grooves, especially along the lateral margins, with the pits radiating distally in a ‘starburst’ pattern (state 3). The remainder of the pits are variable in size (from small to large), irregularly shaped, and relatively close together. In addition, well-developed keels are generally present in these osteoderms. This condition is observed in machimosaurins (*Lemmysuchus*: NHMUK PV R 3618; *Machimosaurus*: ONM 1-25, SMNS 91415, Young et al., 2014). State 2, in which the pits are all irregularly shaped with extreme variation in size and have no ‘starburst’ pattern, is not present in any known teleosauroid taxa.

## Cladistic Analysis: Results

### Most parsimonious unweighted strict consensus

**Figure 59:**
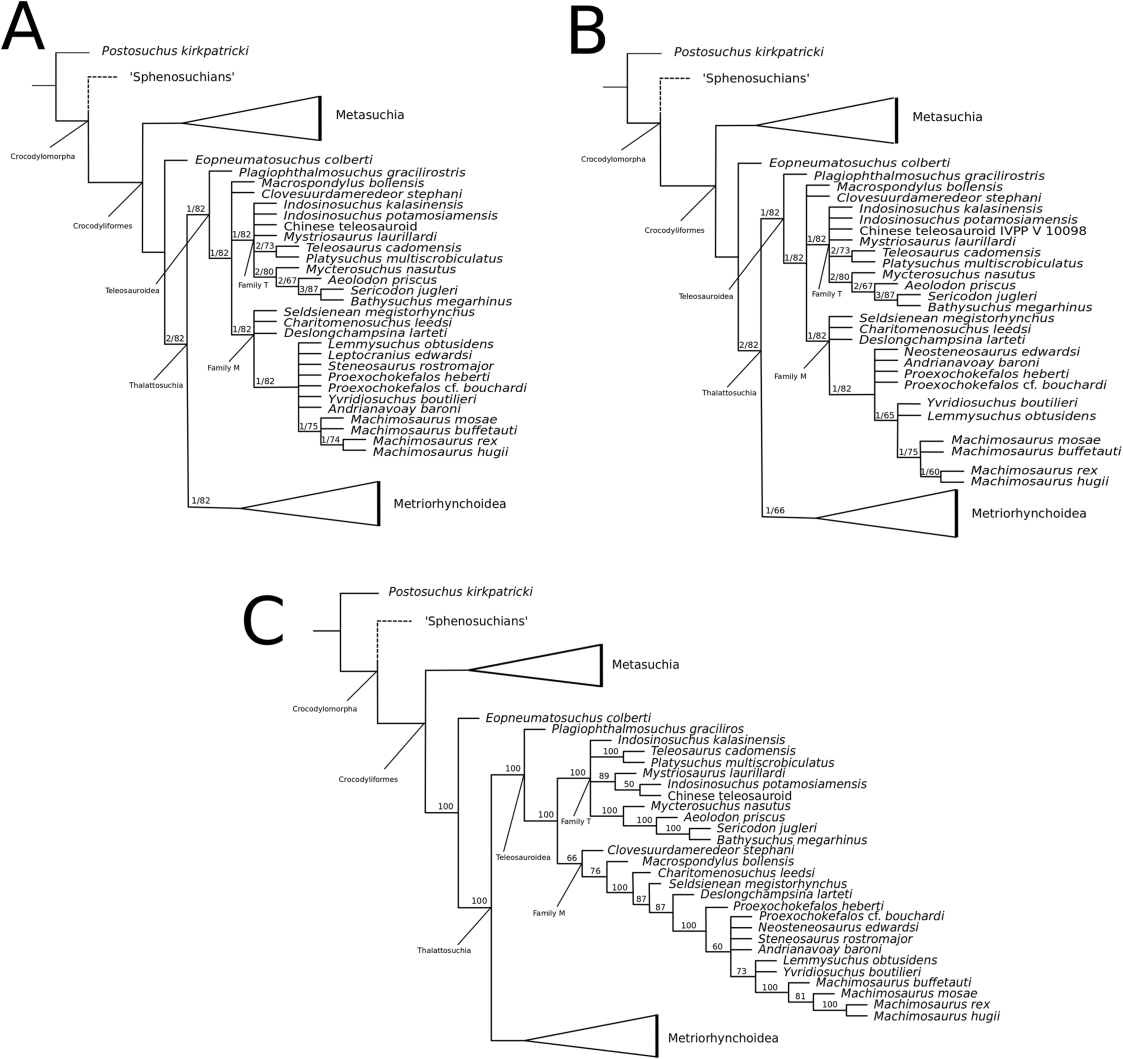
Topologies of the unweighted parsimonious phylogenetic analysis. Results of the unweighted parsimonious phylogenetic analysis, focusing on Teleosauroidea. (A) simplified strict consensus topology (125 MPTs and 1,659 steps: CI = 0.405, RI = 0.844); (B) simplified strict consensus topology excluding *S. rostromajor* (176 MPTs and 1,659 steps: CI = 0.405, RI = 0.844); and (C) parsimonious majority rules topology (125 MPTs and 1,659 steps). In all topologies Teleosauroidea is monophyletic and two distinct families (T and M) are recovered. Bremer support and jackknife values (Bremer/jackknife; A and B) and support percentages (C) are included.

The initial New Technology search recovered 125 most parsimonious trees (MPTs) of 1,659 steps (ensemble consistency index (CI) = 0.405; ensemble retention index (RI) = 0.844; ensemble rescaled consistency index (RCI) = 0.342; ensemble homoplasy index (HI) = 0.595) (Fig. 59A). With TBR branch swapping set to 100, 260 MPTs and 1,659 steps were recovered; when set to 1,000, 2,740 MPTs and 1,659 steps were found, with the best score hitting 301 out of 1,000 times. The overall topology did not change, with or without TBR.

In this topology, *Eopneumatosuchus colberti* Crompton & Smith, 1980, was found to be the immediate outgroup to Thalattosuchia, which was divided into two groups: Metriorhynchoidea and Teleosauroidea. Within Teleosauroidea, *Plagiophthalmosuchus* was recovered as the basal-most teleosauroid. This is weakly supported, with a jackknife percentage of 66% and a Bremer support value of 1. There are two main teleosauroid families recovered (see discussion on clades below), with the taxa *Clovesuurdameredeor* and *Macrospondylus* (which form a separate polytomy) being most closely related to both of them. Within the first family (Family T) (Fig. 59A), *I. kalasinensis*, *I. potamosiamensis*, the Chinese teleosauroid (IVPP V 10098) and *Mystriosaurus* are unresolved with one another and are most closely related to two remaining subfamilies (see below). The taxa *Teleosaurus* and *Platysuchus* are each other’s closest relatives, with a Bremer support value of 2 and jackknife percentage of 54%. Interestingly, *Mycterosuchus*, *Aeolodon*, *Bathysuchus* and *Sericodon* form a distinct subfamily. *Bathysuchus* and *Sericodon* are sister taxa (Bremer support value of 3 and jackknife of 88%); *Aeolodon* is most closely related to *Sericodon*+*Bathysuchus*, and *Mycterosuchus* is most closely related to *Aeolodon*+*Bathysuchus*+*Sericodon*.

Within the second family (Family M) (Fig. 59A), there are multiple unresolved areas. *Seldsienean*, *Deslongchampsina* and *Charitomenosuchus* are unresolved from one another and are situated at the base of this clade (Bremer support value of 1 and jackknife of 66%). Most notably, there is a large polytomy including *Pr. heberti*, *Pr*. cf. *bouchardi*, *Neosteneosaurus*, *S. rostromajor*, *Andrianavoay*, *Lemmysuchus* and *Yvridiosuchus*, and Machimosaurini is not recovered as a monophyletic subgroup. However, when *S. rostromajor* is removed from the analysis (176 MPTs and 1,659 steps: CI = 0.405, RI = 0.844), Machimosaurini becomes a distinct group, with *Lemmysuchus*+*Yvridiosuchus* and *Machimosaurus* separated from *Neosteneosaurus*, *Pr. heberti*, *Pr*. cf. *bouchardi* and *Andrianavoay* (Fig. 59B). In addition, when both *S. rostromajor* and *Andrianavoay* are removed (167 MPTs, 1,659 steps: CI = 0.405, RI = 0.844), *Pr. heberti* and *Pr*. cf. *bouchardi* are unresolved from one another but separated from *Neosteneosaurus*, which by itself becomes most closely related to Machimosaurini. In all iterations (with or without the removal of *S. rostromajor* and *Andrianavoay*), the genus *Machimosaurus* forms its own subgroup, and relationships between the four species are mostly resolved. *Machimosaurus mosae* and *Mac. buffetauti* are unresolved from one another; and *Mac. rex* and *Mac. hugii* are sister taxa (with *Mac. mosae*+*Mac. buffetauti* being most closely related to them).

### Most parsimonious unweighted consensus—majority rules

A parsimonious majority rules topology was produced to evaluate if there were any major changes from the strict consensus. The overall interrelationships within Teleosauroidea are more resolved than in the strict consensus topology (Fig. 59C), particularly within Family M. In Family T (Fig. 59C), *I. kalasinensis* is most closely related to the remaining taxa, and *I. potamosiamensis* and the Chinese teleosauroid (IVPP V 10098) are sister taxa, with *Mystriosaurus* being most closely related to them.

In Family M (Fig. 59C), *Clovesuurdameredeor* is situated at the base of this group, in stark contrast to its initial positioning, and *Deslongchampsina*, *Charitomenosuchus* and *Seldsienean* are all separated. A new subfamily (consisting of *Pr. heberti*, *Pr*. cf. *bouchardi*, *Andrianavoay*, *Neosteneosaurus*, *S. rostromajor* and Machimosaurini) is clearly defined (100%), and *Deslongchampsina* is most closely related to this subfamily. *Proexochokefalos heberti* is most closely related to *Pr*. cf. *bouchardi*+*Neosteneosaurus*+*S. rostromajor*+*Andrianavoay*+Machimosaurini. *Proexochokefalos* cf. *bouchardi*, *Neosteneosaurus*, *S. rostromajor* and *Andrianavoay* are all unresolved from one another, and are most closely related to Machimosaurini. Unlike the strict consensus topology (when all taxa are included), Machimosaurini is relatively well-supported (73%); *Lemmysuchus* and *Yvridiosuchus* (unresolved from one another) are separate from *Andrianavoay*, *Neosteneosaurus* and *S. rostromajor*, and are at the base of Machimosaurini. *Machimosaurus buffetauti* and *Mac. mosae* are separated, with *Mac. mosae* being the more closely related to *Mac. rex* and *Mac. hugii* (which are sister taxa) than *Mac. buffetauti*. It is important to note that when *S. rostromajor* is removed from the majority rules consensus, there is no change to teleosauroid interrelationships.

### Most parsimonious weighted strict consensus

As outlined above, the analysis was run once more using extended implied weights (*k* = 12). Extended implied weights (EIWs) are often used to improve the quality and stability of the results, and are more beneficial for palaeontological datasets than implied weights, which only introduces bias against characters with too many missing scores (Goloboff, 2014). The New Technology search (engines tailored as above) with TBR branch swapping resulted in 47 MPTs and a score of 48.94448. Due to relative clarity in the results, this is the topology referred to when formally naming clades (see below).

**Figure 60:**
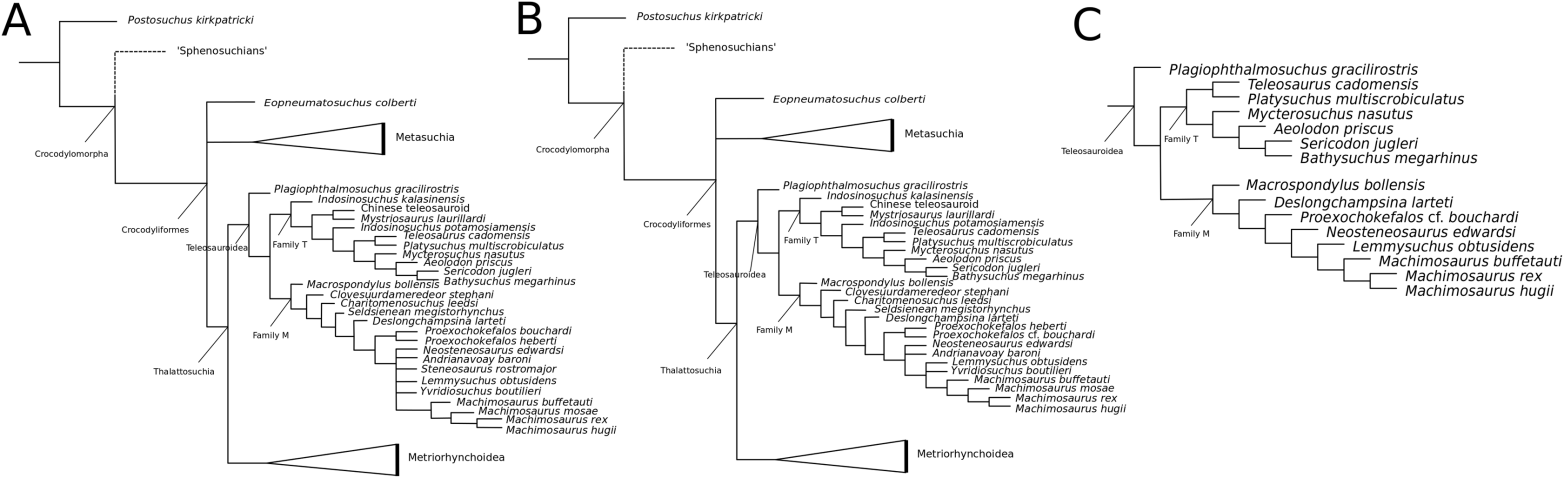
Topologies of the EIW parsimonious phylogenetic analysis, in addition to the maximum agreement subtree. Results of the extended weighted parsimonious phylogenetic analysis, focusing on Teleosauroidea. (A) Simplified strict consensus topology with extended implied weighting (*k* = 12) of the 47 MPTs; (B) simplified strict consensus topology with extended implied weighting (*k* = 12) excluding *S. rostromajor* (39 MPTs); and (C) agreement subtree (based on the unweighted strict consensus) of Teleosauroidea.

The results of the EIW analysis (Fig. 60A) show a more resolved Teleosauroidea than in the original strict consensus and is more similar regarding the majority rules topology. Teleosauroidea is monophyletic, *Plagiophthalmosuchus* is the basal-most teleosauroid, and the two families T and M are recovered. Family T is fully resolved (Fig. 60A), in contrast to both unweighted consensus topologies. Firstly, the Chinese teleosauroid (IVPP V 10098) and *Mystriosaurus* form sister taxa (although, surprisingly, there are no unambiguous synapomorphies to support this), with *I. kalasinensis* (situated at the base of this clade) being most closely related to them; in the majority rules topology, *I. potamosiamensis* was the sister taxon to the Chinese teleosauroid (IVPP V 10098). Here, *I. potamosiamensis* is positioned as most closely related to the *Teleosaurus*+*Platysuchus* subclade and subclade composed of *Mycterosuchus*+*Aeolodon*+*Bathysuchus*+*Sericodon*. *Teleosaurus* and *Platysuchus* are once again sister taxa, and they are most closely related to *Mycterosuchus* and pelagic relatives, which differs from the majority rules topology. The positioning of *Mycterosuchus*, *Aeolodon*, *Sericodon* and *Bathysuchus* are the same as all previous results:

1. *Sericodon* and *Bathysuchus* are sister taxa;
2. *Aeolodon* is most closely related to *Bathysuchus*+*Sericodon*; and
3. *Mycterosuchus* is most closely related to *Aeolodon*+*Bathysuchus*+*Sericodon*.

The majority of Family M is also clearly resolved (Fig. 60A), with slight changes from the majority rules topology:

1. *Macrospondylus*, rather than *Clovesuurdameredeor*, is the basal-most member of this clade; and
2. Notably, and surprisingly, Machimosaurini is not found to be monophyletic, with *Lemmysuchus* and *Yvridiosuchus* forming a polytomy with *Neosteneosaurus*, *S. rostromajor* and *Andrianavoay*. This is similar to the original consensus rather than the majority rules topology.

*Deslongchampsina* is once again found to be most closely related to the subfamily containing *Pr. heberti*, *Pr*. cf. *bouchardi*, *S. rostromajor*, *Andrianavoay* and Machimosaurini. *Proexochokefalos* cf. *bouchardi* and *Pr. heberti* are sister taxa, as in the majority rules topology. When *S. rostromajor* is removed, (Fig. 60B), the only change results in Machimosaurini being consistently recovered, as *Yvridiosuchus* and *Lemmysuchus* are separated from *Neosteneosaurus* and *Andrianavoay*. Interrelationships within *Machimosaurus* taxa were identical to the majority rules topology: *Mac. hugii* and *Mac. rex* are sister taxa, and *Mac. mosae* is most closely related to *Mac. hugii*+*Mac. rex* than *Mac. buffetauti*.

There are possible explanations as to why the tribe Machimosaurini remains unresolved from certain non-machimosaurins when all taxa are included. Firstly, both *S. rostromajor* and *Andrianavoay* are both represented by fragmentary skull material (and therefore scored for a low amount of characters), which may contribute to the lack of resolution. Another crucial factor is the lack of postcranial material for *Andrianavoay*, *S. rostromajor* and *Yvridiosuchus*; machimosaurins have a very distinct postcranium (Hua, 1999; Young et al., 2014; Johnson et al., 2017), which may influence the appearance of the topology. Thirdly, there are no autapomorphies observed in *S. rostromajor*, which is a poorly preserved section of undiagnostic rostrum (see Johnson, Young & Brusatte, 2020, for more information). This may contribute to the uncertainty of its placement as either an intermediate non-machimosaurin (e.g. *Neosteneosaurus*) or basal machimosaurin (e.g. *Yvridiosuchus*).

### Agreement subtree

The maximum agreement subtree (which chooses a subset of species with an equivalent restricted tree in all given evolutionary circumstances; Amir & Keselman, 1997), for Teleosauroidea was also produced (Fig. 60C) from the unweighted strict consensus: *Plagiophthalmosuchus* was recovered as the basal-most teleosauroid, and Families T and M were resolved. In Family T, *Teleosaurus+Platysuchus* and *Mycterosuchus*+*Bathysuchus+Aeolodon+Sericodon* were recovered as monophyletic subclades. In Family M, *Macrospondylus* was situated at the base and *Deslongchampsina* was most closely related to *Pr*. cf. *bouchardi + Neosteneosaurus* + Machimosaurini. Surprisingly, *Pr*. cf. *bouchardi* was recovered at most closely related to *Neosteneosaurus* + Machimosaurini. *Machimosaurus rex* and *Mac. hugii* were also recovered as sister taxa, and *Mac. buffetauti* was most closely related to them. *Lemmysuchus* was situated at the base of Machimosaurini, with *Neosteneosaurus* as the closest relative. Therefore, the taxa identified as hypothetically responsible for poor resolution (not included in the agreement tree) were *Indosinosuchus*, *Mystriosaurus*, the Chinese teleosauroid, *Clovesuurdameredeor*, *Charitomenosuchus*, *Seldsienean*, *S. rostromajor*, *Andrianavoay*, *Pr. heberti*, *Yvridiosuchus* and *Mac. mosae*. This is logical, as most aforementioned taxa either are fragmentary, lack postcrania or are represented by a low number of specimens (excluding *Charitomenosuchus*). As mentioned previously, these are key factors that can lead to polytomies and lack of resolution in trees. However, it is interesting to note that *Pr*. cf. *bouchardi* is included in the agreement subtree as a stable taxon, even though it is a partial skull scored based off specimen photographs.

### Bayesian results

As mentioned previously, three repetitions of MrBayes were run using the following functions: (#1) standard (*rates = equal*); (#2), gamma distribution (*rates = gamma*); and (#3) gamma distribution with variability (*1set applyto = (1) coding = variable*). The standard Bayesian results (#1) are relatively similar to those found in the implied weighting parsimony topology (standard deviation = 0.015520; harmonic mean = −8131.53). Teleosauroidea is monophyletic, *Plagiophthalmosuchus* is the basal-most teleosauroid and both Families T and M are recovered. However, there are slight differences within both subclades. In Family T, *Platysuchus* and *Teleosaurus* (sister taxa) are unresolved with *Mycterosuchus*+relatives and the East Asian teleosauroids+*Mystriosaurus*, and the East Asian teleosauroids (much like in the strict consensus and majority rules topologies), and *I. potamosiamensis* is most closely related to the Chinese teleosauroid+*Mystriosaurus*. In Family M, *Pr*. cf. *bouchardi* and *Pr. heberti* are not sister taxa, but rather *Pr*. cf. *bouchardi* is found to be most closely related to *Neosteneosaurus*+*Andrianavoay*+*S. rostromajor*+Machimosaurini.

**Figure 61:**
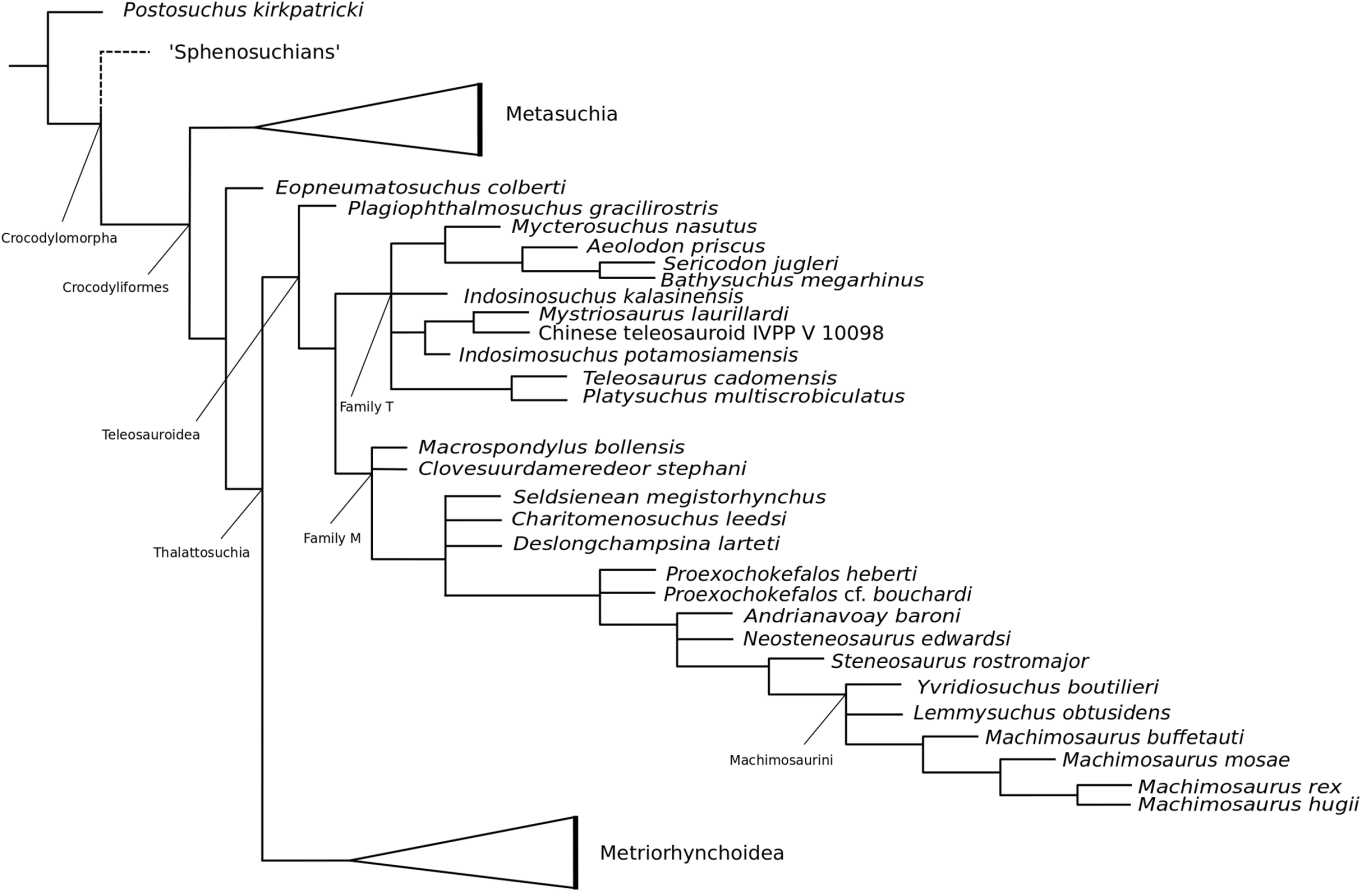
Simplified consensus topology in MrBayes. Simplified consensus topology, produced in MrBayes using gamma distribution (*rates = gamma*), standard deviation = 0.019863, harmonic mean = −7785.47. Note that *S. rostromajor* is recovered as most closely related to Machimosaurini.

In the gamma Bayesian test (#2), the results (standard deviation = 0.019863; harmonic mean = −7785.47) (Fig. 61) are similar to that seen in the standard Bayesian analysis, but with two differences:

1. *Charitomenosuchus*, *Seldsienean* and *Deslongchampsina* are in a polytomy; and
2. *Pr*. cf. *bouchardi* and *Pr. heberti* are in a polytomy.

**Figure 62:**
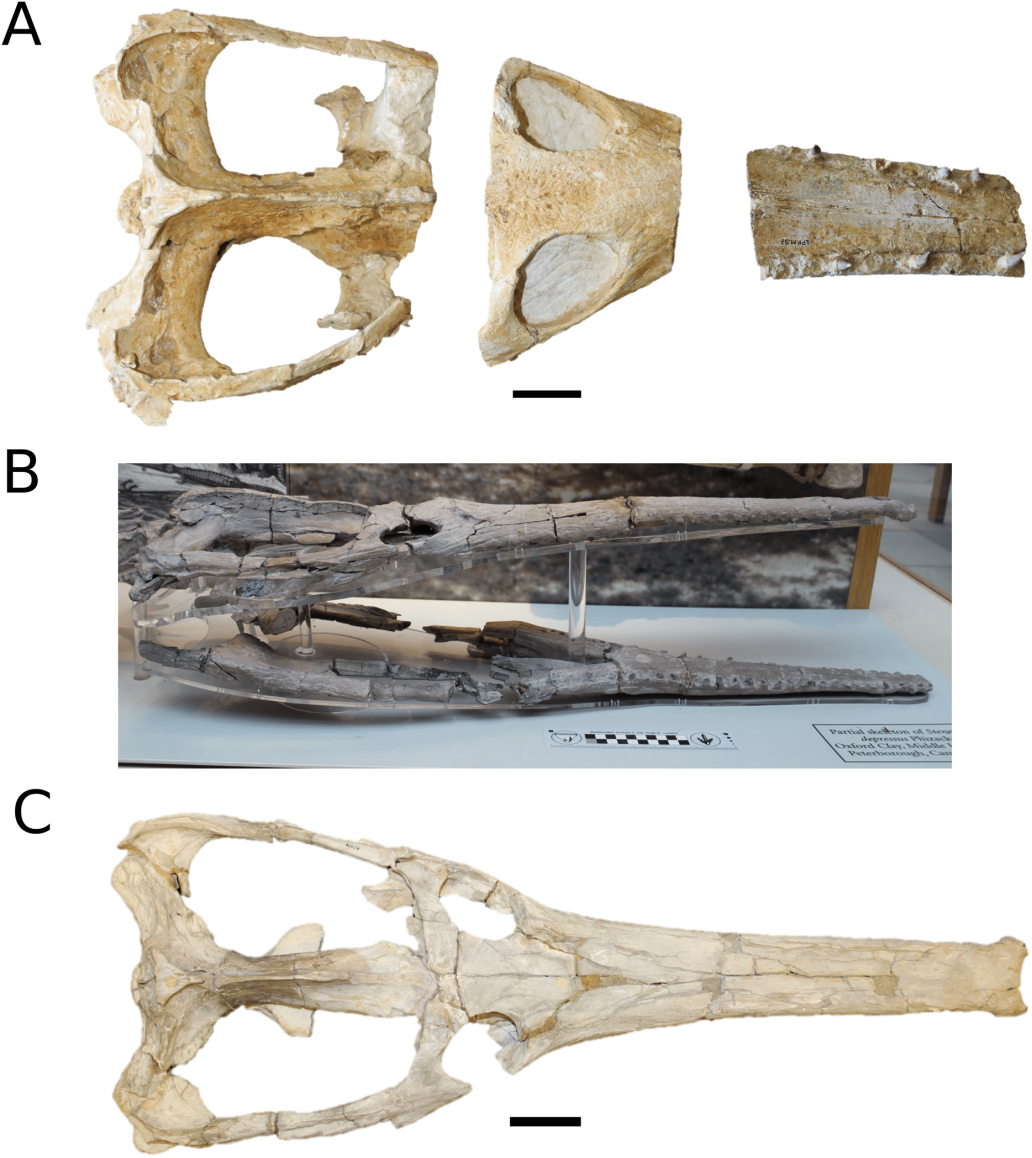
Three excluded teleosauroid taxa from our dataset. Photographs of three well preserved taxa not included in our dataset: (A) *Steneosaurus pictaviensis* (= *Charitomenosuchus leedsi*) LPP.M.37; (B) *Steneosaurus depressus* (= *Proexochokefalos heberti*) OUMNH J.01420; and (C) *Steneosaurus hulkei* (= *Neosteneosaurus edwardsi*) (NHMUK PV R 2074). See text for in-depth explanation as to why these taxa are excluded. Scale bars: 4 cm (A and C) and 10 cm (B).

The gamma variation MrBayes analysis (#3) (standard deviation = 0.017365; harmonic mean = −8130.41) produced a topology identical to that seen in the standard Bayesian analysis. In all Bayesian analyses, *S. rostromajor* is most closely related to Machimosaurini.

## Clades and Their Synapomorphies

Within this section, the synapomorphies uniting major clades are highlighted and discussed. A period and then the synapomorphic character state number follow the character numbers. We have decided to establish the clade names under both the ICZN Code and the International Code of Phylogenetic Nomenclature (hereafter referred to as the PhyloCode) to ensure nomenclatural stability. First the clade will be established under the ICZN Code, giving its diagnosis, then the clade will be established under the PhyloCode, giving its phylogenetic definition.

**Teleosauroidea Geoffroy Saint-Hilaire, 1831**

**Classification note.** Teleosauroidea is a ‘family group’ clade established under the ICZN Code, at the superfamily rank.

**Nominal authority.** The nominal authority is based on Article 36.1 of the ICZN Code (Principal of Coordination, applied to family group names).

**Description.** The superfamily Teleosauroidea is supported by multiple synapomorphies. These include absence of a sclerotic ring (163.0), postorbital medial to the jugal on the postorbital bar (173.0), straightened (sub-rectangular) anterior maxilla in palatal view (184.1), relatively reduced occipital tuberosities (203.1), paired ridges located on the medial ventral surface of the basisphenoid (223.1), a distinctly spatulate anterior dentary with the maximum width at the D3-D4 couplet (254.2), D3 occludes against the premaxillary-maxillary suture (331.0), coracoid with a fan-shape distal end and a triangular-shaped proximal end (402.1), a scapular blade as wide as or narrower than the glenoid region (405.1) and presence of caudal armour (493.0), as well as scoring the ‘pholidosaurid beak’ as inapplicable (47.-). One of these characters is new to the dataset, and another character (47) was re-written and re-scored. It is important to note that in teleosauroids, certain characters score differently than *Pelagosaurus* but are the same for other basal metriorhynchoids (e.g. *Teleidosaurus*). These include a slightly convex or flat frontal (121.0), a broadly curved anterior margin of the external mandibular fenestra (260.0), and well-defined apicobasally aligned ornamental ridges on the dentition (357.4),

**Comments.** Geoffroy Saint-Hilaire (1831: 34) initially defined teleosauroids (interpreted as ‘Teleosauridae’) as a distinct clade, referring to “*un cachet crocodilien*” (“a crocodilian character”). This suggests that he is describing the main features of teleosauroids, although he did not assign a name to this clade (Johnson, Young & Brusatte, 2020). He then proceeds to list the following features as definitive for the group:

1. Large ‘vertical holes’ (supratemporal fenestrae);
2. Vertically placed eyes;
3. A parietal bone that does not intervene between the jugal and temporal;
4. Two arches (“*l’une supérieure jugo-temporale, l’autre inférieure maxillo-tympanique*”: “one superior jugo-temporal, the other lower maxillofacial”);
5. Development of the nasal (cranio-respiratory) canal and temporal region; and
6. ‘Beak-like’ snout.

At the end of this description, Geoffroy Saint-Hilaire (1831: 37–38) writes ‘*Cette dernière combinaison remarquable dans les êtres téléosauriens devient des éléments caractéristiques pour une nouvelle famille; des éléments d’une puissance et d’une valeur à rendre en effet obligatoires les distinctions zoologiques de cette famille, c’est-à-dire l’érection des genres* Téléosaurus *et* Sténéosaurus’ (‘This last remarkable combination in teleosaurs becomes characteristic elements for a new family; elements of power and value to make compulsory the zoological distinctions of this family, that is to say the erection of the genera *Teleosaurus* and *Steneosaurus*’). Geoffroy Saint-Hilaire (1831: 37) considered ‘*la région supérieure et vers la fin de l’arrière-crâne; et d’autre part le museau*’ (‘the upper region and towards the end of the back of the skull; and (on the other hand) the snout’), along with ‘*le canal nasal et le palais*’ (‘the nasal canal and the palat’), to be the most important features when distinguishing teleosauroid species. After Geoffroy Saint-Hilaire’s (1831) work, teleosauroids continued to be traditionally grouped together based on their ‘longirostrine’ skull, dorsally directed orbits and high tooth count (Karl et al., 2008; Young & Andrade, 2009; Ballell et al., 2019). However, recent studies (Young et al., 2014; Foffa et al., 2019; Sachs et al., 2019a) have shown that there is more variation in the teleosauroid cranium than initially thought, and the shape of the skull and number of teeth cannot purely be relied on to define this clade.

***Teleosauroidea Young & Andrade, 2009 nomen cladi conversum***

**Phylogenetic definition.** The largest clade within Thalattosuchia containing *Teleosaurus cadomensis*, but not *Metriorhynchus geoffroyii* Von Meyer, 1832. Young & Andrade (2009) initially defined the superfamily Teleosauroidea as the most inclusive clade consisting of *Teleosaurus cadomensis*, but not *Metriorhynchus geoffroyii*. This is a maximum-clade, or stem-based, definition.

**Figure 63:**
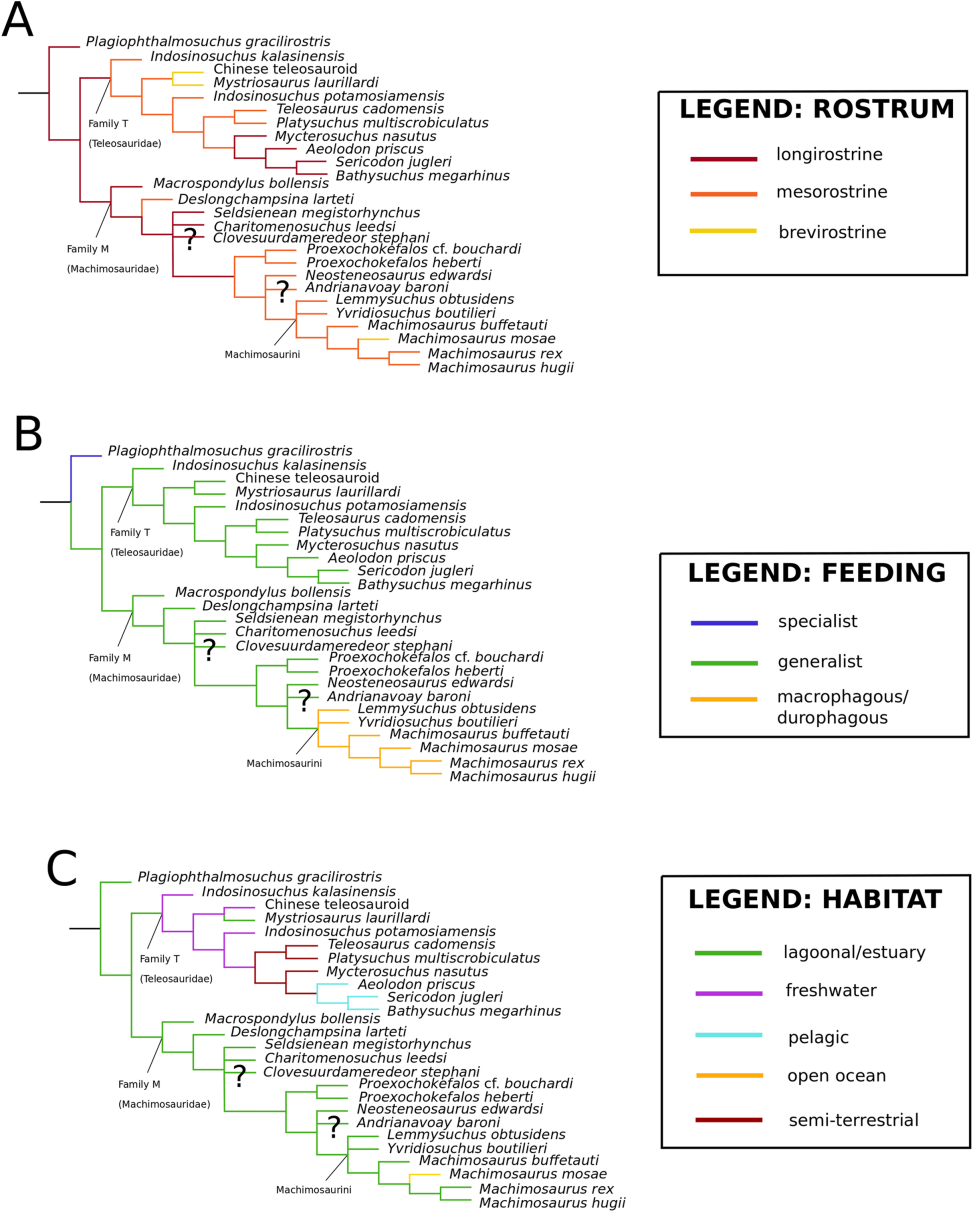
Teleosauroid hypothesised ecomorphologies indicated on the extended implied weighted topology. Hypothesized teleosauroid ecomorphologies mapped onto the extended implied weighted topology (excluding *Steneosaurus rostromajor*: 39 MPTs): (A) rostral morphology; (B) feeding ecology; and (C) palaeohabitat. Note that Family T is more phenotypically plastic than Family M in terms of (A) rostrum and (C) habitat, and that Family M shows a distinctive, linear shift in (A) rostral length and (B) feeding style.

**Figure 64:**
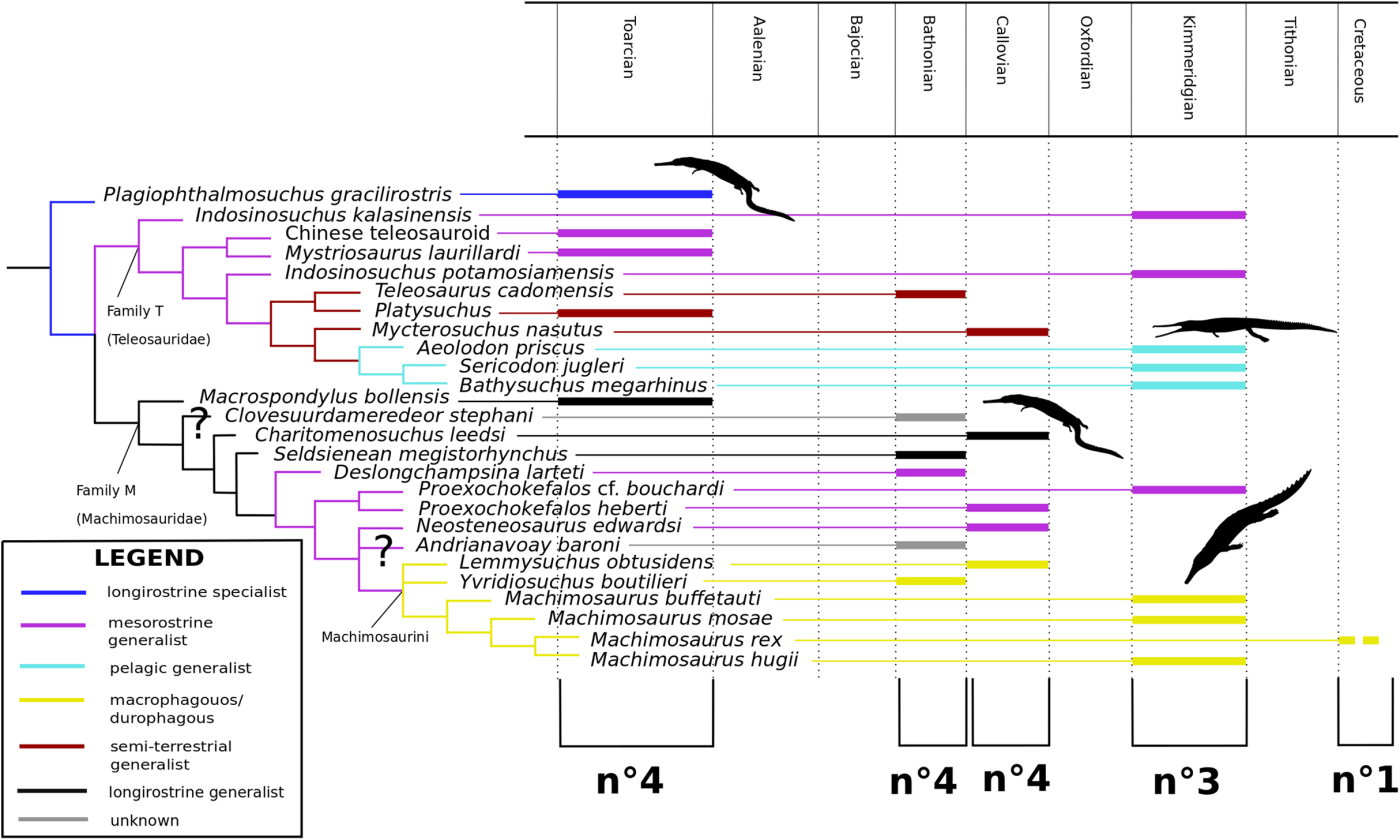
Time calibrated tree. Summary of time-calibrated phylogeny (extended implied weighting excluding *Steneosaurus rostromajor*: 39 MPTs) of teleosauroids, focusing on number (n°) of ecomorphological guilds present during four main time periods (Toarcian, Bathonian, Callovian and Kimmeridgian). Major guilds are as follows: dark blue = longirostrine specialist; purple = mesorostrine generalist; light blue = pelagic generalist; black = longirostrine generalist; yellow = macrophagous/durophagous; red = semi-terrestrial generalist. Grey coloured lines indicate unknown ecomorphology, due to incomplete material. Note that the number of guilds remains constant (four) until the Kimmeridgian, in which there is a drop (three). Silhouettes provided by PhyloPic (by G. Monger, S. Hartman and N. Tamara).

**Reference phylogeny.** Phylogenetic analyses presented herein, see Figs. 59–61, 63 and 64.

**Composition.** *Plagiophthalmosuchus*, Teleosauridae (comprising of the Chinese teleosauroid, *Indosinosuchus*, *Mystriosaurus*, Teleosaurinae (comprising of *Platysuchus* and *Teleosaurus*)), and Aeolodontinae (comprising of *Aeolodon*, *Bathysuchus*, *Mycterosuchus* and *Sericodon*) and Machimosauridae (comprising of *Charitomenosuchus*, *Clovesuurdameredeor*, *Deslongchampsina*, *Macrospondylus*, *Seldsienean* and Machimosaurinae (comprising *Andrianavoay*, *Neosteneosaurus*, *Proexochokefalos* and Machimosaurini (comprising *Lemmysuchus*, *Machimosaurus* and *Yvridiosuchus*))).

**Diagnostic apomorphies.** 47.-; 163.0; 173.0; 184.1; 203.1; 223.1; 254.2; 331.0; 402.1; 405.1; 493.0. (From the dataset herein, the same characters as the ICZN Code description.)

**Teleosauridae (Family T) Geoffroy Saint-Hilaire, 1831**

**Classification note.** Teleosauridae is a ‘family group’ clade established under the ICZN Code, at the family rank.

**Original Definition Comment.** ‘Teleosauridae’ was originally erected and defined by Geoffroy Saint-Hilaire (1825, 1831) and encompassed all teleosauroid species (as discussed above). However, herein Teleosauridae is restricted to the following taxa: the genus *Indosinosuchus*, *Mystriosaurus laurillardi*, *Teleosaurus cadomensis*, *Platysuchus multiscrobiculatus*, *Aeolodon priscus*, *Mycterosuchus nasutus*, *Sericodon jugleri*, *Bathysuchus megarhinus* and the Chinese teleosauroid (IVPP V 10098).

**Description.** A number of synapomorphies supports the monophyly of Teleosauridae. These include anteriorly or anterodorsally oriented external nares (34.0), anterior and anterolateral premaxillary margins that are anteroventral and extend ventrally (48.1), supratemporal fenestrae with noticeably inclined anterior margins (103.1), postorbital overlapping the jugal (158.1), a horizontal pterygoid flange (198.0) and the basisphenoid terminates at the anterior quadrates (225.0).

***Teleosauridae Geoffroy Saint-Hilaire, 1831 nomen cladi conversum***

**Phylogenetic definition**. The largest clade within Teleosauroidea containing *Teleosaurus cadomensis*, but not *Plagiophthalmosuchus gracilirostris* and *Machimosaurus hugii*. This is a maximum-clade, or stem-based, definition.

**Reference phylogeny.** Phylogenetic analyses presented herein, see Figs. 59–61, 63 and 64.

**Composition.** The Chinese teleosauroid, *Indosinosuchus*, *Mystriosaurus*, Teleosaurinae (comprising of *Platysuchus* and *Teleosaurus*) and Aeolodontinae (comprising of *Aeolodon*, *Bathysuchus*, *Mycterosuchus* and *Sericodon*).

**Diagnostic apomorphies.** 34.0; 48.1; 103.1; 158.1; 198.0; 225.0. (From the dataset herein, the same characters as the ICZN Code description.)

**Unnamed clade: the Chinese teleosauroid IVPP V 10098 + *Mystriosaurus laurillardi***

**Comments.** Interestingly, there are no unambiguous synapomorphies that unite this clade, despite its stable position within the weighted parsimonious analysis (Figs. 60A and 60B). This unnamed clade shares one character with *Neosteneosaurus* and machimosaurins (nasals and maxillae are not elongated: 6.0) and one character with *Mac. buffetauti* and *Mac. mosae* (anteroposterior premaxillary length is less than 25% of total rostrum length: 43.0).

**Teleosaurinae Vignaud, 1995 (*Teleosaurus* + *Platysuchus*)**

**Classification note.** Teleosaurinae is a ‘family group’ clade established under the ICZN Code, at the subfamily rank.

**Nominal authority.** The nominal authority is based on Article 36.1 of the ICZN Code (Principal of Coordination, applied to family group names).

**Description.** The subfamily Teleosaurinae consists of the genera *Platysuchus* and *Teleosaurus*, and there are four characters that unite them as sister taxa. These include both the tooth row and quadrate condyle being below the level of the occipital condyle but are unaligned with the tooth row at a lower level (2.5), the frontal-postorbital suture is lower than the intertemporal bar (131.1), densely distributed osteoderms with small round to ellipsoid pits (473.0), and presacral dorsal osteoderms are strongly curved (480.1).

**Comments.** Vignaud (1995) initially diagnosed the subfamily Teleosaurinae as that containing *Platysuchus* and all *Teleosaurus* taxa. Here, *Teleosaurus* is currently limited to just one species, but follows the same proposal put forth in Vignaud (1995), in that *Platysuchus* is most closely related to *Teleosaurus*.

***Teleosaurinae Vignaud, 1995 nomen cladi conversum***

**Phylogenetic definition.** The largest clade within Teleosauroidea containing *Teleosaurus cadomensis*, but not *Aeolodon priscus* and *Indosinosuchus potamosiamensis*. This is a maximum-clade, or stem-based, definition.

**Reference phylogeny.** Phylogenetic analyses presented herein, see Figs. 59–61, 63 and 64.

**Composition.** *Platysuchus* and *Teleosaurus*.

**Diagnostic apomorphies.** 2.5; 131.1; 473.0; 480.1. (From the dataset herein, the same characters as the ICZN Code description.)

**Aeolodontinae subfam. nov. (*Mycterosuchus* + *Aeolodon* + *Bathysuchus* + *Sericodon*)**

**Classification note.** Aeolodontidae is a ‘family group’ clade established under the ICZN Code, at the subfamily rank. urn:lsid:zoobank.org:act:E7A8EDC8-8DF8-4799-AA09-5D6B9287E201.

**Description.** A number of synapomorphies, notably in the premaxilla, supports the subfamily Aeolodontinae, which includes the genera *Mycterosuchus*, *Aeolodon*, *Sericodon* and *Bathysuchus*. These include an ‘8’shaped premaxilla in anterior view (56.1), reduced basioccipital tuberosities (230.0), laterally oriented P1 and P2 (294.2), P1 and P2 do not form a couplet but are situated on the anterior margin of the premaxilla (295.1), P1 and P2 are both on the same transverse plane (298.1) and the anterior margin between the P2-P3 is sub-rectangular, with the P3 being clearly lateral to the P2 (299.1). Four out of six characters are new to this dataset.

**Comments.** Aeolodontinae is also always recovered as a monophyletic subclade, regardless of changing taxa and/or character scores and whether the dataset is run using parsimony or Bayesian criteria. It is interesting to note that, while similar in many aspects concerning the skull (namely the premaxillae), the postcranial material of *Mycterosuchus* differentiates vastly from other members of the group. For example, the proximal humerus is very strongly posteriorly deflected and hooked in *Aeolodon*, similar to members of Machimosauridae (e.g. *Charitomenosuchus*, *Neosteneosaurus*). In *Mycterosuchus*, the proximal humerus is also hooked, but weakly so, and is more club-shaped. The tuberculum and articular facet of the largest dorsal ribs are positioned directly in the middle, which is more similar to *Charitomenosuchus* and opposed to the medial edge position in *Aeolodon*. Other unique postcranial features to *Mycterosuchus* include a longer ulna than radius, an elongated pubic shaft, an enlarged anteromedial femoral tuber and the calcaneal tuber being approximately 25% larger than the astragalus (as discussed above). It is likely that the unique skull characteristics of these taxa are what is supporting this subfamily as monophyletic.

While postcranial materials of *Aeolodon* are well preserved in both specimens (NHMUK PV R 1086 and MNHN.F.CNJ 78), and partially preserved in *Sericodon* (see Schaefer, Püntener & Billon-Bruyat, 2018), it is important to note that there are no postcranial bones of *Bathysuchus* currently recorded. A full, comprehensive comparison of the postcrania of *Aeolodon* and *Sericodon* is essential, to examine if *Sericodon* possesses a reduced appendicular skeleton similar to that seen in *Aeolodon*, which has been hypothesized to be more pelagic than other teleosauroids (see below, as well as Foffa et al. (2019)).

***Aeolodontinae nomen cladi novum***

**Registration number.** urn:lsid:zoobank.org:act:E7A8EDC8-8DF8-4799-AA09-5D6B9287E201.

**Phylogenetic definition.** The largest clade within Teleosauroidea containing *Aeolodon priscus* but not *Indosinosuchus potamosiamensis* and *Teleosaurus cadomensis*. This is a maximum-clade, or stem-based, definition.

**Reference phylogeny.** Phylogenetic analyses presented herein, see Figs. 59–61, 63 and 64.

**Composition.** *Aeolodon*, *Bathysuchus*, *Mycterosuchus* and *Sericodon*.

**Diagnostic apomorphies.** 56.1; 230.0; 294.2; 295.1; 298.1; 299.1. (From the dataset herein, the same characters as the ICZN Code description.)

**Unnamed clade: *Aeolodon* + *Bathysuchus* + *Sericodon***

**Comments.** Interestingly, there are no unambiguous synapomorphies that unite this clade, despite its stable position within the above analyses. This unnamed clade shares two characters with *Plagiophthalmosuchus* and *I. potamosiamensis*: no ornamentation on prefrontal (12.1) and lacrimal (13.1); and one character with *Charitomenosuchus*, *Seldsienean*, *Deslongchampsina* and Machimosaurinae (see below): frontal ornamentation restricted to the centre of the bone (15.1).

**Unnamed clade: *Sericodon* + *Bathysuchus***

**Synapomorphies.** 296.1; 339.1.

**Comments.** *Sericodon* and *Bathysuchus* are united by two characters: a strong lateral expansion of the premaxillae so that P3 and P4 are aligned on the lateral plane of the external margin (296.1) and presence of carinae on the apical third of the tooth (339.1). Despite only two dental synapomorphies, *Sericodon* and *Bathysuchus* are recovered as sister taxa in all analyses.

**Machimosauridae Jouve et al., 2016 fam. nov. (Family M)**

**Classification note.** Machimosauridae is a ‘family group’ clade established under the ICZN Code, at the family rank. urn:lsid:zoobank.org:act:81FB2470-D7E7-4E3E-814B-2AAE996BE5AA.

**Nominal authority.** The nominal authority is based on Article 36.1 of the ICZN Code (Principal of Coordination, applied to family group names).

**Description.** The family Machimosauridae is united by a number of characters; these include the dorsally oriented external nares (34.1), the premaxillary anterior and anterolateral margins are not sub-vertical and do not extend ventrally (48.0), the premaxilla-maxilla suture is sub-rectangular and slightly interdigitating (most noticeably near the midline) (58.1), no anterolateral expansion of the supratemporal fenestrae (103.0), the postorbital excluded from the orbit posteroventral margin (158.0), mostly horizontal pterygoid with a distinct posterolateral angle (198.1) and cultriform process of the basisphenoid exposed and bifurcates the pterygoids (225.1).

***Machimosauridae nomen cladi novum***

**Registration number.** urn:lsid:zoobank.org:act:81FB2470-D7E7-4E3E-814B-2AAE996BE5AA.

**Phylogenetic definition.** The largest clade within Teleosauroidea containing *Machimosaurus hugii*, but not *Plagiophthalmosuchus gracilirostris* and *Teleosaurus cadomensis*. This is a maximum-clade, or stem-based, definition.

**Reference phylogeny.** Phylogenetic analyses presented herein, see Figs. 59–61, 63 and 64.

**Composition.** *Charitomenosuchus*, *Clovesuurdameredeor*, *Deslongchampsina*, *Macrospondylus*, *Seldsienean* and *Machimosaurinae* (comprising *Andrianavoay*, *Neosteneosaurus*, *Proexochokefalos* and *Machimosaurini* (comprising *Lemmysuchus*, *Machimosaurus* and *Yvridiosuchus*)).

**Diagnostic apomorphies.** 34.1; 48.0; 58.1; 103.0; 158.0; 198.1; 225.1. (From the dataset herein, the same characters as the ICZN Code description.)

**Machimosaurinae Jouve et al., 2016 subfam. nov. ( *Proexochokefalos* + *Andrianavoay* + *Neosteneosaurus* + Machimosaurini)**

**Classification note.** Machimosaurinae is a ‘family group’ clade established under the ICZN Code, at the subfamily rank. urn:lsid:zoobank.org:act:918AC1F1-AC04-41D1-91DA-E2D25939EAB9.

**Nominal authority.** The nominal authority is based on Article 36.1 of the ICZN Code (Principal of Coordination, applied to family group names).

**Description.** The subfamily Machimosaurinae is supported by a handful of characters including the supratemporal fenestra length being twice as long as the width (104.1), a shallow Meckelian groove (269.1), a sharply curved angular (270.1) and non-procumbent dentition throughout the entirety of the jaws (325.0). Two of these characters are new to the dataset.

***Machimosaurinae nomen cladi novum***

**Registration number.** urn:lsid:zoobank.org:act:918AC1F1-AC04-41D1-91DA-E2D25939EAB9.

**Phylogenetic definition.** The largest clade within Teleosauroidea containing *Machimosaurus hugii* but not *Deslongchampsina larteti* and *Charitomenosuchus leedsi*. This is a maximum-clade, or stem-based, definition.

**Reference phylogeny.** Phylogenetic analyses presented herein, see Figs. 59–61, 63 and 64.

**Composition.** *Andrianavoay*, *Neosteneosaurus*, *Proexochokefalos* and Machimosaurini (comprising *Lemmysuchus*, *Machimosaurus* and *Yvridiosuchus*).

**Diagnostic apomorphies.** 104.1; 269.1; 270.1; 325.0. (From the dataset herein, the same characters as the ICZN Code description.)

**Features uniting the genus *Proexochokefalos***

**Synapomorphies.** 66.0.

**Comments.** The sole character supporting *Proexochokefalos heberti* and *Proexochokefalos* cf. *bouchardi* as sister taxa is the lack of a midline cavity (= trench) on the nasals, instead being flat (66.0).

**Machimosaurini Jouve et al., 2016 (*Yvridiosuchus* + *Lemmysuchus* + *Machimosaurus*)**

**Classification note.** Machimosaurini is a ‘family group’ clade established under the ICZN Code, at the tribe rank.

**Description.** A number of character states support the monophyly of Machimosaurini. These include parallelogram-shaped supratemporal fenestrae (102.5), blunt apices (327.1), no curvature in the middle to posterior dentition (345.0), false ziphodont serrations restricted to posteriorly-placed tooth crowns (349.2), incipient ziphodont carinae (351.2), rounded true denticles (352.1), all teeth microziphodont (353.1), strongly developed anastomosed pattern on the apices (358.1), three sacral vertebrae (379.1), sub-square ischial plate (449.1), ventrally angled tibial tuberosity (464.1), and keeled osteoderms with variable and elongated pits (473.3). Two of these characters are new to the dataset. Jouve et al. (2016) initially described the tribe Machimosaurini based on the following characteristic features: (1) shortened rostra; (2) enlarged supratemporal fenestrae; (3) reduced tooth counts; and (4) blunt, ornamented dentition.

**Comments.** Certain characteristics of machimosaurins, particularly their teeth, have been documented for many years; *Mac. hugii* was first described by von Meyer in 1837, who made a particular comment about the dentition: ‘*…stumpfkonischen und dicht gestreiften Zähnen besonders charakteristisch herauszustellen…*’ (‘…particularly (conspicuous in) conical and densely striped teeth…’) (Von Meyer, 1837: 560). Sauvage & Liénard (1879: 7) noted ‘*L. forme des vertèbres, la disposition des écussons, la composition de la tête (…), la forme et l’ornamentation des dents…*’ (‘The shape of the vertebrae, the arrangement of the osteoderms, the composition of the head (…), the shape and ornamentation of the teeth…’) when describing *Mac. mosae*. Phillips (1871: 184–185) also defined the teeth of *Yvridiosuchus* (known then as *Teleosaurus brevidens*; see Johnson, Young & Brusatte, 2019) as ‘…rather short (teeth)…a little curved, uniformly striated, the striae growing more prominent toward the point and finer toward the base… (a) slight trace of bicarination on these teeth, near the apex, which is usually blunt…’; he appears to be referring to the anastomosing pattern. Andrews (1913: 132), made note of the third sacral vertebra in *Lemmysuchus*, saying ‘…a remarkable condition is found, there being apparently three sacrals… (seems to be) that the ribs of the first caudal have greatly enlarged and resemble sacral ribs…’ However, Andrews (1913) thought this to be a unique feature in *Lemmysuchus*, not taking into context the same condition seen in species of *Machimosaurus*.

Recent papers have also highlighted several of these features, including: detailed descriptions of the dentition (Young & Steel, 2014; Young et al., 2015a; Jouve et al., 2016); specific features of the skull (Hua, 1996; Young et al., 2014; Fanti et al., 2016; Johnson et al., 2017; Johnson, Young & Brusatte, 2019); reduction in the pelvic bones (Johnson et al., 2017); and the unique sacral anatomy (Martin & Vincent, 2013; Young et al., 2014; Johnson et al., 2017).

***Machimosaurini Jouve et al., 2016, nomen cladi conversum***

**Phylogenetic definition.** The largest clade within Teleosauroidea containing *Machimosaurus hugii*, but not *Neosteneosaurus edwardsi*. This is a maximum-clade, or stem-based, definition.

**Reference phylogeny.** Phylogenetic analyses presented herein, see Figs. 59–61, 63 and 64.

**Composition.** *Lemmysuchus*, *Machimosaurus* and *Yvridiosuchus*.

**Diagnostic apomorphies.** 102.5; 327.1; 345.0; 349.2; 351.2; 352.1; 353.1; 358.1; 379.1; 449.1; 464.1; 473.3. (From the dataset herein, the same characters as the ICZN Code description.)

**Features uniting the genus *Machimosaurus***

**Unambiguous Synapomorphies.** 7.0.

**Ambiguous Synapomorphies.** 32.0; 288.3; 292.-; 293.-; 294.-; 297.-; 300.-; 395.{01}; 406.1.

**Comments.** There are multiple features unique to the genus *Machimosaurus*; however, there is only one definitive character that is preserved in all species: a wider than higher rostrum (7.0). All ambiguous synapomorphies are found in both *Mac. buffetauti* and *Mac. mosae*, but are scored as (?) in *Mac. hugii* and *Mac. rex* due to lacking or fragmentary material. These synapomorphies include simple, straight-lined dentary neurovascular foramina (32.0), three premaxillary alveoli (288.3), the tuberculum and articular facet of dorsal ribs positioned halfway in the middle (395.{01}), scapula with a strongly concave anterior edge (406.1), and inapplicability of ch. 292–294, 297 and 300.

## Discussion

### Areas of uncertainty

The above analyses, similar to recent studies (Ősi et al., 2018; Foffa et al., 2019; Johnson, Young & Brusatte, 2019; Sachs et al., 2019a), find many aspects of the phylogeny to be consistent, including:

1. *Plagiophthalmosuchus gracilirostris* as the basal-most teleosauroid;
2. The recovery of two well defined families (Teleosauridae and Machimosauridae); and
3. The tribe Machimosaurini is situated within Machimosauridae.

Using our updated dataset, we consistently recover the subfamilies Teleosaurinae and Aeolodontinae, regardless of changes and/or additions to the dataset. However, positions of certain taxa regularly change. For example *Pr*. cf. *bouchardi* is recovered as unresolved with other members of Machimosaurinae in the strict consensus topology; however, in the extended implied weighting topologies it is recovered as the sister taxon to *Pr. heberti*. With these degrees of uncertainty, the addition of new characters and teleosauroid taxa has only caused greater ambiguity in certain areas of the tree (especially in the unweighted consensus analysis). While it is undoubtedly important to carefully study, re-analyse and re-describe specimens, and discover new character data, the addition of new characters may not be the key in resolving these issues.

More importantly, one of the major problems is that a single specimen, usually skull material, represents many of these species, such as the Chinese teleosauroid (IVPP V 10098), *Pr. heberti*, *Clovesuurdameredeor* and *Andrianavoay*. In some cases, these specimens are well preserved and offer vital information (e.g. *Pr. heberti*), but there are certain ones that may be key intermediate forms but are too fragmentary to offer any substantial data (e.g. *Andrianavoay*). One contributing factor is that very little fossil prospection is taking place in localities where many of these specimens have been found (e.g. Toarcian outcrops in China, Bathonian locations in Madagascar, Upper Jurassic sites in Thailand). In addition, there are vast areas, particularly along the Gondwanan coasts of Africa and India, which have yielded promising material but have yet to be prospected properly (Phansalkar, Sudha & Khadkikar, 1994; Dridi & Johnson, 2019). This represents a unique opportunity for future work, and the discovery of additional material for existing species will offer a greater resolution into teleosauroid evolution during the Middle to Upper Jurassic and into the Lower Cretaceous.

### Excluded taxa

Certain taxa were omitted from our analysis because (1) the holotype was either destroyed or could not be located or (2) said taxa did not possess any other current substantial material. For example, *Machimosaurus nowackianus*, a specimen comprising of the anterior dentary from Ethiopia, was reported being housed in the GPIT in Tübingen (Young et al., 2014). After its initial description, many researchers attempted to locate it within the collection and were unable (recently, it has been reported as returned from loan in March 2017: R. Irmis, 2019, personal communication). There is one available photograph of the specimen (Young et al., 2014, from Von Huene (1938) fig. 1–4); however, it was shown only in a slightly blurred dorsal view, but more importantly, due to the sheer incompleteness of the specimen and lack of characteristic features, we omitted this taxon from our dataset.

The taxon *Steneosaurus deslongchampsianus* Lennier, 1887, was excluded from our dataset because the holotype (comprising of skull and mandibular material) was destroyed in 1944 (Vignaud, 1995), and there was no other definitive existing material for this particular taxon; currently, line drawings are the only source of information available (see Saville, 1876; Lennier, 1887). While these are invaluable for research, we were wary to score an entire taxon using only drawings; there are many instances (especially during the 19th and early 20th centuries) where figures were either altered, drawn to include missing skeletal elements, or interpreted as similar to other taxa (Andrews, 1913). The holotype of *Teleosaurus geoffroyi* Eudes-Deslongchamps, 1868c was based on three mandibular fragments, which J.A. Eudes-Deslongchamps considered distinct due to ‘*…un nombre sensiblement inférieur de dents*’ (‘…a significantly lower number of teeth’) than *T. cadomensis* (Vignaud, 1995: 181). However, this specimen (now considered an objective junior synonym of *T*. *cadomensis*: see Jouve, 2009) was also destroyed in 1944, and this distinguishing feature cannot be confirmed. In addition, two taxa were disregarded due to specimens simply being too fragmentary. First, the holotype of *Steneosaurus rudis* Sauvage, 1874 consisted of fragmentary pieces of the skull and mandible; it was part of the BHN2R collection, which was later closed in 2003, and it went missing. However, Vignaud (1995) suggested that, due to the robustness of the specimen, it could be referred to as *Machimosaurus* sp. The second example is *Steneosaurus roissyi* Eudes-Deslongchamps, 1869 (MNHN.RJN 130a-c), which consists of a fragmentary piece of the mandible; this material has no distinguishing characteristics and is therefore more apt to be referred to as Teleosauroidea indeterminate.

Three teleosauroid taxa with a considerable amount of material were not included in our analyses. The first is *Steneosaurus pictaviensis* (Fig. 62A). Vignaud (1998: 30–31) described the holotype (LPP.M.35; although this specimen is labelled as LPP.M.37 in collections) and paratype (LPP.M.37, although this is labelled as LPP.M.35 in collections) as being different from *Steneosaurus* (= *Charitomenosuchus*) *leedsi* in that:

1. No antorbital fenestrae (only an underlying depression) were present in *S. pictaviensis*;
2. The maxillae were “*plus élevés*” (“higher than”) *C. leedsi*; and
3. The interalveolar surface of the dentary was smooth and “*sans les deux sillons longitudinaux*” (“without the two longitudinal furrows”), unlike *C. leedsi*.

However, these characters are erroneous; firstly, in *C. leedsi* (NHMUK PV R 3320; NHMUK PV R 3806; BRLSI GP1770a-e), the antorbital fenestrae are very small, shallow and depression-like. In LPP.M.37, there is a small depression where the antorbital fenestrae should be located, similar to *C. leedsi*. Secondly, the crania of many *C. leedsi* specimens (e.g. NHMUK PV R 3320; NHMUK PV R 3806; PETMG R179) are dorsoventrally crushed, so the maxillae appear to be low; however, BRLSI GP1770a-e is three-dimensionally preserved, with the maxillae dorsoventrally high as in LPP.M.37. Lastly, it is unclear what longitudinal furrows Vignaud (1998) was referring to in *C. leedsi*; the interalveolar surface of the dentary (NHMUKL PV R 3320; NHMUK PV R 3806) is smooth, with anteriorly prominent lateral crenulations similar to LPP.M.35. If Vignaud (1998) was referring to the coronoid processes protruding into the dentary, these are quite large in both LPP.M.35 and *C. leedsi* (NHMUK PV R 3320). In addition, LPP.M.35 and LPP.M.37 are comparable to *C. leedsi* (NHMUK PV R 3320; NHMUK PV R 3806) in the following:

1. Frontal with few, circular pits that are largely concentrated in the centre of the bone;
2. Mediolaterally thin posterior processes of the nasals (similar to *T. cadomensis*);
3. Sub-rectangular supratemporal fenestrae;
4. Slender teeth with pointed apices and faint enamel ornamentation; and
5. All referred specimens are middle Callovian in age and are found in corresponding stratigraphic horizons.

Therefore, we consider *S. pictaviensis* as a subjective junior synonym of *C. leedsi*.

The second taxon is *Steneosaurus depressus* Phizackerley, 1951 (OUMNH J.01420) (Fig. 62B). Phizackerley (1951) defined this a distinct species based on the following features: (1) the delicately constructed skull; (2) a slender, rounded rostrum comprising 64% of the total skull length; (3) small orbits; (4) small, slender, curved teeth; and (5) mandibular symphysis occupying roughly 48% of the entire mandible. However, these features can be attributed to sub-adult specimens or are found in other teleosauroid taxa. In addition, OUMNH J.01420 shares the following combination of key characteristics seen in *Pr. heberti* (MNHN.F 1890-13):

1. Enlarged occipital tuberosities (differs from all other members of Teleosauroidea);
2. No antorbital fenestrae;
3. Elongated, slender anterior process of the jugal; and
4. The P1 is oriented anteriorly and the P2 is oriented slightly medially (differs from *Neosteneosaurus* NHMUK PV R 3701).

Therefore, *S. depressus* can tentatively be referred to as a subjective junior synonym of *Pr. heberti*. However, a thorough re-description of both specimens is needed and is beyond the scope of this article.

The final taxon, *Steneosaurus hulkei* (NHMUK PV R 2074) (Fig. 62C), was excluded from our dataset as its holotype likely represents a sub-adult individual. The vertebral neurocentral suture is visibly prominent in young modern crocodylians and gradually closes and disappears in adults, in the direction from the caudals to the cervicals (Brochu, 1996). In the *S. hulkei* holotype, the neurocentral sutures are clearly visible and well-developed in the posterior thoracic vertebrae, suggesting it was a juvenile or sub-adult. In addition, *S. hulkei* displays a mixture of features similar to those seen in *Neosteneosaurus* (NHMUK PV R 2865; PETMG R178) and differs from *Charitomenosuchus* (NHMUK PV R 3320, NHMUK PV R 3806) and *Lemmysuchus* (NHMUK PV R 3168), such as:

1. The cranium is overall more robust than *Charitomenosuchus* (NHMUK PV R 3320);
2. No antorbital fenestrae are present (differs from *Charitomenosuchus* (NHMUK PV R 3320, NHMUK PV R 3168) in which they are present);
3. A subcircular premaxilla-maxilla suture (differs from *Charitomenosuchus* (NHMUK PV R 3320), which has a strongly interdigitating, rectangular premaxilla-maxilla suture);
4. Dorsoventrally short supraoccipital (differs from *Lemmysuchus* (NHMUK PV R 3168) in which the supraoccipital is dorsoventrally tall);
5. Deep reception pits until the posterior region of the maxilla (differs from *Charitomenosuchus* (NHMUK PV R 3806) which has deep reception pits until the mid-maxilla, and *Lemmysuchus* (NHMUK PV R 3168) which has deep reception pits along the entirety of the maxilla);
6. Straightened posteriorly placed cervical ribs (differs from *Lemmysuchus* (NHMUK PV R 3168) which has a curved posteriorly placed cervical rib);
7. Triangular-shaped ischial blade and elongated anterior iliac process (differs from *Lemmysuchus* (NHMUK PV R 3168) in which the ischial blade is sub-square and the anterior iliac process is shortened); and
8. Two sacral vertebrae (differs from *Lemmysuchus* (NHMUK PV R 3168) which has three sacrals).

Therefore, *S. hulkei* can tentatively be referred to as a juvenile individual of *Neosteneosaurus*.

### Ecomorphological diversity

Our new phylogeny clarifies key ecomorphological aspects of teleosauroids, some of which have briefly been discussed in the literature. The ecological structuring of teleosauroids was initially outlined by Hua (1997) and Hua & Buffetaut (1997) but was never discussed or published in detail. Massare (1987) and recently Foffa et al. (2018) characterized a variety of fossil marine reptiles based on features of the teeth, separating various taxa into dietary guilds. In Foffa et al. (2018), seven teleosauroid taxa were included in the analysis. The results showed that *Machimosaurus* and *Lemmysuchus* occupied the crunch guild, which is specialized for handling hard prey (e.g. turtles); the remaining taxa (*Mycterosuchus*, *Charitomenosuchus*, *Neosteneosaurus* and *Proexochokefalos*) fit into the pierce guild, hypothesized to prefer softer prey such as smaller fishes and squid.

There are a number of ecomorphotypes associated with certain teleosauroid taxa which exhibit a distinct pattern of appearance, and there are four well-sampled points during the Jurassic (Toarcian, Bathonian, Callovian and Kimmeridgian) in which specific patterns of ecomorphotypes emerge (see Table 1; Fig. 63). These ecomorphs can be generally defined based on skull shape (longirostrine, mesorostrine or brevirostrine), dentition (for possible feeding style) and additional osteological characters that relate to the environment (e.g. length of the limbs, placement of the orbits). Teleosauroid skulls are generally split into three different ‘rostral morphs’: longirostrine, mesorostrine and brevirostrine (Fig. 63A), which relate to the length of the rostrum. Longirostry (e.g. *Mycterosuchus*) is defined as the preorbital length being 70% or more of the basicranial length; mesorostry (e.g. *Mystriosaurus*) is the preorbital length being 55–70% of the basicranial length; and brevirostry (e.g. *Mac. mosae*) is the preorbital length being 55% or less than the basicranial length (Andrade et al., 2011). This rostral classification is in turn affiliated with features of the teeth, which include overall size and shape of the teeth, shape of apices, and presence or absence of carinae and ornamentation. In addition to these ‘rostral morphs’, teleosauroid feeding ecology can be broadly categorized into two feeding ‘guilds’: specialist (a species that has a limited diet) or generalist (a species able to thrive on a wide variety of food sources), which can be inferred based on the shape, size and apices of their teeth (Feranec, 2007). Macrophagous/durophagous (feeding on hard prey items) is generally regarded as part of the generalist guild (Foffa et al., 2018), but for the purpose of this paper, we refer to it separately.

**Table 1:** Teleosauroid ecomorphotypes by species in four time periods. List of teleosauroid ecomorphotypes in four main time periods: the Toarcian, Bathonian, Callovian and Kimmeridgian. Note that *S. rostromajor* (Oxfordian), *Indosinosuchus* (Late Jurassic) and *Mac. rex* (Hauterivian–Barremian) are not included.

Taxa	Period	Ecomorph	Characteristic features
*Plagiophthalmosuchus gracilirostris*	Toarcian	Longirostrine, specialist	Lateral orbits; elongated snout; slender pointed teeth
*Macrospondylus bollensis*	Toarcian	Longirostrine, generalist	Dorsal orbits; dorsal nares; ‘generalized’ body plan
*Platysuchus multiscrobiculatus*	Toarcian	Longirostrine, semi-terrestrial	Dorsal orbits; anterior nares; heavy, integrated dorsal shield
*Mystriosaurus laurillardi*, Chinese teleosauroid	Toarcian	Mesorostrine, generalist	Dorsal orbits; anterior nares; shorter rostrum
*Seldsienean megistorhynchus*	Bathonian	Longirostrine, generalist	Dorsal orbits and nares; slender, elongated jaws
*Deslongchampsina larteti*	Bathonian	Mesorostrine, generalist	Dorsal orbits and nares; robust pointed teeth
*Teleosaurus cadomensis*	Bathonian	Longirostrine, semi-terrestrial	Dorsal orbits; ‘spindly’ teeth; heavy, integrated dorsal shield
*Yvridiosuchus boutilieri*	Bathonian	Mesorostrine, durophagous	Enlarged fenestrae; anastomosed blunt teeth
*Andrianavoay baroni, Clovesuurdameredeor stephani*	Bathonian	Unknown	N/A
*Charitomenosuchus leedsi*	Callovian	Longirostrine, generalist	Dorsal orbits and nares; gracile skeleton
*Mycterosuchus nasutus*	Callovian	Longirostrine, semi-terrestrial	Dorsal orbits and nares; protruding orbits; relatively elongated limbs; heavy osteoderms
*Neosteneosaurus edwardsi, Proexochokefalos heberti*	Callovian	Mesorostrine, generalist	Dorsal orbits and nares; robust, elongated skulls; large teeth
*Lemmysuchus obtusidens*	Callovian	Mesorostrine, durophagous	Enlarged fenestrae; anastomosed blunt teeth; reduced postcrania
*Proexochokefalos* cf. *bouchardi*	Kimmeridgian	Mesorostrine, generalist	Dorsal orbits and nares; robust elongated skull
*Machimosaurus buffetauti, Machimosaurus mosae, Machimosaurus hugii*	Kimmeridgian	Mesorostrine, durophagous	Enlarged fenestrae; anastomosed blunt teeth; reduced postcrania
*Sericodon jugleri, Bathysuchus megarhinus, Aeolodon priscus*	Kimmeridgian	Longirostrine, pelagic	Protruding orbits; weakly ornamented skull; reduced osteoderms; forelimb reduced

During the Toarcian, *Plagiophthalmosuchus* represented a longirostrine specialist (Figs. 63A and 63B), characterized by its laterally facing orbits, elongated snout and multiple thin, pointed, poorly ornamented teeth, and was likely purely piscivorous (Westphal, 1962). *Macrospondylus* represents a longirostrine generalist and *Mystriosaurus* is a mesorostrine generalist (a massive, less elongated skull with smaller supratemporal fenestrae and more robust teeth). A heavily armoured, semi-terrestrial longirostrine generalist form is found in *Platysuchus*, indicated by the extensive and tightly packed rows of dorsal osteoderms. It is difficult to discern which ecomorphotype the Chinese teleosauroid (IVPP V 10098) fits into, as no teeth are preserved. However, based on both anatomical and phylogenetic data, this taxon would hypothetically have filled a mesorostrine role, possibly a generalist, similar to *Mystriosaurus* (which is a logical assumption, given *Mystriosaurus* is a closely related taxon).

By the Bathonian, basal teleosauroids with laterally oriented orbits had presumably become extinct (only being known from the Toarcian), with the *Plagiophthalmosuchus* ecomorph vacated (and possibly held by basal metriorhynchoids). However, a new ecomorphotype had evolved: the macrophagous/durophagous mesorostrine form, exhibited by *Yvridiosuchus*. A number of specific features, including enlarged supratemporal fenestrae, an extensive neurovascular system and blunt, conical teeth, characterized this ecomorphotype. The larger supratemporal fenestrae would have housed powerful adductor muscles for closing the jaw, and the robust, rounded teeth were advantageous for capturing a wider or more generalised range of prey (Johnson et al., 2017). There has also been some speculation that the evolution of machimosaurin features may have been linked to the evolution of hard shells in turtles; however, this possible correlation is difficult to test, due to the overall extreme diversification and expansion of coastal marine ecosystems (M. Rabi, 2017, personal communication). In addition to the durophagous/macrophagous role, *Seldsienean* filled the longirostrine generalist niche; *Deslongchampsina* filled the niche of mesorostrine generalist; and *Teleosaurus* replaced *Platysuchus* as the longirostrine, semi-terrestrial generalist form. The possible ecomorphotypes for both *Andrianavoay* and *Clovesuurdameredeor* are currently uncertain; morphologically it is clear that they do not represent machimosaurins (e.g. lack two rows of maxillary neurovascular foramina in *Andrianavoay*; no enlarged supratemporal fenestrae in *Clovesuurdameredeor*). Most of the rostral material is missing from *Clovesuurdameredeor*, making it difficult to infer skull and dental morphology. The preserved rostral section (including the anterior and middle maxillae) of *Andrianavoay* has at least 20 maxillary alveoli preserved; due to its position on the phylogeny, it may possibly have been a mesorostrine generalist, similar to *Neosteneosaurus*.

In the mid-Callovian, the ecomorphotypes within this ecological hierarchy did not change. *Lemmysuchus* represented a mesorostrine macrophagous/durophagous form; *Charitomenosuchus* became the longirostrine generalist; *Neosteneosaurus* and *Pr. heberti* both filled the role of mesorostrine generalist; and *Mycterosuchus* represented the longirostrine, semi-terrestrial ecomorphotype. However, in the Kimmeridgian, there was another major shift in ecomorphotype variation. The macrophagous/durophagous form became the most dominant ecomorph, with representatives in *Mac. buffetauti*, *Mac. mosae* (both brevirostrine) and *Mac. hugii* (mesorostrine). The semi-aquatic longirostrine generalist ecomorph disappeared, and the mesorostrine generalist, represented by *Pr*. cf. *bouchardi*, became extremely rare. In addition, another new ecomorphotype evolved: a longirostrine, semi-pelagic generalist form, represented by a handful of genera (*Aeolodon*, *Bathysuchus* and *Sericodon*). During the Upper Jurassic (the exact time is unknown), *Indosinosuchus* represented a probable generalist, mesorostrine form; and in the Hauterivian-Barremian (132–121 Ma), *Mac. rex* embodied the macrophagous/durophagous ecomorph, but all other teleosauroids had presumably disappeared.

These six different ecomorphotypes are scattered across the phylogeny. *Plagiophthalmosuchus*, the basal-most teleosauroid, is the only taxon that is a definitive longirostrine specialist (Fig. 63). Mesorostrine generalists are represented by both teleosaurids and machimosaurids: the Chinese teleosauroid (IVPP V 10098), *Mystriosaurus* and *Indosinosuchus* (Teleosauridae); and *Deslongchampsina*, *Proexochokefalos*, and *Neosteneosaurus* (Machimosauridae) (Fig. 63). Interestingly, the remaining three ecomorphotypes are restricted to certain families. The longirostrine semi-terrestrial form is only found in Teleosauridae, represented by *Platysuchus*, *Teleosaurus* and *Mycterosuchus*. The longirostrine, generalist pelagic ecomorphotype is also restricted to Teleosauridae, as seen in *Aeolodon*, *Sericodon* and *Bathysuchus* (Figs. 63A–63C). The longirostrine generalist (*Macrospondylus*, *Seldsienean*, *Charitomenosuchus*) and mesorostrine/brevirostrine macrophagous/durophagous (*Yvridiosuchus*, *Lemmysuchus*, *Machimosaurus*) ecomorphologies are only found in Machimosauridae (Fig. 63).

As seen in extant crocodylian species, larger individuals tend to be dominant, with larger species occupying prime territories, although this is not an unbreakable rule, as interactions between *Crocodylus rhombifer* (Cuban Crocodile) and *Crocodylus acutus* (American Crocodile) in the Central Americas demonstrate (Targarona et al., 2010; Thorbjarnarson, 2010). It is hypothetical that machimosaurids, being larger and more generalist, were able to assert dominance over smaller teleosaurids if co-existing within the same ecosystem, and therefore occupied more prime territories. This could have acted as a selection pressure and driven the evolution of more specialised ecomorphotypes. This is similar to that seen in extant crocodylian subdivisions of West African ecosystems; the species *Crocodylus suchus* (West African Crocodile), *Mecistops cataphractus* (West African slender-snouted crocodile) and *Osteolaemus tetraspis* (African Dwarf Crocodile) do not inhabit similar bodies of water (Kofron, 1992; Velo-Antón et al., 2014), and with decreasing size, all species live in smaller waterways, with *Osteolaemus* being capable of terrestrial foraging. This could be similar to the hierarchy seen in South American caimans: *Melanosuchus niger* (Black Caiman), *Paleosuchus palpebrosus* (Cuvier’s Dwarf Caiman), *Caiman yacare* (Yacare Caiman), *Caiman crocodilus* (Spectacled Caiman) and *Caiman latirostris* (Broad-Snouted Caiman) (Ross, 1998; Busack & Pandya, 2001; Rebêlo & Lugli, 2001; Vasconcelos et al., 2008).

An additional interesting factor is that, throughout time, there were never more than four ecomorphological ‘guilds’ within teleosauroids (Fig. 64). Mesorostrine generalists (e.g. *Deslongchampsina*) and longirostrine generalists (e.g. *Charitomenosuchus*) were consistently present until the Late Jurassic, whereas the basal longirostrine specialist (*Plagiophthalmosuchus*) was present only during the Early Jurassic. During the Kimmeridgian/Tithonian, there were only three ecomorphs present (Fig. 64) (macrophagous/durophagous, longirostrine pelagic, and mesorostrine generalist forms) with two of these (macrophagous/durophagous and longirostrine pelagic forms) being dominant while the third (mesorostrine generalist form) was much rarer. In addition, Young et al. (2014) noted that, during the Late Jurassic, there was a divide within the genus *Machimosaurus* between ‘open-sea’ *Machimosaurus* body-plans (i.e. *Mac. hugii*, as suggested by the enlarged paraoccipital processes for muscle attachment) and nearshore/turbulent water body-plans (i.e. *Mac. mosae*). The overall reflection of teleosauroid nice partitioning highlights three main points:

1. There was a specific niche partitioning strategy among teleosauroids that lived during similar times;
2. The ecomorphological diversity of teleosauroids was generally stable through time until the Late Jurassic; and
3. After the Late Jurassic, there was a growing divide within Teleosauroidea between near-shore forms and increasingly open-sea species.

### Biogeographical distribution

Throughout their approximately 70-million-year history, teleosauroids achieved near-global distribution. Numerous specimens have been found across both Gondwanan and Laurasian continents, having been reported from the UK and Europe (Eudes-Deslongchamps, 1867; Westphal, 1961, 1962; Andrews, 1909, 1913; Benton & Taylor, 1984; Young et al., 2014; Johnson et al., 2017; Čerňanský et al., 2017; Foffa et al., 2019), Africa (Newton, 1893; De Lapparent, 1955; Buffetaut, Termier & Termier, 1981; Bardet & Hua, 1996; Fara et al., 2002; Fanti et al., 2016; Jouve et al., 2016; Dridi & Johnson, 2019), Asia (Young, 1948; Liu, 1961; Li, 1993; Martin et al., 2019), India (Owen, 1852; Phansalkar, Sudha & Khadkikar, 1994), Siberia (Efimov, 1982, 1988; Storrs & Efimov, 2000), South America (Cortes et al., 2019) and potentially North America (Table 2). Von Huene (1927) described two dorsal vertebrae from the Upper Lias of Portezuelo Ancho in north-western Argentina and attributed them to *Steneosaurus gerthi* (Buffetaut, Termier & Termier, 1981; Gasparini & Fernández, 2005); however, these specimens are now referred to as Thalattosuchia indeterminate (Gasparini & Fernández, 2005).

**Table 2:** Teleosauroid localities and material by genera. Comprehensive list of localities where teleosauroid material has been found; grouped by genera and includes ‘*Steneosaurus*’, ‘*Teleosaurus*’ and *Machimosaurus* spp.

Genera	Country	Locality	Material found
*Plagiophthalmosuchus*	Luxembourg; UK	Dudelange; Whitby	Partial skeleton; skull
*Platysuchus*	Germany; Luxembourg	Holzmaden; Foetz	Complete skeleton; rostrum
*Mystriosaurus*	Germany; UK	Altdorf; Whitby	Complete and partial skulls
Chinese teleosauroid	China	Daxian	Complete skull
*Macrospondylus*	Germany; ?France; Luxembourg; UK	Holzmaden; Bad Boll; Ohmden; Altdorf; Banz; Berg; Schlierbach; Ohmenhausen; ?Yonne; Sanem; Whitby; Sandsend; Greens-Norton	Multiple complete and partial specimens, as well as cranial and postcranial material
*Deslongchampsina*	France; UK	Calvados; Enslow Bridge	Near complete skulls
*Clovesuurdameredeor*	UK	Closworth	Partial skull and mandible
*Yvridiosuchus*	France; UK	Calvados; Enslow Bridge	Complete and partial skulls and mandibles
*Teleosaurus*	?China; France	?Sichuan province; Allemagne; Calvados	Partial cranium; osteoderms; postcranial material
*Andrianavoay*	Madagascar	Unknown	Partial skull and mandible, osteoderm fragment
*Seldsienean*	UK	Enslow Bridge; Kirtlington	Partial mandibles
*Lemmysuchus*	France; UK	Unknown; Peterborough	Complete skull and partial mandible; near complete skeleton and additional skull material
*Charitomenosuchus*	UK	Peterborough	Near compete skeleton as well as additional skull and postcranial material
*Mycterosuchus*	Germany; UK	Unknown; Peterborough	Complete skulls and postcranial material
*Neosteneosaurus*	UK	Peterborough	Near compete skeleton as well as additional skull and postcranial material
*Proexochokefalos*	France; Switzerland	Villers-sur-Mer; Villerville; Courtedoux-sur Combe Ronde	Complete and partial skulls; few postcranial material
*Machimosaurus*	Ethiopia; France; Germany; Portugal; Spain; Switzerland; Tunisia	Feyambiro; Ain; Ambleteuse; Cricqueboeuf; Issoncourt; Neuffen; Leiria; Lagares; Lourinhã; Malhão-Algarve; Peralta; Porto das Barcas; Zimbral; Asturias; Buñol; Solothurn; Touil el Mhahir	Skulls, mandibles, postcrania; numerous isolated teeth
*Indosinosuchus*	Thailand	Pho Noi	Multiple skulls and partial postcranial material
*Sericodon*	Germany; Switzerland	Hannover; Ahlem; Tönniesberg; Courtedoux-Bois de Sylleux; Courtedoux-sur Combe Ronde; Courtedoux-Tchâfouè; Courtedoux-Vâ Tche Tchâ	Numerous teeth; partial skull and postcranial material
*Aeolodon*	France; Germany	Canjuers; Daiting	Near complete skeletons
*Bathysuchus*	France; UK	Quercy; Kimmeridge	Nearly complete skull and partial mandible; rostral material
‘*Steneosaurus*’ sp.	Belgium; France; Germany; India; Poland; Russia; UK	Lorraine; Poitiers; Vaches Noires; Bartenbach; Bhuj; Czarnogłowy; Dagestan; Kirtlington; Whittlesea	Partial rostra and skulls; postcranial material; teeth
‘*Teleosaurus*’ sp.	China; India; UK	Beipei; Kota; Kirtlington; Slape Hill Quarry	Postcranial material; osteoderms
*Machimosaurus* sp.	France; Portugal; Spain; Switzerland; UK	Haudainville; Porto das Barcas; Peralta; Zimbral; Buñol; Moutier; Oker quarry; Solothurn; Lyme Regis; Dorset	Teeth

Despite this vast global dispersal, few studies have examined teleosauroid biogeography in detail. Buffetaut, Termier & Termier (1981) suggested a Laurasian and Gondwanan faunal connection between Tethyan Europe and the southern area of Africa (such as Madagascar) via an epicontinental seaway during the Early Jurassic. In the late Toarcian, the distribution of teleosauroids appear parallel to the ammonite *Bouleiceras*, which occurs in Portugal (Mouterde, 1953), Spain (Geyer, 1965), Chile, Argentina (Von Hillebrandt, 1973), Madagascar, Algeria and Morocco (Buffetaut, Termier & Termier, 1981), suggesting a marine connection from South America around Africa to the Tethyan area. In addition, Hua & Buffetaut (1997) hypothesized that teleosauroid distribution was similar to that of the Saltwater Crocodile (*Crocodylus porosus*) living amongst the Indian Ocean archipelagos.

Fossil localities appear to reflect the biogeographical diversity of teleosauroids. During the upper Toarcian, teleosauroids were already biogeographically distinct. Representatives from both Teleosauridae and Machimosauridae, as well as the basal teleosauroid *Plagiophthalmosuchus*, are found in the Whitby Mudstone Formation in Britain (*Mystriosaurus*, *Macrospondylus*), the ‘*schistes bitumineux*’ in Luxembourg (*Macrospondylus*, *Platysuchus*), an unknown locality in France (*Macrospondylus*) and the Posidonia Shale Formation in Germany (*Platysuchus*, *Macrospondylus*, *Mystriosaurus*). In Asia, the Chinese teleosauroid and indeterminate ‘*Teleosaurus*’ material are noted from the Ziliujing Formation of Beipei, Sichuan in China (Li, 1993; Li et al., 2011). In addition, Toarcian *Steneosaurus* specimens have been reported from Belgium (‘*oolithe ferrugineuse*’), India (Kota Formation), Madagascar (Kandreho Formation), and possibly Portugal (Owen, 1852; Buffetaut, Termier & Termier, 1981; Godefroit, 1994). These multiple occurrences in different localities indicate that during the beginning of teleosauroid evolution, they were already radiating across the world, possibly following the coastline.

During the Aalenian and Bajocian (180.1–169.2 Ma), there are few teleosauroid occurrences, but there are two geographically important ‘*Steneosaurus*’ sp. found in Slovakia (Pieniny Klippen Belt unit; Aalenian) and Dagestan Republic (Karakh Formation; Aalenian). During the Middle Jurassic (Late Aalenian to Early Bajocian), Buffetaut (1979) reported teleosauroid material from Oregon (USA); this material has since been attributed to a member of Metriorhynchoidea (Wilberg, 2015b). However, some non-documented, additional fragments from the same timeframe and locality are still labelled as Teleosauridae (NMNH PAL 357211–357215). In the Bathonian (169.2–164.4 Ma), several teleosauroid genera have been reported from localities in France (*Yvridiosuchus*, *Teleosaurus*, *Seldsienean*, *Deslongchampsina*, ‘*Steneosaurus*’; Eudes-Deslongchamps, 1867; Johnson, Young & Brusatte, 2019), Britain (*Clovesuurdameredeor*, *Yvridiosuchus*, *Teleosaurus*, *Seldsienean*, *Deslongchampsina*; Eudes-Deslongchamps, 1867; Johnson, Young & Brusatte, 2019), Madagascar (*Andrianavoay*; Newton, 1893) and Morocco (Machimosaurini indeterminate).

There is a multitude of occurrences in the Callovian (164.4–159.4 Ma), particularly in Britain (Oxford Clay Formation): taxa found in this area include *Mycterosuchus*, *Charitomenosuchus*, *Neosteneosaurus* and *Lemmysuchus*. Teleosauroids such as *Proexochokefalos* (Marnes de Dives Formation), *Lemmysuchus* (Quercy) and ‘*Steneosaurus*’ sp. (unknown formation) are found in France, as well as ‘*Steneosaurus*’ sp. (Chari Formation) in India. As with the Aalenian-Bajocian, few teleosauroids have been reported from the Oxfordian (159.4–154.1 Ma). However, there are a couple of specimens described from unique localities, such as:

1. *Machimosaurus nowackianus* from Harrar, Ethiopia (Von Huene, 1938; Bardet & Hua, 1996; Young et al., 2014);
2. *Machimosaurus* sp. (*Perisphinctes cautisnigrae* ammonite zone) and *L*. cf. *obtusidens* (Corallian Group; Foffa, Young & Brusatte, 2015) from Britain; and
3. *Steneosaurus rostromajor* (possibly Marnes de Villiers Formation; Cuvier, 1812, 1824; Geoffroy Saint-Hilaire, 1825) from France.

In the Kimmeridgian (154.1–150.7 Ma), teleosauroids are found in several localities: *Bathysuchus* from the Kimmeridge Clay Formation (UK); *Mac. hugii*, *Sericodon* and *Pr*. cf. *bouchardi* from the Reuchenette Formation (Switzerland); *Mac. buffetauti* from the Lacunosamergel Formation (Germany); *Mac. hugii* from the Alcobaça and Lourinhã Formaions (Portugal), as well as the Lastres and Tereñes Formations (Spain) and Calcaires Coquilliers Formation (*P. baylei* Sub-Boreal ammonite Zone; Cricqueboeuf, France); and *Pr*. cf. *bouchardi* from the ‘*Calcaire de Caen*’ (France) (Lepage et al., 2008; Young et al., 2014; Schaefer, Püntener & Billon-Bruyat, 2018; Foffa et al., 2019). In addition, *Machimosaurus* sp. is found in Germany (Langenberg Formation), the UK (Kimmeridge Clay Formation), Switzerland (Reuchenette and unknown Formations) and Portugal (Lourinhã Formation) (Young & Steel, 2014; Young et al., 2014), and ‘*Steneosaurus*’ sp. has been found from the Czarnogłowy quarry in Poland (Čerňanský et al., 2017). Tithonian localities are restricted to the Higueruelas Formation in Spain (*Mac. hugii*), the Mörnsheim Formation in Germany (*Aeolodon*) and the Canjuers lagerstätte and ‘Marnes supérieures de la Meuse’ in France (*Aeolodon* and *Mac. mosae*, respectively). *Indosinosuchus* comes from the Late Jurassic Phu Kradung Formation of Phu Noi (north-eastern Thailand); dating this stratigraphic section is particularly tricky, as vertebrate fossils indicate a Late Jurassic age but palynomorphs suggest Early Cretaceous (Martin et al., 2019). A Late Jurassic, possibly Tithonian, age has been proposed (Liard & Martin, 2011; Cuny et al., 2014; Deesri et al., 2014), but this is currently unconfirmed.

Two geographically important specimens have been attributed to the genus ‘*Steneosaurus*’: a partial skull from the Karakh Formation (Aalenian) of Dagestan, Russia (Efimov, 1988), and two skulls from the Chari Formation (Callovian) near Gujarat, India (Phansalkar, Sudha & Khadkikar, 1994). The Dagestan skull (Efimov, 1988) was housed at the Grozny Petroleum Research Institute (GrozNII) in the Chechen Republic but was destroyed due to military conflict in the area (S. Zaurbekov, 2019, personal communication). This is unfortunate, not only in the loss of three valuable specimens, but also in the fact that their unique locations would provide invaluable information on which teleosaurids and/or machimosaurids spread into these areas. Efimov (1988) described the Dagestan skull as ‘*Вместе с тем в конфигурации краниальной пластины она обнаруживает сходство с верхнеюрскими видами стенеозавра, в частности с*S. larteti *и S. edwardsi*’ (‘At the same time, in the configuration of the cranial plate, it reveals similarities with the Upper Jurassic species (of) *Steneosaurus*, in particular, *S*. *larteti* and *S*. *edwardsi*’) (Efimov, 1988: 52). However, there are no photographs of the specimen, so this is difficult to confirm. Currently, the Gujarat skulls cannot be located; in addition, Phansalkar, Sudha & Khadkikar (1994) did not describe either of the Gujarat specimens, only noting their occurrence within the Chari Formation. There is one photograph of one skull, as well as two drawings, but they are poor, and no anatomical information can be gleaned from them. Khadkikar (1996) briefly noted the skulls, suggesting that they could belong to *S. durobrivensis* (= *S. edwardsi* = *Neosteneosaurus*). Nevertheless, these specimens exhibit the remarkable distributional success and adaptability that teleosauroids were able to achieve.

Based on the biogeography of the above fossil sites, it appears that teleosauroids primarily diversified and dispersed around the Tethys Sea (which was a productive area, consisting of many continental reef ecosystems: Stanley, 1988), and most species were concentrated around the Jurassic tropic belts. This is also consistent with climate data (Rees, Ziegler & Valdes, 2000; Jenkyns et al., 2012; Korte et al., 2015), which suggests rapid warm/cool events influenced by oceanic currents followed by warm conditions (26–30 °C) during the Middle Jurassic, as well as overall minimal global climate change throughout the Jurassic, making the coastlines exceptionally productive. However, there are still three main problems which continue to limit our understanding of teleosauroid dispersal and distribution through time. Firstly, there is a substantial area where material is either missing or severely fragmentary, including the Tethys coast of Africa and the eastern coast of Africa (ranging from Ethiopia to Madagascar). Secondly, the lack of confident identification for the lost Chechen material (Aalenian), and the Indian (Toarcian and Callovian) and Chinese (Toarcian) specimens limits our knowledge of which species of teleosauroids were able to successfully disperse into these areas. Lastly, the South American record for teleosauroids is surprisingly non-existent, as they are known only from the Early Cretaceous (Cortes et al., 2019). As teleosauroids must have dispersed through multiple routes along the Jurassic coastlines, it would be logical that they were able to migrate into the South American area during this time. It is therefore essential that future research examines material from, as well as exploring more of, these areas. As with patterns in teleosauroid ecomorphology, genera within both families were established in different locations (see Table 2). Teleosauridae were restricted to Laurasian continents, with *Teleosaurus*, *Aeolodon*, *Mystriosaurus* and *Bathysuchus* known from the UK and Europe; *Mycterosuchus* from Britain and Germany; *Platysuchus* from Europe (Germany and Luxembourg); and *Indosinosuchus* and the Chinese teleosauroid (and possibly *Teleosaurus*) from Asia. Machimosauridae have an overall wider geographical span, ranging from the UK and Europe to northern Africa, Madagascar and possibly India, with machimosaurins in particular being prevalent in Africa. The phylogeny also shows that teleosauroids were able to distribute across the continent early in their evolution; *Plagiopthalmosuchus*, three teleosaurids (*Mystriosaurus*, *Platysuchus*, the Chinese teleosauroid) and one machimosaurid (*Macrospondylus*) were definitively present during the early Toarcian in five distinct localities.

### Palaeoenvironment and the importance of freshwater teleosauroids

The majority of teleosauroid species are found in semi-aquatic or marginal marine (generally coastal and lagoonal) environments, and certain taxa are hypothesized to have lived in semi-pelagic (*Aeolodon*, *Bathysuchus* and *Sericodon*), semi-terrestrial (*Mycterosuchus*, *Teleosaurus* and *Platysuchus*) and open ocean (*Mac. hugii*) ecosystems (refer to Fig. 63C). However, three purely East Asian teleosauroids, the Chinese teleosauroid (IVPP V 10098) and two species of *Indosinosuchus*, are found in freshwater deposits (Li, 1993; Martin et al., 2016, 2019). This is intriguing, as no other teleosauroids are known from these types of deposits. In environmental terms, this is striking with reference to two points: (1) adult vs juvenile habitat preference; and (2) specific osteological features.

Some modern crocodylians, such as *Cr. porosus* (Saltwater Crocodile), often prefer different habitats depending on their age (juvenile/sub-adult vs. adult) (Read et al., 2004), which is often related to body size and food preference (Taylor, 1979; Magnusson, Da Silva & Lima, 1987). In general, adults are more common in estuary or brackish regions, whereas juveniles and sub-adults prefer freshwater ecosystems such as rivers or lakes. It is possible that teleosauroids adopted a similar pattern, with mature individuals frequenting semi-marine habitats, and hatchlings and juveniles in freshwater environments. However, small specimens of *Macrospondylus* (less than 1 m total length) have been found in the Posidonia Shale Formation from Holzmaden (e.g. SMNS 10,000), which consists of marginal marine sedimentological deposits. In addition, adult individuals of *Cr. porosus* (Webb, Manolis & Brien, 2010), *Crocodylus acutus* (American Crocodile) (Thorbjarnarson et al., 2006) and possibly *Crocodylus siamensis* (Siamese Crocodile) (Smith, 1931; Platt et al., 2006) have been known to thrive in both saltwater and freshwater ecosystems.

Certain osteological characteristics in mature individuals can also be indicative of preferential habitat. The Indian gharial (*Gavialis gangeticus*), which is confined to riverine ecosystems, has distinctive protruding eyes (= telescoped orbits) that aid in capturing fish (Whitaker & Basu, 1983). In gavialoids, these telescoped orbits are homoplastic and independently evolved twice, once in advanced *Gryposuchus* species (*Gr. colombianus* and *Gr. croizati*) from South America, and once in Asian *Gavialus* (Salas-Gismondi et al., 2016). The depositional settings in which these taxa are found are fluvial-dominated paleoenvironments, which suggests that well-developed telescoped orbits are correlated with riverine ecosystems (Salas-Gismondi et al., 2016). In teleosauroids, *Indosinosuchus potamosiamensis* displays distinctive telescopic orbits (although not as widely separated as *Gavialis*) and is found in freshwater deposits (Martin et al., 2019), similar to *Gryposuchus* species. It would therefore be logical to assume that *Indosinosuchus kalasinensis*, from the same deposits, would also have had telescoped orbits; however, the skull (PRC-239) is slightly dorsoventrally crushed, making this confirmation difficult. Interestingly, *Mycterosuchus nasutus*, and more subtly *Teleosaurus cadomensis*, have telescoped orbits; it is thus hypothesized that these two taxa may have also preferred riverine/fluvial areas rather than marginal marine ecosystems.

In other fossil crocodylomorphs, the dyrosaurid *Acherontisuchus guajiraensis* Hastings, Bloch & Jaramillo, 2011 is hypothesized to have inhabited calmer, fluvial waters than other Old World dyrosaurids. The slender and narrow ischial shaft of this taxon had reduced surface area for attachment surfaces of the *m. rectus abdominis* and *m. ischiopubis*, which are responsible for respiration and pitch control in water (Hastings, Bloch & Jaramillo, 2011). The ischial shaft in teleosauroids is not as narrow or elongated as in dyrosaurids; the ischial shaft of the supposed fluvial *I. potamosiamensis* (PRC-27: Martin et al., 2019) does not look particularly different from the majority of teleosauroids (e.g. *Charitomenosuchus*, *Neosteneosaurus*), excluding machimosaurins (e.g. *Lemmysuchus*). In addition, the sedimentology (Cerrejón Formation, Colombia) along with associated flora and fauna, suggest that *A. guajiraensis* lived in a freshwater habitat. All specimens of *A. guajiraensis* are mature individuals, with specimens ranging from 4.6 to 6.4 m in length (Hastings, Bloch & Jaramillo, 2011). Adult specimens of the pholidosaurids *Sarcosuchus*, *Elosuchus* and *Meridiosaurus* are also thought to have inhabited freshwater ecosystems (Fortier, Perea & Schultz, 2011). Therefore, it is possible that mature teleosauroids did indeed frequent freshwater ecosystems, but solely in eastern Laurasian regions. More discoveries are needed from freshwater deposits in Europe to test whether many marginal marine teleosauroids were solely marine taxa.

One additional salient feature of teleosauroids is the position of the external nares. They are described as being either anterodorsally (e.g. in *Indosinosuchus*) or dorsally (e.g. in *Deslongchampsina*) oriented. However, in *Mystriosaurus*, the external nares are directed anteriorly (Sachs et al., 2019a). This is intriguing, as this positioning would not be practical for a semi-aquatic lifestyle. It is hypothetical that, due to this unusual placement of the external nares, *Mystriosaurus* was more terrestrial, or spent a greater amount of time on land, than other teleosauroids. Indeed, this example shows just how possible it is that some teleosauroids were, in actuality, not particularly well suited for living in water.

### Teleosaurids vs. machimosaurids

In terms of morphology and ecology, teleosaurids are more phenotypically plastic than machimosaurids (see Fig. 63). They display three distinct ecomorphs (mesorostrine generalist, longirostrine pelagic specialist and longirostrine semi-terrestrial generalist) and potentially occupied four environmental habitats (semi-marine, pelagic, freshwater and semi-terrestrial). In contrast, machimosaurids seem to display an almost linear pattern: basal machimosaurids (e.g. *Macrospondylus*) are longirostrine, semi-marine generalists; more derived machimosaurines (e.g. *Deslongchampsina*, *Proexochokefalos*) are mesorostrine, semi-marine generalists, with more robust teeth; and machimosaurins (e.g. *Lemmysuchus*, *Machimosaurus*) are large-bodied, durophagous, semi-marine taxa, with complex dentition and robust skeletons. In terms of abundance and geographical dispersal, teleosaurids appear to be less common than machimosaurids, and based on current knowledge, were restricted to Laurasia. Machimosaurids as a whole, particularly *Macrospondylus*, have high abundance, and decrease in numbers after the Callovian. During the Kimmeridgian, *Machimosaurus* was the most common teleosauroid genus, but was less abundant than other contemporaneous marine reptiles. The distribution of machimosaurids is generally in Sub-Boreal European and Gondwanan areas and their dispersal was expansive, with multiple occurrences found in the UK, Europe and Africa, and potentially India. However, there is a possible instance of them being found in Siberia (see above). It is possible that machimosaurids had larger ranges than contemporaneous teleosaurids, with teleosaurids being more specialized and therefore restricted to certain environments. These ideas, reinforced by the phylogeny, show that teleosauroids were without doubt much more diverse, in terms of morphology, ecology and geography, than previously thought.

An additional factor that differs between teleosaurids and machimosaurids is body size. Machimosaurids reached over 5 m in total length during the lower Toarcian (e.g. *Macrospondylus*; Westphal, 1961); they continued to get bigger in the Middle and Late Jurassic, and into the Cretaceous (with *Mac. rex* hypothesized to be around 7.15 m in total length; Young et al., 2016). Teleosaurids remained smaller in every ecosystem in which they co-existed with machimosaurids; only the taxa *Mystriosaurus* and *Mycterosuchus* came close to the body sizes of machimosaurids. It is possible that this difference in body size is related to territory, locomotor and thermoregulation performance, and food sources, as in modern crocodylians (Grigg et al., 1998; Elsworth, Seebacher & Franklin, 2003).

## Conclusions

Despite an increase in morphological work within the past decade, the evolutionary relationships of teleosauroids are poorly understood and little studied, and thus their macroevolutionary patterns are rarely evaluated. One major issue is the genus *Steneosaurus*, which is often recovered as paraphyletic or polyphyletic in phylogenetic analyses. Following on our recent re-classification of *Steneosaurus* as a nomen dubium and an invalid genus (Johnson, Young & Brusatte, 2020), we herein presented an in-depth phylogenetic evaluation of Teleosauroidea. We firstly proposed the following changes to teleosauroid nomenclature, as a direct result of the invalidity of *Steneosaurus*: seven new generic names (*Plagiophthalmosuchus*, *Clovesuurdameredeor*, *Seldsienean*, *Charitomenosuchus*, *Proexochokefalos*, *Andrianavoay* and *Neosteneosaurus*) and one new species (*Indosinosuchus kalasinensis*); and the resurrection of three historical genera (*Macrospondylus*, *Aeolodon* and *Sericodon*). Secondly, we described 38 new characters and 19 additional characters that are important and distinctive in teleosauroid morphology and discussed how these characters differ between taxa. Thirdly, we listed the results of the phylogenetic analyses based on our updated H+Y data matrix, containing 153 taxa (including 27 teleosauroids) and 502 osteological characters. Our results showed that both parsimony and Bayesian topologies are relatively consistent with one another. Next, we propose and define the following taxonomic clades: the families Teleosauridae (re-defined) and Machimosauridae, and the subfamilies Aeolodontinae and Machimosaurinae (which includes Machimosaurini). Finally, we evaluated the ecomorphology and distribution of teleosauroids, based on our new phylogeny. Teleosauridae and Machimosauridae are morphologically distinct, with differing biogeographic distributions (Teleosauridae is Laurasian and Machimosauridae is Sub-Boreal European-Gondwanan), habitat preferences and feeding strategies. The phylogeny infers that the teleosaurids were overall more phenotypically plastic than machimosaurids, with an east-Asian freshwater clade, a nascent pelagic clade, and a heavily armoured clade; machimosaurids were dominant in terms of abundance and dispersal, with a linear pattern of morphological changes. By evaluating our updated phylogeny, it is clear that teleosauroids were, in terms of morphology, ecology and geography, more diverse than previously thought.

## Supplemental Information

10.7717/peerj.9808/supp-1 Supplemental Information 1 CrocSuperMatrix Project Overview, datasets, and references.

10.7717/peerj.9808/supp-2 Supplemental Information 2 Matrix.

10.7717/peerj.9808/supp-3 Supplemental Information 3 EIW raw data code.

10.7717/peerj.9808/supp-4 Supplemental Information 4 Original raw data code.

10.7717/peerj.9808/supp-5 Supplemental Information 5 Detailed character list.

10.7717/peerj.9808/supp-6 Supplemental Information 6 Teleosauroid specimen list.

10.7717/peerj.9808/supp-7 Supplemental Information 7 Extended Implied Weighting Entire Phylogenetic Tree.

## Abbreviations

BHN2: Muséum d’Histoire Naturelle de Boulogne-sur-Mer, France (closed in 2003)
BIRUG: Lapworth Museum of Geology, Birmingham, UK
BRLSI: Bath Royal Literary and Scientific Institution, Bath, UK
BSY: Catalogue du patrimoine paléontologique jurassien—A16, Porrentruy, Switzerland
CAMSM: Sedgwick Museum of Earth Science, Cambridge, UK
DFMMh: Dinosaurier-Freilichtmuseum Münchehagen, Lower Saxony, Germany
DONMG: Doncaster Museum, Doncaster, UK
DORCM: Dorset County Museum, Dorchester, UK
FMNH: Field Museum of Natural History, Chicago, USA
GPIT: Paläontologische Sammlung der Eberhard Karls Universität, Tübingen, Germany
GrozNII: Grozny Petroleum Research Institute, Chechen Republic, Russia
GZG: Geologisches institut Geologisch-Paläontologisches, Göttingen, Germany
HLMD: Hessisches Landesmuseum, Darmstadt, Germany
IRSNB: Institut Royal des Sciences Naturelles de Bruxelles, Brussels, Belgium
IVPP: Institute of Paleontology and Paleoanthropology, Beijing, China
LMH: Landesmuseum, Hannover, Germany
LPP: Institut de paléoprimatologie, paléontologie, humaine évolution et paléoenvironnements Université de Poitiers, Poitiers, France
MCNV: Museo de Ciencias Naturales de Valencia, Spain
MG: Museu Geológico, Lisbon, Portugal
ML: Museu da Lourinhã, Lourinhã, Portugal
MMG: Staaliches Museum für Mineralogie und Geologie, Dresden, Germany
MMT: Musée d’Art et d’Histoire Michel Hachet, Toul, France
MNHN: Muséum National d’Histoire Naturelle, Paris, France
MNHNL: Musée National d’Histoire naturelle, Luxembourg City, Luxembourg
MPV: Musée paléontologique (Paléospace) de Villers-sur-Mer, Normandy, France
MUHNAC: Museu Nacional de História Natural e da Ciência Lisbon, Lisbon, Portugal
NHMUK: Natural History Museum, London, UK
NAMU: Naturkunde-Museum Bielefeld, Bielefeld, Germany
NHMW: Naturhistorisches Museum Wien, Vienna, Austria
NM: Národní museum, Prague, Czech Republic
NMS: Naturmuseum Solothurn, Switzerland
NMNSJ: National Museum of Nature and Science, Tokyo, Japan
NOTNH: Nottingham Natural History Museum, Nottingham, UK
NZM-PZ: Naturhistoriska Riksmuseet Palaeozoological, Stockholm, Sweden
ONM: Office National des Mines, Tunis, Tunisia
OUMNH: Oxford University Museum of Natural History, Oxford, UK
PETMG: Peterborough Museum and Art Gallery, Peterborough, UK
PIN: Paleontological Institute, Moscow, Russia
PMU: Evolutionsmuseet Uppsala Universitet, Uppsala, Sweden
PRC: Palaeontological Research and Education Centre, Maha Sarakham University, Thailand
SCR: Catalogue du patrimoine paléontologique jurassien—A16, Porrentruy, Switzerland
SMF: Naturmuseum Senckenberg Frankfurt, Germany
SMHM: Staaliches Naturhistorisches Museum, Braunschweig, Germany
SMNS: Staatliches Museum für Naturkunde Stuttgart, BadenWürttemberg, Germany
TCH: Catalogue du patrimoine paléontologique jurassien—A16, Porrentruy, Switzerland
UH: Urweltmuseum Hauff Holzmaden, Germany
VTT: Catalogue du patrimoine paléontologique jurassien—A16, Porrentruy, Switzerland
YORYM: Yorkshire Museum, York, UK

## Anatomical

ac: Acetabulum
?an: Possible angular
an: Angular
anas: Anastomosing pattern (tooth)
ant il pr: Anterior iliac process
antorb f: Antorbital fenestra
art: Articular
?atl-ax: Possible atlas-axis complex
atl: Atlas
ax: Axis
basiocc: Basioccipital
?basisph: Possible basisphenoid
basisph: Basisphenoid
cerv r: Cervical rib
cerv v: Cervical vertebra
cn XII: Cranial nerve XII
cor: Coracoid
cor f: Coracoid foramen
cor gr: Coronoid groove
D3: Third dentary alveolus
D4: Fourth dentary alveolus
D16: Sixteenth denary alveolus
D17: Seventeenth dentary alveolus
den: Dentary
dors os: Dorsal osteoderm
dors v: Dorsal vertebra
ectopt: Ectopterygoid
ex n: External nares
f: Frontal
f m: Foramen magnum
fem: Femur
fem h: Femoral head
gl f: Glenoid fossa
hum: Humerus
hum h: humeral head
il: Ilium
isch: Ischium
isch bl: Ischial blade
j: Jugal
?l: Possible lacrimal
l: Lacrimal (lachrymal)
k: Keel (osteoderm)
li: Limb bone (unknown)
M10: Tenth maxillary alveolus
M12: Twelfth maxillary alveolus
mand f: Mandibular fenestra
mand sy: Mandibular symphysis
meck c: Meckelian canal (=groove)
mx: Maxilla
mx al: Maxillary alveolus
n: Nasal
occ con: Occipital condyle
od: Odontoid
orb: Orbit
os: Osteoderm fragment
P1: First premaxillary alveolus
P2: Second premaxillary alveolus
P3: Third premaxillary alveolus
?p: Possible parietal
p: Parietal
?pal: Possible palatine
pal: Palatine
pes: Pes (foot)
pmx: Premaxilla
porb: Postorbital
pop: Paraoccipital process
prez: Prezygapophysis
prf: Prefrontal
pt: Pterygoid
pub b: Pubic blade
q: Quadrate
qj: Quadratojugal
rad: Radius
retroart pr: Retroarticular process
S?1: Possible first sacral vertebra
S1: First sacral vertebra
S3: Third sacral vertebra
spl: Splenial
s: Squamosal
sub f: Suborbital fenestra
sup fen: Supratemporal fenestra
supraac cr: Supraacetabular crest
supraocc: Supraoccipital
suran: Surangular
t: Isolated tooth
?tib: Possible tibia
tib: tibia
ul: ulna

## Additional Information and Declarations

### Data Availability

The following information was supplied regarding data availability: The raw data are available in the Supplemental Files.

### New Species Registration

The following information was supplied regarding the registration of a newly described species: Publication LSID: urn:lsid:zoobank.org:pub:7CC3CA17-F08F-48AD-9F16-8537B6BAAC1F. Clovesuurdameredeor LSID: urn:lsid:zoobank.org:act:B9FC0E91-9153-4F6B-B4B7-817839A9E7DD. Andrianavoay LSID: urn:lsid:zoobank.org:act:90C7838E-BE28-4615-BB85-BB04B67F1304. Charitomenosuchus LSID: urn:lsid:zoobank.org:act:DE54456D-A305-4A5D-8209-A987982B200C. Neosteneosaurus LSID: urn:lsid:zoobank.org:act:09ADDEA4-AB2B-40A4-AAFF-19819898532F. Plagiophthalmosuchus LSID: urn:lsid:zoobank.org:act:1AC91E3C-FC9A-470B-B9A9-3220B9823C0F. Proexochokefalos LSID: urn:lsid:zoobank.org:act:FC885641-54CC-421D-84E7-0341140EB704. Seldsienean LSID: urn:lsid:zoobank.org:act:A5177ED2-1416-4C54-A169-05591DA55D80. Indosinosuchus kalasinensis LSID: urn:lsid:zoobank.org:act:2B7DB5BB-1F93-457F-A295-0409ECCD3998. Aeolodontinae LSID: urn:lsid:zoobank.org:act:E7A8EDC8-8DF8-4799-AA09-5D6B9287E201. Machimosauridae LSID: urn:lsid:zoobank.org:act:81FB2470-D7E7-4E3E-814B-2AAE996BE5AA. Machimosaurinae LSID: urn:lsid:zoobank.org:act:918AC1F1-AC04-41D1-91DA-E2D25939EAB9.
