# Supplementary material for: The phylogenetics of Teleosauroidea (Crocodylomorpha, Thalattosuchia) and implications for their ecology and evolution
Source: PeerJ. 2020 Oct 8;8:e9808. doi: 10.7717/peerj.9808 (PMC7548081; doi:10.7717/peerj.9808)
Supplement: Supplemental Information 5 [file peerj-08-9808-s005.docx]

Supplementary Data: Detailed Character Descriptions for

THE PHYLOGENETICS OF TELEOSAUROIDEA (CROCODYLOMORPHA, THALATTOSUCHIA) AND IMPLICATIONS FOR THEIR ECOLOGY AND EVOLUTION

*by* M. M. Johnson^1^, M. T. Young^1^, and S. L. Brusatte^1,2^

^1^Grant Institute, School of Geosciences, The King’s Buildings, University of Edinburgh, James Hutton Road, Edinburgh, EH9 3FE, United Kingdom

^2^National Museum of Scotland

S1) New characters pertaining to teleosauroids

S2) Previous characters pertaining to teleosauroids

**S1) New characters pertaining to teleosauroids**

The 38 new characters introduced here were formulated to describe thalattosuchian, specifically teleosauroid, anatomical variation. These characters are relevant to the interrelationships of teleosauroids and many highlight previously unexamined morphological divergence between two large subclades within the group (see below). These characters are new and are here used in a cladistic analysis for the first time, and all states (indicated by a number in brackets) are subsequently figured. Character numbering follows the numbering used in the full list of characters for the present analysis, and corresponding figures are listed.

**12.** Ornamentation on prefrontal in dorsal view: present, with shallow to deep pits and/or grooves (0), or absent (1) (Fig. 29).

This character was inspired by the variety of ornamentation patterns found on the prefrontal of teleosauroid taxa. Ornamentation is either absent (state 1) or comes in the form of shallow to deep pits, in addition to shallow to deep, elongated and thin grooves (state 0). State 1 occurs in very few teleosauroids, including the basal teleosauroid *Plagiophthalmosuchus* (NHMUK PV OR 14792), *I. potamosiamensis* (PRC-11), *Aeolodon* (MNHN.F.CNJ 78), *Sericodon* (Schaefer, Püntener & Billon-Bruyat, 2018), and *Bathysuchus* (Foffa et al., 2019). The majority of teleosauroids are scored as state 0; these include the Chinese teleosauroid (IVPP V 10098), *Platysuchus* (SMNS 9930), *Teleosaurus* (MNHN AC 8746), *Mycterosuchus* (NHMUK PV R 2617), *Macrospondylus* (GPIT-RE-9427; MMG BwJ 565; SMNS 51555), *Clovesuurdameredeor* (NHMUK PV OR 49126), *Seldsienean* (MMT P28-1), *Charitomenosuchus* (NHMUK PV R 3320), *Deslongchampsina* (OUMNH J.29851), *Proexochokefalos* (MNHN.F 1890-13), *Neosteneosaurus* (PETMG R 178) and machimosaurins (*Yvridiosuchus*: OUMNH J.1401; *Lemmysuchus*: LPP.M.21; *Mac. buffetauti*: SMNS 91415). However, in most taxa with state 0, the prefrontal ornamentation is limited, with few shallow grooves and pits. This is observed in *Macrospondylus*, where in many specimens the prefrontal ornamentation is so faint it appears unornamented (e.g. SMNS 51753; SMNS 51957; SMNS 81699). The prefrontal in *Yvridiosuchus* (OUMNH J.1401) displays more ornamentation (mainly grooves) throughout the entirety of the bone, but these grooves are still relatively shallow; this is similar to the condition seen in *Clovesuurdameredeor* (NHMUK PV OR 49126). *Seldsienean* (MMT P28-1) displays prefrontal ornamentation, but it consists mainly of small, scattered, circular pits. In contrast, *Mycterosuchus* (NHMUK PV R 2617) and *Mystriosaurus* (NHMUK PV OR 14781) both display characteristically deep, elongated grooves with little to no pits, and the entirety of the prefrontal is ornamented.

Note that in some taxa (such as *I. kalasinensis*: PRC-239), the prefrontal ornamentation is either poorly preserved or hard to see (either in the specimen or in published figures), and therefore was scored as unknown (?). It is also important to acknowledge that lack of ornamentation has also been attributed to juvenile individuals (see Vignaud, 1995); while there is evidence for this (e.g. juvenile specimens of *Macrospondylus* (SMNS 10 000 and NHMW 1848 0031 0001), in our analysis this character was scored using only adult specimens, as juveniles were excluded from our dataset. Young et al. (2014) noted that adult specimens of *Mac. hugii* had reduced cranial ornamentation, in particular on the premaxillae and maxillae. While not as evident as osteoderm ornamentation, skull ornamentation plays a small role in thermoregulation (e.g. Seidel, 1979; Grigg & Seebacher, 2001) by increasing surface area (Clarac et al., 2015). It is possible that taxa with more ornamented skulls (such as *Mystriosaurus* and *Mycterosuchus*) exhibited more of a terrestrial, basking behaviour than those with less or absent cranial ornamentation (such as *Mac. hugii* and *Aeolodon*), which may have been more aquatic/pelagic. In addition, Young et al. (2013) evaluated the evolution of the dermatocrania in *Torvoneustes coryphaeus*, *Torvoneustes carpenteri* (Wilkinson, Young & Benton, 2008) and associated Late Kimmeridgian metriorhynchids, concluding that reduction and/or loss of dermatocranial ornamentation may have reduced drag, making locomotion through open water more efficient. It is possible that the reduction of dermatocranial ornamentation evolved independently in teleosauroids and allowed them to move more energy efficiently in the water.

**13.** Ornamentation on lacrimal in dorsal view: present (0), with shallow to deep pits and/or grooves, or absent (1) (Fig. 29).

As with the above character, the ornamentation displayed on the lacrimal (=lachrymal) differs between taxa. Ornamentation is either absent (state 1) or comes in the form of shallow to deep pits, as well as shallow to deep, elongated and thin grooves (state 0). The majority of teleosauroids (*Mystriosaurus*: NHMUK PV OR 14781; the Chinese teleosauroid: IVPP V 10098; *Platysuchus*: SMNS 9930; *Teleosaurus*: MNHN AC 8746; *Mycterosuchus*: NHMUK PV R 2617; *Clovesuurdameredeor*: NHMUK PV OR 49126; *Deslongchampsina*: OUMNH J.29851; *Proexochokefalos*: MNHN.F 1890-13; *Lemmysuchus*: NHMUK PV R 3168; *Mac. buffetauti*: SMNS 91415) exhibit state 0, with some form of ornamentation being present. Similar to the prefrontal, the lacrimals of both *Mycterosuchus* (NHMUK PV R 2617) and *Mystriosaurus* (NHMUK PV OR 14781) are extremely ornamented, and have numerous deep, elongated grooves. Generally, in teleosauroids with lacrimal ornamentation, the areas that contribute to the anterior and anterolateral orbital rims have multiple small pits and/or grooves. In *Yvridiosuchus* (OUMNH J.1401), the posterolateral area of the lacrimal (the anterolateral margin of the orbit) is particularly well ornamented with small, shallow pits, so much so that the surface of the bone appears perforated (this is best observed in lateral view).

However, there are exceptions: the lacrimal of the Chinese teleosauroid (IVPP V 10098) is sparsely ornamented, with very few shallow grooves; at first glance, it appears to be unornamented. In *Seldsienean* (MMT P28-1) the lacrimal ornamentation consists mainly of small, scattered, circular pits as in the prefrontal. State 1 (lack of ornamentation) occurs in six taxa: *I. potamosiamensis* (PRC-11), *Aeolodon* (MNHN.F.CNJ 78), *Plagiophthalmosuchus* (NHMUK PV OR 14792), *Macrospondylus* (SMNS 51563), *Charitomenosuchus* (NHMUK PV R 3320) and *Sericodon* (Schaefer, Püntener & Billon-Bruyat, 2018). As discussed in ch. 12, lack of ornamentation has previously been attributed to juveniles (e.g. Vignaud, 1995); however, this character was scored using adult specimens.

It is important to note that the prefrontal and lacrimal ornamentation characters are not dependent on each other. For example, *Charitomenosuchus* (NHMUK PV R 3320) is scored as having ornamentation on the prefrontal (state 0), but lacking ornamentation on the lacrimal (state 1). Lack or presence of ornamentation may also be attributed to habitat preference (see above).

**15.** Frontal, extension of ornamentation: extends from the centre of the frontal to lateral- and anterior-most regions (0), restricted to centre of the frontal (1) or no ornamentation (2) (Fig. 29).

The frontal of teleosauroids is a single bone that is consistently ornamented throughout the majority of the group, excluding *Bathysuchus* (unnumbered LPP specimen) and juveniles, such as SMNS 10 000 and NHMUK PV R 2074 (although this individual still displays weak, minimalistic ornamentation at the centre of the frontal). Ornamentation consists of pits and/or elongated grooves (typically a combination of the two), which are often deep and well-developed. The range of this ornamentation varies between taxa, either extending from the centre of the frontal to the anterior- and lateral-most areas (state 0) or is restricted to the midline or centre of the frontal (state 1), with minimal extension.

*Plagiophthalmosuchus* (NHMUK PV OR 14792), *Clovesuurdameredeor* (NHMUK PV OR 49126), *Macrospondylus* (MMG BwJ 565; SMNS 51563) and many basal teleosauroids (*Mystriosaurus*: NHMUK PV OR 14781; the Chinese teleosauroid: IVPP V 10098; *Indosinosuchus*: PRC-11, PRC-239; *Platysuchus*: SMNS 9930; *Teleosaurus* MNHN AC 8746; *Mycterosuchus*: NHMUK PV R 2617), display state 0; in general (e.g. *Mystriosaurus*: NHMUK PV OR 14781; *Macrospondylus*: MMG BwJ 565), the pits/grooves are very closely packed together, sometime merging into one another. However, in *Plagiophthalmosuchus* (MNHNL TU515) and the Chinese teleosauroid (IVPP V 10098), these irregularly shaped pits are noticeably well spaced apart from one another. In *Clovesuurdameredeor* (NHMUK PV OR 49126), the pits/grooves are deeper at the centre of the frontal and become shallower when radiating outwards; however, they are still present at the anterior- and lateral-most areas of the frontal, noticeably the anterior area, where they contact the nasal-frontal suture. The majority of more derived teleosauroids (*Charitomenosuchus*: NHMUK PV R 3320; *Seldsienean*: MMT P28-1; *Deslongchampsina*: OUMNH J.29851; *Proexochokefalos*: MNHN.F 1890-13; *Neosteneosaurus*: PETMG R178; *Yvridiosuchus*: OUMNH J.1401; *Lemmysuchus*: LPP.M.21; *Mac. buffetauti*: SMNS 91415), along with *Sericodon* (SCR010312 in Schaefer, Püntener & Billon-Bruyat, 2018) and *Aeolodon* (MNHN.F.CNJ 78), share state 1. In *Yvridiosuchus* (OUMNH J.1401), the pits do extend laterally, but do not reach the lateral- or anterior-most region of the frontal; this taxon was still scored as state 1.

It has been suggested that *Bathysuchus* lacks any frontal ornamentation (Vignaud, 1995), similar to juvenile individuals. However, there may possibly be weak, nearly unnoticeable pits and grooves restricted to the midline of the frontal in this taxon (Fig.), in an LPP unnumbered specimen (Foffa et al., 2019). Due to this uncertainty, this taxon was scored as (?). This is similar to the condition seen in *Steneosaurus hulkei* (= *Neosteneosaurus* edwardsi) Andrews, 1913 (NHMUK PV R 2074), which is considered to be a sub-adult; however, whether or not this indicates that the LPP *Bathysuchus* (Foffa et al., 2019) is a sub-adult is beyond the scope of this manuscript, as more work into teleosauroid ontogeny is required.

**43.** Premaxilla in dorsal view, the total anteroposterior length relative to total rostrum length is less than 25% (0) or approximately 25% or greater (1) (Fig. 30).

This character focuses on the total anteroposterior premaxillary length in relation to the total anteroposterior rostrum length of a cranium. When defining the rostral length, this refers to the length between the anterior-most premaxillae to the anterior orbital margin. This character is related to the traditional classification of a rostrum as either longirostrine or mesorostrine. Longirostry refers to the preorbital length being 70% or more of the total basicranial length, and mesorostry states that the preorbital length is approximately 55-70% of the total basicranial length (Young & Andrade, 2009).

In the majority of teleosauroids, the premaxillary anteroposterior length is greater than 25% relative to the rostral length (state 1). This condition is observed in the basal teleosauroid *Plagiophthalmosuchus* (NHMUK PV OR 14792), as well as all taxa that are, in technical terms, longirostrine (e.g. *Indosinosuchus*: PRC239; *Mycterosuchus*: NHMUK PV R 2617; *Aeolodon*: MNHN.F.CNJ 78; *Macrospondylus*: SMNS 18672; *Proexochokefalos*: MNHN.F 1890-13; *Lemmysuchus*: NMHUK PV R 3168). Few teleosauroids have a premaxillary anteroposteriorly length that is less than 25% of the rostral length (state 0). This is observed in *Mac. buffetauti* (SMNS 91415) and *Mac. mosae* (IRSNB cast; Hua, 1999) as well as *Mystriosaurus* (NHMUK PV OR 14781) and the Chinese teleosauroid (IVPP V 10098).

This character is particularly intriguing regarding thalattosuchians as a clade. In teleosauroids, purely mesorostrine taxa (*Mystriosaurus*, the Chinese teleosauroid and *Machimosaurus*) exhibit the basal condition (state 0), whereas the longirostrine taxa (including the basal-most teleosauroid) are state 1. This differs from that seen in Metriorhynchoidea; state 0 is seen in the basal metriorhynchoids *Pelagosaurus* and *Teleidosaurus calvadosii* Eudes-Deslongchamps, 1866b, whereas state 1 is a defining character in Metriorhynchidae, in which some members significantly shorten their snouts. In metriorhynchids, the premaxillae noticeably elongate, so much so that they nearly come into contact with the nasals in more derived forms (e.g. Metriorhynchus LPP.M.48). However, in teleosauroids, both the premaxillae and maxillae are elongated, so the relative proportions of these bones to one another do not change. This way, the premaxillae never come close to contacting the nasals. Developmentally, there appears to be a change between these two clades in the shortening of the snout, which warrants further investigation.

**56.** Premaxilla in dorsal view, the anterior and posterior medial margins of the external nares are formed by two bulbous projections, which are either absent (0) or present (1) (Fig. 31).

In most teleosauroids, the medial margins of the external nares are minimally convex (more noticeable in the posterior margin than the anterior margin) (state 0), causing the external nares to appear D-shaped in dorsal view. This is the condition seen in the basal *Plagiophthalmosuchus* (NHMUK PV OR 14792) in addition to *Mystriosaurus* (NHMUK PV R OR 14781), *Indosinosuchus* (PRC11; PRC-239), the Chinese teleosauroid (IVPP V 10098), *Platysuchus* (SMNS 9930), *Macrospondylus* (MMG BwJ 565), *Charitomenosuchus* (NHMUK PV R 3806), *Proexochokefalos* (MNHN.F 1890-13), *Neosteneosaurus* (NHMUK PV R 2865) and Machimosaurini (*Yvridiosuchus*: OUMNH J.1401; *Lemmysuchus*: NHMUK PV R 3168; *Mac. buffetauti*: SMNS 91415). In *Deslongchampsina* (OUMNH J.29851), the external narial margins have no convexity, and are relatively straight and vertical.

In certain taxa, however, both the anterior and posterior margins are strongly convex, and appear ‘bulging’ in dorsal view. These bulbous projections extend laterally from the medial margins of the external nares, which causes them to appear distinctly ‘8’-shaped. This condition (state 1) is synapomorphic in a unique clade containing *Mycterosuchus* (NHMUK PV R 2617), *Bathysuchus* (unnumbered LPP specimen) (Foffa et al., 2019), and possibly *Aeolodon* (MNHN.F.CNJ 78) (however, specimens of this taxon are dorsoventrally crushed and slightly distorted, so it is difficult to say with certainty if it is present). It is interesting to note that the external nares of *Mystriosaurus* (NHMUK PV OR 14781) are in a way similar to state 1 in that they are more mediolaterally expanded and slightly bulging; however, the posterior margin is noticeably more bulbous (as in taxa with state 0) than the anterior margin, giving the external nares a slight ‘D’-shape in dorsal view. Some specimens of *Macrospondylus* (e.g. SMNS 59736) appear to have state 1, but these are dorsoventrally crushed; this distorts the bones, making it appear as though the medial margins are bulbous whereas in reality they are not. This character was briefly introduced and discussed in Foffa et al. (2019) but was not included in the supplementary appendix.

**58.** Premaxilla in dorsal view, the shape of the anteroposterior premaxilla-maxilla contact is triangular (0), subcircular (1) or ‘ragged’ (2) (Fig. 31).

Surprisingly, the premaxilla-maxilla contact differs between teleosauroid genera, with no clear explanation as to why. In the basal-most form (*Plagiophthalmosuchus*: NHMUK PV OR 14792), as well as the Chinese teleosauroid (IVPP V 10098); *Indosinosuchus* (PRC-11; PRC-239); *Platysuchus* (SMNS 9930); *Aeolodon* (MNHN.F.CNJ 78), *Mycterosuchus* (NHMUK PV R 2617), *Bathysuchus* (unnumbered LPP specimen) and *Macrospondylus* (SMNS 51753; SMNS 51984), the contact is triangular with slight or no interdigitating areas (state 0). An intermediate condition (state 1) shows the contact to be anteroposteriorly short and subcircular in shape (more posteromedially horizontally oriented than state 0), with a weak to moderate degree of interdigitating regions, generally close to the midline of the rostrum. This occurs in *S. rostromajor* (MNHN.RJN 134c-d) as well as *Mystriosaurus* (NHMUK PV OR 14781), *Andrianavoay* (NHMUK PV R 1999), *Proexochokefalos* (MNHN.F 1890-13), *Neosteneosaurus* (NHMUK PV R 2865) and members of Machimosaurini (*Yvridiosuchus*: OUMNH J.1401; *Lemmysuchus*: NHMUK PV R 3168, LPP.M.21; *Machimosaurus*: SMNS 91415). A third condition (state 2) is found in *Charitomenosuchus* (NHMUK PV R 3320, NHMUK PV R 3806) and *Seldsienean* (MMT P28-1): the premaxilla-maxilla suture is anteroposteriorly elongated, sub-rectangular and highly interdigitating, giving it a ‘ragged’-like appearance. In *Seldsienean* (MMT P28-1), the interdigitating areas of the suture are more laterally spread out, while in *Charitomenosuchus* (NHMUK PV R 3320) they are closer together.

As touched upon when describing cranial ornamentation, in juveniles the premaxilla-maxilla suture is expected to not be as strongly integrated as in adults (e.g. NHMW 1848-0031-0001), although the posterior-most area of the suture is jagged in young *Gavialis* specimens (Gold, 2011). However, as mentioned previously, no juveniles were scored for our dataset.

**64.** Nasals, elongate posterior process that does not (0) or does (1) contact anterior rim of orbit (Fig. 32).

In the majority of teleosauroids (e.g. the Chinese teleosauroid: IVPP V 10098; *Platysuchus*: SMNS 9930; *Mycterosuchus*: NHMUK PV R 2617; *Seldsienean*: MMT P28-1; *Deslongchampsina*: OUMNH J.29851; *Neosteneosaurus*: NHMUK PV R 3701; *Lemmysuchus*: LPP.M.21), including the basal-most teleosauroid (*Plagiophthalmosuchus*: NHMUK PV OR 14792), the posterior processes of the nasals reach or extend slightly past the anterior rim of the orbits (state 0). In addition, these processes are positioned medially, slightly mediolaterally thin in the posterior-most area, and do not come into close contact with the medial orbital margin. They are shorter and broader in *Clovesuurdameredeor* (NHMUK PV OR 49126) than any other teleosauroid and are relatively short (but not as broad) in *Mystriosaurus* (NHMUK PV OR 14781). In *Yvridiosuchus* (OUMNH J.1401), these processes do reach past the anterior orbital rim but not substantially, are medially broad and do not contact the medial rim of the orbit; this taxon was therefore scored as state 0. However, *I. potamosiamensis* (PRC-11) clearly possesses state 1, in which the nasals have extraordinarily anteroposteriorly elongated posterior processes; these are mediolaterally thin and come into contact with the medial rim of the orbit.

This feature was briefly touched upon in Martin et al. (2019); it was referred to as present in all *Indosinosuchus* taxa, because of it being noticeable in PRC-11 (the designated holotype of *I. potamosiamensis*). However, *I. kalasinensis* (PRC-239) is poorly preserved in that area, and therefore it is currently scored as unknown (?) in our dataset.

**124.** Frontal, anteromedial process shape and length relative to nasals: anterior projection of frontal is mediolaterally broad and does not extend far anteriorly past anterior orbital rim into nasals (0) or anterior projection of frontal is mediolaterally thin and extends anteriorly past anterior orbital rim into nasals (1) (Fig. 32).

This character focuses on the relative shape, width and length of the frontal anteromedial process in relation to the nasals. In the majority of teleosauroids, this process is triangular, thin and anteromedially elongated, usually extending past the anterior orbital margin (state 1). This is seen in taxa such as the basal-most form *Plagiophthalmosuchus* (NHMUK PV OR 14792) as well as *Mystriosaurus* (NHMUK PV OR 14781), the Chinese teleosauroid (IVPP V 10098), *Indosinosuchus* taxa (PRC 11; PRC 239), *Platysuchus* (SMNS 9930), *Mycterosuchus* (NHMUK PV R 2617), *Aeolodon* (MNHN.F.CNJ 78), *Macrospondylus* (MMG BwJ 565; SMNS 51555), *Charitomenosuchus* (NHMUK PV R 3320), *Deslongchampsina* (OUMNH J.29851), *Proexochokefalos* (MNHN.F 1890-13), *Neosteneosaurus* (MNHN.RJN 118; PETMG R178) and Machimosaurini (*Yvridiosuchus* OUMNH J.1401; *Lemmysuchus* LPP.M.21; *Mac. buffetauti* SMNS 91415). It is interesting to note that the anteromedial frontal processes in *Yvridiosuchus*, *Indosinosuchus*, *Charitomenosuchus* and *Mac. buffetauti* are considerably more elongated and mediolaterally thin than in the other aforementioned taxa.

The processes seen in *Teleosaurus* (MNHN AC 8746) and *Sericodon* (SCR010-312 in Schaefer, Püntener & Billon-Bruyat, 2018) appear to be state 1; however, both taxa were scored as {01}. Only one taxon, *Clovesuurdameredeor* (NHMUK PV OR 49126), expresses state 0, in which the anteromedial frontal process is noticeably mediolaterally broadened (giving it a subcircular appearance in dorsal view) and anteroposteriorly short; therefore, this character state is currently autapomorphic to *Clovesuurdameredeor*.

**125.** Frontal in dorsal view, small anterolateral projections between nasals and prefrontals are absent (0) or present (1) (Fig. 32).

The frontal projections are two small triangular-shaped intrusions of bone that are anterolaterally oriented, situated between the prefrontals and nasals, and only observed in dorsal view. Most teleosauroids do not have these extra projections; instead, the frontal suture is flush with that of the posterior nasal processes (state 0). This condition is clearly seen in the basal teleosauroid *Plagiophthalmosuchus* (NHMUK PV OR 14792) and the Chinese teleosauroid (IVPP V 10098), *Indosinosuchus* (PRC-11, PRC-239), *Platysuchus* (SMNS 9930), *Teleosaurus* (MNHN AC 8746), *Mycterosuchus* (NHMUK PV R 2617), *Aeolodon* (MNHN.F.CNJ 78), *Macrospondylus* (MMG BwJ 565), *Clovesuurdameredeor* (NHMUK PV OR 49126), *Charitomenosuchus* (NHMUK PV R 3320), *Deslongchampsina* (OUMNH J.29851), *Proexochokefalos* (MNHN.F 1890-13), *Neosteneosaurus* (NHMUK PV R 2865), *Yvridiosuchus* (OUMNH J.1401) and *Lemmysuchus* (LPP.M.21). The presence of these frontal projections is an apomorphic state, however, in the taxon *Mac. buffetauti* (Martin & Vincent, 2013; SMNS 91415), in which they are large, mediolaterally broadened and clearly noticeable (state 1). *Clovesuurdameredeor* (NHMUK PV OR 49126) may also have state 1, but it is uncertain if this represents a true suture or a fracture (the latter appears to be more likely). Therefore, this taxon is currently scored as state 0.

**167.** Jugal anterior process is absent (0) or is slender, elongated and extends anteriorly (1) (Fig. 33).

The jugal is a large, triradiate bone, with the anterior area forming the lateral border and ventral margin of the orbits. The majority of teleosauroids have a shortened anterior process of the jugal that does not extend past the anterior orbital margin (state 0). This is clearly seen in the basal form *Plagiophthalmosuchus* (MNHNL. TU515) as well as *Mystriosaurus* (NHMUK PV OR 14781), the Chinese teleosauroid (IVPP V 10098), *Platysuchus* (SMNS 9930), *Teleosaurus* (MNHN AC 8746), *Mycterosuchus* (NHMUK PV R 2617), *Macrospondylus* (PMU R161) and *Deslongchampsina* (OUMNH J.29851). In *Deslongchampsina* (OUMNH J.29851), the anterior jugal process is marginally extended past the anterior orbital margin, but barely and it is still shortened and rounded; therefore, it is scored as state 0.

However, an interesting feature in most Oxford Clay Formation (OCF) and equivalent taxa is that the anterior jugal becomes dorsoventrally curved, narrow and anteroposteriorly elongated, and extends substantially past the anterior orbital margin, at times nearly to the posterior region of the antorbital fenestra. Johnson et al. (2017) first highlighted and figured this character, best seen in lateral view, in *Lemmysuchus* (PETMG R39). This condition (state 1) is also present in the taxa *Charitomenosuchus* (NHMUK PV R 3320), *Neosteneosaurus* (MNHN.RJN 118; PETMG R178), *Proexochokefalos* (MNHN.F 1890-130) and other members of Machimosaurini in addition to *Lemmysuchus* (*Yvridiosuchus*: OUMNH J.1401; *Mac. buffetauti*: SMNS 91415). This anterior process is particularly long in *Yvridiosuchus* (OUMNH J.1401) and encounters the posterior-most margin of the antorbital fenestra. However, *Mycterosuchus*, another OCF taxon, is currently scored as 0; the holotype (NHMUK PV R 2617) does not appear to have this condition. However, another specimen (CAMSM J.1420) currently thought to be attributed to this genus displays state 1 and is currently being investigated.

184. Maxilla in palatal view, shape of anterior maxilla is tapering (subtriangular) (0) or straightened (sub-rectangular) (1) (Fig. 34).

This character focuses on the anterior premaxilla-maxilla contact in palatal view, which is positioned parallel to the fourth premaxillary alveolus. State 1 is a synapomorphic character for members of Teleosauroidea (e.g. the Chinese teleosauroid: IVPP V 10098; *Yvridiosuchus*: OUMNH J.1401); the contact is horizontal and straight, and sub-rectangular in shape. This character is one key difference from Metriorhynchoidea, in which the contact is subtriangular and anteriorly directed (state 0) (e.g. *Metriorhynchus superciliosus*: LPP.M.48).

**208.** Paraoccipital process approximately the same size (0) or substantially larger than the remainder of the exoccipital-opisthotic (1) (Fig. 35).

The paraoccipital processes are the posterior-most part of the exoccipital-opisthotics, which makes up most of the occiput; they contact the supraoccipital dorsally and forms the dorsal and lateral margins of the foramen magnum (Brusatte et al., 2016; Johnson et al., 2018). The paraoccipital processes are pronounced, horizontally directed and have a circular or straightened (e.g. *Plagiophthalmosuchus*: MNHNL TU515) distal margin. The paraoccipital processes are approximately the same size as the rest of the exoccipital-opisthotic (state 0). This is seen in the basal form *Plagiophthalmosuchus* (MNHNL TU515) as well as most teleosauroids (the Chinese teleosauroid: IVPP V 10098; *Indosinosuchus*: PRC-11, PRC-239; *Platysuchus*: SMNS 9930; *Teleosaurus*: MNHN AC 8746; *Mycterosuchus*: NHMUK PV R 2617; *Bathysuchus*: unnumbered LPP specimen; *Sericodon*: SCR010-312 in Schaefer, Püntener & Billon-Bruyat, 2018; *Macrospondylus*: SMNS 81699; *Clovesuurdameredeor*: NHMUK PV OR 49126; *Charitomenosuchus*: NHMUK PV R 3320; *Deslongchampsina*: OUMNH J.29851; *Proexochokefalos*: MNHN.F 1890-13; *Neosteneosaurus*: PETMG R178; *Yvridiosuchus*: OUMNH J.29850; *Lemmysuchus*: NHMUK PV R 3168; *Mac. buffetauti*: SMNS 91415; *Mac. mosae*: Young et al., 2014). The paraoccipital processes may be marginally smaller than the remaining exoccipital-opisthotics in few teleosauroids (*Plagiophthalmosuchus*: MNHNL TU515; *Mac. buffetauti*: SMNS 91415). In *Mac. hugii* (MG-8730-2), the paraoccipital processes are noticeably and substantially larger than the remaining exoccipital-opisthotics; this condition (state 1) is autapomorphic for this taxon. Due to these large, expanded paraoccipital processes, the cervicocranial depressor muscles (which attach to the processes) would have been well-developed, possibly assisting *Mac. hugii* with diving (Krebs, 1968; Young et al., 2014).

**269.** Splenials in dorsal view, the excavation of Meckelian groove on the dorsal surface of symphyseal splenials is deep (0) or shallow (1) (Fig. 36).

This character focuses on the excavation of the Meckelian groove (=canal) seen on the dorsal surface of the symphyseal splenials. The Meckelian groove is the area of the lower jaw where the posterior two halves articulate and exposes the Meckelian cartilage (an attachment surface for muscles that assist in closing the jaw) (Holliday & Nesbitt, 2013). This is a key feature that helps in distinguishing one particular subgroup of teleosauroids (including *Proexochokefalos*, *Neosteneosaurus* and Machimosaurini) from the rest of Teleosauroidea.

In more basal and longirostrine teleosauroids (e.g. *Teleosaurus*: Eudes-Deslongchamps, 1867-69; *Mycterosuchus*: NHMUK PV R 2617; *Macrospondylus*: SMNS 53422; *Seldsienean*: OUMNH J.1414; *Charitomenosuchus*: NHMUK PV R 3806), the Meckelian groove is anteroposteriorly long relative to jaw length and deeply excavated (state 1). In the taxa *Proexochokefalos* (MNHN.F 1890-13), *Neosteneosaurus* (NHMUK PV R 3701) and Machimosaurini (*Yvridiosuchus*: OUMNH J.1417; *Lemmysuchus*: LPP.M.21; *Machimosaurus*: SMNS 91415; NMS 7021 in Young et al., 2014), the Meckelian groove is shallow with little to no excavation (state 0). This feature of teleosauroids was first discussed by Johnson et al. (2017), and then again in Johnson et al. (2019), but was not included in the supplementary appendices.

Holliday & Nesbitt (2013) suggested that differences in mandibular symphysis anatomy, including those in the Meckelian groove, are indicative of a major shift in diet and cranial functions. This, paired with a modification towards larger supratemporal fenestrae and more robust skulls and teeth (excluding the dentition of *Proexochokefalos*), indicate the changing of prey preferences in a unique subclade of teleosauroids (see below), and the modifications in the skull that allow them to acquire said prey. In addition, Holliday & Nesbitt (2013) also noted that fusing the opposing mandibular symphyses acts as a strengthening mechanism (against torsion and maintaining force transference between both sides of the jaw); while both halves of the jaw are unfused in all teleosauroids, this joint is significantly less perceptible in *Neosteneosaurus* (NHMUK PV R 3701) and machimosaurins (e.g. *Yvridiosuchus*: OUMNH J.1417; *Lemmysuchus*: LPP.M.21).

**270.** Angular dorsal curvature is gradual (0) or sharp and abrupt (1) (Fig. 37).

In most teleosauroids, the angular is an elongated, robust bone of the posterior mandible that occupies substantially more area than the articulating surangular. It is dorsoventrally deep, especially in *Proexochokefalos* (MNHN.F 18901-13) and machimosaurins (*Yvridiosuchus*: OUMNH J.29850; *Lemmysuchus*: NHMUK PV R 3168; *Machimosaurus*: IRSNB cast), forms the posteroventral and ventral borders of the mandibular fenestra, and contacts the articular posteriorly and dentary anteriorly. Specifically, the posterior angular contacts the retroarticular process of the articular. In the majority of teleosauroids, the ventral margin of the angular gradually curves posterodorsally (state 0). This condition is seen in *Indosinosuchus* (PRC-11; PRC-239), *Platysuchus* (SMNS 9930), *Sericodon* (SCR010-1184 in Schaefer, Püntener & Billon-Bruyat, 2018), *Aeolodon* (MNHN.F.CNJ 78), *Macrospondylus* (SMNS 51753), *Charitomenosuchus* (NHMUK PV R 3806) and *Seldsienean* (OUMNH J.1414). Both *Plagiophthalmosuchus* (MNHNL TU515; NHMUK PV OR 15500) and *Mystriosaurus* (NHMUK PV OR 14781) also display state 0; however, the anterior-most angular is straight (horizontally directed), and the dorsoposterior curvature is poor and limited to the posterior area.

The curvature of the angular differs in *Proexochokefalos* (MNHN.F 1890-13), *Neosteneosaurus* (PETMG R178) and Machimosaurini (*Yvridiosuchus*: OUMNH J.29850; *Lemmysuchus*: NHMUK PV R 3168; *Machimosaurus*: IRSNB cast, SMNS 91415), in which the dorsoposterior curvature is immediate, sharp and abrupt (state 1). This curve mainly influences the attachments of the *m. pterygoideus ventralis* on the lateral and ventral sides, as well as the m. depressor mandibulae on the dorsal side, which aid in closing the jaw (Holliday, 2006; Holiday et al., 2013). This character is best observed in lateral view.

The differences in this character was first highlighted and figured by Johnson et al. (2017), and subsequently in Johnson et al. (2018), Martin et al. (2019), Johnson et al. (2019) and Sachs et al. (2019a), but was not included in the phylogenetic analyses.

**291.** Maxilla, reception pits are either absent, shallow throughout, or conspicuous only in the anterior maxilla (0) or pronounced and deep throughout the entirety of the maxilla (1) (Fig. 38).

The maxillae are two paired, elongate, tooth-bearing bones that represent a substantial part of the rostrum in crocodylomorphs, notably in longirostrine forms. Along the lateral surface of the maxillae, reception pits for the opposing tooth row (dentary) are visible, parallel and slightly dorsally positioned to the tooth row. State 0 includes taxa that have either shallow or absent reception pits; however, it is important to note that reception pits are present in all teleosauroids, so for the purposes of this analysis, state 0 of character 291 focuses purely on taxa with shallow reception pits. These may vary substantially in terms of noticeability; for example, they are present but near invisible in the basal taxon *Plagiophthalmosuchus* (MNHNL TU515) and are relatively shallow in most taxa (*Mystriosaurus*: NHMUK PV OR 14781; *Indosinosuchus*: PRC 11, PRC-239; the Chinese teleosauroid: IVPP V 10098; *Platysuchus*: SMNS 9930; *Teleosaurus*: MNHN AC 8746, Eudes-Deslongchamps, 1867-69; *Mycterosuchus*: NHMUK PV R 2617; *Bathysuchus*: unnumbered LPP specimen; *Sericodon*: BSY008-622 in Schaefer, Püntener & Billon-Bruyat, 2018; *Aeolodon*: MNHN.F.CNJ 78; *Macrospondylus*: SMNS 51753, SMNS 51957). In these taxa, the reception pits are noticeable but small and shallow throughout the anterior and mid-maxilla, and gradually disappear posteriorly. *Mystriosaurus* (NHMUK PV OR 14781) is interesting because the reception pits are relatively deep at the mid-maxilla (however, this may be due to preservation), and *I. kalasinensis* (PRC 239) has small, shallow reception pits throughout the entirety of the maxilla. In addition, state 0 includes taxa that have noticeable reception pits in the anterior region of the maxilla that gradually disappear towards the mid-to posterior regions. This condition is seen in the genera *Deslongchampsina* (OUMNH J.29851) and *Charitomenosuchus* (NHMUK PV R 3806); the reception pits are well developed and deep in the anterior maxilla, but they quickly disappear by the middle of the maxilla.

In some taxa, however, the reception pits are deep and noticeable throughout the near-entirety or entirety of the maxilla, notably so in the anterior and middle regions, although they do become smaller when progressing posteriorly. This condition is seen in machimosaurins (*Yvridiosuchus*: OUMNH J.29850; *Lemmysuchus*: NHMUK PV R 3618; *Mac. buffetauti*: SMNS 91415) as well as *Andrianavoay* (NHMUK PV R 1999), *S. rostromajor* (MNHN.RJN 134c-d, to some extent) and large individuals of *Neosteneosaurus* (PETMG R178). In machimosaurins (*Yvridiosuchus*: OUMNH J.29850; *Lemmysuchus*: NHMUK PV R 3618; *Mac. buffetauti*: SMNS 91415), the reception pits are small but deep until the posterior-most maxilla, whereas in *Andrianavoay* (NHMUK PV R 1999), *S. rostromajor* (MNHN.RJN 134c-d) and *Neosteneosaurus* (PETMG R178) they become small and shallow but are still present after the mid-maxilla. Young et al. (2014) first highlighted the presence of deep maxillary reception pits in the genus *Machimosaurus*. Subsequent studies (Johnson et al., 2017; Johnson, Young & Brusatte, 2019; Sachs et al., 2019a) noted the characteristics of these reception pits, but did not include them in the phylogenetic analyses.

**292.** Premaxilla, P1-P2 either does not form a couplet and the interalveolar spacing between P1-P2 and P3-P4 relatively the same size (0) or forms a couplet with the interalveolar spacing between P1-P2 and P3-P4, with P1-P2 being separated by a thin lamina and P3-P4 being well separated (1) (Fig. 39).

In palatal view, there are four premaxillary alveoli in the majority of teleosauroids. This excludes members of *Machimosaurus* (Young et al., 2014), which have three, as well as *Platysuchus*, *Bathysuchus*, *Teleosaurus* and *Sericodon*, which are all reported to have five (Lamouroux, 1820; von Meyer, 1845; Eudes-Deslongchamps, 1869; Westphal, 1961; Westphal, 1962; Johnson et al., 2018; Schaefer, Püntener & Billon-Bruyat, 2018). The first (P1) and second (P2) premaxillary alveoli are situated anterior to the third (P3) and fourth (P4), which are positioned posterolaterally. The fifth (P5) premaxillary alveolus (present in *Bathysuchus*, *Sericodon* and *Platysuchus*) is positioned dorsally in comparison to the P1 to P4 (Foffa et al., 2019). As such, the interalveolar distance varies between these alveoli. The P1 and P2 can be well separated in a way similar to that between the P3 and P4; the interalveolar spacing is large and noticeable, with the adjacent alveoli at a further distance from one another. This condition (state 0) occurs in *Platysuchus* (MNHNL TU895), *Sericodon* (SCR011-406 in Schaefer, Püntener & Billon-Bruyat, 2018), *Bathysuchus* (DORCM G.05067i) and *Mycterosuchus* (CAMSM J.1420).

In contrast, in the majority of teleosauroids the P3 and P4 remain separate, but the P1 and P2 are situated closely together and are either separated by a small, thin interalveolar lamina, or appear slightly merged together, thereby creating a P1-P2 ‘couplet’ (state 1). This state is seen in *Mystriosaurus* (NHMUK PV OR 14781), the Chinese teleosauroid (IVPP V 10098), *I. potamosiamensis* (PRC-11) and one subclade of teleosauroids (*Macrospondylus* SMNS 18672; *Charitomenosuchus*: NHMUK PV R 3806; *Deslongchampsina*: OUMNH J.29851; *Proexochokefalos*: MNHN.F 1890-13; *Neosteneosaurus*: NHMUK PV R 2865; *Yvridiosuchus*: OUMNH J.1401; *Lemmysuchus*: NOTNH FS3361), excluding *Machimosaurus*. In *I. potamosiamensis* (PRC-11), *Charitomenosuchus* (NHMUK PV R 3806) and the Chinese teleosauroid (IVPP V 10098), this interalveolar lamina between the P1-P2 is extremely thin.

Note that this character is not applicable for taxa that have fewer than four premaxillary alveoli; therefore, this character is inapplicable for the genus *Machimosaurus* (or the Teleosauroidea indeterminate Luxembourg specimen MNHNL TU164). The Chinese teleosauroid (IVPP V 10098) was initially thought to have three premaxillary alveoli (Li, 1993; Wilberg, 2015a), but in actuality, the P1 is very small, and a barely noticeable (due to poor preservation) interalveolar lamina separates it from the P2. Unfortunately, the anterior premaxilla is not preserved in either *S. rostromajor* (MNHN.RJN 134c-d) or *Andrianavoay* (NHMUK PV R 1999), two important fragmentary taxa that muddy the phylogenetic relationship between *Neosteneosaurus* and Machimosaurini. Johnson et al. (2017) briefly drew attention to the feature by examining it in *Lemmysuchus* (LPP.M.21; NOTNH FS3361). Foffa et al. (2019) then did so with *Bathysuchus* and closely related taxa; however, this character was not included in the phylogenetic analysis. This character is in connection with ch. 293, 294 and 295.

**293.** Premaxilla, P3-P4 couplet is present (0) or absent (1) (Fig. 39).

In most teleosauroids, the interalveolar spacing is generally noticeable and well-developed between the P3 and the P4, but it is usually small (possibly due to both alveoli being quite large); the alveoli are therefore closely spaced together, forming a couplet (state 0). This is present in most teleosauroids (*Mystriosaurus*: NHMUK PV OR 14781; *I. potamosiamensis*: PRC-11; *Platysuchus*: MNHNL TU895; *Mycterosuchus*: CAMSM J.1420; *Macrospondylus* SMNS 81699; *Charitomenosuchus*: NHMUK PV R 3806; *Deslongchampsina*: OUMNH J.29851; *Proexochokefalos*: MNHN.F 1890-13; *Neosteneosaurus*: NHMUK PV R 2865; *Yvridiosuchus*: OUMNH J.1401; *Lemmysuchus*: NOTNH FS3361). State 1 is found in both *Bathysuchus* (NHMUK PV OR 43086, DORCM G.05067i) and the Chinese teleosauroid (IVPP V 10098), in which the P3-P4 are widely spaced apart from one another, and therefore do not form a couplet.

As with ch. 292, this character is not applicable for taxa (members of the genus *Machimosaurus*) that have fewer than four premaxillary alveoli. This character is in connection with ch. 292, 294 and 295.

**294.** Premaxilla in palatal view, both P1 and P2 are oriented anteriorly (0), P1 is oriented anteriorly and P2 slightly medially (1), or both P1 and P2 are oriented laterally (2) (Fig. 39).

The orientation of the first two premaxillary alveoli differs between certain teleosauroids and may have certain phylogenetic implications. In many teleosauroids, both the P1 and P2 are oriented anteriorly (state 0). This occurs in *Mystriosaurus* (NHMUK PV OR 14781), *I. potamosiamensis* (PRC11), *Platysuchus* (MNHNL TU895), *Macrospondylus* (SMNS 18672), *Deslongchampsina* (OUMNH J.29851), *Neosteneosaurus* (NHMUK PV R 28650), *Yvridiosuchus* (OUMNH J.1401) and *Lemmysuchus* (NOTNH FS3361). In a second condition (state 1), the P1 is oriented anteriorly, but the P2 is oriented slightly medially. This is seen in *Charitomenosuchus* (NHMUK PV R 3806) and *Proexochokefalos* (MNHN.F 1890-13). A third condition (state 2), which occurs in *Bathysuchus* (Foffa et al., 2019), *Sericodon* (SCR011-406 in Schaefer, Püntener & Billon-Bruyat, 2018) and *Mycterosuchus* (CAMSM J.1420), is that the P1 and P2 are both strongly oriented laterally, appearing almost horizontally placed.

Foffa et al. (2019) initially noted that the positioning of premaxillary alveoli had phylogenetic importance, which was one of the factors contributing to the construction of this character. As with ch. 292-293, this character is not applicable for taxa (members of the genus *Machimosaurus*) that have fewer than four premaxillary alveoli. This character is in connection with ch. 292, 293 and 295.

**295.** Premaxilla, both P1 and P2 do not form a couplet and are either not oriented on the anterior margin of the premaxilla (0) or are oriented on the anterior margin of the premaxilla (1) (Fig. 39).

In certain teleosauroids, if the P1-P2 alveolar complex does not form a couplet, these two alveoli are positioned either on or slightly ventral to the anterior margin of the premaxilla. In *Platysuchus* (SMNS 9930), the P1 and P2 do not form such a couplet and both alveoli are not oriented on the anterior margin of the premaxilla (state 0). However, in the genera *Bathysuchus* (DORCM G.05067i, unnumbered LPP specimen), *Sericodon* (SCR011-406 in Schaefer, Püntener & Billon-Bruyat, 2018) and *Mycterosuchus* (CAMSM J.1420), the P1 and P2 do not form a couplet but are noticeably oriented on the anterior margin of the premaxilla (state 1).

As with ch. 292 to 294, this character is not applicable for taxa that have fewer than four premaxillary alveoli (members of the genus *Machimosaurus*), or for taxa that have the P1-P2 alveolar couplet (e.g. *I. potamosiamensis*: PRC-11; the Chinese teleosauroid: IVPP V 10098; *Proexochokefalos*: MNHN.F 1890-13; *Neosteneosaurus*: NHMUK PV R 2865; *Yvridiosuchus*: OUMNH J.1401). This character is in connection with ch. 292, 293 and 294. Foffa et al. (2019) initially described this character, but it was not included in the phylogenetic analysis.

**296.** Premaxilla with no strong lateral expansion (0) or strong lateral expansion so that P3 and P4 are aligned on the lateral plane of the external margin, more so than P2 (1) (Fig. 39).

In most teleosauroids, the P3 and P4 are positioned posteriorly to the P1 and P2 and are aligned on a vertical plane of the lateral margin, whereas the P1 and P2 are aligned more laterally. This is, in part, due to little or no lateral expansion of the premaxillae (state 0). This condition can be clearly seen in *Plagiophthalmosuchus* (NHMUK PV OR 14792), more basal teleosauroids such as *Mystriosaurus* (NHMUK PV OR 14781), *Platysuchus* (MNHNL TU895), *I. potamosiamensis* (PRC-11), *Teleosaurus* (Eudes-Deslongchamps, 1867-69), *Mycterosuchus* (CAMSM J.1420) and *Macrospondylus* (SMNS 18672, SMNS 81699), and in more derived teleosauroids (*Charitomenosuchus*: NHMUK PV R 3806; *Deslongchampsina*: OUMNH J.29851; *Proexochokefalos*: MNHN.F 1890-13; *Neosteneosaurus*: NHMUK PV R 2865; *Yvridiosuchus*: OUMNH J.1401; *Lemmysuchus*: LPP.M.21). In select taxa, the premaxillae are laterally expanded, with the P3 and P4 aligned on a different plane (state 1). This occurs in *Bathysuchus* (DORCM G.05067i; unnumbered LPP specimen) and *Sericodon* (Schaefer, Püntener & Billon-Bruyat, 2018). Foffa et al. (2019) initially highlighted the lateral expansion seen in *Bathysuchus* (NHMUK PV OR 43086), but this character was not included in the phylogenetic analysis.

**297.** Premaxilla, very small first premaxillary alveolus with the second premaxillary alveolus being much larger (0) or the first and second premaxillary alveoli are relatively the same size (1) (Fig. 39).

In most teleosauroids, the size of the P1 and P2 are relatively the same, with both being slightly smaller than the P3 and P4 (which is often the largest, as it houses the large fourth premaxillary tooth) (state 1). This condition is observed in *I. potamosiamensis* (PRC-11), *Mycterosuchus* (CAMSM J.1420), *Bathysuchus* (DORCM G.05067i), *Deslongchampsina* (OUMNH J.29851), *Seldsienean* (MMT P28-1), *Charitomenosuchus* (NHMUK PV R 3806), *Proexochokefalos* (MNHN.F 1890-13), *Neosteneosaurus* (NHMUK PV R 2865), *Yvridiosuchus* (OUMNH J.1401) and *Lemmysuchus* (LPP.M.21). In both *Deslongchampsina* (OUMNH J.29815) and *Yvridiosuchus* (OUMNH J.1401), the P1 is slightly smaller than the P2, but not substantially so.

In certain teleosauroids, the P1 is considerably smaller than the P2, with the P1 being 25% or less the size of the P2 (state 0). This condition is observed in the Chinese teleosauroid (IVPP V 10098) and *Macrospondylus* (SMNS 81699). It is also interesting to note that this feature is also clearly displayed in SNHM-IG-008-R, a taxon referred to as Steneosaurus aff. bollensis (currently thought to be *Mystriosaurus* sp.; MMJ, pers. obs.). However, this specimen is still currently being worked on (by MMJ and colleagues) and is therefore currently not included in the dataset. In addition, the entirety of the P1 and P2 of both verified *Mystriosaurus* laurillardi specimens (HLMD V946-948; NHMUK PV OR 14781: Sachs et al., 2019a) cannot be seen to confirm this. As with ch. 292 to 295, this character is not applicable for taxa with fewer than four premaxillary alveoli (members of the genus *Machimosaurus*).

**339.** Dentition, carinae on the apical third of a tooth are present and well pronounced (0) or absent/weakly pronounced (1) (Fig. 40).

The overall definition, as well as the appearance, of carinae differs with respect to true-ziphodont (all teeth possess serrated or denticulated carinae) or false-ziphodont (noticeable superficial enamel ornamentation contacts the keel) dentition. In general, carinae are defined as sharp, narrow ridges or ‘keels’ running apicobasally along the crown, representing the cutting edge of the tooth. Carinae can be observed in the dentition of theropods (Currie, 1995; Buffetaut et al., 2008; Hendrickx, Mateus & Araújo, 2015), various crocodylomorphs (Prasad & Broin, 2002; Andrade & Bertini, 2008; Andrade et al., 2011), mosasaurs (Lindgren, 2005; Jagt et al., 2005; Grigoriev, 2014) and some ichthyosaurs (Fischer et al., 2011), among other tetrapods. At times, carinae can be difficult to distinguish from enamel ridges (Young & Steel, 2014).

All known teleosauroids possess carinae (excluding the Chinese teleosauroid IVPP V 10098, *Andrianavoay* NHMUK PV R 1999, *Clovesuurdameredeor* NHMUK PV OR 49126 and P. cf. *bouchardi* [Lepage et al., 2008], as none have any teeth preserved); however, the relative noticeability of these ridges differs between taxa. The carinae of *Plagiophthalmosuchus* (MNHNL TU515), *Mystriosaurus* (NHMUK PV OR 14781), *Platysuchus* (SMNS 9930), *Bathysuchus* (DORCM G.05067iv) and *Macrospondylus* (SMNS 51563) are fine and faint, whereas those of *Neosteneosaurus* (PETMG R178) and machimosaurins (*Yvridiosuchus*: OUMNH J.29850; *Lemmysuchus*: NHMUK PV R 3168; *Mac. buffetauti*: SMNS 91415) are well-developed. In addition, most teleosauroids have carinae that extend the entire apicobasal length of the tooth, (state 0). These is seen in the basal form *Plagiophthalmosuchus* (MNHNL TU515) and *Mystriosaurus* (NHMUK PV OR 14781), *I. kalasinensis* (PRC-239), *Mycterosuchus* (NHMUK PV R 2617), *Aeolodon* (MNHN.F.CNJ 78) *Charitomenosuchus* (NHMUK PV R 3806), *Proexochokefalos* (MNHN.F 1890-13) *Seldsienean* (OUMNH J.1414), *Neosteneosaurus* (PETMG R178), *Lemmysuchus* (NHMUK PV R 3168) and *Mac. hugii* (MG8730-1). However, two taxa (*Bathysuchus*: DORCM G.05067iv; *Sericodon*: TCH005-151 in Schaefer, Püntener & Billon-Bruyat, 2018) have carinae that only extend two-thirds the apicobasal length of the tooth, from the base to the apex and are absent at the apex (state 1).

**340.** Dentition, enamel ridges on the apical third of a tooth are absent (0) or present (1) (Fig. 40).

Enamel ridges are elongated, thin, apicobasally oriented crenulations that cover the near entirety of the enamel surface of the tooth. They generally run parallel to one another, flank the carinae on either side and follow the curvature of the tooth.

In teleosauroids, the enamel ridges are either faint and/or difficult to see (e.g. *Plagiophthalmosuchus*: MNHNL TU515), or noticeable and well-developed (e.g. *Mycterosuchus*: NHMUK PV R 2617). In most teleosauroids (including the basal taxon *Plagiophthalmosuchus*), on the basal 4/5ths of the tooth these ridges are generally numerous, continuous from the base and aligned parallel with one another. In machimosaurins (*Yvridiosuchus*: OUMNH J.29851; *Lemmysuchus*: NHMUK PV R 3168; *Mac. buffetauti*: SMNS 91415; *Mac. hugii* MG-8730-1; *Mac. rex*: ONM NG 7), at the apical fifth these ridges abruptly change, becoming shorter and randomly spaced in an anastomosed pattern (see ch. 358 below). Enamel ridges are present on the entirety of the crown, including the apex (state 1) in the basal-most form *Plagiophthalmosuchus* (MNHNL TU515), along with most teleosauroids (*Mystriosaurus*: NHMUK PV OR 14781; *I. kalasinensis*: PRC-239; *Mycterosuchus*: NHMUK PV R 2617; *Bathysuchus*: DORCM G.05067iv; *Aeolodon*: MNHN.F.CNJ 78; *Macrospondylus*: SMNS 53422; *Charitomenosuchus*: NHMUK PV R 3806; *Seldsienean*: OUMNH J.1414; *Deslongchampsina*: OUMNH J.29851; *Neosteneosaurus*: PETMG R178; machimosaurins: OUMNH J.29850, NHMUK PV R 3168; NHMW 1846.III.208). In *Proexochokefalos* (MNHN.F 1890-13), the teeth appear to have enamel ridges that reach the apices, but many are covered in a thin coating of adhesive substance, so it is difficult to tell if they are either poorly or well developed; therefore, this taxon has been scored as {01}. Only in one confirmed taxon, *Sericodon* (TCH005-151 in Schaefer, Püntener & Billon-Bruyat, 2018), are the enamel ridges absent from the apex (state 0). However, it is interesting to note that an unnumbered MNHN ‘Steneosaurus’ sp. and unnumbered NHMW *Teleosaurus* also display state 0.

**394.** Cervical ribs in lateral view, the anteroposterior ridge of large, more posteriorly placed cervical ribs is straight (0) or dorsoventrally curved (1) (Fig. 41).

Most teleosauroids that can be scored for this character exhibit T-shaped (in dorsal view) cervical ribs where the anteroposterior ridge is horizontal or straightened (state 0), which is observed in all the ribs, including the larger, posterior ones. Taxa with state 0 include *Platysuchus* (SMNS 9930), *Mycterosuchus* (NHMUK PV R 2617), *Charitomenosuchus* (NHMUK PV R 3806) and *Neosteneosaurus* (PETMG R178). However, in *Lemmysuchus* (NHMUK PV R 3168), the largest, most posteriorly placed cervical ribs have a distinct dorsomedial curvature along the anteroposterior ridge, appearing slightly concave in lateral view (state 1). It is unclear if this is a synapomorphy for Machimosaurini: in *Mac. mosae* (Hua, 1999), the cervical ribs are partially preserved, and it is not clear if these are the more anterior or posteriorly placed ones (the larger posterior cervical ribs on the IRSNB *Mac. mosae* cast appear to be curved). Seven cervical ribs are preserved in *Mac. buffetauti*, with the largest one being anteroposteriorly straight (more similar to *Neosteneosaurus*: PETMG R178; *Charitomenosuchus*: NHMUK PV R 3806; *Platysuchus*: SMNS 9930). Note that this character was initially described and figured in Johnson et al. (2017).

**395.** Dorsal ribs, the positioning of both the tuberculum and articular facet is on the medial edge (0), directly in the middle (1), or on the lateromedial edge (2) (Fig. 42).

The tuberculum, located on the posterior surface of the dorsal (thoracic) rib, is a relatively small protrusion that articulates with the opposite facet located on the transverse process of the same numbered vertebra. In contrast, the flattened articular facets are separated from the top of the tuberculum; these bony knobs are divided into superior (top) and inferior (bottom) surfaces, which are placed close together and articulate with the adjoining facet on the vertebral processes.

In most teleosauroids with preserved dorsal ribs, both the tuberculum and articular facet are positioned on the medial edge of the rib (state 0). This is observed in *Platysuchus* (SMNS 9930), *Macrospondylus* (SMNS 51753, SMNS 18672), *Aeolodon* (MNHN.F.CNJ 78) and *Lemmysuchus* (NHMUK PV R 3168). In two taxa (*Mycterosuchus*: NHMUK PV R 2617; *Charitomenosuchus*: NHMUK PV R 3806), the tuberculum and articular facets have shifted laterally and are placed directly in the middle of the rib (state 1). In *Neosteneosaurus* (NHMUK PV R 3701, PETMG R178), the tuberculum and articular facets have shifted even further laterally so that they are positioned on the lateromedial edge of the rib (state 2). In *Mac. buffetauti* (SMNS 91415), there are approximately twelve dorsal ribs present, but few are complete with a relatively well preserved rib head (which includes the capitulum, articular facets and tuberculum). In addition, it is difficult to confidently locate where these surfaces are positioned in the dorsal rib of *Sericodon* (SCR010-312 in Schaefer, Püntener & Billon-Bruyat, 2018), but they appear to be either medially or lateromedially placed.

**396.** Dorsal ribs in lateral view, the tuberculum is pronounced (0) or weak (1) (Fig. 42).

As mentioned in ch. 395, the tuberculum is a relatively reduced protrusion on the proximal half of the dorsal (thoracic) rib. However, the size of the tuberculum changes drastically in teleosauroids, particularly in the largest dorsal ribs. In *Mycterosuchus* (NHMUK PV R 2617), *Neosteneosaurus* (PETMG R178), *Lemmysuchus* (NHMUK PV R 3168) and *Mac. buffetauti* (SMNS 91415), the tuberculum is well-developed and pronounced, as large as the capitulum and anteroposteriorly elongated, giving it an oval shape (state 0). In certain taxa (*Sericodon*: Schaefer, Püntener & Billon-Bruyat, 2018; *Aeolodon*: MNHN.F.CNJ 78; *Macrospondylus*: SMNS 51753; *Charitomenosuchus*: NHMUK PV R 3806), the tuberculum is reduced, small and circular in shape (state 1). In this state, it is much smaller than the capitulum; this is well exemplified in *Charitomenosuchus* (NHMUK PV R 3806). In *Platysuchus* (SMNS 9930), the tubercula of the anterior dorsal ribs are pronounced; however, in the middle to posterior ribs most of the rib heads are covered by osteoderms, although the tuberculum appears to be relatively reduced compared to the anterior ones. Due to this uncertainty, *Platysuchus* was scored as {01}. Johnson et al. (2017) first noted and figured this feature, specifically focusing on the differences between *Lemmysuchus* (NHMUK PV R 3168), *Charitomenosuchus* (NHMUK PV R 3806) and *Neosteneosaurus* (NHMUK PV R 3701).

**398.** Second sacral vertebrae, the anterior margin of the posterior area of the second sacral vertebra has either a small, non-expanding flange (0) or a large, expanded and projecting flange (1) (Fig. 43).

In crocodylomorphs, the posterior area of the second sacral vertebra has an anterior margin that is both anteroposteriorly and dorsoventrally expanded into a projection or ‘flange’ of bone, which allows for a secure attachment to the ilium, thus influencing body movement. This ‘flange’ is either small and non-expanding (state 0), or noticeably expanded and anteroposteriorly protruding (state 1). All scored teleosauroids exhibit state 1, as there is always an expanded flange present on the anterior margin; however, the size and development differ. In the taxa *Mycterosuchus* (NHMUK PV R 2617), *Charitomenosuchus* (NHMUK PV R 3806), *Lemmysuchus* (NHMUK PV R 3168) and *Mac. mosae* (Hua, 1999; Young et al., 2014), the flange is considerably larger, more pronounced and well-developed. In *Macrospondylus* (MMG BwJ 595) and *Neosteneosaurus* (NHMUK PV R 3701) the flange is still present, but it is much smaller and less obvious. This character was initially described and figured in Johnson et al. (2017).

**417.** Radius and ulna, the same length (0) or the ulna is longer (1) (Fig. 44).

The radius and ulna are two bones of the forelimb; they articulate proximally with the humerus and distally with the carpal (wrist) bones. In teleosauroids, the radius is a straight, squat, unornamented bone with a slightly expanded head that is positioned laterally; in contrast, the ulna is noticeably proximally expanded with a flattened, dorsoventrally elongated olecranon process, and is positioned medially. The radius and ulna articulate with one another; the radial head has a small facet for articulation with the ulna. In the majority of teleosauroids, the radius and ulna are approximately the same size (Andrews, 1913), with the ulna being marginally longer; this is seen in taxa such as *Platysuchus* (SMNS 9930), *Aeolodon* (MNHN.F.CNJ 78), *Macrospondylus* (SMNS 51563, SMNS 53422), *Charitomenosuchus* (NHMUK PV R 3608), *Neosteneosaurus* (PETMG R178) and *Lemmysuchus* (NHMUK PV R 3168). However, in the genus *Mycterosuchus* (NHMUK PV R 2617) the ulna is roughly 18% longer than the radius, which is unusual. While Andrews (1913) did note the large radius and ulna of *Mycterosuchus* relative to other ‘Steneosaurus’ species, the relation between the two bones was not included in his observations.

**430.** Pubis, the shape of distal rim of distal pubic blade is straight and square-like (0) or curved and rounded (1) (Fig. 45).

The pubis is one of the three bones of the hip, and is divided into three distinct parts: (i) the pubic head, a rounded proximal knob of bone that articulates with the ischium and ilium; (ii) the pubic shaft, the middle of the bone; and (iii) the pubic plate (=blade), a distally directed, flattened expansion of bone. This character concerns the distal rim of the pubic blade, considering its shape and appearance. In most scored teleosauroids, the ventral (distal) margin of the pubic blade is anteriorly curved and rounded in lateral view (state 1). This is the case in *Charitomenosuchus* (NHMUK PV R 3806), *Macrospondylus* (SMNS 51957), *Neosteneosaurus* (PETMG R178), *Lemmysuchus* (NHMUK PV R 3168) and *Mac. mosae* (Hua, 1999; Young et al., 2014). However, in two taxa the distal rim of the pubic blade is straightened and relatively square-like (state 0): *Mycterosuchus* (NHMUK PV R 2617) and *Platysuchus* (SMNS 9930).

**431.** Pubis, the pubic shaft is shorter (0) or longer (1) than the pubic blade (Fig. 45).

The pubis is relatively anteroposteriorly elongated in teleosauroids, and both the pubic shaft and pubic blade represent a significant part of this elongation. In most taxa, the pubic shaft is either approximately the same length or slightly anteroposteriorly shorter than the pubic blade (state 0). This is the condition seen in six scored teleosauroids: *Macrospondylus* (SMNS 51957), *Charitomenosuchus* (NHMUK PV R 3806), *Lemmysuchus* (NHMUK PV R 3168), *Mac. mosae* (Hua, 1999), *Platysuchus* (SMNS 9930) and *Sericodon* (SCR010-312 in Schaefer, Püntener & Billon-Bruyat, 2018). However, the pubic shaft is significantly longer (over 50%) than the pubic blade (state 1) in one taxon (*Mycterosuchus*: NHMUK PV R 2617) and represents an apomorphic trait of this genus. A longer, lankier pubis may also have an effect on pelvic girdle aspiration; in modern crocodylians, the pubic bones are highly mobile and can be rotated using pelvic and hypaxial muscles as an ‘aspiration pump’ used in breathing (Rathke, 1866; Farmer & Carrier, 2000; Claessons, 2004).

**434.** Ilium, the anterior iliac process is long and slender (0), or short and robust (1) (Fig. 46).

The anterior, or preacetabular, process is a projection of bone situated on the anterodorsal rim of the ilium. In most teleosauroids, this process is anteroposteriorly elongated, mediolaterally slender, and straight with little to no curvature (state 0). This is seen in *Platysuchus* (SMNS 9930), *Teleosaurus* (NHMUK PV R 1782a), *Sericodon* (SCR010-312 in Schaefer, Püntener & Billon-Bruyat, 2018), *Aeolodon* (MNHN.F.CNJ 78), *Macrospondylus* (MMG BwJ 565), *Charitomenosuchus* (NHMUK PV R 3806; Andrews, 1913) and *Neosteneosaurus* (PETMG R178). In contrast, state 1 describes the anterior process as anteroposteriorly shortened, robust and chunky in appearance, with a slight lateral curvature. This morphology is present in the machimosaurins *Lemmysuchus* (NHMUK PV R 3168) and *Mac. mosae* (Hua, 1999; Young et al., 2014), as well as the basal metriorhynchoid *Pelagosaurus* (MNHN.RJN 463) and members of Metriorhynchidae (e.g. Tyrannoneustes lythrodectikos Young et al., 2013; Cricosaurus lithographicus; Cricosaurus araucanensis [Herrera, Fernández & Gasparini, 2013]; Fraas, 1902; Andrews, 1913). The shortening and general robustness of the ilium in machimosaurins may be due to living in a higher energy environment, or indicative of more terrestrial movement. Johnson et al. (2017) described and figured this character specifically in machimosaurins.

**438.** Supraacetabular iliac crest is pronounced (0) or shallow and poorly developed (1) in medial view (Fig. 46).

The supraacetabular iliac crest, observed in medial view, is a projection of bone that runs along the anterior margin of the acetabulum. In non-machimosaurins (*Plagiophthalmosuchus*: NHMUK PV OR 14792; *Platysuchus*: SMNS 9930; *Teleosaurus*: NHMUK PV R 1782a; *Sericodon*: SCR010-312 in Schaefer, Püntener & Billon-Bruyat, 2018; *Macrospondylus*: SMNS 51984; *Charitomenosuchus*: NHMUK PV R 3806; *Neosteneosaurus*: NHMUK PV R 3701, PETMG R178) the supraacetabular crest is enlarged and pronounced, jutting out laterally and slightly overhanging the acetabulum (state 0). In state 1, the supraacetabular crest is poorly developed, with either shallow or no outward projection. This is the case in the machimosaurins *Lemmysuchus* (NHMUK PV R 3168; Johnson et al., 2017) and *Mac. mosae* (Hua, 1999). In addition, the supraacetabular crest in state 1 is anteroposteriorly short and its posterior extension along the anterior margin of the acetabulum varies between taxa. For example, in *Lemmysuchus* (NHMUK PV R 3168), the supraacetabular crest is greatly reduced with very little posterior extension; it is difficult to even distinguish from the acetabulum (although this may be due to poor preservation).

**449.** Ischium, the posteroventral margin of ischial blade is triangular (0) or sub-square (1) (Fig. 47).

The ischial blade (=plate) is an expanded, mediolaterally flattened sheet of bone that makes up the distal area of the ischium. In most teleosauroids, the ischial blade is gracile, mediolaterally thin and anteroposteriorly elongated, with the posteroventral margin having a triangular-like shape (state 0). This morphology is present in *Platysuchus* (SMNS 9930), *Teleosaurus* (NHMUK PV R 1638), *Mycterosuchus* (CAMSM J.1420), *Macrospondylus* (SMNS 51957), *Charitomenosuchus* (NHMUK PV R 3806) and *Neosteneosaurus* (NHMUK PV R 3701, PETMG R178). A second condition (state 1) is that the posteroventral margin is noticeably anteroposteriorly shortened and dorsoventrally broad, giving it a sub-square shape. This state is unique to machimosaurins (*Lemmysuchus*: NHMUK PV R 3168; *Mac. mosae*: ISRNB cast; Hua, 1999; Young et al., 2014). Wilkinson, Young & Benton (2008) first took note of this feature in a metriorhynchid ilium (Torvoneustes carpenteri), and Johnson et al. (2017) described and figured it in relation to teleosauroids (specifically *Lemmysuchus* and closely related taxa).

When examining all bones of the pelvis, there appear to be two distinct morphotypes in teleosauroids: the slender, more gracile morphotype (e.g. *Charitomenosuchus*) and the stocky, robust morphotype (e.g. *Lemmysuchus*). The general shortening and robustness of the pelvis in certain teleosauroids may be due to living in a higher energy or more terrestrial environments, as mentioned previously (Johnson et al., 2017). In addition, a stockier pelvis may also have an effect on pelvic girdle aspiration: in modern alligators, the pubic bones are rotated using pelvic and hypaxial muscles, which increase lung ventilation (Claessens, 2004). A decrease in surface area for pelvic muscle attachment may indicate less reliance on pelvic girdle aspiration, and more so on diaphragmatic and costosternal breathing.

**456.** Femur in dorsal view, the anteromedial tuber is present and small (0), or the largest of the proximal tubera (1) (Fig. 48).

The femora of teleosauroids have historically been regarded as relatively similar throughout the entirety of the group (Andrews, 1909, 1913). However, there are some subtle yet key differences in femoral anatomy between genera. One of these characters focusses on the anteromedial tuber of the proximal femoral head, which articulates with the acetabulum of the ilium. There are three tubera present on the head of the femur: the anteromedial, posteromedial and anterolateral tubera. The presence of a proximal anteromedial tuber is a characteristic synapomorphy for all Archosauria (Nesbitt, 2011), and the posteromedial tuber is generally the largest of the three.

In most teleosauroids, the posteromedial tuber remains the largest, and the anteromedial tuber is present but relatively small (state 0). This is the condition seen in *Platysuchus* (SMNS 9930), *Sericodon* (SCR010-312 in Schaefer, Püntener & Billon-Bruyat, 2018), *Aeolodon* (MNHN.F.CNJ 78), *Macrospondylus* (SMNS 18672), *Charitomenosuchus* (NHMUK PV R 3806), *Neosteneosaurus* (PETMG R178) and machimosaurins (*Lemmysuchus*: NHMUK PV R 3168; *Machimosaurus*: Hua, 1999) (although it is interesting to note that in *Sericodon*, the anterolateral tuber is well-developed but is still not nearly as pronounced as the posteromedial tuber). The genus *Mycterosuchus* (NHMUK PV R 2617), however, has an anteromedial tuber that is noticeably well pronounced and well-developed, and it is the largest of all proximal tubera (state 1).

**459.** Femur, the distal medial and lateral condyles are the same size (0), or the medial condyle is larger than the lateral condyle (1) (Fig. 48).

Another femoral feature that can differ between teleosauroids is the size of the lateral and medial condyles of the distal end. These condyles are anteroposteriorly elongated and rounded at the margins. They are separated by an intermediate groove, and the lateral condyle articulates with the proximal tibia whereas the medial condyle articulates with both the proximal tibia and fibula. The flexor digitorum longus (via tendon) and flexor hallucis longus (via fleshy attachment) muscles both originate at the lateral condyle and are responsible for extension of the knee and flexing the digits (Klinkhamer et al., 2017).

In most teleosauroids, the medial and lateral condyles of the femur are approximately the same size (state 0). This condition is seen in the basal form *Plagiophthalmosuchus* (NHMUK PV OR 14792), as well as *Platysuchus* (SMNS 9930), *Aeolodon* (MNHN.F.CNJ 78), *Macrospondylus* (SMNS 51555) and *Lemmysuchus* (NHMUK PV R 3168). In certain teleosauroid genera, however, the femoral medial condyle is noticeably larger than the femoral lateral condyle (state 1). This is the case in *Mycterosuchus* (NHMUK PV R 2617) and *Neosteneosaurus* (NHMUK PV R 3701, PETMG R178). Note that dorsoventral crushing can distort the femoral condyles, making it appear as though the medial condyle is larger than the lateral condyle, such as in certain *Macrospondylus* specimens. Johnson et al. (2017) initially described and figured this character.

**464.** Tibia in lateral view, the angle of tibial tuberosity is horizontal (0) or ventral (1) (Fig. 49).

The tibial tuberosity is an oblong, prominent shelf of bone on the proximal anterior area of the tibia, and is an insertion point for the tendon of the quadriceps femoris. In most scored teleosauroids, the tibial tuberosity is horizontally placed in lateral view (state 0). This is seen in the basal form *Plagiophthalmosuchus* (NHMUK PV OR 14792) as well as *Platysuchus* (SMNS 9930), *Mycterosuchus* (NHMUK PV R 2617), *Aeolodon* (MNHN.F.CNJ 78), *Macrospondylus* (SMNS 51984), *Charitomenosuchus* (NHMUK PV R 3806) and *Neosteneosaurus* (NHMUK PV R 3701, PETMG R178). In select teleosauroids, the angle of the tibial tuberosity is strongly ventrally displaced. This condition (state 1) is seen in machimosaurins (*Lemmysuchus*: NHMUK PV R 3168; *Machimosaurus*: IRSNB cast; Hua, 1999).

**466.** Calcaneum, the calcaneum tuber is the same size (0) or larger (1) than the astragalus (Fig. 50).

The calcaneum is the largest of the tarsal bones, forming the heel of the foot. It attaches to the distal tarsals and has a strong convex surface for articulation with the fibula (Sereno & Arcucci, 1990). In contrast, the astragalus is a roughly circular anklebone that is tightly bound to the distal ends of the tibia and fibula (Sereno & Arcucci, 1990). It is the insertion point for the fibularis longus muscle, which flexes the ankle (Klinkhamer et al., 2017). Both the calcaneum and astragalus are approximately the same shapes in all scored teleosauroids; both tarsal bones are also relatively the same size as one another (state 0), with the calcaneum being marginally larger. This condition is observed in *Platysuchus* (SMNS 9930), *Macrospondylus* (MMG BwJ 565, SMNS 51984), *Charitomenosuchus* (NHMUK PV R 3806), *Neosteneosaurus* (PETMG R178) and *Lemmysuchus* (NHMUK PV R 3168). However, in *Mycterosuchus* (NHMUK PV R 2617) the enlarged calcaneum tuber is noticeably larger than the astragalus (state 1), by approximately 25%. This condition is currently autapomorphic for this genus.

**489.** Sacral dorsal armour (osteoderms), the dorsal keel is elongated and shallow (0) or elongated and pronounced (1) (Fig. 51).

In teleosauroids, the sacral dorsal osteoderms are often the largest, being elongated and oval-shaped. In addition, there is often a longitudinal ridge (or keel) running anteroposteriorly across the near-entirety of these osteoderms, terminating at an anterior peg-like structure. In certain teleosauroids, this keel is anteroposteriorly elongated but shallow (state 0). This condition is seen in *Plagiophthalmosuchus* (NHMUK PV OR 14792), *Platysuchus* (SMNS 9930), *Teleosaurus* (NHMUK PV R 4207, NHMUK PV OR 32584), *Aeolodon* (NHMUK PV R 1086, MNHN.F.CNJ 78), *Macrospondylus* (SMNS 51563) and *Charitomenosuchus* (NHMUK PV R 3806). In more derived teleosauroids, the keel of the sacral osteoderms is elongated, well-developed and pronounced (state 1), and is often considerably more thickened than in state 0. State 1 is well exemplified in large specimens of *Neosteneosaurus* (PETMG R178) as well as the machimosaurin *Lemmysuchus* (NHMUK PV R 3168). It is interesting to note that in *Mac. buffetauti* (SMNS 91415), the preserved osteoderms appear to have a shallow keel, more similar to *Platysuchus* (SMNS 9930) or *Teleosaurus* (NHMUK PV R 4207); however, it is unclear whether these osteoderms are part of the sacral dorsal shield, as they are associated with dorsal vertebrae. Therefore, *Mac. buffetauti* is currently scored as (?). This is also the case with *Mac. rex* (Fanti et al., 2016). Hua (1999) mentioned a thickened, distinct keel on the ‘lumbar’ osteoderms of *Mac. mosae*; the accompanying photographs are too dark to confidently determine this, though; therefore, *Mac. mosae* is also currently scored as (?).

**S2) Previous characters pertaining to teleosauroids**

In addition to the 38 new characters described above, several original characters from the 2016 H+Y dataset are key in differentiating between various teleosauroid taxa. In particular, 19 characters are anatomically distinct, variant and important in teleosauroids and are described in detail as follows:

**10.** Rostrum narrows markedly in dorsal view immediately in front of the orbits (0), or there is no narrowing (1) (Fig. 52).

This character is linked with character 151 (see below) and focuses on the morphology of telescopic orbits. In most teleosauroids, the posterior portion of the rostrum will either narrow slightly mediolaterally or not narrow at all, instead becoming flush with the anterior rim of the orbit (state 1). This is seen in *Plagiophthalmosuchus* (NHMUK PV OR 14792), *Mystriosaurus* (NHMUK PV OR 14781), the Chinese teleosauroid (IVPP V 10098), *Platysuchus* (SMNS 9930), and a particular subclade of teleosauroids (e.g. *Macrospondylus* MMG BwJ 565; *Charitomenosuchus*: NHMUK PV R 3806; *Clovesuurdameredeor*: NHMUK PV OR 49126; *Proexochokefalos*: MNHN.F 1890-13; *Neosteneosaurus*: PETMG R178; *Yvridiosuchus* OUMNH J.1401, *Mac. buffetauti* SMNS 91415). In certain teleosauroids, however, there is a distinct and pronounced narrowing, or mediolateral compression, of the rostrum immediately anterior to the orbits, causing the dorsal margins of the orbits to become upturned (state 0). This condition is in *Mycterosuchus* (NHMUK PV R 2617), *Aeolodon* (MNHN.F.CNJ 78), *I. potamosiamensis* (PRC-11), *Teleosaurus* (MNHN AC 8746), *Sericodon* (Schaefer, Püntener & Billon-Bruyat, 2018), *Bathysuchus* (unnumbered LPP specimen) and *Seldsienean* (MMT P28-1).

The skull of *I. kalasinensis* (PRC-239) has been both dorsoventrally flattened and slightly anteriorly distorted. However, while there is a noticeable narrowing of the rostrum, this begins further anteriorly than in taxa with state 1 (*Mycterosuchus*; *Aeolodon*; *I. potamosiamensis*; *Teleosaurus*; *Sericodon*; *Bathysuchus*; *Seldsienean*), and there is no immediate narrowing anterior to the orbital margin (contrary to Martin et al. [2019]). The rostrum appears to be flush with the rim of the anterior orbital margin (best seen on the left side). Therefore, this taxon was scored as state 0. Young et al. (2016) first described this character; it has subsequently been used in the datasets of Ristevski et al. (2018), Ősi et al. (2018), Foffa et al. (2019), Johnson et al. (2019) and Sachs et al. (2019a, 2019b).

**27.** Neurovascular foramina of the premaxillae/maxillae, represented by a single line of small sub-circular openings (0), or two lines (one dorsal, one ventral) of large, circular openings (1) (Fig. 53).

On the lateral premaxillae and maxillae, teleosauroids possess numerous neurovascular foramina. These openings are possibly involved with multiple mechanoreceptory function such as prey detection, tactile discrimination or disruption in the surrounding water (e.g. Soares, 2002; Leitch & Catania, 2012). In most teleosauroids, the neurovascular foramina are small and subcircular in shape on both the premaxilla and maxilla and are generally consistent in size and number. On the premaxilla, these foramina are restricted to the anteroventral and lateroventral margins of the external nares. On the ventrolateral surface of the maxilla, dorsal to the tooth row, they form a single line and are relatively well spaced. This condition (state 0) is seen in taxa such as the basal-most teleosauroid *Plagiophthalmosuchus* (NHMUK PV OR 14792) and *Platysuchus* (SMNS 9930), *Mycterosuchus* (NHMUK PV R 2617), *Macrospondylus* (PMU R161), and *Neosteneosaurus* (NHMUK PV 2865). *Deslongchampsina* (OUMNH J. 29851) also has restricted foramina on the premaxilla as well as a single line on the maxilla; however, the foramina are larger than those seen in other taxa with state 0, and are slightly anteroposteriorly elongated on the maxilla (most notably at the anterior and middle areas of the rostrum).

State 1 is seen in the genus *Mystriosaurus* (NHMUK PV R 14781) along with members of Machimosaurini (*Yvridiosuchus*: OUMNH J.1401, OUMNH J.29850; *Lemmysuchus*: NHMUK PV R 3168; *Mac. buffetauti*: SMNS 91415; *Mac. mosae*: Young et al., 2014): these taxa display large, deep, numerous, sub-circular neurovascular foramina (although the foramina in *Mystriosaurus* are smaller than in machimosaurins). The premaxillary openings are generally circular in shape, located around the ventral, lateral and anteroventral margins of the external nares and cluster together (especially around the external nares’ lateral margins). On the maxilla, the foramina are more anteroposteriorly elongated and situated in two parallel lines, one dorsal to the tooth row with an additional line above it (state 1). The foramina are closely spaced together at the anterior part of the maxilla, but they gradually become more distanced from one another further posteriorly. In addition, it is interesting to note that the premaxillary foramina are exceptionally large in *Yvridiosuchus* (OUMNH J.29850) as well as only around the anteroventral margin of the external nares in *I. kalasinensis* (PRC-239).

Andrade et al. (2011) initially described this character with respect to neurovascular foramina in the taxon Goniopholis kiplingi (DORCM 12154). It has been subsequently updated by Ristevski et al. (2018), Ősi et al. (2018), Foffa et al. (2019), Johnson et al. (2019) and Sachs et al. (2019a, 2019b). We re-wrote this character to merge the absence of neurovascular foramina or presence of them as a single line into one state (state 0). This was in response to observing two parallel lines in machimosaurins (*Yvridiosuchus*: OUMNH J.1401, OUMNH J.29850; *Lemmysuchus*: LPP.M.21; *Machimosaurus*: SMNS 91415). In addition, this character may need additional re-definition, as George & Holliday (2013) recently questioned the use of facial neurovascular foramina as osteological correlates.

**34.** External nares oriented anteriorly or anterodorsally (0), or dorsally (1) (Fig. 54).

The orientation of the external nares is often used to categorize teleosauroids. In a certain group of predominately Laurasian teleosauroids, the external nares face either anteriorly or anterodorsally (state 0). This condition occurs in *Mystriosaurus* (NHMUK PV OR 14781), the Chinese teleosauroid (IVPP V 1009), *Mycterosuchus* (NHMUK PV R 2617), *Teleosaurus* (Eudes-Deslongchamps, 1867-69), *Platysuchus* (SMNS 9930), *Aeolodon* (MNHN.F.CNJ 78), *Sericodon* (SCR011-406 in Schaefer, Püntener & Billon-Bruyat, 2018) and *Bathysuchus* (unnumbered LPP specimen). In *Mystriosaurus* (HLMD V946-948, NHMUK PV OR 14781), the external nares are oriented fully anteriorly, while in the other taxa they are oriented anterodorsally. In predominately Sub-Boreal/Gondwanan teleosauroids, the external nares are oriented dorsally (state 1). This is seen in *Macrospondylus* (PMU R161), *Charitomenosuchus* (NHMUK PV R 3806), *Deslongchampsina* (OUMNH J.29851), *Proexochokefalos* (MNHN.F 1890-13), *Neosteneosaurus* (NHMUK PV R 2865) and machimosaurins (*Yvridiosuchus*: OUMNH J.1401; *Lemmysuchus*: LPP.M.21; *Machimosaurus*: SMNS 91415). Turner & Pritchard (2015) modified this character from Clark (1994). It has been included in the datasets of Ristevski et al. (2018), Ősi et al. (2018), Foffa et al. (2019), Johnson et al. (2019) and Sachs et al. (2019a, 2019b).

**48.** Premaxilla in lateral view, the anterior and anterolateral premaxillary margins are not sub-vertical, or do not extend ventrally (0), or the anterior and anterolateral margins are orientated anteroventrally and extend ventrally (1) (Fig. 53).

This character is important, as the states distinguish the two teleosauroid families. In one subclade, the anterior and anterolateral margins of the premaxilla are not sub-vertical and do not extend ventrally (state 0) when compared to the rest of the premaxilla; rather, they are anterodorsally curved in a continuous arc throughout. This condition is seen in the basal teleosauroid *Plagiophthalmosuchus* (NHMUK PV OR 14792) as well as *Macrospondylus* (PMU R161), *Seldsienean* (MMT P28-1), *Charitomenosuchus* (NHMUK PV R 3806), *Deslongchampsina* (OUMNH J.29851), *Proexochokefalos* (MNHN.F 1890-13), *Andrianavoay* (NHMUK PV R 1999), *Neosteneosaurus* (NHMUK PV R 2865) and Machimosaurini (*Yvridiosuchus*: OUMNH J.29850; *Lemmysuchus*: NHMUK PV R 3168; members of *Machimosaurus*: SMNS 91415, IRSNB cast, Young et al. [2014]). In the second subclade, the anterior and anterolateral premaxillary margins are strongly oriented anteroventrally and extend ventrally in lateral view, giving these margins a near-vertical appearance. This condition (state 1) occurs in *Mystriosaurus* (NHMUK PV OR 14781), the Chinese teleosauroid (IVPP V 10098), *Platysuchus* (SMNS 9930), *Mycterosuchus* (NHMUK PV R 2617), *I. potamosiamensis* (PRC-11), *Bathysuchus* (unnumbered LPP specimen) and *Aeolodon* (MNHN.F.CNJ 78). It is particularly well-developed in *Mystriosaurus* (NHMUK PV OR 14781) and the Chinese teleosauroid (IVPP V 10098).

Ristevski et al. (2018) first defined and included this character in their accompanying H+Y dataset. It has since been included by Ősi et al. (2018), Foffa et al. (2019), Johnson et al. (2019) and Sachs et al. (2019a, 2019b). However, in our current dataset, this character has been heavily modified to focus on the teleosauroid anteroventral extension of the premaxilla, which is not homologous with the pholidosaurid ventral verticalisation of the premaxilla. Therefore, it is now scored as inapplicable for pholidosaurids and goniopholidids.

**83.** Antorbital fenestrae/cavity, absent (0) or present (1) (Fig. 52).

The external antorbital fenestra is an opening situated anterior to the orbit and is visible in both dorsal and lateral views. It is surrounded by the maxilla and lacrimal bones, with the maxilla contributing to the ventral margin, and the lacrimal contributing to the dorsal margin. This structure is a synapomorphy of Archosauriformes (Witmer, 1997; Leardi, Pol & Fernández, 2012), and hosts a pneumatic sinus. Modern crocodylians have lost this feature, internalizing the antorbital sinus, and in thalattosuchians, particularly metriorhynchids, the antorbital fenestra has often been associated with an exocrine gland (e.g. Leardi, Pol & Fernández, 2012 and references therein). In Teleosauroidea, this opening has been previously interpreted as homologous to the antorbital fenestra of other archosaurs (Leardi, Pol & Fernández, 2012). Generally, teleosauroids, therefore, have very little development of the antorbital fossa (Witmer, 1997).

In most teleosauroids, a small, slit-like or subcircular antorbital fenestra is present (state 1). This condition is seen in taxa such as *Mycterosuchus* (NHMUK PV R 2617), *Indosinosuchus* (PRC-11, PRC-239), *Teleosaurus* (MNHN AC 8746), *Charitomenosuchus* (NHMUK PV R 3806), *Macrospondylus* (MMG BwJ 565) and *Yvridiosuchus* (OUMNH J.1401). The antorbital fenestrae in *Macrospondylus* (MMG BwJ 565), *Teleosaurus* (MNHN AC 8746) and *Charitomenosuchus* (NHMUK PV R 3320) are particularly shallow. In *Plagiophthalmosuchus* (NHMUK PV OR 14792) and *Deslongchampsina* (OUMNH J.29851), the antorbital fenestrae are large and elongated (see ch. 86). However, in *Proexochokefalos* (MNHN.F 1890-13), *Neosteneosaurus* (PETMG R178) and select members of Machimosaurini (*Lemmysuchus*: LPP.M.21; *Machimosaurus*: SMNS 91415; Young et al., 2014) the antorbital fenestrae (and internal antorbital fossae) are absent (state 0). In *Clovesuurdameredeor* (NHMUK PV OR 49126), it is unclear whether the antorbital fenestrae are absent or present, due to poor preservation. However, there appears to be evidence of a smooth ventral margin with a small accompanying depression in the area where the antorbital fenestrae should be located (similar to that seen in *Charitomenosuchus*); therefore, this taxon was scored as state 1.

This character has been subsequently modified from Clark (1994) and Andrade et al. (2011), and was initially combined with an additional character in Young & Andrade (2009), Young et al. (2011), Young et al. (2012), Young et al. (2013), Young (2014), Young et al. (2016) and Ristevski et al. (2018). It is included in its current form in the H+Y datasets from Ősi et al. (2018), Foffa et al. (2019), Johnson et al. (2019) and Sachs et al. (2019a, 2019b).

**86.** Antorbital fenestrae/cavity sub-circular (0) or anteroposteriorly elongated (1) in shape (Fig. 52).

As mentioned in the previous character, most teleosauroids possess small antorbital fenestrae. In addition to the presence or absence of the fenestrae, their size and shape are also distinguishing features within teleosauroids. In most taxa, the openings are subcircular or sub-oval in shape (state 0). This condition is seen in *Mystriosaurus* (NHMUK PV OR 14781), the Chinese teleosauroid (IVPP V 10098), *Indosinosuchus* (PRC-11; PRC-239), *Platysuchus* (SMNS 9930), *Teleosaurus* (MNHN AC 8746), *Mycterosuchus* (NHMUK PV R 2617), *Macrospondylus* (SMNS 51555), *Charitomenosuchus* (NHMUK PV R 3320) and *Yvridiosuchus* (OUMNH J.1401). Most notably, in *Plagiophthalmosuchus* (NHMUK PV OR 14792) and *Deslongchampsina* (OUMNH J.29851: Johnson, Young & Brusatte, 2019), the antorbital fenestrae are large and anteroposteriorly elongated (state 1), making them appear fully oval- or teardrop-shaped. In *Deslongchampsina* (OUMNH J.29851), the antorbital fenestra is approximately 24-25% of the anteroposterior orbital length and 25% of the mediolateral orbital width; in *Plagiophthalmosuchus* (NHMUK PV OR 14792) it is approximately 57% anteroposterior orbital length and 25% of the mediolateral orbital width. The antorbital fenestra (best seen on the right side) of *Mystriosaurus* (NHMUK PV OR 14781) is also relatively large (approximately 27% of the anteroposterior orbital length), but is not considerably elongated nor oval-shaped as in either *Plagiophthalmosuchus* or *Deslongchampsina*, and is therefore scored as state 0. Note that this character is not applicable for those taxa that lack antorbital fenestrae: *Proexochokefalos* (MNHN.F 1890-13), *Neosteneosaurus* (PETMG R178), *Lemmysuchus* (LPP.M.21) and *Machimosaurus* (SMNS 91415; Young et al., 2014). This particular character also does not score for the elongated antorbital/preorbital cavity of metriorhynchoids.

This character was modified from Young (2006) and Andrade et al. (2011). It was included in Wilkinson, Young & Benton (2008), Young & Andrade (2009), Young et al. (2012) and Young et al. (2013). It is part of the H+Y datasets from Young et al. (2016), Ristevski et al. (2018), Ősi et al. (2018), Foffa et al. (2019), Johnson et al. (2019) and Sachs et al. (2019a, 2019b).

**102.** Supratemporal fenestrae, shape is either longitudinal ellipsoid or subrectangular (0), square-shaped (regular quadrilateral) (1), transverse (= extended) triangle (2), circular (3), triangle-shaped (three 60° points) (4), or parallelogram (5) (Fig. 55).

The supratemporal fenestrae, large openings for jaw musculature in the posterior half of the skull, are bordered by the frontal anteromedially, postorbital anterolaterally, squamosal posterolaterally and parietal posteromedially. The postorbital and squamosal contact one another along the lateral border, forming the supratemporal arch. Large supratemporal fenestrae increase the area for additional jaw adductor musculature (Romer, 1956).

Teleosauroids show variance in the shape of the supratemporal fenestrae. The majority of taxa have a sub-rectangular shaped fenestra, in which the anteroposterior axis is greater than 10% longer than the lateromedial axis (state 0). This is the condition seen in *Plagiophthalmosuchus* (NHMUK PV OR 14792; MNHNL TU515), *Platysuchus* (SMNS 9930), the Chinese teleosauroid (IVPP V 10098), *Mycterosuchus* (NHMUK PV R 2617), *Aeolodon* (MNHN.F.CNJ 78), *Sericodon* (Schaefer, Püntener & Billon-Bruyat, 2018), *Bathysuchus* (unnumbered LPP specimen), *Macrospondylus* (MMG BwJ 565), *Clovesuurdameredeor* (NHMUK PV OR 49126), *Charitomenosuchus* (NHMUK PV R 3320), *Pr.* cf. *bouchardi* (Lepage et al., 2008), *Proexochokefalos* (MNHN.F 1890-13) and *Neosteneosaurus* (NHMUK PV R 2865, PETMG R178). Two teleosauroids, *I. potamosiamensis* (PRC-11) and *Teleosaurus* (MNHN AC 8746), show state 1, which is square-shaped supratemporal fenestrae; as with state 0, the anteroposterior axis is over 10% longer than the lateromedial axis. In *Mystriosaurus* (NHMUK PV OR 14781; Sachs et al., 2019a), the openings are approximately isosceles trapezoid-shaped (roughly sub-square). In Machimosaurini (*Yvridiosuchus*: OUMNH J.29850; *Lemmysuchus*: NHMUK PV R 3168; *Mac. buffetauti*: SMNS 91415; *Mac. mosae*: IRSNB cast, Young et al., 2014; *Mac. hugii*: NMS 7029) the supratemporal fenestrae are extremely elongated and parallelogram-shaped (state 5), with the lateral and medial margins, and anterior and posterior margins being sub-parallel. This state is a putative apomorphy within machimosaurins.

This character has been heavily modified from Young & Andrade (2009), Andrade et al. (2011), Young et al. (2011), Young et al. (2012), Young et al. (2013), Young (2014) and Young et al. (2016). It was then included in the H+Y datasets from Ristevski et al. (2018), Ősi et al. (2018), Foffa et al. (2019), Johnson et al. (2019), Sachs et al. (2019a, 2019b). This character is a combination of character 111 from Andrade et al. (2011) and characters 50 to 52 from Young et al. (2016).

**103.** Anterior margin shape of supratemporal fenestra, no anterolateral expansion of the supratemporal fenestrae/fossae (0), or the anterior margin noticeably inclined anterolaterally (1) (Fig. 55).

The anterior margin of the supratemporal fenestra is another feature distinguishing two main teleosauroid families. It is formed by the frontal and postorbital (the postorbital bar) anteriorly and laterally, and the frontal medially. In most teleosauroids, this margin is not anterolaterally expanded, and the anterolateral corners of the supratemporal fossae are parallel to the anteromedial corners, which makes the anterior margin of the supratemporal fenestrae appear horizontal in dorsal view (state 0). This condition is seen in the basal teleosauroid *Plagiophthalmosuchus* (NHMUK PV OR 17892) as well as one teleosauroid subclade (*Macrospondylus* MMG BwJ 565; *Clovesuurdameredeor*: NHMUK PV OR 49126; *Charitomenosuchus*: NHMUK PV R 3320; *Seldsienean*: MMT P28-1; *Deslongchampsina*: OUMNH J.29851; *Proexochokefalos*: MNHN.F 1890-13; *Pr.* cf. *bouchardi*: Lepage et al., 2008; *Neosteneosaurus*: PETMG R178; *Yvridiosuchus* OUMNH J.29850; *Lemmysuchus*: NHMUK PV R 3168; *Mac. buffetauti*: SMNS 91415; *Mac. mosae*: Hua, 1999; *Mac. hugii*: NMS 7029; *Mac. rex*: Fanti et al., 2016). However, in the second subclade, the anterolateral corners of the supratemporal fossae are noticeably more inclined anteriorly than the anteromedial corners of the supratemporal fossae (state 1), giving the anterior margin an anteroposteriorly tilted appearance in dorsal view. State 1 is seen in *Mystriosaurus* (NHMUK PV OR 14781), the Chinese teleosauroid (IVPP V 10098), *Platysuchus* (SMNS 9930), *Mycterosuchus* (NHMUK PV R 2617), *Indosinosuchus* (PRC-11, PRC-239) and *Aeolodon* (MNHN.F.CNJ 78). This ‘tilted’ anterior margin of the supratemporal fenestra is well displayed in *Mystriosaurus* (NHMUK PV OR 14781).

Ristevski et al. (2018) first included this character the H+Y dataset and is in updated versions by Ősi et al. (2018), Foffa et al. (2019) Johnson et al. (2019) and Sachs et al. (2019a, 2019b).

**104.** Supratemporal fenestrae, overall anteroposterior length is either less than or sub-equal to the anterior width (0), or is twice as long as the anterior width, or more (1) (Fig. 55).

This character is related in part to ch. 102, specifically regarding the parallelogram-shaped supratemporal fenestrae see in Machimosaurini. It concerns the overall anteroposterior elongation of the supratemporal fenestra and how the mediolateral width relates to total length. It is important to note that this character is not homologous to the anteroposterior elongation of the supratemporal fenestrae in other clades, as the extreme anteroposterior elongation of the proötics, laterosphenoids, postorbital posterior processes, parietal anterior process and frontal posterior process causes the elongation.

In most teleosauroids, the anteroposterior length of the supratemporal fenestrae is approximately the same as the width (state 0). This condition is in the basal-most form *Plagiophthalmosuchus* (NHMUK PV OR 14792) as well as *Mystriosaurus* (NHMUK PV OR 14781), *Indosinosuchus* (PRC-11; PRC-239), *Platysuchus* (SMNS 9930), *Teleosaurus* (MNHN AC 8746), *Mycterosuchus* (NHMUK PV R 2617), *Bathysuchus* (unnumbered LPP specimen), *Aeolodon* (MNHN.F.CNJ 78), *Macrospondylus* (MMG BwJ 565), *Clovesuurdameredeor* (NHMUK PV OR 49126), *Charitomenosuchus* (NHMUK PV R 3806) and *Deslongchampsina* (OUMNH J.29851). In more derived teleosauroids, the anteroposterior width of the supratemporal fenestrae are approximately twice as long as the width (state 1). This condition is in *Proexochokefalos* (MNHN.F 189013), *Pr.* cf. *bouchardi* (Lepage et al., 2008), *Neosteneosaurus* (PETMG R178) and machimosaurins (*Yvridiosuchus*: OUMNH J.29850; *Lemmysuchus*: NHMUK PV R 3168; *Machimosaurus*: SMNS 91415, IRSNB cast, Young et al. [2014]). In the genus *Machimosaurus*, the width of the supratemporal fenestrae increases but the extreme elongation of the bones is still present.

Ristevski et al. (2018) first included this character in the corresponding HY dataset, with the focus being on goniopholidids. It has since then been used in Ősi et al. (2018), Foffa et al. (2019), Johnson et al. (2019) and Sachs et al. (2019a, 2019b).

**151.** The circumorbital dorsal margins of the orbits are flush with the skull dorsal surface (0), upturned (prominent along the orbital medial margin in dorsal view, with the frontal interorbital margins being upturned) (1), or upturned along with the posterior margins (the frontal lateral process anterior margins are also upturned) (2) (Fig. 52).

This character is linked with character 10 (see above) and concerns the shape of telescopic orbits. In taxa with enlarged, protruding eyes, the dorsal margin of the orbit (which includes the prefrontal and the lacrimal) is abruptly dorsally oriented. This creates a slight ridge along this margin. In the majority of teleosauroids, the orbital dorsal margins are flush (=flattened) with the skull dorsal surface (state 0) and display no evidence of any dorsal upturn. This condition is seen in the basal teleosauroid *Plagiophthalmosuchus* (NHMUK PV OR 14792) as well as *Mystriosaurus* (NHMUK PV OR 14781), the Chinese teleosauroid (IVPP V 10098), *I. kalasinensis* (PRC-239), *Platysuchus* (SMNS 9930), *Macrospondylus* (MMG BwJ 565), *Clovesuurdameredeor* (NHMUK PV OR 49126), *Charitomenosuchus* (NHMUK PV R 3320), *Deslongchampsina* (OUMNH J.29851), *Proexochokefalos* (MNHN.F 1890-13), *Neosteneosaurus* (NHMUK PV R 2865) and Machimosaurini (*Yvridiosuchus*: OUMNH J.1401; *Lemmysuchus*: LPP.M.21; *Mac. buffetauti*: SMNS 91415; *Mac. mosae*: Hua, 1999; *Mac. hugii*: Young et al., 2014). Four teleosauroid taxa (*I. potamosiamensis*: PRC-11; *Mycterosuchus*: NHMUK PV R 2617; *Teleosaurus*: MNHN AC 8746; *Aeolodon*: MNHN.F.CNJ 78) have a definitive upturning of the orbital dorsal margin (state 1), contributing to the protruding appearance of the orbits. This condition may also be present in *Bathysuchus*; however, the only available skull (unnumbered LPP specimen) is severely anteroventrally distorted in the orbital areas (note that it appears intact in dorsal view, but in lateral view, it is evident of this misconfiguration). Due to incomplete material and poor preservation, this taxon is currently scored as (?). In *Seldsienean* (MMT P28-1), the upturn is significantly less than in *I. potamosiamensis*, *Mycterosuchus*, *Teleosaurus* and *Aeolodon*.

This character was initially modified from Brochu (1999) and Salas-Gismondi et al. (2016), and was included in Ősi et al. (2018), Foffa et al. (2019), Johnson et al. (2019) and Sachs et al. (2019a, 2019b).

**158.** Orbit, the postorbital is excluded from the orbit posteroventral margin or only present in the posteroventral margin (0), or the postorbital reaches the orbit posteroventral margin and extensively forms part of the orbit ventral margin (1) (Fig. 56).

The postorbital is the bone situated directly behind the orbit, forming the immediate posterior orbital margin as well as the lateral and posteroventral borders of the supratemporal fenestra. In most teleosauroids, the postorbital does not contact the posteroventral margin of the orbit (state 0). This is the condition seen in the basal-most teleosauroid (*Plagiophthalmosuchus*: MNHNL TU515, NHMUK PV OR 14792) as well as more derived taxa (e.g. *Charitomenosuchus*: NHMUK PV R 3806; *Deslongchampsina*: OUMNH J.29851; *Proexochokefalos*: MNHN.F 1890-13; *Neosteneosaurus*: NHMUK PV R 2865, PETMG R178; *Yvridiosuchus*: OUMNH J.29850; *Mac. mosae*: IRSNB cast). However, in some teleosauroid taxa, the postorbital contacts the posteroventral margin of the orbit, forming a substantial proportion of the orbital ventral margin. Due to this extension, the postorbital often overlaps the posterior part of the jugal. This condition (state 1) is found in basal teleosauroids (*Mystriosaurus*: NHMUK PV OR 14781; the Chinese teleosauroid: IVPP V 10098; *I. potamosiamensis*: PRC-11; *Platysuchus*: SMNS 9930; *Teleosaurus*: MNHN AC 8746; *Mycterosuchus*: CAMSM J.1420).

It is important to note that some dorsoventrally crushed skulls appear as if they have state 1 (e.g. certain specimens of *Macrospondylus*). This character was initially combined with another character in Young & Andrade (2009), Young et al. (2011) and Young et al. (2013). It has been included in Young et al. (2012) and Young (2014), and in the H+Y datasets from Young et al. (2016), Ristevski et al. (2018), Ősi et al. (2018), Foffa et al. (2019) Johnson et al. (2019) and Sachs et al. (2019a, 2019b).

**225.** Basisphenoid, exposure anterior to the quadrates in palatal view: absent or basisphenoid terminates approximately level to the anterior extent of the quadrates (0), or basisphenoid ‘rostrum’ (= cultriform process) is exposed along the palatal surface anterior to the quadrates and continues to bifurcate the pterygoids (1) (Fig. 57).

The basisphenoid is a bone of the posterior cranium that forms the floor of the braincase, anterior to the basioccipital. It contacts the basioccipital ventrally, the laterosphenoid dorsally and the quadrate anteromedially. The posterior basisphenoid is broad, and the anterior basisphenoid is elongated and narrow (Brusatte et al., 2016). There are two elongated anteroposteriorly- and posterolaterally-directed processes (=prongs) that can also be seen in occipital view.

In certain teleosauroids, when examining the anterior exposure of the basisphenoid in palatal view, this bone is either absent or terminates approximately at the level of the anterior-most quadrates (state 0). This is the condition seen in *I. potamosiamensis* (PRC-11) and *Mycterosuchus* (CAMSM J.1420). In addition, it is important to note that this morphology is absent in both *Teleosaurus* (MNHN AC 8746) and the Chinese teleosauroid (IVPP V 10098) (also scored as state 0). In the majority of teleosauroids, the basisphenoid is well exposed along the palatal surface anterior to the quadrates and bifurcates the pterygoids (state 1), which is caused by the posterior expansion of the posterior margin of the pterygoid; the anterior part of the quadrates, and the lateral margins of the basisphenoid, are obscured. However, a distinct basisphenoid ‘rostrum’ is present that continues to separate the pterygoids anteriorly. State 1 is a putative synapomorphy of one teleosauroid subclade and is seen in *Macrospondylus* (SMNS 81699), *Clovesuurdameredeor* (NHMUK PV OR 49126), *Charitomenosuchus* (NHMUK PV R 3320), *Deslongchampsina* (OUMNH J.29851), *Proexochokefalos* (MNHN.F 1890-13), *Neosteneosaurus* (NHMUK PV R 2865), *Yvridiosuchus* (OUMNH J.403) and *Lemmysuchus* (LPP.M.21). State 1 also appears to be present in *Clovesuurdameredeor* (NHMUK PV OR 49126), but the anterior basisphenoid is poorly preserved; this taxon is therefore currently scored as (?). In addition, it is not present in the basal metriorhynchoid *Pelagosaurus* or members of Metriorhynchidae.

Wilkinson, Young & Benton (2008) first included this character in a phylogenetic matrix, which was later modified in Young & Andrade (2009) and Young et al. (2011). The current written character is found in Young et al. (2012), Young et al. (2013), Young (2014) and Young et al. (2016), and within the H+Y datasets from Ristevski et al. (2018), Ősi et al. (2018), Foffa et al. (2019) Johnson et al. (2019) and Sachs et al. (2019a, 2019b).

**327.** Teeth along the entirety of the tooth row, with sharp, pointed apices (0) or blunt, round apices (1) (Fig. 40).

Teeth that are elongate and slender with pointed apices (state 0) can clearly be seen in the basal-most form *Plagiophthalmosuchus* (MNHNL TU515) and in most teleosauroids (e.g. *I. kalasinensis*: PRC-238, PRC-239; *Platysuchus*: SMNS 9930; *Mycterosuchus*: NHMUK PV R 2617; *Bathysuchus*: DORCM G.05067iv; *Sericodon* (TCH005-151 in Schaefer, Püntener & Billon-Bruyat, 2018), *Macrospondylus*: MNHNL TU799; *Charitomenosuchus*: NHMUK PV 3806; *Seldsienean*: OUMNH J.1414). While the taxa *Mystriosaurus* (HLMD V946-948, NHMUK PV OR 14781), *Proexochokefalos* (MNHN.F 1890-13), *Deslongchampsina* (OUMNH J.29851) and *Neosteneosaurus* (PETMG R178) possess teeth with pointed apices (and are therefore scored as state 0), it is important to note that the overall dentition of these four genera are more robust than in the other aforementioned teleosauroids. In particular, the posterior teeth of *Neosteneosaurus* (PETMG R178) are noticeably more conical but continue to retain a pointed apex. The tribe Machimosaurini (Jouve et al., 2016) is unique in that all members (*Yvridiosuchus*: OUMNH J.29850; *Lemmysuchus*: NHMUK PV R 3618; *Machimosaurus*: LMH 16387, LMH 16405, MG-8730-1, ONM NG 7, SMF 2027, SMNS 91415) have conical teeth with blunt, rounded apices (state 1) throughout the entirety of the dentition. Von Meyer (1837) initially noted the rounded apices of these teeth in *Mac. hugii*, and since then this character has become a staple of machimosaurin dental morphology.

Young et al. (2011) first included this character into a phylogenetic matrix, and it has been subsequently included in the following HY datasets: Young et al. (2016); Ristevski et al. (2018); Ősi et al. (2018); Foffa et al. (2019) Johnson et al. (2019) and Sachs et al. (2019a, 2019b).

**358.** Morphology of apical enamel surface ornamentation, macroscopic anastomosed pattern absent (0) or present (1) (Fig. 40).

As with the above character, the apices of the teeth are relatively smooth and unornamented aside from the enamel ridges that reach the tip of the apex (state 0) in most teleosauroids. This is the condition seen in *Plagiophthalmosuchus* (MNHNL TU515), as well as *Mystriosaurus* (NHMUK PV OR 14781); *I. kalasinensis* (PRC-239); *Platysuchus* (SMNS 9930); *Teleosaurus* (Eudes-Deslongchamps, 1867-69); *Mycterosuchus* (NHMUK PV R 2617); *Bathysuchus* (DORCM G.05067iv); *Sericodon* (TCH005-151 in Schaefer, Püntener & Billon-Bruyat, 2018); *Aeolodon* (NHMUK PV R 1086); *Macrospondylus* (MNHNL TU799); *Charitomenosuchus* (NHMUK PV R 3806); *Seldsienean* (OUMNH J.1414); *Deslongchampsina* (OUMNH J.29851); *Proexochokefalos* (MNHN.F 1890-13); and *Neosteneosaurus* (NHMUK PV R 3701; PETMG R178). However, the tribe Machimosaurini evolved a complex ornamentation pattern (state 1), in addition to other features of the dentition (see above), hypothesised to be related to a more durophagous diet (Johnson, Young & Brusatte, 2019). This pattern is often referred to as ‘anastomosed’, which is described in zoological terms as the joining of structures to form a branching network. In machimosaurin teeth, this manifests as the branching and criss-crossing of enamel ridges: this translates into a rough, ‘wrinkled’ texture, visible to the naked eye, on the apical third of the tooth. Anastomosed teeth are one of the characteristic features in machimosaurins, present in all members of the group (*Yvridiosuchus*: OUMNH J.29850; *Lemmysuchus*: NHMUK PV R 3168; *Machimosaurus*: SMNS 91415, MG-8730-1, ONM NG 7, SMF 2027). This type of anastomosed pattern also appears in the geosaurin *Torvoneustes* (Andrade, Young & Desojo, 2010) and select members of Goniopholididae (e.g. *Anteophthalmosuchus* and *Goniopholis*: see Andrade et al., 2011; Salisbury & Naish, 2011).

Young et al. (2012) first described and included this character in a phylogenetic dataset. It has since then been listed in Young (2014) as well as the H+Y versions found in Young et al. (2016), Ristevski et al. (2018), Ősi et al. (2018), Foffa et al. (2019), Johnson et al. (2019) and Sachs et al. (2019a, 2019b).

**379.** Number of sacral vertebrae: two (0) or three (1) (Fig. 43).

In the majority of teleosauroids, there are two sacral vertebrae (state 0). This condition is seen in the basal form *Plagiophthalmosuchus* (NHMUK PV OR 14792) as well as *Platysuchus* (SMNS 9930), *Teleosaurus* (NHMUK PV OR 32588), *Mycterosuchus* (NHMUK PV R 2617), *Aeolodon* (MNHN.F.CNJ 78), *Macrospondylus* (SMNS 52034), *Charitomenosuchus* (NHMK PV R 3806), and *Neosteneosaurus* (NHMUK PV R 3701, PETMG R178). However, in scored members of Machimosaurini (*Lemmysuchus*: NHMUK PV R 3618; *Mac. mosae*: IRSNB cast, Hua, 1999), three sacral vertebrae are present, which is a unique feature of this clade. The first two vertebrae are true sacrals, with the first caudal vertebra appearing and functioning as a third sacral. This ‘pseudosacral’ has large, mediolaterally expanding transverse processes as well as an expanded lateral iliac attachment area, similar to the two primary sacrals.

Buscalioni & Sanz (1988) initially reported the presence of more than two lumbar (sacral) vertebrae in crocodylomorphs in atoposaurids. Afterwards, Pol & Apesteguia (2005) reported the fusion of sacral two plus the first caudal vertebra for *Notosuchus*. Andrade et al. (2011) mentioned that the number of sacral vertebrae is increased by adding the last dorsal/lumbar or the first caudal, and that the fusion found in *Notosuchus* differs from that in *Alligatorellus* and *Montsecosuchus* (fusion of the first and second sacrals). Specifically, for teleosauroids, this character was first noted in detail by Andrews (1913) when describing ‘*Steneosaurus*’ *obtusidens* (= *Lemmysuchus* *obtusidens*). Recently, Scheyer et al. (2019) reported three sacral vertebrae (two dorsals and one functioning dorsosacral) in the giant caimanine *Purussaurus mirandai* Aguilera, Riff & Bocquentin-Villanueva, 2006. The authors suggested that a longer sacrum (with three sacrals) may be hypothetically related to large size and body mass, or a more upright limb orientation, increasing the stability of this area of the vertebral column and improving transmission of forces across the pelvis and caudal region (Scheyer et al., 2019).

The three sacrals, in relation to teleosauroids, have been described and figured by Young et al. (2014) and Johnson et al. (2017), and have been included in the datasets by Andrade et al. (2011), Ristevski et al. (2018), Ősi et al. (2018), Foffa et al. (2019), Johnson et al. (2019) and Sachs et al. (2019a, 2019b).

**410.** Humerus, humeral head: confined to the proximal surface (0), gently posteriorly expanded and hooked (1), or very strongly posteriorly deflected and hooked (2) (Fig. 58).

In scored teleosauroids, the proximal area of the humerus is either gently posteriorly expanded and hooked (state 1) or strongly deflected and hooked (state 2); it is never confined to the proximal surface (state 0). In basal teleosauroids such as *Plagiophthalmosuchus* (NHMUK PV OR 14792), *Platysuchus* (SMNS 9930), *Teleosaurus* (OUMNH J.26801), *Macrospondylus* (SMNS 51957) and *Mycterosuchus* (NHMUK PV R 2617), the proximal humerus (or humeral head) is anteroposteriorly elongated and gently but noticeably hooked (state 1). Of the aforementioned taxa, the humeral head of *Macrospondylus* (SMNS 51563) is the most elongated and appears somewhat rod-like, with a very slight hook. While currently scored as state 1, it is interesting to note that the humerus of *Mycterosuchus* (NHMUK PV R 2617) is odd: while still hooked, it is noticeably less so than in other teleosauroids (e.g. *Charitomenosuchus*: NHMUK PV R 3806; *Neosteneosaurus*: PETMG R178), with the proximal end being more circular, proximally oriented and ‘club’-like. However, it is important to consider that this feature may be due to dorsoventral crushing, as many OCF taxa are prone to this type of preservation. In the teleosauroids *Aeolodon* (MNHN.F.CNJ 78), *Charitomenosuchus* (NHMUK P R 3806) and *Neosteneosaurus* (PETMG R178), the posterior deflection of the proximal humerus is strong, so much so that the proximal epiphysis is noticeably posterior to the distal epiphysis. This posterior deflection is much more pronounced than in any other thalattosuchian taxa.

This character was initially modified from Nesbitt (2011), and again in Young et al. (2012). Young et al. (2016) included the addition of state 2. This updated version has been used in Ristevski et al. (2018), Ősi et al. (2018), Foffa et al. (2019) Johnson et al. (2019) and Sachs et al. (2019a, 2019b).

**420.** Ulna, olecranon process mediolaterally compressed and greatly proximally expanded: no (0), yes (1) (Fig. 44).

The olecranon process (the expanded concavity of the proximal ulna) forms the elbow of the forelimb and articulates with the proximal head of the radius. It is an insertion point for the *triceps longus lateralis*, *triceps longus medialis* and *anconeus humeralis lateralis* (all involved with elbow extension) (Klinkhamer et al., 2017). Creating a very broad olecranon process allows for greater surface area for muscle attachment. Only two basal teleosauroids (*Platysuchus*: SMNS 9930; *Macrospondylus* SMNS 53422) score as 0, in which the olecranon process is neither compressed nor expanded. Interestingly, more derived teleosauroids score as state 1, where the olecranon process is both greatly expanded and mediolaterally compressed. This is seen in *Mycterosuchus* (NHMUK PV R 2617), *Aeolodon* (MNHN.F.CNJ 78), *Charitomenosuchus* (NHMUK PV R 3806), *Neosteneosaurus* (PETMG R178) and *Lemmysuchus* (NHMUK PV R 3168).

Ősi et al. (2018) first defined this character, and it is listed in recent datasets (Foffa et al., 2019; Johnson, Young & Brusatte, 2019; Sachs et al., 2019a, 2019b).

**440.** Ilium, postacetabular (= posterior) process expanded into a thin ‘fan’ shape: no (0), yes (1) (Fig. 46).

In most teleosauroids, the postacetabular (=posterior) iliac process, which is situated posteriorly on the dorsal margin of the ilium, is either anteroposteriorly shortened, robust and process-like (state 0) or anteroposteriorly expanded and mediolaterally thin, expanding it into a ‘fanlike’ shape (state 1), and is best seen in either lateral or medial view. In *Charitomenosuchus* (NHMUK PV R 3806), *Neosteneosaurus* (PETMG R178), *Lemmysuchus* (NHMUK PV R 3816) and *Mac. mosae* (Young et al., 2014), state 1 is present, with the postacetabular process lengthened into a mediolaterally thin ‘fan-like’ shape. However, it is important to note that state 1 is a putative apomorphy of derived teleosauroids, and is not seen in basal taxa such as *Plagiophthalmosuchus* (NHMUK PV OR 14792), *Platysuchus* (SMNS 9930), *Teleosaurus* (NHMUK PV OR 32588), *Sericodon* (SCR010-312 in Schaefer, Püntener & Billon-Bruyat, 2018) and *Macrospondylus* (SMNS 18672, SMNS 51753).

Young et al. (2012) first highlighted this character, and was subsequently found in Young (2014), Wilberg (2015b), Young et al. (2016), Ristevski et al. (2018), Ősi et al. (2018), Foffa et al. (2019) Johnson et al. (2019) and Sachs et al. (2019a, 2019b).

**473.** Ornamentation (dorsal osteoderms), the pits are either small round to ellipsoid and very densely distributed (0), large round to ellipsoid and well separated (1), irregularly shaped with an extreme variation in size, with elongate pits present on the ventrolateral surface running from the keel to the lateral margin (2), or variable in both size, shape and length that radiate in a starburst pattern (3) (Fig. 51).

Osteoderms are bony deposits that form scales, plates, or other structures within the skin. These dorsal structures are highly developed and are arranged in dorsal longitudinal rows (Seidel, 1979), and display a delay in development when compared to the rest of the skeleton (Vickaryous & Hall, 2008). In extant crocodylians, the pitting ornamentation of dorsal osteoderms are vascularized (Seidel, 1979; Grigg & Seebacher, 2001; Young et al., 2014; Clarac et al., 2019), and may play a role with regards to thermoregulation or basking behaviours (Young et al., 2014; Clarac et al., 2019). In teleosauroids, and other fossil crocodylomorph groups, two parallel rows of mediolaterally elongate osteoderms contribute to the paramedian shield (Johnson et al., 2018). The shape of the osteoderms differs in specific areas of the body (Andrews, 1913). Cervical ones are small and generally box- or square-shaped, with a very faint or absent keel and no anterolateral process. The thoracic/sacral dorsal osteoderms (which are typically the largest, notably in the sacral area) are anteroposteriorly elongated and either oval or rectangular in shape. Generally, a pronounced, anteroposteriorly directed keel runs the length of the osteoderm, and the anterolateral process is well developed. In the caudal area, the osteoderms begin similar in shape and size to the sacral ones, but become progressively smaller and more subcircular, with the keel disappearing in the posterior-most ones. In the more posteriorly placed caudal osteoderms, the anterolateral process takes up the entirety of the anterior margin. The dorsal area of all osteoderms is convex and covered with pits, and the ventral area is smooth, slightly concave and unaltered.

While the overall shape of the dorsal osteoderms is consistent in certain areas of the body across taxa, the ornamentation (or pitting) pattern differs, most notably in the thoracic/sacral osteoderms. In most teleosauroids, the pits are large, subcircular to ellipsoid in shape, and generally well separated from one another. This condition (state 1) is seen in *Plagiophthalmosuchus* (NHMUK PV OR 14792), *Mycterosuchus* (NHMUK PV R 2617), *Charitomenosuchus* (NHMUK PV R 3806) and *Neosteneosaurus* (NHMUK PV R 2865; NHMUK PV R 3701; PETMG R178). In *Charitomenosuchus* (NHMUK PV R 3806), the pits are arranged in a semi-circular pattern, and the larger ones are situated more towards the lateral margins of the osteoderm. In *Neosteneosaurus* (NHMUK PV R 2865), most pits are exceptionally large (especially situated in the centre of the osteoderm), subcircular and fewer in number. While the osteoderm ornamentation in the holotype of *Macrospondylus* (MMG BwJ 595) is poorly preserved, the pits appear to be large and semi-ellipsoid with a strong anteroposterior keel. The pits also appear to be more closely placed to one another, which is observed in other *Macrospondylus* specimens (e.g. MMG BwJ 565; SMNS 51563; SMNS 51753), with a thin ridge separating them. In two teleosauroid taxa, the ornamental pits are small, round, and extremely densely distributed throughout the entirety of the dorsal osteoderms (state 0). This is seen in *Platysuchus* (SMNS 9930) and *Teleosaurus* (NHMUK PV R 119a). Certain teleosauroids, however, possess thoracic/sacral osteoderms with exceptionally enlarged, elongated pits; due to this elongation and large size, these pits merge with one another and become elongated grooves, especially along the lateral margins, with the pits radiating distally in a ‘starburst’ pattern (state 3). The remainder of the pits are variable in size (from small to large), irregularly shaped, and relatively close together. In addition, well-developed keels are generally present in these osteoderms. This condition is observed in machimosaurins (*Lemmysuchus*: NHMUK PV R 3618; *Machimosaurus*: ONM 1-25, SMNS 91415, Young et al., 2014). State 2, in which the pits are all irregularly shaped with extreme variation in size and have no ‘starburst’ pattern, is not present in any known teleosauroid taxa.

Young et al. (2011) first observed this difference in ornamentation, and it has since been used, often in modified form, in the following studies: Young et al. (2012); Young et al. (2013); Young (2014); Young et al. (2016); and Ristevski et al. (2018). The current iteration of this character can be found in Ősi et al. (2018), Foffa et al. (2019), Johnson et al. (2019) and Sachs et al. (2019a, 2019b).
