# Supplementary material for: The phylogenetics of Teleosauroidea (Crocodylomorpha, Thalattosuchia) and implications for their ecology and evolution
Source: PeerJ. 2020 Oct 8;8:e9808. doi: 10.7717/peerj.9808 (PMC7548081; doi:10.7717/peerj.9808)
Supplement: Supplemental Information 6 [file peerj-08-9808-s006.docx]

List of important teleosauroid specimens, including specimen accession number, institution where each specimen is located, taxon attributed and type of material (e.g. skull, nearly complete skeleton). Note that some Teleosauroidea indeterminate specimens are included, as they represent significant material.

| **Specimen Number** | **Location (Institution)** | **Taxon** | **Type of Material** |
| --- | --- | --- | --- |
| BHN2 R 59 | Muséum d’Histoire Naturelle de Boulogne-sur-Mer (France) (closed in 2003) | *Proexochokefalos* cf. *bouchardi* | Skull material |
| BIRUG 102704 | Lapworth Museum of Geology (UK) | *Lemmysuchus* cf. *obtusidens* | Partial skull |
| BRLSI GP1770a-e | Bath Royal Literary and Scientific Institution (UK) | *Charitomenosuchus leedsi* | Complete skull and mandible |
| BRLSI M.1698 | Bath Royal Literary and Scientific Institution (UK) | *Teleosaurus cadomensis* | Osteoderms and small elements |
| BSY006-348 | A16 Paleontology Porrentruy (l’Office fédéral des routes and la République et Canton du Jura) | *Sericodon jugleri* | Partial mandible |
| BSY007-134 | A16 Paleontology Porrentruy (l’Office fédéral des routes and la République et Canton du Jura) | *Sericodon jugleri* | Partial mandible |
| BSY008-622 | A16 Paleontology Porrentruy (l’Office fédéral des routes and la République et Canton du Jura) | *Sericodon jugleri* | Partial mandible |
| CAMSM J.1420 | Sedgwick Museum of Earth Science | *Mycterosuchus nasutus* | Nearly complete skeleton |
| DFMMh FV 330 | Dinosaurier-Freilichtmuseum Münchehagen (Germany) | *Machimosaurus buffetauti* | Isolated tooth crown |
| DFMMh FV 541 | Dinosaurier-Freilichtmuseum Münchehagen, (Germany) | *Machimosaurus buffetauti* | Isolated tooth crown |
| DONMG unnumbered | Doncaster Museum (UK) | *Plagiophthalmosuchus gracilirostris* | Nearly complete skull and mandible |
| DORCM G.05067i-v | Dorset County Museum | *Bathysuchus megarhinus* | Premaxillae, isolated tooth and partial osteoderm |
| GPIT-RE-7286 | Paläontologische Sammlung der Eberhard Karls Universität (Germany) | *Neosteneosaurus edwardsi* | Complete skeleton |
| GPIT-RE-9425 | Paläontologische Sammlung der Eberhard Karls Universität (Germany) | *Macrospondylus bollensis* | Complete skeleton |
| GPIT-RE-9427 | Paläontologische Sammlung der Eberhard Karls Universität (Germany) | *Macrospondylus bollensis* | Nearly complete skeleton |
| GPIT-RE-09146 | Paläontologische Sammlung der Eberhard Karls Universität (Germany) | Teleosauroidea indeterminate | Multiple associated postcranial elements |
| GZG unnumbered | Geologisches institute Geologisch-Paläontologisches (Germany) | *Mycterosuchus nasutus* | Complete skull |
| GZG unnumbered | Geologisches institute Geologisch-Paläontologisches (Germany) | *Sericodon jugleri* | Partial skull |
| HLMD V946-948 | Hessisches Landesmuseum | *Mystriosaurus laurillardi* | Partial skull |
| IRSNB unnumbered | Institut Royal des Sciences Naturelles de Bruxelles (Belgium) | *Machimosaurus mosae* | Cast of complete skeleton |
| IRSNB R 0140 | Institut Royal des Sciences Naturelles de Bruxelles (Belgium) | Teleosauroidea indeterminate | Partial skull |
| IRSNB R 0144 | Institut Royal des Sciences Naturelles de Bruxelles (Belgium) | Teleosauroidea indeterminate | Partial mandible |
| IVPP V 10098 | Institute of Paleontology and Paleoanthropology (China) | The Chinese teleosauroid previously referred to as *Peipehsuchus teleorhinus* | Complete skull |
| LMH 16645-46 | Landesmuseum Hannover | *Sericodon jugleri* | Anterior mandible |
| LPP unnumbered | Institut de paléoprimatologie, paléontologie, humaine évolution et paléoenvironnements Université de Poitiers (France) | *Bathysuchus megarhinus* | Partial rostrum, mandible and skull |
| LPP.T.1 | Institut de paléoprimatologie, paléontologie, humaine évolution et paléoenvironnements Université de Poitiers (France) | *Seldsienean megistorhynchus* | Partial mandible |
| LPP.M.21 | Institut de paléoprimatologie, paléontologie, humaine évolution et paléoenvironnements Université de Poitiers (France) | *Lemmysuchus obtusidens* | Nearly complete skull and mandible |
| MCNV-CC-4 | Museo de Ciencias Naturales de Valencia (Spain) | *Machimosaurus hugii* | Isolated tooth crown |
| MG unnumbered | Museu Geológico (Portugal) | *Machimosaurus hugii* | Isolated tooth |
| MG-25 | Museu Geológico (Portugal) | *Machimosaurus hugii* | Rostral piece |
| MG-8730-1 | Museu Geológico (Portugal) | *Machimosaurus hugii* | Rostral piece |
| MG-8730-2 | Museu Geológico (Portugal) | *Machimosaurus hugii* | Occipital |
| ML 491 | Museu da Lourinhã (Portugal) | *Machimosaurus hugii* | Isolated tooth |
| ML 647 | Museu da Lourinhã (Portugal) | *Machimosaurus hugii* | Isolated tooth |
| ML 657 | Museu da Lourinhã (Portugal) | *Machimosaurus hugii* | Isolated tooth |
| ML 658 | Museu da Lourinhã (Portugal) | *Machimosaurus hugii* | Isolated tooth |
| MMG BwJ 565 | Staaliches Museum für Mineralogie und Geologie (Germany) | *Macrospondylus bollensis* | Complete skeleton |
| MMG BwJ 595 | Staaliches Museum für Mineralogie und Geologie (Germany) | *Macrospondylus bollensis* | Partial postcranial skeleton |
| MMG BwJ 689 | Staaliches Museum für Mineralogie und Geologie (Germany) | *Macrospondylus bollensis* | Nearly complete skeleton |
| MMT P28-1 | Musée d'art et d'histoire de Toul (France) | *Seldsienean megistorhynchus* | Partial skull and mandible |
| MNHN unnumbered | Muséum National d’Histoire Naturelle Paris (France) | *Teleosaurus cadomensis* | Cast; anterior lower jaw with in situ teeth |
| MNHN.F 1890-13 | Muséum National d’Histoire Naturelle Paris (France) | *Proexochokefalos heberti* | Complete skull and mandible |
| MNHN.F AC 8746 | Muséum National d’Histoire Naturelle Paris (France) | *Teleosaurus cadomensis* | Partially complete skull |
| MNHN.F.CNJ 78 | Muséum National d’Histoire Naturelle Paris (France) | *Aeolodon priscus* | Nearly complete skeleton |
| MNHN.RJN 134c-d | Muséum National d’Histoire Naturelle Paris (France) | *Steneosaurus rostromajor* | Partial rostrum |
| MNHN.RJN 118 | Muséum National d’Histoire Naturelle Paris (France) | *Neosteneosaurus edwardsi* | Partial skull |
| MNHNL TU155 | Musée National d’Histoire naturelle Luxembourg (Luxembourg) | *Macrospondylus* cf. *bollensis* | Thorax |
| MNHNL TU164 | Musée National d’Histoire naturelle Luxembourg (Luxembourg) | Teleosauroidea indeterminate | Partial rostrum |
| MNHNL TU799 | Musée National d’Histoire naturelle Luxembourg (Luxembourg) | *Macrospondylus bollensis* | Partial skull and associated material |
| MNHNL TU895 | Musée National d’Histoire naturelle Luxembourg (Luxembourg) | *Platysuchus multiscrobiculatus* | Partial rostrum |
| MPV V1600.Bo | Musée paléontologique (Paléospace) de Villers-sur-Mer (France) | *Machimosaurus buffetauti* | Anterior rostrum and mandible |
| MPV V1601.Bo | Musée paléontologique (Paléospace) de Villers-sur-Mer (France) | *Machimosaurus buffetauti* | Partial rostrum |
| NHMUK PV R 324 | Natural History Museum London (UK) | *Macrospondylus bollensis* | Partial skull |
| NHMUK PV R 756 | Natural History Museum London (UK) | *Macrospondylus bollensis* | Partial skull |
| NMHUK PV R 1086 | Natural History Museum London (UK) | *Aeolodon priscus* | Nearly complete skeleton |
| NHMUK PV R 1088 | Natural History Museum London (UK) | *Macrospondylus bollensis* | Partial skeleton |
| NHMUK PV R 1752 | Natural History Museum London (UK) | *Sericodon jugleri* | Isolated teeth |
| NHMUK PV R 1999 | Natural History Museum London (UK) | *Andrianavoay baroni* | Partial skull and mandible, one osteoderm fragment |
| NHMUK PV R 2075 | Natural History Museum London (UK) | *Neosteneosaurus edwardsi* | Partial skull and mandible, and associated postcranial material |
| NHMUK PV R 2076 | Natural History Museum London (UK) | *Neosteneosaurus edwardsi* | Partial mandible and associated postcranial material |
| NHMUK PV R 2167 | Natural History Museum London (UK) | *Mycterosuchus nasutus* | Complete skull and mandible, with additional material |
| NHMUK PV R 2619 | Natural History Museum London (UK) | *Charitomenosuchus leedsi* | Complete mandible and additional material |
| NHMUK PV R 2865 | Natural History Museum London (UK) | *Neosteneosaurus edwardsi* | Complete skull, assorted vertebrae and isolated teeth |
| NHMUK PV R 3168 | Natural History Museum London (UK) | *Lemmysuchus obtusidens* | Nearly complete skeleton |
| NHMUK PV R 3320 | Natural History Museum London (UK) | *Charitomenosuchus leedsi* | Nearly complete skull |
| NHMUK PV R 3701 | Natural History Museum London (UK) | *Neosteneosaurus edwardsi* | Nearly complete skull and mandible, and partial skeleton |
| NHMUK PV R 3806 | Natural History Museum London (UK) | *Charitomenosuchus leedsi* | Nearly complete skeleton |
| NHMUK PV R 3892 | Natural History Museum London (UK) | *Mycterosuchus nasutus* | Dorsal and sacral vertebrae |
| NHMUK PV R 3898 | Natural History Museum London (UK) | *Neosteneosaurus edwardsi* | Femur, ilium and ischium |
| NHMUK PV R 4059 | Natural History Museum London (UK) | *Mycterosuchus nasutus* | Partial skull |
| NHMUK PV R 4207 | Natural History Museum London (UK) | *Teleosaurus cadomensis* | Dorsal osteoderms |
| NHMUK PV R 5703 | Natural History Museum London (UK) | *Macrospondylus bollensis* | Complete skull and mandible |
| NHMUK PV OR 119a | Natural History Museum London (UK) | *Teleosaurus cadomensis* | Dorsal osteoderms |
| NHMUK PV OR 14436 | Natural History Museum London (UK) | *Macrospondylus bollensis* | Partial skull |
| NHMUK PV OR 14438 | Natural History Museum London (UK) | *Macrospondylus bollensis* | Partial skull |
| NHMUK PV OR 14781 | Natural History Museum London (UK) | *Mystriosaurus laurillardi* | Nearly complete skull and mandible |
| NHMUK PV OR 14792 | Natural History Museum London (UK) | *Plagiophthalmosuchus gracilirostris* | Nearly complete skeleton |
| NHMUK PV OR 15500 | Natural History Museum London (UK) | *Plagiophthalmosuchus gracilirostris* | Complete skull and mandible |
| NHMUK PV OR 32588 | Natural History Museum London (UK) | *Teleosaurus cadomensis* | Dorsal, sacral and caudal vertebrae |
| NHMUK PV OR 32657 | Natural History Museum London (UK) | *Teleosaurus cadomensis* | Femur |
| NHMUK PV OR 32680 | Natural History Museum London (UK) | *Teleosaurus cadomensis* | Ischium |
| NHMUK PV OR 33124 | Natural History Museum London (UK) | *Teleosaurus cadomensis* | Mandibular symphysis |
| NHMUK PV OR 39788 | Natural History Museum London (UK) | *Teleosaurus cadomensis* | Partial rostrum |
| NHMUK PV OR 43086 | Natural History Museum London (UK) | *Bathysuchus megarhinus* | Partial rostrum |
| NHMUK PV OR 49126 | Natural History Museum London (UK | *Clovesuurdameredeor stephani* | Partial skull and anterior section of mandible |
| NHMW-1848-0031-0001 | Naturhistorisches Museum Wien (Austria) | *Macrospondylus bollensis* | Complete skeleton |
| NHMW 1856.36.22 | Naturhistorisches Museum Wien (Austria) | *Machimosaurus hugii* | Isolated tooth |
| NHMW-1878-0047-0001 | Naturhistorisches Museum Wien (Austria) | *Macrospondylus bollensis* | Complete skeleton |
| NHMW-1882-0026-4082 | Naturhistorisches Museum Wien (Austria) | *Macrospondylus bollensis* | Nearly complete skeleton |
| NMS 8342 | Naturmuseum Solothurn (Switzerland) | *Machimosaurus hugii* | Isolated tooth crown |
| NMNSJ | National Museum of Nature and Science (Japan) | *Macrospondylus bollensis* | Complete skeleton |
| NOTNH FS3361 | Nottingham Natural History Museum | *Lemmysuchus obtusidens* | Partial rostrum |
| NRM-PZ R.144 | Naturhistoriska Riksmuseet Palaeozoological (Sweden) | *Neosteneosaurus edwardsi* | Partial sacral vertebra |
| NRM-PZ R.2053 | Naturhistoriska Riksmuseet Palaeozoological (Sweden) | *Neosteneosaurus edwardsi* | Tibia |
| NRM-PZ R.2074 | Naturhistoriska Riksmuseet Palaeozoological (Sweden) | *Neosteneosaurus edwardsi* | Femur |
| NRM-PZ R.2337 | Naturhistoriska Riksmuseet Palaeozoological (Sweden) | *Sericodon jugleri* | Two isolated teeth |
| ONM NG 1-25, 80, 81, and 83-87 | Office National des Mines (Tunisia) | *Machimosaurus rex* | Partial skull with associated vertebrae, osteoderms and teeth |
| OUMNH unnumbered | Oxford University Museum of Natural History (UK) | *Macrospondylus bollensis* | Partial skull |
| OUMNH J.1401 | Oxford University Museum of Natural History (UK) | *Yvridiosuchus boutilieri* | Partial skull |
| OUMNH J.1403 | Oxford University Museum of Natural History (UK) | *Yvridiosuchus boutilieri* | Nearly complete skull |
| OUMNH J.1404 | Oxford University Museum of Natural History (UK) | *Yvridiosuchus boutilieri* | Partial mandible |
| OUMNH J.1414 | Oxford University Museum of Natural History (UK) | *Seldsienean megistorhynchus* | Near complete mandible |
| OUMNH J.1417 | Oxford University Museum of Natural History (UK) | *Yvridiosuchus boutilieri* | Partial mandible |
| OUMNH J.29815 | Oxford University Museum of Natural History (UK) | *Neosteneosaurus edwardsi* | Partial skull |
| OUMNH J.29850 | Oxford University Museum of Natural History (UK) | *Yvridiosuchus boutilieri* | Nearly complete skull and mandible |
| OUMNH J.29851 | Oxford University Museum of Natural History (UK) | *Deslongchampsina larteti* | Nearly complete skull |
| PETMG R39 | Peterborough Museum and Art Gallery (UK) | *Lemmysuchus obtusidens* | Rostral-orbital section |
| PETMG R175 | Peterborough Museum and Art Gallery (UK) | *Neosteneosaurus edwardsi* | Complete skeleton |
| PETMG R178 | Peterborough Museum and Art Gallery (UK) | *Neosteneosaurus edwardsi* | Nearly complete skeleton |
| PETMG R179 | Peterborough Museum and Art Gallery (UK) | *Charitomenosuchus leedsi* | Complete skull |
| PIN 5008s2 | Paleontological Institute (Russia) | *Macrospondylus bollensis* | Complete skeleton |
| PMU R161 | Evolutionsmuseet Uppsala Universitet (Sweden) | *Macrospondylus bollensis* | Complete skeleton |
| PRC-11 | Palaeontological Research and Education Centre (Thailand) | *Indosinosuchus potamosiamensis* | Complete skull and mandible |
| PRC-238 | Palaeontological Research and Education Centre (Thailand) | *Indosinosuchus potamosiamensis* | Partial skull and mandible |
| PRC-239 | Palaeontological Research and Education Centre (Thailand) | *Indosinosuchus kalasinensis* | Nearly complete skull and mandible |
| SCR010-312 | A16 Paleontology Porrentruy (l’Office fédéral des routes and la République et Canton du Jura) (Switzerland) | *Sericodon jugleri* | Partial skull and skeleton |
| SCR010-1184 | A16 Paleontology Porrentruy (l’Office fédéral des routes and la République et Canton du Jura) (Switzerland) | *Sericodon jugleri* | Nearly complete mandible |
| SCR011-2460 | A16 Paleontology Porrentruy (l’Office fédéral des routes and la République et Canton du Jura) (Switzerland) | *Sericodon jugleri* | Partial mandible |
| SCR011-406 | A16 Paleontology Porrentruy (l’Office fédéral des routes and la République et Canton du Jura) (Switzerland) | *Sericodon jugleri* | Partial rostrum |
| SMF R 123 | Naturmuseum Senckenberg Frankfurt (Germany) | *Neosteneosaurus edwardsi* | Complete skull and nearly complete mandible |
| SMF R 431a-b | Naturmuseum Senckenberg Frankfurt (Germany) | *Sericodon jugleri* | Isolated teeth |
| SMF R 4318 | Naturmuseum Senckenberg Frankfurt (Germany) | *Sericodon jugleri* | Isolated teeth |
| SNHM-IG-008-R | Staaliches Naturhistorisches Museum Braunschweig (Germany) | *Mystriosaurus* sp. | Complete skull and nearly complete mandible, with a partial skeleton |
| SMNS 9930 | Staatliches Museum für Naturkunde Stuttgart (Germany) | *Platysuchus multiscrobiculatus* | Nearly complete skeleton |
| SMNS 18672 | Staatliches Museum für Naturkunde Stuttgart (Germany) | *Macrospondylus bollensis* | Nearly complete skeleton |
| SMNS 20280 | Staatliches Museum für Naturkunde Stuttgart (Germany) | *Macrospondylus bollensis* | Complete skull |
| SMNS 20283 | Staatliches Museum für Naturkunde Stuttgart (Germany) | *Macrospondylus bollensis* | Partial skull |
| SMNS 51555 | Staatliches Museum für Naturkunde Stuttgart (Germany) | *Macrospondylus bollensis* | Nearly complete skeleton |
| SMNS 51563 | Staatliches Museum für Naturkunde Stuttgart (Germany) | *Macrospondylus bollensis* | Complete skeleton |
| SMNS 51753 | Staatliches Museum für Naturkunde Stuttgart (Germany) | *Macrospondylus bollensis* | Complete skeleton |
| SMNS 51957 | Staatliches Museum für Naturkunde Stuttgart (Germany) | *Macrospondylus bollensis* | Complete skeleton |
| SMNS 51984 | Staatliches Museum für Naturkunde Stuttgart (Germany) | *Macrospondylus bollensis* | Complete skeleton |
| SMNS 53422 | Staatliches Museum für Naturkunde Stuttgart (Germany) | *Macrospondylus bollensis* | Partial skeleton |
| SMNS 58876 | Staatliches Museum für Naturkunde Stuttgart (Germany) | *Macrospondylus bollensis* | Nearly complete skeleton |
| SMNS 81699 | Staatliches Museum für Naturkunde Stuttgart (Germany) | *Macrospondylus bollensis* | Nearly complete skeleton |
| SMNS 91415 | Staatliches Museum für Naturkunde Stuttgart (Germany) | *Machimosaurus buffetauti* | Complete skull and mandible, and associated postcranial material |
| SMNS 10 000 | Staatliches Museum für Naturkunde Stuttgart (Germany) | *Macrospondylus bollensis* | Nearly complete skeleton |
| TCH005-151 | A16 Paleontology Porrentruy (l’Office fédéral des routes and la République et Canton du Jura) (Switzerland) | *Sericodon jugleri* | Isolated teeth |
| TCH007-215 | A16 Paleontology Porrentruy (l’Office fédéral des routes and la République et Canton du Jura) (Switzerland) | *Sericodon jugleri* | Nearly complete mandible |
| UH 1 | Urweltmuseum Hauff Holzmaden | *Platysuchus multiscrobiculatus* | Complete skeleton |
| UH 7 | Urweltmuseum Hauff Holzmaden | *Mystriosaurus laurillardi* | Complete skull and mandible |
| VTT006-171 | A16 Paleontology Porrentruy (l’Office fédéral des routes and la République et Canton du Jura) (Switzerland) | *Sericodon jugleri* | Partial mandible |
| YORM 2012.38 | Yorkshire Museum | *Plagiophthalmosuchus gracilirostris* | Nearly complete skull |
