# Supplementary figures and images for: The phylogenetics of Teleosauroidea (Crocodylomorpha, Thalattosuchia) and implications for their ecology and evolution
Source: PeerJ. 2020 Oct 8;8:e9808. doi: 10.7717/peerj.9808 (PMC7548081; doi:10.7717/peerj.9808)

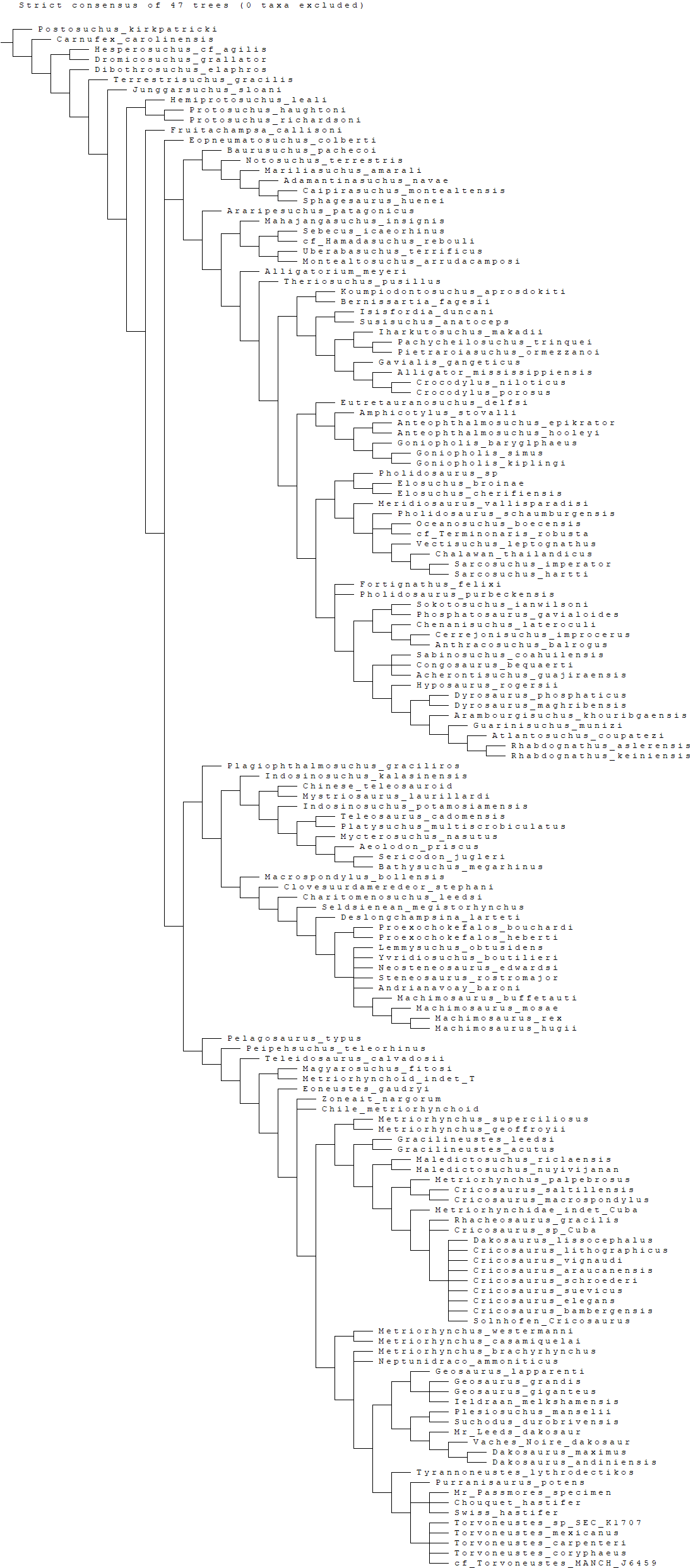

Supplement: Supplemental Information 7 [file peerj-08-9808-s007.png]
